# Artificial intelligence-driven prediction and validation of blood–brain barrier permeability and absorption, distribution, metabolism, excretion profiles in natural product research laboratory compounds

**DOI:** 10.37796/2211-8039.1474

**Published:** 2024-12-01

**Authors:** Jai-Sing Yang, Eddie TC. Huang, Ken YK. Liao, Da-Tian Bau, Shih-Chang Tsai, Chao-Jung Chen, Kuan-Wen Chen, Ting-Yuan Liu, Yu-Jen Chiu, Fuu-Jen Tsai

**Affiliations:** a Department of Medical Research, China Medical University Hospital, China Medical University, Taichung, Taiwan; b NVIDIA AI Technology Center, NVIDIA Corporation, USA; c Graduate Institute of Biomedical Sciences, China Medical University, Taichung, Taiwan; d Terry Fox Cancer Research Laboratory, Department of Medical Research, China Medical University Hospital, Taichung, Taiwan; e Department of Biological Science and Technology, China Medical University, Taichung, Taiwan; f Proteomics Core Laboratory, Department of Medical Research, China Medical University Hospital, Taichung, Taiwan; g Graduate Institute of Integrated Medicine, College of Chinese Medicine, Medical University, Taichung, Taiwan; h Genetics Generation Advancement Corporation, Molecular Science and Digital Innovation Center, Taipei, Taiwan; i Million-Person Precision Medicine Initiative, Department of Medical Research, China Medical University Hospital, Taichung, Taiwan; j Division of Plastic and Reconstructive Surgery, Department of Surgery, Taipei Veterans General Hospital, Taipei, Taiwan; k Department of Surgery, School of Medicine, National Yang Ming Chiao Tung University, Taipei, Taiwan; l School of Chinese Medicine, College of Chinese Medicine, China Medical University, Taichung, Taiwan; m China Medical University Children’s Hospital, Taichung, Taiwan; n Department of Medical Genetics, China Medical University Hospital, Taichung, Taiwan

**Keywords:** Large language model (LLM), Machine learning (ML), Blood-brain barrier (BBB), Absorption, distribution, metabolism, and excretion (ADME), Natural products research laboratories (NPRL)

## Abstract

**Introduction:**

Our previous research demonstrated that a large language model (LLM) based on the transformer architecture, specifically the MegaMolBART encoder with an XGBoost classifier, effectively predicts the blood–brain barrier (BBB) permeability of compounds. However, the permeability coefficients of compounds that can traverse this barrier remain unclear. Additionally, the absorption, distribution, metabolism, and excretion (ADME) characteristics of substances obtained from the Natural Product Research Laboratory (NPRL) at China Medical University Hospital (CMUH) have not yet been determined.

**Objectives:**

The study aims to investigate the pharmacokinetic ADME properties and BBB permeability coefficients of NPRL compounds.

**Materials and methods:**

A combined model using a transformer-based MegaMolBART encoder and XGBoost classifier was employed to predict BBB permeability. Machine learning (ML) tools from Discovery Studio were used to assess the ADME characteristics of the NPRL compounds. The CCK-8 assay was conducted to evaluate the cytotoxic effects of NPRL compounds on bEnd.3 brain endothelial cells after exposure to 10 μg/mL of the compounds. We assessed the permeability coefficient by subjecting bEnd.3 cell monolayers to the test compounds and measuring the permeability of FITC-dextran.

**Results:**

There were 4956 compounds that could cross the blood–brain barrier (BBB+) and 2851 that could not (BBB−) in the B3DB dataset that was utilized for training. A total of 2461 BBB+ and 2184 BBB− compounds were used in the NPRL-CMUH dataset for testing. The permeability coefficient of temozolomide (TMZ) and 21 other BBB + compounds exceeded 10 × 10^−7^ cm/s. Computational analysis revealed that NPRL compounds exhibited a variety of ADME characteristics.

**Conclusion:**

Computer-based predictions for the NPRL of CMUH compounds regarding their capacity to traverse the BBB are verified by the findings. Artificial intelligence (AI) prediction models have effectively identified the potential ADME characteristics of various compounds.

## 1. Introduction

Human health and well-being face substantial challenges due to neurological conditions, including Alzheimer’s disease (AD), Parkinson’s disease (PD), stroke (both ischemic and hemorrhagic), epilepsy, headaches, and brain tumors [1,2]. Identifying compounds capable of crossing the blood–brain barrier (BBB) and effectively targeting the central nervous system (CNS) remains a significant challenge in developing treatments for neurological disorders. This highly selective boundary restricts the passage of most substances into the brain [3,4]. To be considered viable therapeutic agent candidates for CNS diseases, compounds must demonstrate therapeutic efficacy and possess the ability to cross the BBB to reach their intended targets [4,5]. Candidates that can cross the BBB usually have certain traits: they are very attracted to fats, have a low molecular mass (usually less than 400–600 Da), do not bind well to plasma proteins very well, and do not break apart easily at physiological pH condition [6,7]. These strict requirements for BBB permeability make it challenging for larger molecules such as peptides, recombinant proteins, monoclonal antibodies, and gene therapies to traverse the barrier independently [8,9]. Furthermore, even when compounds capable of crossing the BBB are identified, their progression is often hindered by their suboptimal pharmacokinetic (PK) properties. These PK properties, which include absorption, distribution, metabolism, excretion, and toxicity (ADMET) profiles, contribute to high failure rates during new drug development [10,11]. In recent years, advancements in computational prediction models and artificial intelligence (AI) have significantly improved the efficiency of identifying compounds with desirable BBB permeability and ADMET characteristics [12–15].

Various AI research groups have developed predictive models for BBB penetration utilizing these tools to expedite CNS drug discovery [1,16–20]. For instance, Zhang *et al.* employed MolconnZ, MOE, and Dragon models to develop a quantitative structure–activity relationship (QSAR) model for BBB permeability, utilizing data from 159 compounds capable of passing through the BBB [21]. Similarly, Wang *et al.* applied six machine learning (ML) algorithms to categorize 2358 compounds using the scikit-learn framework (version 0.19.1) [22]. Miao *et al.* implemented POLY-Support Vector Machine (POLY-SVM), Sigmoid-Support Vector Machine (Sigmoid-SVM), K-Nearest Neighbor (KNN), Radial Basis Function-Support Vector Machine (RBF-SVM), Decision Tree (DT), and other deep learning techniques to predict BBB permeability [23]. Liang *et al.* investigated classification models to elucidate the structural features and transformation rules associated with compounds that can traverse the BBB [24]. Kumar *et al.* introduced “DeePred-BBB,” a deep learning-based model capable of predicting BBB permeability from simplified molecular input line entry system (SMILES) representations (Table 1) [25].

**Fig. 1:**
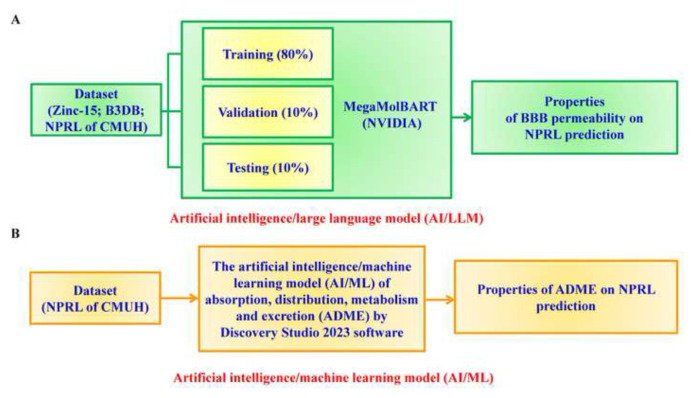
AI models for predicting BBB permeability and ADME properties. (A) A system that employs AI/LLM to predict how compounds cross the BBB. This system analyzes molecular properties using NVIDIA’s MegaMolBART decoder and a SMILES encoder that works with the B3DB, Zinc-15 and NPRL of CMUH datasets. (B) The ML model, implemented using the AI/ML model by Discovery Studio 2023 software, generates predictions of ADME properties.

Our previous research demonstrated the ability of the large language model (LLM) in the transformer-based MegaMolBART encoder with XGBoost classifier to predict BBB permeability across an extensive compound library, although permeability coefficients remain undetermined. Furthermore, the pharmacokinetic ADME characteristics of compounds from the Natural Product Research Laboratory (NPRL) at China Medical University Hospital (CMUH) have not been fully explored. This study’s objective was to use the FITC-dextran extravasation model to develop an *in vitro* model to assess BBB permeability and predict the pharmacokinetic (PK) behavior of NPRL-CMUH compounds. Additionally, this study integrates machine learning (ML) techniques (Fig. 1).

## 2. Methods

### 2.1. Cell culture

The bEnd.3 cells (immortalized mouse brain endothelial cells) from ATCC (Catalog no. CRL-2299; Manassas, VA, USA) were cultured in Dulbecco’s Modified Eagle’s Medium (DMEM) (American Type Culture Collection, Manassas, VA, USA) supplemented with 10% fetal bovine serum (R&D Systems, Minneapolis, MN, USA) and 100 μg/mL penicillin/streptomycin (Sigma–Aldrich, St. Louis, MO, USA) at 5% CO_2_ and 95% air [26,27].

### 2.2. *In vitro* permeability assay

The bEnd.3 cells (5.0 × 10^4^ cells/cm^2^) were cultured onto 24-well transwell inserts (pore size 0.4 μm; Corning Inc., Corning, NY, USA). After 72 h, the cells formed a confluent monolayer and were then treated with NPRL compounds (10 μg/mL) or dimethyl sulfoxide (DMSO) (1 μL) as control for 12 h. Following the treatment, the inserts and chambers were PBS washed, and the medium was replaced with phenol-red free medium. Subsequently, 10 kDa FITC-dextran (10 mg/mL; 10 μL; Sigma–Aldrich) was added to the upper inserts and incubated at 37 °C for 2 h. Fluorescence was measured in the upper and lower chambers using a fluorescence plate reader (SpectraMAX M3; Molecular Devices, Sunnyvale, CA, USA) at an excitation/emission wavelength of 490 nm/520 nm [27]. The permeability coefficient was calculated using the following formula:


$$\text{Permeability coefficient }(\text{cm}/\text{s}):{\text{P}}_{\text{dextran}}=({\text{RFU}}_{\text{lower}}/{\text{RFU}}_{\text{upper}})\;(\text{V})\;(1/\text{t})\;(1/\text{A})$$

RFU_lower_: relative fluorescent units in the lower well.
RFU_upper_: relative fluorescent units in the upper well.
V: volume of the bottom well.
T: time that the FITC-dextran was allowed to diffuse.
A: total surface area of the monolayer (cm^2^).

### 2.3. Cytotoxicity assay

The bEnd 3 cells (5 × 10^3^ cells/well) were seeded in 96-well plates, and then incubated for 24 h in a humidified incubator at 37 °C with 5% CO_2_. NPRL compounds (10 μg/mL) were then added to the culture media in the plates. The plates were incubated for an additional 20 h. Following this, 10 μL of CCK-8 solution was added to each well. The plates were incubated for 1–4 h, after which the absorbance was measured at 450 nm using an ELISA reader (detection: 450 nm, reference: 650 nm) [28].

### 2.4. *In silico* BBB permeability prediction

We employed the transformer-based Mega-MolBART encoder (NVIDIA, Version 1.0; URL: https://catalog.ngc.nvidia.com/orgs/nvidia/teams/clara/models/megamolbart) with an XGBoost classifier (URL: github.com/dmlc/xgboost) to predict BBB permeability [1]. This model was trained on the B3DB dataset and tested on the NPRL compound library. The MegaMolBART encoder processes the molecular structure information, while the XGBoost classifier uses this encoded information to make BBB permeability predictions. This approach allows efficient and accurate assessment of a molecule’s ability to cross the BBB based on its structural features.

### 2.5. *In silico* pharmacokinetic ADME properties prediction

*In silico* pharmacokinetic properties prediction studies utilized the ADME algorithm of the Discovery Studio 2023 machine learning model (ML) software (BIOVIA), accessible at (URL: https://www.3ds.com/products/biovia/discovery-studio/qsar-admet-predictive-toxicology) (Fig. 1). This software calculates various ADME properties, including aqueous solubility (predicting the solubility of each compound in water at 25 °C), BBB penetration, CYP2D6 binding (predicting cytochrome P450 2D6 enzyme inhibition), human intestinal absorption (HIA) after oral administration, plasma protein binding (PPB) (predicting the likelihood that a compound will be highly bound to carrier proteins in the blood), as well as AlogP98 and PSA_2D [29,30].

The training set for the aqueous solubility prediction model comprised 775 compounds with molecular weights ranging from 50 to 800. These compounds included alkanes, alkenes, alkynes, halogens, amines, alcohols, nitrogen-containing compounds, ketones, aldehydes, and organic acids. The plot of predicted versus experimental data yielded a linear regression of LogSw (25 °C, pH = 7.0) with R^2^ = 0.84 and a standard deviation (SD) of 0.87. The pre-test dataset included 34 compounds, resulting in regression statistics of R^2^ = 0.88 and SD = 0.79. Additionally, a validation test dataset of 1615 compounds from the PDR and Comprehensive Medicinal Chemistry database (CMC) produced an overall RMSE (SD) of 1.0 [31].

The BBB penetration model makes educated guesses about how much of a drug will enter the brain after oral administration by using data from over 800 compounds known to penetrate the BBB. This model employs a quantitative linear regression for predicting blood–brain penetration, accompanied by 95% and 99% confidence ellipses in the ADMET_PSA_2D and ADMET_AlogP98 planes [32].

The cytochrome P450_2D6 (CYP2D6) model predicts CYP2D6 enzyme inhibition based on 2D chemical structures. Using modified Bayesian learning [33], training set of 151 structurally different compounds with known CYP2D6 inhibition constants were used to make it. The AI/ML model was created using 182 compounds in the training dataset for human intestinal absorption. This model incorporates AlogP98 and 2D polar surface area (PSA_2D). Compounds with well-documented human intestinal absorption exhibit at least 90% absorption into the human bloodstream and typically fall within the 95% and 99% confidence ellipse regions [34].

The plasma protein binding model predicts whether a compound is likely to be highly bound (≥90% bound) to carrier proteins in the blood. The training dataset comprised 854 compounds divided into 329 binders and 525 non-binders. A modified Bayesian learning approach created a binary classification model [35,36].

### 2.6. Statistical analysis

The results are shown as the mean ± standard deviation (SD). Statistical analysis is performed using a one-way analysis of variance or Tukey’s test. Levels of significance are represented as ***P < 0.001, indicating statistical discrepancies between the control group and the NPRL-treated groups [37–39].

## 3. Results

### 3.1. *In silico* BBB permeability prediction

**Fig. 2:**
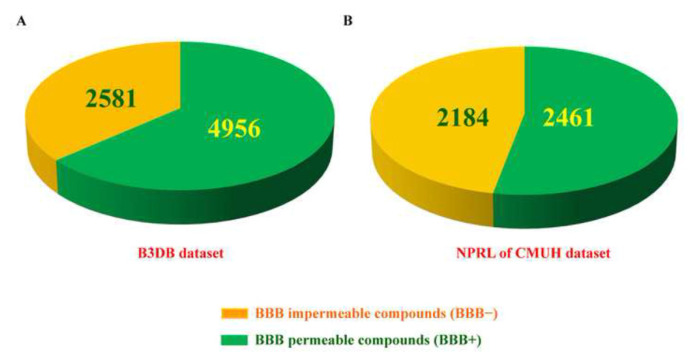
BBB permeability distribution of compounds in the training and testing datasets. This figure displays compounds that can (BBB+) and cannot (BBB−) penetrate the blood–brain barrier from two databases: the B3DB training dataset (A) and the NPRL-CMUH testing dataset (B). (A) In the B3DB dataset, the total number of BBB-impermeable (BBB−) compounds is 2581, while the number of BBB-permeable (BBB+) compounds is 4956. (B) In the NPRL of CMUH dataset, the total number of BBB-impermeable compounds is 2184, and the number of BBB-permeable compounds is 2461.

In our prior research, we utilized a LLM-based on the transformer-based MegaMolBART encoder with XGBoost classifier architecture to forecast the BBB permeability of compounds sourced from the NPRL. We validated these predictions by conducting *in vitro* experiments with human BBB spheroid cells and evaluating BBB integrity using liquid chromatography-tandem mass spectrometry (LC-MS/MS). Our findings highlighted the significant potential of this approach for advancing BBB permeability research in new drug discovery. The LLM of the transformer-based MegaMolBART encoder with XGBoost classifier exhibited strong predictive performance, as demonstrated by the composition of the training and testing datasets. The training dataset sourced from the B3DB database, comprised 4956 BBB-permeable (BBB+) and 2851 BBB-impermeable (BBB−) compounds (Fig. 2A). The NPRL testing dataset included 2461 BBB+ and 2184 BBB− compounds (Fig. 2B).

### 3.2. *In vitro* BBB permeability and cytotoxicity analysis

**Fig. 3:**
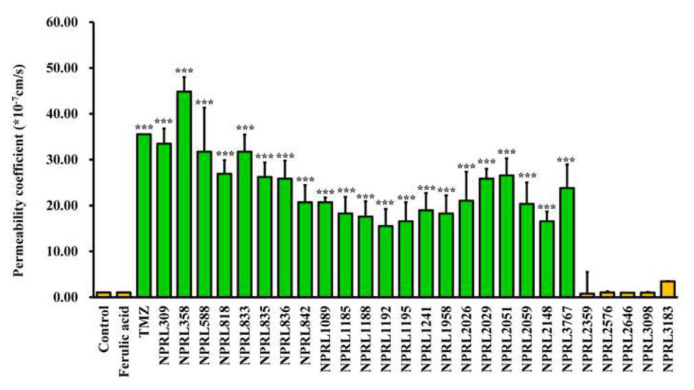
Permeability coefficients of NPRL compounds on bEnd.3 cells-FITC-dextran extravasation model. The chart illustrates the permeability coefficients (*10^−7^ cm/s) for various NPRL compounds, including control substances such as ferulic acid and TMZ. Each column in the graph represents a specific NPRL compound, highlighting notable differences in permeability. Asterisks are used to denote statistical significance, indicating which compounds exhibit significant permeability compared to the control group. NPRL compounds show a range of permeability effects, emphasizing their varying capacities to traverse the BBB. (***P < 0.001 relative to the respective vehicle-treated control groups).

The effect of NPRL compounds on endothelial cell permeability was evaluated using an *in vitro* blood–brain barrier (BBB) model consisting of confluent bEnd.3 monolayers grown in transwell chambers. As shown in Fig. 3, exposure to 10 μg/mL of various NPRL substances (NPRL309, NPRL358, NPRL588, NPRL818, NPRL833, NPRL835, NPRL836, NPRL842, NPRL1089, NPRL1185, NPRL1188, NPRL1192, NPRL1195, NPRL1241, NPRL1958, NPRL2026, NPRL2029, NPRL2051, NPRL2059, NPRL2148, and NPRL3767) led to a notable increase in monolayer permeability, as evidenced by the greater diffusion of 10 kDa FITC-dextran across the transwell membrane. Conversely, five BBB-impermeable compounds (NPRL2359, NPRL2576, NPRL2646, NPRL3098, and NPRL3183) reduced the monolayer permeability (Fig. 3).

**Fig. 4:**
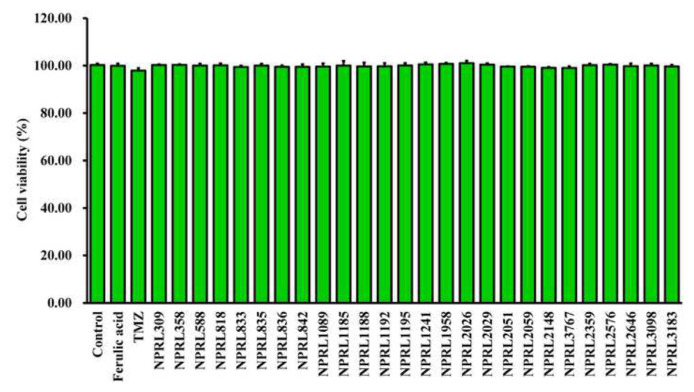
Cytotoxicity effects of NPRL compounds on bEnd.3 cells. bEnd.3 cells (5 × 10^3^ cells/mL/well) were seeded in 96-well plates and exposed to NPRL compounds (10 μg/mL) for 24 h. Cytotoxicity was assessed using the CCK-8 assay (n = 3).

To verify that the enhanced BBB permeability was not caused by the toxic effects on bEnd.3 cells, we assessed cell viability using CCK-8 assays. The results, depicted in Fig. 4, demonstrated that exposing bEnd.3 cells (10 μg/mL) of NPRL compounds had a negligible cytotoxic impact. This indicated that the observed permeability changes were not a result of compound-induced cell damage. These results corroborate our *in silico* predictions of the BBB permeability characteristics of the NPRL compound library.

### 3.3. *In silico* prediction of pharmacokinetic properties (ADME)

**Fig. 5:**
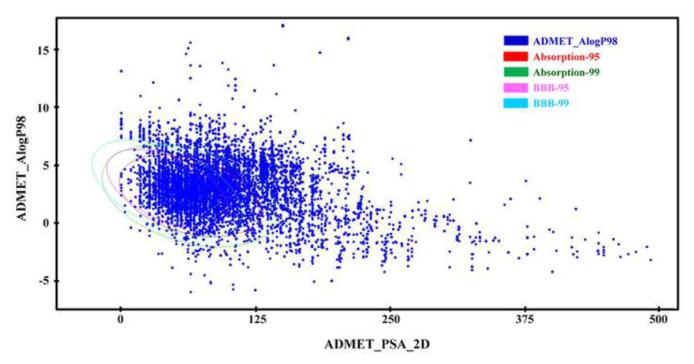
ADME plot for various NPRL compounds. Each compound’s 2D polar surface area (PSA_2D) is plotted against the calculated atom-type partition coefficient (ALogP98). The ellipse’s perimeter predicts good absorption without violating ADME properties.

To estimate the drug-like potential of the NPRL compounds, an *in silico* ADME study was conducted using Discovery Studio 2023 software within a ML model. This study predicted various pharmacokinetic properties, including intestinal absorption, compound solubility, metabolism by CYP2D6, plasma protein binding (PPB) capability, and blood–brain barrier (BBB) permeability, alongside the compound’s AlogP98a and PSA properties. Computer analysis of the ADME parameters showed that the NPRL compounds had different pharmacokinetic profiles, as shown in Fig. 5 and Table 2. Additional raw data are presented in Supplementary Table 1 (https://www.biomedicinej.com/cgi/editor.cgi?article=1474&window=additional_files&context=biomedicine). These results highlight the differences in ADME properties among the compounds, providing essential information for the future refinement of new drug development processes (see Table 2).

## 4. Discussion

*In vitro* and *in vivo* experiments and clinical trials typically yield the most accurate BBB permeability data. However, obtaining experimental BBB permeability data for numerous compounds, particularly across extensive libraries, is both time consuming and expensive [20]. To address these challenges, AI-based computational methods have been developed to assess BBB permeability, providing a crucial and strategic alternative [1,40]. These AI approaches offer significant benefits, including improved efficiency, reduced costs, and the ability to quickly process large volumes of data [41,42]. In contemporary research, most scientists have utilized the chemical characteristics of substances, such as molecular fingerprints and two-dimensional molecular descriptors, to develop predictive models. Various datasets and algorithms assess the likelihood of compounds penetrating the BBB [43–48]. This method speeds up the process of finding possible drugs for disorders of the CNS by allowing many compounds to be screened simultaneously and reducing the need for extensive experimental testing [43–48].

We used a LLM built on the transformer-based MegaMolBART encoder with XGBoost classifier architecture to try to guess how compounds from the NPRL would pass through the BBB (Fig. 1 up, and Fig. 2). We also performed *in vitro* experiments (Fig. 1 down and Fig. 3) to see how NPRL compounds affected bEnd.3 brain endothelial cells and found BBB permeability coefficients (Fig. 3). The findings revealed that cytotoxicity did not affect the observed BBB permeability of the NPRL compounds, as the bEnd.3 cells exhibited no notable toxic effects at the concentrations tested (Fig. 4). Our research demonstrated the effectiveness of integrating computational predictive models with laboratory-based validation to enhance BBB permeability research in drug development. It is essential to use both these methods to find promising drug candidates for further study, as this study shows that the LLM model can accurately predict BBB permeability without being affected by cytotoxic effects [1].

Researchers continue to face challenges in verifying the mechanisms of action (MOA) for substances that cross the BBB and in validating the accuracy of predictive models without extensive *in vivo* testing [23,49,50]. Several critical factors influence the ability of a compound to penetrate the BBB, including lipophilicity, molecular weight, shape, and charge [51–55]. Additionally, a substance’s permeability may be affected by its interactions with various cellular components, such as active or passive membrane transporters, metabolic enzymes, and efflux pumps [52,54,56,57]. Pathological conditions or therapeutic agents can also alter the effectiveness and distribution of compounds by modifying BBB permeability [58,59]. When utilizing *in vitro* models to assess BBB permeability, as in this study, it is essential to consider the physicochemical properties of the compounds, the presence of an unstirred water layer, and the characteristics of the filter [60,61]. These factors can significantly affect the permeability coefficients obtained in cell-based experiments.

The ADME parameters of NPRL compounds were analyzed to determine their pharmacokinetic characteristics (Fig. 5, Table 2 and Supplementary Table 1 (https://www.biomedicinej.com/cgi/editor.cgi?article=1474&window=additional_files&context=biomedicine)). These findings provide valuable insights for the development of pharmaceutical formulations, including those for oral and intravenous delivery, while also advancing the study of drug-metabolomics and pharmacokinetics (PK) and pharmacodynamics (PD). Our *in vitro* experimental results support computer-generated predictions regarding the BBB permeability of NPRL compounds, indicating that computational models can serve as reliable initial tools in new drug discovery. However, further *in vivo* research is needed to validate these predictions fully and to enhance our understanding of the mechanisms of action for potential clinical applications.

## 5. Conclusion

We conducted research that supports the numerous advantages of employing deep learning techniques to predict the permeability of the BBB. However, it is important to recognize that the current methods are still insufficient to completely elucidate the complex processes involved in drug-transport across the BBB. At present, we are unable to accurately differentiate between the therapeutic effects, adverse effects, and secondary outcomes resulting from a compound’s ability to penetrate the BBB, which has significant implications for pharmacological studies. Future research will focus on experimental investigations using *in vivo* animal models and cellular assays to address these knowledge deficits. The objective of these endeavors is to clarify the MOA and explore the potential therapeutic applications of the identified compounds. This type of research is crucial for improving the precision of predictions made by deep learning models and broadening our understanding of pharmacokinetics at the BBB.

## Supplementary Information







## Figures and Tables

**Table 1:** The ratio of BBB permeability in different compound libraries.

Dataset	BBB permeable compounds (BBB+)	BBB impermeable compounds (BBB−)	URL	Ref
LightBBB	5453	1709	https://bio.tools/lightbbb	[62]
DeePred-BBB	2607	998	https://github.com/12rajnish/DeePred-BBB	[25]
B3DB	4956	2851	https://github.com/theochem/B3DB	[4]
NPRL of CMUH	2461	2184	Non	[1]

**Table 2:** ADME analysis of NPRL (NPRL 1–30) using AI/ML module.

Name	Intestinal absorption level	Solubility Level	CYP2D6 score^a^	Plasma Protein Binding (PPB) score^a^	AlogP98^a^	PSA 2D^a^
NPRL 1	Good	Good	−3.777	−0.526	1.847	43.531
NPRL 2	Good	Low	−2.420	1.485	3.133	35.160
NPRL 3	Good	Low	−3.319	1.108	3.133	35.160
NPRL 4	Good	Low	−4.024	0.090	3.173	35.160
NPRL 5	Good	Low	−4.813	0.167	2.670	44.091
NPRL 6	Good	Low	−2.554	5.820	4.165	35.160
NPRL 7	Good	Low	−1.619	1.550	4.046	35.160
NPRL 8	Good	Low	−2.817	2.375	3.841	35.160
NPRL 9	Good	Low	−3.608	−0.330	2.614	35.160
NPRL 10	Good	Low	−2.626	2.861	3.502	35.160
NPRL 11	Good	Good	−4.328	−0.813	2.375	44.091
NPRL 12	Good	Low	−3.920	1.076	3.173	35.160
NPRL 13	Good	Low	−4.282	2.662	3.619	35.160
NPRL 14	Good	Low	−2.819	0.272	3.117	44.091
NPRL 15	Good	Low	−2.196	2.535	4.532	35.160
NPRL 16	Good	Low	−2.497	3.348	3.580	35.160
NPRL 17	Good	Good	−4.125	1.033	2.410	44.091
NPRL 18	Good	Good	−5.625	−1.216	1.644	76.791
NPRL 19	Good	Low	−3.382	3.386	3.619	35.160
NPRL 20	Good	Low	−4.100	−0.093	2.670	44.091
NPRL 21	Good	Very low, but possible	−1.772	5.748	4.875	61.391
NPRL 22	Good	Low	−2.668	0.703	3.609	44.091
NPRL 24	Good	Low	−3.394	3.250	4.327	35.160
NPRL 25	Good	Good	−4.541	2.225	0.660	122.783
NPRL 26	Good	Low	−2.424	3.252	3.481	106.042
NPRL 27	Good	Low	−0.764	4.043	3.502	52.461
NPRL 28	Moderate	Low	−3.281	2.271	4.757	106.042
NPRL 29	Good	Low	−6.166	−3.413	2.137	112.450
NPRL 30	Moderate	Low	−7.498	−5.892	1.272	134.455

a ALogP 98 score of <5 and PSA score of <140 indicates good absorption and cell permeability, PPB score of 2.226 or less reflect highly bound (90%) to plasma protein, CYP2D6 score of <0.162 indicate non inhibitor of CYP2D6.

## List of abbreviations

AI: Artificial intelligence
AD: Alzheimer’s disease
ADME: Absorption, distribution, metabolism, excretion
ADMET: Absorption, distribution, metabolism, excretion, and toxicity
BBB: Blood–brain barrier
bEnd.3: Brain endothelial cells bEnd.3
CCK-8: Cell counting kit-8
CMUH: China Medical University Hospital
CNS: Central nervous system
CYP2D6: Cytochrome P450_2D6 enzyme
DMEM: Dulbecco’s Modified Eagle medium
DMSO: Dimethyl sulfoxide
FBS: Fetal bovine serum
FITC-dextran: Fluorescein isothiocyanate-dextran
HIA: Human intestinal absorption
LC-MS/MS: liquid chromatography-tandem mass spectrometry
LLM: Large Language Model
ML: Machine learning
MOA: Mechanism of action
NPRL: Natural Product Research Laboratory
PD: Pharmacodynamics
PK: Pharmacokinetics
PPB: Plasma protein binding
PSA_2D: 2D polar surface area
QSAR: Quantitative structure–activity relationship
SMILES: Simplified molecular input line entry system
