# Supplementary material for: Artificial intelligence-driven prediction and validation of blood–brain barrier permeability and absorption, distribution, metabolism, excretion profiles in natural product research laboratory compounds
Source: Biomedicine (Taipei). 2024 Dec 1;14(4):82–91. doi: 10.37796/2211-8039.1474 (PMC11703399; doi:10.37796/2211-8039.1474)
Supplement: Supplementary file 1 [file bmed-14-04-082-s001.pdf]

Supplementary Table S1: Raw data of ADMET analysis on NPRL-CMUH dataset using AI/ML module.						
Name	Intestinal absorption level	Solubility level	CYP2D6 score	Plasma Protein Binding (PPB) score	AlogP98	PSA 2D
NPRL 1	Good	good	-3.777	-0.526	1.847	43.531
NPRL 2	Good	low	-2.420	1.485	3.133	35.160
NPRL 3	Good	low	-3.319	1.108	3.133	35.160
NPRL 4	Good	low	-4.024	0.090	3.173	35.160
NPRL 5	Good	low	-4.813	0.167	2.670	44.091
NPRL 6	Good	low	-2.554	5.820	4.165	35.160
NPRL 7	Good	low	-1.619	1.550	4.046	35.160
NPRL 8	Good	low	-2.817	2.375	3.841	35.160
NPRL 9	Good	low	-3.608	-0.330	2.614	35.160
NPRL 10	Good	low	-2.626	2.861	3.502	35.160
NPRL 11	Good	good	-4.328	-0.813	2.375	44.091
NPRL 12	Good	low	-3.920	1.076	3.173	35.160
NPRL 13	Good	low	-4.282	2.662	3.619	35.160
NPRL 14	Good	low	-2.819	0.272	3.117	44.091
NPRL 15	Good	low	-2.196	2.535	4.532	35.160
NPRL 16	Good	low	-2.497	3.348	3.580	35.160
NPRL 17	Good	good	-4.125	1.033	2.410	44.091
NPRL 18	Good	good	-5.625	-1.216	1.644	76.791
NPRL 19	Good	low	-3.382	3.386	3.619	35.160
NPRL 20	Good	low	-4.100	-0.093	2.670	44.091
NPRL 21	Good	very low, but possible	-1.772	5.748	4.875	61.391
NPRL 22	Good	low	-2.668	0.703	3.609	44.091
NPRL 24	Good	low	-3.394	3.250	4.327	35.160
NPRL 25	Good	good	-4.541	2.225	0.660	122.783
NPRL 26	Good	low	-2.424	3.252	3.481	106.042
NPRL 27	Good	low	-0.764	4.043	3.502	52.461
NPRL 28	Moderate	low	-3.281	2.271	4.757	106.042
NPRL 29	Good	low	-6.166	-3.413	2.137	112.450
NPRL 30	Moderate	low	-7.498	-5.892	1.272	134.455
NPRL 31	Moderate	low	-2.903	2.385	4.119	106.042
NPRL 32	Good	good	-6.629	-4.594	0.751	121.497
NPRL 34	Very poor	very low, but possible	-3.035	1.498	8.541	106.042
NPRL 35	Very poor	very low, but possible	-2.185	0.765	17.665	106.042
NPRL 36	Good	good	-9.081	-4.352	1.365	84.195
NPRL 37	Good	good	-5.952	-4.951	1.618	85.646
NPRL 38	Good	opti mal	-5.496	-5.750	0.470	60.003
NPRL 39	Good	opti mal	-6.649	-4.803	0.936	70.134
NPRL 40	Good	opti mal	-5.405	-4.196	-0.287	60.832
NPRL 41	Good	opti mal	-4.546	-2.831	-0.182	87.063
NPRL 42	Good	good	-0.983	-0.188	2.255	54.854
NPRL 43	Good	opti mal	-5.510	-3.899	-0.219	54.854
NPRL 44	Good	low	-1.105	2.283	3.260	73.277
NPRL 45	Good	low	-1.748	5.017	4.419	61.391
NPRL 46	Good	good	-6.551	-2.550	0.938	52.461
NPRL 47	Good	good	-6.617	-1.199	2.314	61.391
NPRL 48	Good	good	-9.466	-3.852	1.466	56.341
NPRL 49	Moderate	very low, but possible	-3.254	9.880	6.024	23.985
NPRL 50	Good	low	-0.690	5.468	4.473	35.160
NPRL 52	Good	low	-0.383	6.607	4.560	35.160
NPRL 53	Good	good	-4.069	-0.602	0.805	69.762
NPRL 55	Good	low	-3.672	-0.290	2.687	35.160
NPRL 56	Good	good	-7.592	-3.188	1.181	85.722
NPRL 59	Moderate	low	-3.222	2.727	4.059	106.042
NPRL 60	Good	good	-6.077	0.117	1.437	53.021
NPRL 61	Moderate	low	-3.222	3.073	4.059	106.042
NPRL 62	Good	low	-3.095	4.218	2.562	106.042
NPRL 64	Good	low	-3.095	4.218	2.562	106.042
NPRL 66	Moderate	opti mal	-1.560	0.517	-0.837	122.783
NPRL 67	Good	good	-8.775	0.050	1.331	95.844
NPRL 68	Good	good	-6.027	-1.858	0.690	79.561
NPRL 69	Good	opti mal	-6.181	-4.947	0.959	88.677
NPRL 70	Good	opti mal	-7.571	-2.764	1.185	76.791
NPRL 71	Good	low	-3.177	2.473	2.562	106.042
NPRL 72	Good	good	-3.649	1.783	2.534	53.021
NPRL 73	Good	good	-8.899	-0.059	1.331	95.844
NPRL 74	Good	good	-6.151	-1.619	0.690	79.561
NPRL 75	Good	opti mal	-4.700	-6.549	1.415	67.861
NPRL 76	Good	good	-5.654	-2.408	1.435	78.692
NPRL 77	Good	low	-1.970	3.415	4.763	78.692
NPRL 78	Good	good	-1.415	-2.184	2.313	43.531
NPRL 79	Good	good	-3.181	-1.311	2.109	43.531
NPRL 80	Good	low	-1.763	3.949	4.763	78.692
NPRL 82	Good	good	-4.581	-2.557	2.153	52.461
NPRL 85	Good	good	-5.663	-2.747	2.058	76.791

NPRL 87	Good	very low, but possible	-0.747	9.078	5.136	35.160
NPRL 88	Good	low	-2.382	3.946	4.144	35.160
NPRL 89	Good	good	-2.895	0.443	2.595	43.531
NPRL 91	Good	opti mal	-6.312	-3.495	0.888	55.417
NPRL 95	Good	good	-5.045	-2.859	2.041	85.722
NPRL 96	Good	good	-0.632	0.570	3.136	47.046
NPRL 97	Good	good	-4.543	-0.540	1.514	43.531
NPRL 98	Good	low	-1.029	4.947	4.939	69.762
NPRL 99	Good	good	-5.861	-3.287	1.435	78.692
NPRL 104	Good	good	-4.666	-2.469	1.384	52.461
NPRL 105	Good	low	-1.019	5.483	5.016	35.160
NPRL 106	Good	low	-3.119	-1.075	2.687	35.160
NPRL 107	Good	good	-1.439	-0.854	2.297	52.461
NPRL 108	Good	low	-1.673	5.065	4.590	35.160
NPRL 109	Good	good	-7.446	-3.727	1.299	85.722
NPRL 111	Good	low	-2.045	4.498	3.884	61.391
NPRL 112	Good	good	-7.249	-1.585	1.440	43.531
NPRL 113	Good	opti mal	-5.785	-2.578	2.077	58.931
NPRL 114	Good	low	-4.619	-0.459	2.399	78.692
NPRL 115	Poor	very low, but possible	-4.799	-0.444	5.558	140.084
NPRL 116	Good	very low, but possible	-4.150	0.763	4.578	82.207
NPRL 117	Moderate	very low, but possible	-4.661	1.851	5.152	87.622
NPRL 118	Good	low	-7.024	1.752	2.970	116.808
NPRL 119	Moderate	very low, but possible	-5.509	-0.909	3.980	113.853
NPRL 120	Good	low	-7.024	1.752	2.970	116.808
NPRL 121	Poor	Extremely low	-5.417	-1.061	6.535	113.853
NPRL 122	Good	opti mal	-6.312	-3.629	0.888	55.417
NPRL 124	Poor	very low, but possible	-5.991	-1.218	5.112	140.084
NPRL 125	Very poor	Extremely low	-5.898	3.447	7.364	113.243
NPRL 126	Poor	very low, but possible	-4.646	0.102	5.927	140.084
NPRL 127	Poor	very low, but possible	-5.603	0.713	6.538	140.084
NPRL 128	Poor	very low, but possible	-6.501	0.284	6.044	140.084
NPRL 129	Good	low	-6.284	-1.666	2.873	91.137
NPRL 130	Good	good	-5.625	-1.216	1.644	76.791
NPRL 131	Good	low	0.028	7.295	4.496	8.930
NPRL 133	Good	good	-2.464	0.135	2.650	67.699
NPRL 134	Good	good	-4.400	-4.772	1.828	47.046
NPRL 135	Good	low	-2.057	5.672	4.049	35.160
NPRL 136	Good	good	-3.060	3.018	2.560	76.791
NPRL 137	Good	good	-4.436	-2.695	2.375	29.745
NPRL 139	Good	good	-5.535	0.319	1.887	43.531
NPRL 140	Good	good	-7.690	-1.563	1.684	76.791
NPRL 141	Good	low	-1.849	2.139	3.525	8.930
NPRL 142	Good	opti mal	-2.583	-7.130	-0.041	47.046
NPRL 145	Good	low	-2.769	-2.174	3.042	76.791
NPRL 146	Good	opti mal	-5.010	-2.685	1.459	50.561
NPRL 149	Good	low	-1.844	6.760	4.590	35.160
NPRL 150	Good	opti mal	-5.510	-2.428	0.642	52.461
NPRL 151	Good	good	-4.115	-2.088	0.688	26.230
NPRL 152	Good	good	-2.552	-4.052	1.586	67.861
NPRL 153	Good	low	-0.749	3.604	3.133	55.976
NPRL 154	Good	good	0.504	3.497	2.928	55.976
NPRL 155	Good	low	-1.260	3.533	3.856	35.160
NPRL 156	Good	low	-2.497	3.309	4.532	35.160
NPRL 157	Good	good	-5.934	-0.568	2.748	76.791
NPRL 158	Good	good	-6.630	1.954	2.130	76.791
NPRL 159	Good	good	-3.415	-0.634	2.383	67.861
NPRL 164	Good	good	-5.343	-2.765	1.401	26.230
NPRL 165	Good	good	-1.083	-0.436	3.502	47.046
NPRL 166	Good	good	-7.249	-2.411	1.440	43.531
NPRL 168	Good	very low, but possible	-2.362	4.024	4.110	23.985
NPRL 169	Good	good	-3.258	-1.035	2.385	35.160
NPRL 170	Good	low	-1.043	4.183	3.988	52.461
NPRL 171	Good	very low, but possible	-1.491	3.244	4.612	35.160
NPRL 172	Good	low	-1.148	4.316	4.117	55.976
NPRL 173	Good	low	-2.298	1.708	3.271	35.160
NPRL 174	Good	low	-3.365	2.186	3.117	76.791
NPRL 175	Poor	low	-5.752	-3.969	4.282	143.288
NPRL 177	Moderate	very low, but possible	1.278	6.805	5.820	15.055
NPRL 178	Good	low	-0.322	1.471	3.055	73.277
NPRL 179	Good	good	-3.219	0.302	2.685	29.745
NPRL 180	Good	very low, but possible	0.576	7.600	5.091	35.160
NPRL 181	Good	low	-0.276	3.118	3.778	52.461
NPRL 182	Good	low	-0.097	-0.263	3.577	47.046
NPRL 184	Good	good	-5.170	-6.088	1.649	79.064
NPRL 185	Good	good	-2.596	-3.117	1.837	52.461

NPRL 186	Good	low	-2.446	-0.289	3.201	55.976
NPRL 187	Good	good	-2.392	0.404	2.333	43.531
NPRL 188	Good	low	-1.178	4.537	4.120	35.160
NPRL 189	Good	good	-3.791	-5.551	1.932	47.046
NPRL 190	Good	very low, but possible	0.507	7.586	5.577	35.160
NPRL 191	Good	good	-4.392	-1.264	1.857	43.531
NPRL 193	Good	low	-0.744	1.542	3.067	73.277
NPRL 194	Good	very low, but possible	0.316	6.344	5.583	35.160
NPRL 195	Good	good	-2.734	1.859	2.631	76.791
NPRL 196	Good	very low, but possible	-2.006	5.042	4.606	35.160
NPRL 197	Good	good	-5.946	-0.685	1.401	43.531
NPRL 199	Good	low	-3.788	2.893	3.929	35.160
NPRL 201	Very poor	too soluble	-2.642	-5.420	-2.314	76.232
NPRL 202	Very poor	very low, but possible	-6.417	-0.974	5.332	166.315
NPRL 203	Poor	very low, but possible	-4.816	0.744	6.263	140.084
NPRL 204	Good	opti mal	-6.197	-3.192	0.199	60.832
NPRL 205	Good	good	-3.872	-0.874	1.636	53.021
NPRL 206	Good	good	-5.796	-0.214	1.644	76.791
NPRL 207	Good	low	-4.385	2.368	3.619	35.160
NPRL 208	Very poor	Extremely low	-6.050	-0.149	7.025	140.084
NPRL 209	Very poor	very low, but possible	-6.314	-2.007	3.915	166.624
NPRL 210	Very poor	very low, but possible	-5.801	-0.277	4.953	160.900
NPRL 211	Good	good	-3.631	1.350	2.384	53.021
NPRL 212	Good	good	-2.740	-1.426	2.057	44.091
NPRL 213	Poor	very low, but possible	-4.970	0.557	5.558	140.084
NPRL 214	Very poor	low	-7.353	-2.562	4.846	166.315
NPRL 215	Poor	very low, but possible	-6.050	-0.490	6.302	140.084
NPRL 216	Very poor	very low, but possible	-7.201	-1.010	5.520	184.175
NPRL 217	Good	good	-0.684	-1.327	3.000	55.976
NPRL 218	Good	opti mal	-4.669	0.517	0.685	61.391
NPRL 219	Good	good	-5.099	-2.551	1.128	52.461
NPRL 220	Good	good	-5.363	-1.510	1.887	43.531
NPRL 221	Good	good	-6.653	0.306	2.130	76.791
NPRL 222	Poor	very low, but possible	-6.050	-0.490	6.302	140.084
NPRL 223	Very poor	very low, but possible	-6.452	-0.673	4.467	160.900
NPRL 225	Poor	Extremely low	-3.806	1.250	6.545	140.084
NPRL 227	Very poor	very low, but possible	-5.127	2.401	6.990	166.315
NPRL 228	Poor	very low, but possible	-4.516	0.589	6.004	140.084
NPRL 230	Poor	Extremely low	-2.494	3.688	5.950	113.853
NPRL 231	Very poor	Extremely low	-6.126	2.534	7.076	140.084
NPRL 232	Very poor	very low, but possible	-5.127	2.401	6.990	166.315
NPRL 235	Poor	very low, but possible	-5.455	-0.125	4.799	149.014
NPRL 237	Good	low	-1.812	0.361	3.426	44.091
NPRL 239	Poor	very low, but possible	-4.666	-1.244	5.541	149.014
NPRL 240	Poor	very low, but possible	-5.260	2.769	6.590	140.084
NPRL 242	Good	low	-1.935	4.538	4.319	26.230
NPRL 245	Poor	very low, but possible	-5.991	-1.218	5.111	140.084
NPRL 246	Poor	very low, but possible	-6.864	-0.791	5.598	140.084
NPRL 248	Poor	very low, but possible	-6.571	-2.522	5.095	149.014
NPRL 249	Poor	very low, but possible	-5.040	-0.894	6.470	140.084
NPRL 250	Poor	very low, but possible	-5.858	-1.325	5.095	149.014
NPRL 252	Poor	very low, but possible	-6.388	-1.407	4.799	149.014
NPRL 258	Poor	Extremely low	-6.012	0.691	6.752	140.084
NPRL 259	Poor	very low, but possible	-5.346	2.131	6.474	140.084
NPRL 260	Good	good	-4.409	0.179	2.100	76.791
NPRL 262	Poor	Extremely low	-6.817	1.087	6.876	140.084
NPRL 263	Very poor	low	-7.353	-2.562	4.846	166.315
NPRL 264	Poor	very low, but possible	-5.603	0.713	5.816	140.084
NPRL 265	Poor	very low, but possible	-5.603	0.713	5.816	140.084
NPRL 268	Very poor	Extremely low	-5.707	-1.965	4.664	166.624
NPRL 270	Poor	Extremely low	-6.095	1.511	6.749	140.084
NPRL 271	Very poor	very low, but possible	-6.282	-2.612	5.293	166.315
NPRL 272	Good	good	-4.474	0.912	2.090	76.791
NPRL 274	Poor	very low, but possible	-6.837	-1.263	5.856	140.084
NPRL 276	Good	good	-6.790	1.190	1.048	34.601
NPRL 277	Good	opti mal	-2.108	0.254	0.813	51.902
NPRL 281	Good	opti mal	-4.606	-5.500	0.882	90.578
NPRL 282	Good	opti mal	-6.443	-3.711	0.642	52.461
NPRL 283	Good	low	-3.528	-0.047	2.980	44.091
NPRL 284	Good	low	-0.510	5.774	3.458	52.461
NPRL 285	Good	opti mal	-4.536	-2.972	0.892	95.993
NPRL 286	Good	good	-1.444	0.163	2.357	47.046
NPRL 287	Good	good	-4.617	-1.800	1.827	61.391
NPRL 288	Good	very low, but possible	1.415	2.835	4.957	26.230
NPRL 289	Good	good	-3.696	-5.436	1.667	52.461
NPRL 290	Good	opti mal	-4.551	-3.078	1.124	69.762

NPRL 291	Good	opti mal	-5.238	-3.054	0.211	55.417
NPRL 293	Good	good	-2.698	0.850	2.766	44.091
NPRL 294	Good	opti mal	-4.991	-5.880	1.115	64.347
NPRL 295	Good	opti mal	-2.855	-2.223	1.652	76.791
NPRL 296	Good	good	-5.081	-1.737	1.456	104.923
NPRL 297	Good	good	-2.566	-3.172	1.867	44.091
NPRL 298	Moderate	very low, but possible	-0.513	2.011	6.047	26.230
NPRL 299	Moderate	too soluble	-4.370	-4.875	-0.532	35.160
NPRL 300	Good	opti mal	-4.268	-3.462	1.314	55.976
NPRL 301	Good	good	-4.633	-1.766	1.446	99.508
NPRL 302	Good	good	-3.395	0.232	2.031	70.321
NPRL 305	Good	low	-2.248	3.368	3.641	44.091
NPRL 306	Good	low	-1.375	6.160	4.033	44.091
NPRL 308	Good	good	1.088	1.694	4.006	34.601
NPRL 309	Good	good	-3.433	-1.851	1.811	55.976
NPRL 310	Good	opti mal	-3.537	-6.735	1.088	67.861
NPRL 311	Good	low	-2.931	2.981	3.822	97.112
NPRL 313	Good	good	-1.931	-2.004	2.711	73.277
NPRL 316	Good	good	-1.008	-4.052	3.258	41.631
NPRL 318	Good	good	-2.564	-2.786	2.053	35.160
NPRL 320	Good	good	-4.041	-4.641	1.608	26.230
NPRL 322	Good	good	0.684	-0.307	3.091	47.046
NPRL 325	Good	very low, but possible	-1.010	4.351	4.934	78.692
NPRL 329	Good	low	-0.560	6.459	4.550	35.160
NPRL 331	Good	low	-1.944	2.563	3.579	35.160
NPRL 332	Good	good	-6.389	4.824	3.843	91.137
NPRL 333	Moderate	low	-2.753	2.441	5.715	76.232
NPRL 334	Good	good	-6.288	4.913	3.520	96.552
NPRL 335	Good	good	-3.355	0.192	2.533	99.457
NPRL 336	Good	good	-10.838	3.317	4.272	90.975
NPRL 337	Moderate	low	-3.983	6.610	5.445	73.277
NPRL 338	Good	low	-5.859	3.416	5.031	56.373
NPRL 339	Good	good	-5.816	4.790	3.953	96.552
NPRL 340	Very poor	opti mal	-7.098	-1.185	8.543	133.690
NPRL 341	Good	good	-9.691	3.165	3.575	90.975
NPRL 342	Good	good	-6.043	-2.176	2.606	98.085
NPRL 343	Good	low	-5.369	4.770	4.222	53.021
NPRL 344	Good	low	-3.281	1.945	4.519	79.252
NPRL 345	Good	good	-8.783	-1.024	3.516	82.604
NPRL 346	Good	good	-8.771	0.603	2.810	111.628
NPRL 347	Good	good	-6.428	6.309	4.329	91.137
NPRL 348	Good	good	-7.775	4.009	2.631	108.438
NPRL 349	Moderate	good	-10.623	1.130	3.821	114.746
NPRL 350	Good	good	-5.482	4.286	3.046	87.622
NPRL 351	Good	low	-6.381	6.162	4.865	70.321
NPRL 352	Good	low	-6.178	5.378	5.068	70.321
NPRL 353	Moderate	low	-5.124	6.598	5.981	70.321
NPRL 354	Very poor	very low, but possible	-5.101	6.192	7.806	70.321
NPRL 355	Poor	low	-1.974	12.374	5.771	87.622
NPRL 356	Good	low	-3.857	3.526	4.094	53.021
NPRL 357	Good	low	-4.574	4.072	3.636	53.021
NPRL 358	Good	low	-3.446	5.290	4.113	51.851
NPRL 359	Good	low	-5.011	5.336	5.072	70.321
NPRL 360	Good	good	-4.698	2.940	3.354	52.461
NPRL 361	Moderate	low	-3.848	6.482	6.014	52.461
NPRL 362	Good	low	-6.282	3.646	5.160	78.692
NPRL 363	Good	low	-1.310	1.676	4.718	76.232
NPRL 364	Good	good	-3.109	1.040	2.903	76.232
NPRL 365	Good	low	-0.953	3.641	4.718	76.232
NPRL 366	Good	low	-2.092	6.748	5.169	52.461
NPRL 367	Good	good	-5.445	0.621	2.813	73.526
NPRL 368	Good	good	-6.166	4.936	3.254	79.252
NPRL 369	Good	low	-5.360	5.404	5.388	70.321
NPRL 370	Moderate	low	-5.631	3.786	5.223	88.182
NPRL 371	Good	low	-3.851	4.298	4.127	35.160
NPRL 372	Good	low	-4.851	5.187	5.030	53.021
NPRL 373	Good	low	-4.553	5.187	4.562	53.021
NPRL 374	Good	low	-2.911	5.391	4.127	35.160
NPRL 375	Good	good	-3.341	1.354	3.676	58.931
NPRL 378	Good	good	-3.971	1.204	3.218	58.931
NPRL 379	Good	good	-3.481	-0.444	3.434	79.747
NPRL 380	Good	low	-5.481	4.164	3.023	102.946
NPRL 381	Good	good	-1.910	1.931	2.750	58.931
NPRL 382	Good	good	-7.413	5.219	4.103	96.552
NPRL 383	Good	low	-8.578	3.665	3.974	104.364
NPRL 384	Good	good	-3.837	0.681	3.660	100.562

NPRL 385	Good	low	-7.919	4.155	3.974	104.364
NPRL 386	Good	low	-5.914	5.294	4.067	60.832
NPRL 387	Good	good	-7.072	1.421	3.220	90.578
NPRL 388	Good	good	-3.059	2.925	4.144	58.931
NPRL 389	Good	good	-6.715	3.386	3.220	90.578
NPRL 390	Moderate	good	-7.383	3.261	2.764	122.224
NPRL 391	Good	good	-8.004	5.494	3.277	104.364
NPRL 392	Moderate	low	-8.293	4.103	5.022	104.364
NPRL 394	Good	low	-4.276	5.131	4.111	44.091
NPRL 395	Good	low	-4.065	5.927	4.578	44.091
NPRL 396	Good	low	-4.065	5.927	4.578	44.091
NPRL 397	Good	good	-2.839	0.419	3.902	79.747
NPRL 398	Good	good	-2.839	0.517	3.902	79.747
NPRL 399	Poor	good	-8.646	3.257	3.881	147.895
NPRL 400	Good	good	-6.853	0.532	3.582	82.937
NPRL 401	Good	low	-5.811	0.965	4.320	79.585
NPRL 403	Good	good	-1.148	-2.874	3.191	62.135
NPRL 404	Good	low	-5.505	1.197	3.441	79.585
NPRL 405	Moderate	low	-5.249	3.145	5.680	69.762
NPRL 406	Good	good	-3.456	-1.815	3.177	50.250
NPRL 407	Good	opti mal	-5.092	-0.662	2.678	41.569
NPRL 408	Good	good	-3.411	0.209	3.434	79.747
NPRL 409	Good	good	-3.820	1.274	2.731	76.232
NPRL 410	Good	low	-5.270	0.921	4.741	90.578
NPRL 411	Good	low	-4.995	1.775	4.043	90.578
NPRL 413	Moderate	low	-5.333	2.127	5.438	90.578
NPRL 414	Good	good	-4.330	-2.763	2.935	71.065
NPRL 415	Good	opti mal	-4.797	-0.828	2.436	62.384
NPRL 416	Poor	very low, but possible	-3.328	4.893	6.784	35.160
NPRL 417	Good	good	-9.496	2.712	2.459	111.069
NPRL 418	Good	good	-4.589	-2.149	2.355	39.822
NPRL 419	Good	good	-6.785	-3.018	2.942	70.903
NPRL 420	Good	opti mal	-8.164	-1.365	2.443	62.222
NPRL 421	Good	low	-2.788	2.962	5.230	47.046
NPRL 425	Moderate	low	-3.164	0.708	6.258	55.417
NPRL 426	Good	good	-9.413	0.740	3.326	72.966
NPRL 427	Good	good	-6.610	-0.923	3.429	56.225
NPRL 429	Good	good	-4.005	-3.466	3.459	50.250
NPRL 430	Good	good	-2.794	-2.713	3.397	50.250
NPRL 431	Good	low	-2.875	2.218	4.567	47.046
NPRL 432	Good	good	-3.720	-1.248	3.728	59.328
NPRL 433	Good	low	-7.217	1.423	3.923	86.503
NPRL 434	Moderate	low	-1.610	5.079	5.649	17.300
NPRL 435	Good	good	-3.242	0.792	2.811	62.284
NPRL 436	Good	good	-4.050	-0.568	2.605	71.741
NPRL 437	Good	good	-8.281	2.778	3.109	107.716
NPRL 438	Good	good	-2.580	0.517	3.838	76.232
NPRL 439	Good	good	-3.500	0.599	4.127	100.562
NPRL 440	Good	low	-3.670	0.789	4.268	38.116
NPRL 441	Good	good	-3.129	1.419	4.611	58.931
NPRL 442	Good	good	-3.702	1.918	4.064	64.347
NPRL 443	Moderate	low	-8.086	5.250	4.442	104.364
NPRL 444	Poor	good	-8.733	4.263	4.349	147.895
NPRL 445	Good	good	-8.748	-0.430	3.546	72.966
NPRL 446	Good	good	-4.420	1.075	4.149	50.250
NPRL 447	Good	low	-4.483	2.289	4.375	38.365
NPRL 448	Moderate	low	-8.343	4.240	4.947	104.364
NPRL 449	Good	low	-8.678	6.186	5.023	69.762
NPRL 451	Good	low	-3.986	6.058	5.262	52.461
NPRL 452	Good	low	-2.900	3.989	5.036	64.347
NPRL 453	Good	low	-2.942	2.620	4.811	76.232
NPRL 454	Good	low	-4.157	4.036	4.289	52.461
NPRL 455	Good	low	-0.905	3.329	4.123	38.116
NPRL 456	Good	low	-4.234	6.000	4.272	60.832
NPRL 457	Moderate	very low, but possible	-3.427	4.113	6.038	47.046
NPRL 459	Moderate	very low, but possible	-9.079	6.829	6.187	69.762
NPRL 460	Good	good	-3.206	0.699	3.198	76.232
NPRL 461	Moderate	low	-3.881	2.609	5.256	97.048
NPRL 462	Good	good	-10.609	2.731	3.263	99.905
NPRL 463	Good	good	-9.635	1.075	3.535	99.905
NPRL 464	Moderate	low	-9.072	0.718	4.136	111.694
NPRL 466	Very poor	very low, but possible	-8.358	3.953	7.145	104.923
NPRL 467	Poor	very low, but possible	-9.033	6.070	6.090	87.063
NPRL 468	Poor	good	-6.941	4.862	4.290	122.033
NPRL 469	Poor	low	-6.594	3.208	5.407	122.033
NPRL 470	Moderate	low	-5.421	6.588	5.952	70.321

NPRL 471	Poor	low	-5.538	6.712	6.857	70.321
NPRL 472	Moderate	low	-6.995	4.205	5.093	96.552
NPRL 473	Poor	low	-5.783	5.850	5.817	96.552
NPRL 474	Good	good	-4.146	-1.923	1.868	64.347
NPRL 477	Good	good	-4.658	0.673	2.596	43.531
NPRL 478	Good	good	-3.297	1.390	2.316	69.762
NPRL 479	Good	good	-6.203	2.806	1.387	78.133
NPRL 481	Good	good	-4.626	-0.378	2.370	55.417
NPRL 482	Good	good	-3.423	-5.060	1.642	76.232
NPRL 484	Good	opti mal	-4.414	-7.337	0.446	67.551
NPRL 491	Good	good	-5.701	-0.450	1.162	90.018
NPRL 492	Good	low	0.943	6.261	3.878	39.041
NPRL 493	Good	very low, but possible	-0.860	1.665	4.646	39.041
NPRL 495	Good	very low, but possible	1.113	6.974	5.740	30.414
NPRL 496	Good	very low, but possible	-0.178	3.934	4.383	39.041
NPRL 497	Good	low	-0.683	1.819	3.881	47.971
NPRL 498	Good	low	0.526	3.052	3.881	47.971
NPRL 499	Good	very low, but possible	-0.032	2.647	4.384	39.041
NPRL 501	Good	very low, but possible	-0.214	3.527	4.840	39.041
NPRL 502	Good	very low, but possible	-0.860	1.574	4.646	39.041
NPRL 503	Good	very low, but possible	-0.499	2.558	4.646	39.041
NPRL 504	Good	very low, but possible	-0.822	4.534	4.988	39.041
NPRL 505	Good	very low, but possible	-1.488	3.662	4.988	39.041
NPRL 506	Good	very low, but possible	-1.173	1.946	5.251	39.041
NPRL 507	Good	very low, but possible	-0.641	3.080	5.251	39.041
NPRL 508	Good	very low, but possible	-0.884	3.472	5.444	39.041
NPRL 509	Good	low	-0.068	2.341	4.502	39.041
NPRL 510	Good	low	0.297	3.675	4.486	47.971
NPRL 511	Good	very low, but possible	-0.676	3.248	4.988	39.041
NPRL 512	Good	low	-1.083	2.237	4.486	47.971
NPRL 513	Good	low	-2.642	-5.223	1.883	61.939
NPRL 514	Good	good	-5.678	-7.117	1.234	79.240
NPRL 516	Good	good	-7.873	-6.365	1.351	53.009
NPRL 522	Good	low	-2.604	2.650	2.509	61.477
NPRL 524	Good	good	-2.044	-4.111	1.352	53.009
NPRL 526	Good	good	-6.734	-2.178	0.100	42.393
NPRL 527	Good	low	-4.153	1.946	2.876	42.840
NPRL 529	Good	low	-1.898	4.758	3.207	61.477
NPRL 530	Good	low	-1.435	2.330	2.827	42.840
NPRL 532	Good	good	-8.466	-2.485	1.335	61.939
NPRL 534	Good	good	-10.752	-2.300	1.335	61.939
NPRL 535	Good	good	-5.123	-3.093	1.556	53.009
NPRL 536	Good	good	-6.909	-1.891	1.934	47.971
NPRL 537	Good	good	-10.990	-4.561	1.087	47.971
NPRL 540	Good	low	-1.415	0.432	2.948	62.260
NPRL 541	Good	low	-6.096	0.152	2.825	92.062
NPRL 543	Good	low	-3.513	1.772	3.961	74.761
NPRL 544	Good	low	-0.074	0.208	3.728	48.530
NPRL 545	Good	low	-0.952	1.017	2.451	56.901
NPRL 546	Good	low	-5.283	0.704	2.969	65.831
NPRL 548	Good	good	-8.045	-0.394	1.896	100.432
NPRL 551	Good	good	-7.133	-10.285	2.151	97.972
NPRL 552	Good	good	0.770	1.754	3.140	35.160
NPRL 553	Good	good	-0.254	-1.748	2.747	55.976
NPRL 554	Good	good	-2.457	-2.067	2.505	76.791
NPRL 555	Good	low	0.844	1.251	4.220	76.791
NPRL 556	Good	low	0.335	0.100	3.230	52.461
NPRL 557	Good	low	2.236	2.176	4.480	59.491
NPRL 558	Very poor	very low, but possible	-1.855	2.430	8.144	73.836
NPRL 559	Good	good	-3.436	2.272	2.948	100.626
NPRL 560	Moderate	low	-3.733	2.003	5.331	94.652
NPRL 561	Good	low	2.906	0.428	4.462	55.976
NPRL 562	Good	low	0.790	0.967	4.671	53.021
NPRL 564	Good	opti mal	-3.847	-1.776	0.997	73.836
NPRL 565	Good	good	-4.866	-3.842	1.206	69.762
NPRL 566	Moderate	very low, but possible	2.314	2.644	6.531	26.790
NPRL 568	Good	good	-3.445	-0.845	2.282	35.160
NPRL 570	Very poor	Extremely low	-5.944	5.527	13.545	73.277
NPRL 571	Moderate	very low, but possible	-0.124	3.459	6.664	31.865
NPRL 572	Very poor	Extremely low	-0.184	3.164	8.630	29.745
NPRL 573	Very poor	Extremely low	0.167	3.620	10.949	17.860
NPRL 574	Good	opti mal	-8.225	-7.823	-0.437	118.422
NPRL 576	Poor	very low, but possible	-5.657	4.964	6.005	98.948
NPRL 577	Very poor	Extremely low	-2.218	4.364	8.472	64.347
NPRL 578	Very poor	Extremely low	-2.218	5.764	8.091	64.347
NPRL 579	Very poor	Extremely low	-2.286	4.539	8.025	64.347

NPRL 580	Very poor	Extremely low	-3.869	14.037	12.486	64.347
NPRL 582	Poor	very low, but possible	-5.657	4.964	6.005	98.948
NPRL 583	Very poor	very low, but possible	-5.035	3.414	7.142	85.162
NPRL 584	Moderate	low	-4.830	4.281	4.237	104.923
NPRL 585	Moderate	low	-5.710	3.685	4.237	104.923
NPRL 587	Poor	low	-5.307	5.090	5.539	94.092
NPRL 588	Good	low	-4.086	-0.358	3.053	55.814
NPRL 589	Good	good	-5.166	-2.561	1.966	43.531
NPRL 590	Good	good	-2.355	0.615	2.227	73.277
NPRL 591	Good	opti mal	-3.605	-0.757	1.649	55.976
NPRL 592	Good	low	-1.996	2.274	4.087	35.160
NPRL 594	Good	low	-4.563	-1.068	3.780	38.513
NPRL 597	Good	low	-0.859	3.080	4.345	35.160
NPRL 598	Good	low	-0.536	2.373	4.332	35.160
NPRL 599	Good	low	-1.132	1.797	3.153	35.160
NPRL 601	Good	low	-0.874	1.849	3.880	35.160
NPRL 602	Good	low	-1.087	3.193	3.502	35.160
NPRL 603	Good	low	-3.398	-0.223	4.038	38.513
NPRL 614	Good	good	-4.035	0.091	2.183	61.391
NPRL 620	Good	good	-3.051	-1.123	2.348	47.046
NPRL 622	Good	good	-5.890	1.656	1.647	67.537
NPRL 625	Good	low	-5.786	3.604	2.975	76.467
NPRL 626	Good	low	-4.059	1.937	3.249	76.467
NPRL 627	Good	good	-2.489	1.038	2.119	55.976
NPRL 630	Good	low	-2.274	0.627	3.274	76.467
NPRL 631	Good	low	-3.714	-3.360	3.543	91.522
NPRL 632	Good	low	-5.343	3.320	3.710	70.321
NPRL 636	Good	good	-2.010	0.262	2.530	55.976
NPRL 640	Good	low	-2.136	0.044	3.621	35.160
NPRL 641	Good	good	-1.508	1.617	2.996	55.976
NPRL 642	Good	low	-1.066	2.113	4.194	35.160
NPRL 644	Good	good	-1.206	2.218	3.311	55.976
NPRL 653	Good	low	-1.974	0.611	3.244	35.160
NPRL 656	Good	low	1.299	3.322	4.726	37.491
NPRL 657	Good	low	-5.578	3.792	3.324	76.467
NPRL 658	Good	low	-3.851	1.630	3.598	76.467
NPRL 659	Good	good	-3.208	-3.759	2.063	76.319
NPRL 660	Good	low	-3.560	-3.172	3.892	91.522
NPRL 661	Good	very low, but possible	3.071	4.612	5.421	35.160
NPRL 662	Good	low	-2.455	1.167	4.357	58.096
NPRL 663	Good	low	-3.302	-0.511	3.543	46.422
NPRL 664	Good	very low, but possible	3.279	5.437	5.679	35.160
NPRL 665	Good	good	-3.919	0.838	3.257	38.513
NPRL 667	Good	good	-4.212	0.244	2.422	38.513
NPRL 669	Good	good	-1.341	0.253	2.928	47.046
NPRL 670	Good	low	-0.572	4.620	4.282	44.091
NPRL 671	Good	good	-4.234	-2.935	1.797	47.046
NPRL 675	Good	low	-0.440	3.004	4.478	35.160
NPRL 676	Good	low	0.726	3.850	4.737	35.160
NPRL 677	Good	low	0.140	3.578	4.931	35.160
NPRL 679	Good	good	-4.054	-2.309	2.679	52.461
NPRL 680	Moderate	very low, but possible	2.785	4.596	6.188	35.160
NPRL 682	Good	good	-2.727	-1.337	1.948	64.347
NPRL 684	Good	low	0.518	3.105	4.247	46.422
NPRL 685	Good	good	-0.885	1.175	3.005	44.091
NPRL 686	Good	low	1.467	2.118	3.882	48.880
NPRL 688	Good	very low, but possible	5.027	2.615	5.413	26.230
NPRL 689	Good	low	-0.537	2.761	4.957	26.230
NPRL 690	Good	low	-0.458	-0.448	4.350	49.166
NPRL 691	Good	low	-1.218	0.861	4.326	52.461
NPRL 692	Good	low	-2.735	-1.720	3.536	37.491
NPRL 693	Good	good	-2.819	-0.880	2.611	52.461
NPRL 694	Good	low	-0.187	1.244	4.455	35.160
NPRL 695	Good	low	-0.612	0.373	4.438	44.091
NPRL 698	Good	low	-5.716	1.612	4.333	86.914
NPRL 700	Good	low	-0.089	1.733	4.448	48.880
NPRL 702	Good	good	-2.202	-0.690	1.633	52.461
NPRL 703	Good	low	1.127	3.180	4.806	37.491
NPRL 704	Good	very low, but possible	-0.101	3.311	5.292	37.491
NPRL 707	Good	opti mal	-5.929	-3.985	-0.162	64.347
NPRL 720	Good	low	-1.987	0.769	3.553	26.230
NPRL 723	Good	good	-4.408	-8.600	2.335	76.995
NPRL 724	Very poor	Extremely low	-5.795	7.052	8.358	82.207
NPRL 725	Very poor	Extremely low	-4.878	4.997	8.541	91.137
NPRL 726	Very poor	Extremely low	-3.413	8.295	8.590	64.347
NPRL 727	Very poor	Extremely low	-5.353	5.151	8.557	82.207

NPRL 728	Very poor	Extremely low	-4.082	5.577	9.338	64.347
NPRL 729	Very poor	Extremely low	-4.254	6.470	9.338	64.347
NPRL 730	Very poor	Extremely low	-4.082	7.021	9.338	64.347
NPRL 731	Very poor	Extremely low	-2.014	9.086	9.254	64.347
NPRL 732	Very poor	Extremely low	-1.952	9.773	9.254	64.347
NPRL 733	Very poor	Extremely low	-2.426	6.608	9.498	64.347
NPRL 734	Very poor	Extremely low	-5.770	6.662	8.573	73.277
NPRL 736	Very poor	Extremely low	-5.472	5.395	8.376	82.207
NPRL 737	Very poor	Extremely low	-5.039	2.267	8.575	91.137
NPRL 738	Very poor	Extremely low	-6.828	2.265	9.032	91.137
NPRL 739	Very poor	Extremely low	-3.639	5.691	8.393	82.207
NPRL 740	Very poor	Extremely low	-3.647	3.461	8.608	73.277
NPRL 741	Very poor	Extremely low	-3.645	3.577	10.192	73.277
NPRL 742	Very poor	Extremely low	-3.964	6.910	9.041	73.277
NPRL 743	Very poor	Extremely low	-6.910	7.835	9.041	73.277
NPRL 744	Very poor	Extremely low	-7.603	8.171	9.025	82.207
NPRL 745	Good	low	0.417	0.218	3.747	60.832
NPRL 746	Very poor	Extremely low	-0.239	3.912	8.138	0.000
NPRL 747	Good	good	-4.960	-0.390	1.770	60.832
NPRL 748	Poor	very low, but possible	-2.590	4.494	6.805	64.347
NPRL 751	Good	low	-2.234	2.199	3.139	95.211
NPRL 752	Good	good	-2.456	0.785	1.642	95.211
NPRL 753	Good	good	0.758	5.355	3.243	29.121
NPRL 754	Very poor	Extremely low	-3.337	3.315	7.121	64.347
NPRL 755	Good	low	-3.095	3.786	2.562	106.042
NPRL 756	Very poor	very low, but possible	-3.394	6.621	7.226	106.042
NPRL 757	Very poor	Extremely low	-2.087	1.813	8.314	72.155
NPRL 758	Good	low	-4.240	2.168	3.718	106.042
NPRL 759	Good	good	-2.730	0.958	2.288	44.091
NPRL 760	Moderate	very low, but possible	-0.220	1.742	6.266	35.720
NPRL 762	Good	good	-5.420	0.983	1.437	53.021
NPRL 763	Good	good	-4.700	-2.260	1.594	79.252
NPRL 764	Good	opti mal	-4.955	-0.547	0.514	61.391
NPRL 765	Poor	very low, but possible	-3.483	-2.400	6.674	79.811
NPRL 766	Good	good	-2.785	2.078	2.933	53.021
NPRL 767	Moderate	very low, but possible	0.451	4.848	6.222	35.720
NPRL 768	Good	low	-0.195	4.208	3.718	35.720
NPRL 769	Good	good	-4.014	-2.058	3.238	100.626
NPRL 770	Good	good	-4.132	2.799	2.185	53.021
NPRL 771	Good	good	-5.138	-1.483	1.446	51.643
NPRL 772	Good	good	-8.822	0.863	2.184	28.561
NPRL 773	Good	opti mal	-4.704	-0.175	0.659	80.205
NPRL 774	Good	good	-2.402	2.385	2.397	60.573
NPRL 775	Good	good	-2.462	0.903	1.725	47.605
NPRL 776	Good	low	-2.052	1.462	4.752	79.252
NPRL 777	Good	opti mal	-3.523	-4.112	0.665	68.421
NPRL 778	Poor	good	-2.226	-1.894	4.654	132.832
NPRL 779	Poor	low	-3.436	1.273	5.710	123.902
NPRL 780	Poor	very low, but possible	-3.414	-1.781	6.487	88.741
NPRL 781	Good	low	-1.873	-0.868	4.582	79.252
NPRL 783	Good	good	-1.635	0.212	2.708	73.836
NPRL 784	Good	good	-2.448	-1.081	3.669	79.252
NPRL 785	Good	good	-4.851	3.299	3.613	100.626
NPRL 786	Very poor	Extremely low	-1.709	5.477	8.120	69.981
NPRL 787	Very poor	Extremely low	0.613	5.357	8.652	43.531
NPRL 788	Good	very low, but possible	-4.521	2.604	4.598	93.533
NPRL 789	Very poor	Extremely low	-1.349	5.371	8.507	66.466
NPRL 791	Very poor	Extremely low	-0.367	2.537	8.741	83.920
NPRL 792	Very poor	Extremely low	-1.659	5.576	7.841	52.461
NPRL 793	Moderate	low	-2.824	2.507	4.647	106.042
NPRL 794	Poor	very low, but possible	-3.037	3.447	6.653	106.042
NPRL 795	Poor	low	-3.860	2.685	5.265	141.762
NPRL 796	Poor	low	-2.038	3.844	5.572	106.042
NPRL 797	Poor	low	-3.278	4.956	5.696	123.902
NPRL 799	Very poor	good	-3.434	2.482	5.631	159.623
NPRL 801	Poor	very low, but possible	-3.121	6.575	6.702	106.042
NPRL 802	Good	low	-2.935	2.558	3.734	106.042
NPRL 803	Good	good	-3.678	0.869	2.401	106.042
NPRL 804	Poor	very low, but possible	-2.964	3.809	5.493	106.042
NPRL 805	Moderate	low	-1.630	1.837	4.600	106.042
NPRL 806	Poor	low	-2.998	3.622	5.150	106.042
NPRL 807	Poor	low	-1.562	1.824	5.493	106.042
NPRL 808	Poor	low	-2.011	3.419	5.559	106.042
NPRL 809	Poor	low	-2.638	2.500	5.536	106.042
NPRL 811	Good	low	-3.349	3.144	2.263	105.483
NPRL 814	Poor	low	-3.919	3.347	5.895	123.902

NPRL 815	Good	low	-4.604	1.827	3.151	65.831
NPRL 816	Good	good	-2.583	1.896	2.471	26.790
NPRL 817	Good	low	-2.620	5.190	4.087	47.043
NPRL 818	Good	low	-4.730	4.013	3.190	64.344
NPRL 819	Good	good	-3.066	1.382	2.459	40.795
NPRL 820	Good	low	-3.060	5.880	3.155	64.282
NPRL 821	Good	low	-2.766	2.946	2.844	64.282
NPRL 822	Good	low	-3.817	5.134	3.904	64.282
NPRL 823	Good	low	-1.198	2.339	5.215	44.650
NPRL 824	Good	low	-0.999	-0.021	2.768	65.831
NPRL 825	Good	low	-2.019	-0.002	4.058	48.530
NPRL 826	Good	low	-4.983	-0.614	3.899	65.831
NPRL 827	Good	low	-3.746	2.228	4.103	64.282
NPRL 828	Good	good	-9.281	-0.651	1.777	60.683
NPRL 829	Good	low	-1.903	-1.201	4.480	44.650
NPRL 830	Good	very low, but possible	-1.329	3.439	5.632	48.530
NPRL 831	Good	very low, but possible	-3.604	2.212	5.483	65.831
NPRL 832	Moderate	very low, but possible	-1.404	2.485	5.686	64.282
NPRL 833	Good	low	5.209	7.541	2.990	26.790
NPRL 834	Good	low	0.913	6.102	2.920	17.860
NPRL 835	Good	low	-0.413	11.174	4.626	30.670
NPRL 836	Good	low	0.149	6.193	4.350	30.670
NPRL 837	Good	very low, but possible	2.117	9.577	4.832	17.860
NPRL 838	Poor	very low, but possible	2.663	10.017	6.675	21.212
NPRL 839	Good	good	-2.657	-0.582	2.143	37.800
NPRL 840	Good	low	1.703	4.263	2.807	37.800
NPRL 841	Good	good	-3.444	2.953	2.126	46.731
NPRL 842	Good	low	-0.054	2.997	3.051	37.800
NPRL 843	Good	very low, but possible	-0.043	3.898	4.826	75.791
NPRL 844	Moderate	very low, but possible	1.194	5.199	5.491	75.791
NPRL 845	Good	very low, but possible	-1.356	3.323	4.735	80.927
NPRL 846	Good	very low, but possible	0.955	5.404	4.914	80.927
NPRL 847	Good	very low, but possible	-0.945	5.810	5.312	75.791
NPRL 848	Very poor	Extremely low	4.184	7.471	7.259	54.877
NPRL 849	Poor	Extremely low	4.731	6.151	6.773	54.877
NPRL 850	Poor	Extremely low	4.074	7.857	6.756	63.807
NPRL 851	Good	very low, but possible	1.361	4.028	5.794	35.160
NPRL 853	Good	good	0.865	2.584	3.306	26.790
NPRL 854	Good	good	1.877	2.527	3.067	35.160
NPRL 855	Good	good	0.665	3.274	1.571	52.461
NPRL 856	Good	very low, but possible	-0.636	1.186	5.533	64.744
NPRL 857	Very poor	very low, but possible	-2.181	-0.762	10.722	103.420
NPRL 859	Very poor	very low, but possible	1.303	2.865	8.274	74.396
NPRL 860	Moderate	low	1.345	2.451	5.393	86.281
NPRL 861	Moderate	very low, but possible	1.466	4.108	5.977	53.580
NPRL 862	Good	good	0.108	0.279	2.682	47.605
NPRL 863	Good	good	0.108	0.279	2.682	47.605
NPRL 864	Good	good	-1.477	-0.803	3.029	0.000
NPRL 865	Good	good	-3.318	-2.227	1.865	17.300
NPRL 866	Good	low	-3.265	3.663	3.582	53.021
NPRL 867	Moderate	too soluble	-3.991	-4.809	-1.259	60.832
NPRL 868	Good	low	-0.089	4.536	2.357	60.832
NPRL 869	Good	opti mal	-6.181	-0.201	1.025	95.993
NPRL 870	Good	too soluble	-2.764	-1.536	-1.023	104.364
NPRL 871	Good	too soluble	-3.050	-2.893	-0.860	78.133
NPRL 872	Good	low	-1.679	3.922	3.453	53.021
NPRL 873	Good	low	1.632	4.570	2.993	47.605
NPRL 874	Good	very low, but possible	3.130	6.250	4.985	35.720
NPRL 875	Good	low	0.156	4.776	3.776	43.531
NPRL 876	Good	low	0.391	3.301	3.665	47.046
NPRL 877	Good	low	-1.708	4.046	2.416	87.063
NPRL880	Good	good	-6.465	-16.390	1.940	87.012
NPRL881	Good	good	-7.817	-15.447	2.127	77.554
NPRL882	Good	low	-2.573	-6.504	4.472	74.202
NPRL883	Good	very low, but possible	-5.785	-7.590	4.752	74.202
NPRL884	Moderate	low	-6.821	-6.707	4.160	117.025
NPRL885	Poor	low	-9.954	-9.867	3.546	132.080
NPRL886	Good	good	-7.127	-16.810	1.591	87.012
NPRL887	Good	low	-5.334	-13.211	3.509	104.313
NPRL891	Good	low	-4.366	-10.708	4.217	100.992
NPRL892	Moderate	low	-8.417	-12.689	4.160	117.025
NPRL894	Good	low	-4.531	-14.032	4.637	74.202
NPRL895	Very poor	low	-7.866	-12.262	4.055	159.848
NPRL898	Good	low	-3.313	-7.376	4.415	74.202
NPRL899	Good	low	-5.187	-10.472	3.661	95.017
NPRL901	Moderate	low	-8.533	-11.074	3.751	122.373

NPRL902	Poor	low	-4.844	-9.487	2.373	139.969
NPRL903	Good	low	-6.964	-11.624	3.240	113.802
NPRL904	Very poor	good	-8.750	-15.283	2.047	162.904
NPRL906	Very poor	low	-11.069	-15.931	2.062	182.792
NPRL908	Poor	low	-4.833	-9.490	2.832	139.969
NPRL910	Good	good	-8.626	-9.876	1.750	124.696
NPRL912	Poor	good	-6.770	-11.843	2.168	139.969
NPRL913	Good	good	-8.339	-12.679	1.450	113.210
NPRL914	Poor	low	-10.660	-18.943	2.739	147.136
NPRL916	Good	low	-6.822	-16.058	2.845	104.313
NPRL920	Moderate	low	-5.472	-4.462	4.490	99.724
NPRL921	Good	low	-2.222	-4.689	4.546	83.691
NPRL922	Moderate	good	-9.929	-10.883	1.141	130.673
NPRL923	Good	good	-10.186	-7.185	1.393	106.902
NPRL924	Good	low	-3.429	-6.210	3.543	56.341
NPRL925	Good	low	-2.725	-5.958	3.543	56.341
NPRL927	Good	good	-7.890	-8.159	0.805	99.873
NPRL929	Good	good	-8.630	-7.256	1.191	82.572
NPRL930	Good	low	-3.691	-0.133	2.576	53.021
NPRL931	Good	low	-0.290	4.672	3.348	53.021
NPRL933	Good	low	-7.824	-17.916	2.828	113.243
NPRL934	Good	low	-4.763	-13.361	3.050	104.313
NPRL935	Moderate	low	-6.392	-18.231	2.812	122.173
NPRL936	Moderate	low	-6.609	-18.333	2.795	131.103
NPRL941	Good	good	-10.484	-10.136	1.402	124.696
NPRL942	Very poor	low	-8.461	-9.878	3.484	152.681
NPRL943	Good	low	-4.069	-8.808	3.013	109.858
NPRL944	Very poor	low	-4.515	-8.706	2.160	168.790
NPRL945	Very poor	low	-7.721	-9.655	3.378	195.504
NPRL946	Moderate	low	-7.563	-5.317	4.326	107.535
NPRL947	Very poor	good	-7.421	-9.467	1.376	189.605
NPRL948	Moderate	good	-9.824	-11.382	1.196	136.460
NPRL950	Good	low	-5.004	-5.201	3.815	97.972
NPRL951	Very poor	low	-9.120	-9.045	3.729	151.626
NPRL952	Good	good	-3.288	-3.697	2.349	88.986
NPRL953	Poor	very low, but possible	-8.165	-3.627	5.668	90.234
NPRL955	Very poor	low	-9.602	-8.436	5.513	168.927
NPRL956	Very poor	very low, but possible	-9.480	-8.408	6.901	168.927
NPRL957	Very poor	low	-8.974	-9.157	3.513	168.927
NPRL958	Good	good	-6.328	-12.490	1.925	109.858
NPRL959	Moderate	low	-5.495	-6.068	4.334	109.858
NPRL960	Very poor	low	-6.865	-3.896	3.484	152.681
NPRL961	Moderate	low	-3.280	-3.484	4.254	109.858
NPRL967	Poor	low	-6.344	-11.749	2.843	136.398
NPRL968	Moderate	low	-2.542	-3.446	3.795	109.858
NPRL969	Poor	low	-5.394	-2.461	4.084	135.380
NPRL970	Moderate	low	-1.555	-1.969	4.283	101.487
NPRL971	Good	low	-0.925	-2.206	3.972	44.091
NPRL972	Good	good	-3.085	-4.238	2.159	105.002
NPRL973	Good	low	-0.379	-3.358	2.857	53.021
NPRL974	Good	low	-1.257	-3.110	3.065	64.906
NPRL977	Very poor	low	-8.290	-6.623	3.484	152.681
NPRL978	Very poor	low	-9.720	-10.408	3.921	157.270
NPRL979	Moderate	low	-5.468	-8.887	3.136	127.159
NPRL981	Good	good	-5.243	0.559	2.115	47.046
NPRL982	Good	good	-5.917	-1.420	1.723	76.791
NPRL983	Good	opti mal	-4.739	-4.117	1.832	41.631
NPRL984	Good	low	-8.678	3.257	3.087	99.165
NPRL985	Poor	very low, but possible	-3.704	2.056	5.777	105.483
NPRL986	Poor	very low, but possible	-5.038	3.785	5.777	105.483
NPRL987	Poor	very low, but possible	-5.306	2.582	5.777	105.483
NPRL988	Very poor	Extremely low	-1.166	2.947	7.695	87.622
NPRL989	Very poor	Extremely low	-0.554	5.934	7.695	87.622
NPRL990	Very poor	Extremely low	-0.230	4.640	7.695	87.622
NPRL991	Good	low	-4.424	3.776	3.343	35.160
NPRL992	Very poor	Extremely low	-3.824	3.266	7.695	87.622
NPRL994	Good	low	-4.783	0.278	2.689	35.160
NPRL995	Good	low	-4.755	0.024	2.661	35.160
NPRL996	Good	good	-7.551	-2.173	1.200	76.791
NPRL997	Poor	very low, but possible	-5.626	-1.274	4.657	140.084
NPRL1000	Good	opti mal	-7.029	-1.840	0.686	76.791
NPRL1001	Poor	very low, but possible	-5.729	-0.859	6.090	140.084
NPRL1002	Poor	very low, but possible	-5.510	-0.581	4.600	140.084
NPRL1003	Poor	very low, but possible	-4.989	2.948	5.568	133.492
NPRL1004	Poor	very low, but possible	-5.161	2.250	5.568	133.492
NPRL1005	Good	opti mal	-7.916	-5.973	1.103	47.131

NPRL1006	Poor	very low, but possible	-4.881	0.266	5.046	140.084
NPRL1007	Poor	very low, but possible	-6.114	-2.114	5.396	140.084
NPRL1008	Very poor	very low, but possible	-4.888	1.370	5.353	154.089
NPRL1009	Good	good	-6.576	-1.171	2.132	35.246
NPRL1010	Very poor	very low, but possible	-7.613	1.663	5.155	163.019
NPRL1011	Very poor	very low, but possible	-7.289	-0.137	4.953	163.019
NPRL1012	Good	good	-6.901	-0.938	1.535	25.539
NPRL1013	Good	opti mal	-7.525	-4.392	1.309	37.424
NPRL1014	Very poor	very low, but possible	-5.864	-2.232	4.349	152.894
NPRL1015	Very poor	low	-5.515	-2.220	4.212	157.385
NPRL1016	Very poor	very low, but possible	-6.981	-3.037	3.917	166.624
NPRL1017	Good	low	-3.916	-0.871	2.338	25.539
NPRL1018	Good	opti mal	-6.281	-2.716	0.868	55.417
NPRL1019	Good	good	-5.797	1.021	2.601	47.046
NPRL1020	Good	low	-4.250	0.683	3.699	35.160
NPRL1021	Good	low	-4.717	2.209	3.175	35.160
NPRL1022	Good	good	-5.386	-0.859	1.368	76.791
NPRL1023	Good	good	-5.755	-1.260	2.209	76.791
NPRL1024	Good	good	-5.990	-3.409	1.686	76.791
NPRL1026	Poor	very low, but possible	-6.560	-1.689	6.123	140.084
NPRL1027	Good	good	-7.723	-2.047	1.172	76.791
NPRL1028	Poor	very low, but possible	-7.622	-2.358	6.013	122.783
NPRL1029	Good	good	-1.103	1.688	2.722	47.046
NPRL1030	Poor	very low, but possible	-6.259	-1.119	5.952	140.084
NPRL1031	Poor	very low, but possible	-4.762	3.801	6.176	140.084
NPRL1032	Very poor	very low, but possible	-6.913	0.016	5.476	163.019
NPRL1033	Poor	very low, but possible	-6.268	-1.119	5.574	140.084
NPRL1034	Very poor	very low, but possible	-7.835	-1.527	4.744	163.019
NPRL1035	Poor	very low, but possible	-5.616	-1.752	6.580	140.084
NPRL1036	Poor	very low, but possible	-6.530	-6.316	3.137	140.084
NPRL1037	Very poor	very low, but possible	-6.409	-3.994	4.800	164.155
NPRL1038	Very poor	low	-3.802	-3.832	4.591	152.367
NPRL1039	Poor	good	-4.319	-4.166	3.501	149.993
NPRL1040	Poor	very low, but possible	-4.791	1.261	6.792	87.622
NPRL1041	Poor	low	-3.581	1.195	5.847	105.483
NPRL1042	Poor	very low, but possible	-3.416	0.955	5.880	87.622
NPRL1043	Good	very low, but possible	-0.208	3.691	5.707	53.021
NPRL1044	Poor	very low, but possible	-6.008	3.885	6.713	105.483
NPRL1045	Very poor	very low, but possible	-7.731	1.197	5.599	173.268
NPRL1046	Poor	very low, but possible	-4.869	5.185	6.782	87.622
NPRL1047	Moderate	very low, but possible	-2.010	2.866	6.350	53.021
NPRL1052	Good	good	-4.443	-4.476	2.144	67.861
NPRL1055	Good	good	-4.945	1.127	1.676	73.277
NPRL1056	Poor	very low, but possible	-4.463	4.293	6.746	87.622
NPRL1058	Moderate	low	-6.146	-0.131	4.719	94.327
NPRL1059	Good	low	-6.216	-0.331	4.280	94.327
NPRL1060	Good	low	-3.873	6.175	3.292	52.461
NPRL1061	Poor	very low, but possible	-5.446	-5.337	6.582	76.878
NPRL1062	Poor	very low, but possible	-4.594	1.062	6.785	78.692
NPRL1067	Good	very low, but possible	-2.653	6.177	4.221	44.091
NPRL1068	Good	low	-5.201	5.338	3.276	61.391
NPRL1069	Good	low	-8.418	3.744	2.700	95.284
NPRL1071	Good	good	-6.001	3.330	1.317	94.092
NPRL1072	Good	low	-2.940	4.717	4.205	53.021
NPRL1073	Good	low	-0.133	1.862	3.924	64.347
NPRL1076	Good	low	-5.228	3.940	3.614	67.026
NPRL1083	Good	opti mal	-7.203	-4.056	0.617	100.562
NPRL1084	Good	good	-6.530	-2.079	0.873	97.048
NPRL1085	Poor	very low, but possible	-6.798	-1.579	6.052	140.084
NPRL1086	Good	low	-4.083	-1.490	2.146	44.091
NPRL1087	Good	good	-1.860	-2.029	2.627	73.707
NPRL1088	Very poor	very low, but possible	-6.375	-4.993	4.219	166.624
NPRL1089	Good	good	-1.426	5.020	2.901	50.398
NPRL1090	Good	low	0.737	5.229	3.127	38.513
NPRL1092	Good	good	-1.692	2.083	2.676	62.284
NPRL1093	Good	good	-3.724	3.287	2.246	85.722
NPRL1094	Poor	very low, but possible	-4.059	3.418	6.160	149.014
NPRL1096	Good	low	-1.718	8.083	3.931	38.513
NPRL1098	Good	low	-2.272	1.860	2.953	58.307
NPRL1099	Good	good	-2.969	3.542	2.216	92.029
NPRL1101	Good	good	-1.944	1.421	2.966	67.861
NPRL1103	Good	good	-1.689	1.108	2.966	67.861
NPRL1104	Good	low	-4.242	7.064	3.292	52.461
NPRL1105	Good	low	-3.987	6.750	3.292	52.461
NPRL1106	Good	good	-2.544	4.188	2.442	80.144
NPRL1107	Good	very low, but possible	-2.909	6.490	4.221	44.091

NPRL1109	Poor	very low, but possible	-3.909	4.599	6.355	143.437
NPRL1110	Good	low	-4.024	3.844	2.732	85.722
NPRL1111	Good	low	-3.768	3.531	2.732	85.722
NPRL1112	Poor	very low, but possible	-5.279	3.802	6.646	149.014
NPRL1113	Poor	very low, but possible	-5.023	3.488	6.646	149.014
NPRL1114	Good	good	-5.938	-3.025	1.183	85.722
NPRL1115	Good	very low, but possible	-2.734	7.845	4.417	38.513
NPRL1116	Good	low	-4.449	4.130	2.937	67.237
NPRL1118	Good	good	-2.412	-1.327	2.464	76.791
NPRL1119	Good	good	-0.603	1.954	2.685	67.861
NPRL1120	Good	low	-4.204	3.479	2.790	61.391
NPRL1121	Good	low	-3.688	3.041	3.493	64.906
NPRL1122	Good	low	-4.860	5.005	2.790	61.391
NPRL1124	Good	low	-2.028	7.217	3.012	52.461
NPRL1125	Good	low	-1.619	6.661	3.715	55.976
NPRL1127	Good	low	-2.755	2.703	3.719	53.021
NPRL1128	Good	low	-3.905	4.683	3.719	53.021
NPRL1129	Good	good	-2.401	0.341	2.229	94.652
NPRL1130	Good	good	-3.551	2.321	2.229	94.652
NPRL1131	Good	low	-2.429	4.628	2.451	85.722
NPRL1132	Very poor	very low, but possible	-5.360	2.729	6.143	157.944
NPRL1133	Good	good	-2.626	0.529	2.464	76.791
NPRL1134	Good	low	-3.600	4.555	3.493	64.906
NPRL1135	Good	low	-4.116	4.993	2.790	61.391
NPRL1136	Good	low	-2.667	4.218	3.719	53.021
NPRL1137	Good	good	-2.313	1.855	2.229	94.652
NPRL1142	Good	good	-5.695	-1.628	2.260	112.188
NPRL1143	Moderate	low	-2.777	-0.910	3.140	120.841
NPRL1144	Good	good	-0.083	2.340	2.685	67.861
NPRL1145	Very poor	very low, but possible	-5.598	-1.123	8.200	94.327
NPRL1146	Good	very low, but possible	-2.539	5.601	4.221	44.091
NPRL1147	Good	low	-3.027	4.802	3.719	53.021
NPRL1149	Good	low	-2.086	6.310	3.012	52.461
NPRL1150	Good	low	-1.877	6.118	3.012	52.461
NPRL1151	Good	low	-1.083	5.743	3.940	44.091
NPRL1152	Good	low	-0.875	5.552	3.940	44.091
NPRL1153	Good	low	-2.336	2.243	2.865	85.722
NPRL1157	Good	low	-7.902	3.189	3.404	98.799
NPRL1159	Good	low	-3.170	4.894	3.995	55.976
NPRL1160	Good	low	-3.815	5.729	3.292	52.461
NPRL1161	Good	low	-6.582	3.272	3.629	86.914
NPRL1162	Good	very low, but possible	-2.482	5.155	4.221	44.091
NPRL1163	Good	low	-3.985	1.526	3.146	85.722
NPRL1165	Good	low	-3.407	2.883	3.493	64.906
NPRL1168	Good	good	-6.092	-1.498	2.374	110.684
NPRL1169	Good	low	-4.275	4.858	3.594	61.391
NPRL1171	Good	very low, but possible	-4.906	4.696	4.486	61.391
NPRL1173	Good	good	-6.150	-0.497	2.374	110.684
NPRL1175	Good	good	-5.241	2.017	1.731	94.092
NPRL1176	Good	low	-4.230	5.374	3.555	52.461
NPRL1177	Good	very low, but possible	-3.338	4.800	4.483	44.091
NPRL1178	Good	low	-4.173	4.556	3.555	52.461
NPRL1179	Good	low	-4.995	1.818	3.408	85.722
NPRL1180	Good	good	-6.284	1.686	2.699	53.046
NPRL1181	Good	low	0.496	4.445	3.510	43.337
NPRL1182	Good	good	-5.542	4.780	2.699	53.046
NPRL1183	Good	good	-5.930	3.336	2.699	53.046
NPRL1184	Good	low	-7.025	1.921	3.402	53.046
NPRL1185	Good	low	0.173	5.659	3.510	43.337
NPRL1186	Good	low	0.424	5.767	3.510	43.337
NPRL1187	Good	low	-0.291	2.774	4.213	43.337
NPRL1188	Good	low	-0.808	10.137	3.478	61.197
NPRL1189	Good	low	-0.486	8.923	3.478	61.197
NPRL1190	Good	low	-0.728	10.005	3.478	61.197
NPRL1191	Good	low	-1.227	6.911	4.180	61.197
NPRL1192	Good	very low, but possible	-1.309	9.815	4.296	29.121
NPRL1193	Good	low	-0.234	4.918	2.925	30.111
NPRL1194	Good	good	-3.718	2.731	2.955	43.840
NPRL1195	Good	low	1.207	6.619	2.908	39.041
NPRL1196	Good	low	-0.421	4.777	2.908	39.041
NPRL1197	Good	low	-4.220	5.693	3.677	85.097
NPRL1198	Moderate	low	0.321	10.092	4.957	87.708
NPRL1199	Good	low	-0.475	7.543	3.531	42.393
NPRL1200	Good	very low, but possible	-0.412	8.052	5.432	14.613
NPRL1201	Good	low	-1.481	8.305	4.253	32.473
NPRL1202	Good	low	-1.359	4.616	4.809	11.260

NPRL1203	Good	very low, but possible	-0.911	4.118	4.792	20.191
NPRL1204	Good	very low, but possible	-1.422	8.650	4.776	29.121
NPRL1205	Good	low	-1.152	9.737	4.079	40.382
NPRL1207	Good	low	-1.073	9.605	4.079	40.382
NPRL1208	Good	very low, but possible	-1.417	6.087	4.782	40.382
NPRL1209	Good	low	-0.004	8.062	2.898	47.971
NPRL1210	Good	low	2.768	6.433	3.816	58.307
NPRL1211	Good	good	-3.197	-0.611	1.655	52.461
NPRL1212	Good	opti mal	-6.688	-1.972	0.591	61.701
NPRL1213	Good	low	-5.588	4.326	2.707	70.631
NPRL1214	Good	good	-2.787	4.602	2.759	50.926
NPRL1215	Good	good	-3.143	2.665	1.877	79.561
NPRL1216	Good	low	-2.036	6.249	2.929	61.701
NPRL1217	Good	low	-1.243	4.798	2.099	70.631
NPRL1218	Good	good	-2.673	4.928	2.246	68.786
NPRL1222	Good	good	-5.771	-0.992	1.153	61.391
NPRL1223	Good	low	-1.691	4.835	2.676	56.901
NPRL1224	Good	good	-9.541	1.263	2.366	56.901
NPRL1225	Good	good	-2.993	2.201	1.999	79.252
NPRL1226	Good	good	-5.975	3.917	0.808	91.502
NPRL1228	Good	low	-1.474	4.637	3.653	93.285
NPRL1229	Good	low	-1.474	4.637	3.653	93.285
NPRL1232	Good	low	-2.241	3.557	2.698	52.410
NPRL1235	Good	low	2.147	10.600	4.212	33.463
NPRL1237	Good	low	-1.253	6.433	3.643	102.215
NPRL1238	Good	good	-4.657	2.494	2.128	60.123
NPRL1239	Good	good	-5.499	3.164	2.087	63.476
NPRL1240	Good	good	-3.954	0.285	1.446	47.193
NPRL1241	Good	low	-0.438	7.155	2.898	47.971
NPRL1242	Good	good	-5.160	4.381	2.939	52.770
NPRL1243	Good	low	0.484	5.119	2.908	39.041
NPRL1244	Good	good	0.385	2.436	1.911	66.678
NPRL1245	Good	low	-3.480	8.751	4.027	64.744
NPRL1247	Good	low	0.868	5.107	3.611	58.307
NPRL1250	Good	low	0.879	4.631	4.433	61.822
NPRL1251	Good	good	1.511	5.970	2.117	66.678
NPRL1252	Good	good	1.132	6.702	2.117	66.678
NPRL1253	Good	very low, but possible	-2.658	4.581	4.186	102.215
NPRL1254	Good	good	-5.028	-0.657	1.153	61.391
NPRL1255	Good	low	-1.723	5.374	3.441	47.971
NPRL1259	Good	low	-3.160	5.348	4.430	25.092
NPRL1260	Good	good	-6.601	-1.448	0.803	69.762
NPRL1261	Good	good	-3.726	-0.968	2.101	94.092
NPRL1262	Good	low	-2.386	4.694	2.929	61.701
NPRL1263	Good	good	-1.372	4.380	2.612	52.770
NPRL1264	Good	good	-2.473	7.705	1.202	74.202
NPRL1265	Very poor	too soluble	-0.554	-6.639	-2.909	69.203
NPRL1266	Good	good	-1.748	1.745	1.449	110.834
NPRL1267	Moderate	opti mal	-7.074	-5.467	1.498	3.352
NPRL1268	Good	opti mal	-4.579	2.980	1.118	34.601
NPRL1270	Good	low	-0.264	3.225	4.207	32.076
NPRL1271	Good	low	0.290	2.383	4.190	41.006
NPRL1272	Good	low	-1.437	7.399	4.174	49.936
NPRL1273	Good	low	-4.340	5.445	4.071	41.006
NPRL1275	Good	low	-4.613	1.359	3.100	41.006
NPRL1277	Good	low	-4.948	-4.109	3.053	107.577
NPRL1278	Good	very low, but possible	-4.675	16.149	4.015	86.762
NPRL1280	Good	good	-5.638	-2.329	2.557	52.891
NPRL1281	Good	very low, but possible	-2.301	2.299	5.098	39.041
NPRL1282	Good	low	-1.352	1.679	3.520	39.041
NPRL1284	Good	low	-2.803	1.325	3.891	39.041
NPRL1285	Good	low	-2.222	1.689	3.405	39.041
NPRL1286	Good	very low, but possible	-1.066	2.256	5.524	39.041
NPRL1287	Good	low	-1.417	0.842	2.204	57.123
NPRL1288	Good	good	-1.223	-0.982	2.793	46.752
NPRL1289	Good	good	-3.014	-0.436	2.307	46.752
NPRL1290	Good	low	-0.386	-0.109	3.558	52.547
NPRL1292	Good	low	-2.004	-0.822	3.190	69.568
NPRL1294	Good	good	-3.303	-1.599	1.627	77.939
NPRL1295	Moderate	low	-8.228	10.872	3.369	124.836
NPRL1296	Poor	very low, but possible	-0.587	1.497	6.478	64.152
NPRL1297	Good	good	-2.240	-0.447	2.648	32.076
NPRL1299	Good	good	-2.062	-0.256	2.873	46.884
NPRL1300	Good	low	-2.922	-1.714	3.214	38.513
NPRL1301	Good	low	-1.355	2.584	4.662	26.230
NPRL1302	Good	low	-0.678	1.583	4.116	26.230

NPRL1303	Good	good	-3.095	-0.744	2.195	26.230
NPRL1305	Good	low	-0.352	3.556	3.571	34.601
NPRL1306	Good	low	-1.663	-2.562	2.674	43.531
NPRL1307	Good	good	-4.366	-6.742	1.955	76.232
NPRL1309	Good	good	-4.060	-0.429	2.337	76.232
NPRL1310	Good	low	-2.706	-0.820	2.784	81.648
NPRL1311	Good	good	-6.093	-3.035	1.864	102.463
NPRL1312	Good	low	-1.907	2.247	3.519	60.832
NPRL1313	Good	low	-1.608	0.901	3.257	55.417
NPRL1314	Good	low	-1.737	-3.027	2.874	55.417
NPRL1315	Good	low	-3.390	4.896	3.034	81.648
NPRL1316	Good	opti mal	-3.629	-0.458	0.022	90.018
NPRL1317	Good	low	-3.603	-2.345	2.251	47.411
NPRL1320	Good	low	-3.974	1.312	3.587	41.006
NPRL1322	Good	good	-7.518	-2.664	2.071	52.891
NPRL1323	Good	low	-4.513	2.111	2.477	39.041
NPRL1325	Good	low	-3.609	1.414	4.188	20.191
NPRL1326	Good	good	-6.438	4.821	3.066	39.041
NPRL1327	Good	low	-2.362	7.236	2.690	55.417
NPRL1328	Good	low	-1.148	2.981	4.092	43.464
NPRL1329	Good	good	-3.712	6.848	2.050	55.417
NPRL1330	Good	low	-4.218	6.853	3.414	87.063
NPRL1331	Good	good	-5.612	5.464	1.715	95.434
NPRL1334	Good	low	-1.379	6.572	3.750	47.046
NPRL1335	Good	good	-3.103	-1.642	2.037	81.648
NPRL1336	Good	low	-1.584	4.199	2.361	51.902
NPRL1337	Good	low	-2.052	4.831	3.250	64.347
NPRL1339	Good	low	-1.223	13.372	3.985	43.531
NPRL1340	Good	low	-3.462	14.031	2.285	51.902
NPRL1341	Good	low	-5.139	18.470	4.269	47.411
NPRL1342	Good	low	-5.474	4.231	2.947	60.222
NPRL1343	Good	low	-3.812	-0.808	2.991	60.222
NPRL1344	Good	low	-4.238	5.363	3.197	60.222
NPRL1346	Moderate	very low, but possible	-9.803	16.128	5.295	90.234
NPRL1347	Moderate	very low, but possible	-7.582	5.215	4.429	103.045
NPRL1348	Moderate	very low, but possible	-6.627	5.013	4.679	103.045
NPRL1349	Moderate	very low, but possible	-7.135	17.757	4.656	103.045
NPRL1350	Good	good	-4.918	4.649	-0.097	86.503
NPRL1351	Good	good	-5.253	-0.208	1.213	64.712
NPRL1352	Good	good	-6.208	0.152	0.963	64.712
NPRL1353	Good	low	-5.474	4.231	2.947	60.222
NPRL1354	Good	low	-4.522	17.526	3.750	64.712
NPRL1355	Good	good	-3.531	-1.731	1.540	44.091
NPRL1356	Good	good	-4.612	-0.844	1.573	26.230
NPRL1357	Good	good	-4.068	-0.313	1.556	35.160
NPRL1358	Good	good	-2.253	-0.167	1.565	35.720
NPRL1359	Good	opti mal	-3.786	-3.136	1.176	47.605
NPRL1360	Very poor	Extremely low	0.290	1.644	7.267	26.790
NPRL1361	Good	opti mal	-4.118	-1.657	1.209	29.745
NPRL1362	Very poor	Extremely low	-0.250	2.690	7.300	8.930
NPRL1363	Good	low	-0.551	2.199	4.178	44.650
NPRL1364	Good	low	-3.022	5.794	3.518	53.580
NPRL1365	Good	low	-3.333	8.340	3.551	35.720
NPRL1366	Good	opti mal	-4.501	-3.106	1.331	47.046
NPRL1367	Good	good	1.513	1.397	3.140	35.160
NPRL1368	Good	opti mal	-5.334	-3.414	1.331	47.046
NPRL1369	Good	good	-1.555	1.288	3.037	26.230
NPRL1370	Good	opti mal	-2.631	-1.260	0.986	38.675
NPRL1371	Very poor	very low, but possible	-0.033	3.007	7.078	17.860
NPRL1372	Good	low	0.055	5.357	5.144	26.790
NPRL1373	Good	low	1.394	3.887	4.842	47.605
NPRL1374	Poor	very low, but possible	-4.367	6.150	6.778	70.321
NPRL1375	Good	opti mal	-1.217	0.868	1.430	20.815
NPRL1377	Very poor	Extremely low	3.109	5.624	7.522	0.000
NPRL1378	Good	very low, but possible	-2.673	4.753	5.466	60.683
NPRL1379	Good	low	2.464	6.725	4.207	29.745
NPRL1381	Moderate	very low, but possible	1.090	6.995	6.204	26.790
NPRL1382	Very poor	Extremely low	3.061	8.446	9.933	35.720
NPRL1383	Good	low	1.212	4.354	3.547	26.230
NPRL1384	Good	good	-1.784	1.715	1.794	17.300
NPRL1386	Good	good	0.522	2.219	2.780	0.000
NPRL1388	Good	low	0.333	9.156	4.497	8.930
NPRL1391	Good	opti mal	-4.048	-1.590	1.408	44.091
NPRL1392	Good	opti mal	-3.506	-1.058	1.044	47.605
NPRL1393	Good	good	-0.626	2.079	2.393	26.790
NPRL1394	Very poor	very low, but possible	0.163	2.831	7.058	29.745

NPRL1396	Good	good	-3.773	-0.362	2.059	26.230
NPRL1397	Good	low	-1.583	4.547	3.932	17.860
NPRL1398	Good	good	-4.195	2.237	2.851	8.930
NPRL1399	Good	low	-2.575	5.834	5.185	44.650
NPRL1400	Good	good	-0.355	2.549	3.820	56.535
NPRL1401	Good	good	-4.516	-0.759	2.842	43.840
NPRL1402	Good	low	2.277	5.857	3.723	43.531
NPRL1404	Good	good	-5.704	-3.032	1.689	60.123
NPRL1405	Good	good	-3.142	-0.003	2.674	42.823
NPRL1406	Very poor	Extremely low	-2.101	4.153	7.195	51.753
NPRL1407	Good	low	-5.880	5.971	3.899	51.753
NPRL1408	Very poor	very low, but possible	-0.213	3.426	8.202	35.720
NPRL1409	Good	opti mal	-5.008	-2.812	1.325	63.638
NPRL1410	Moderate	very low, but possible	-0.405	5.588	5.911	60.683
NPRL1411	Good	low	-1.733	3.682	4.102	72.568
NPRL1412	Poor	very low, but possible	-5.888	6.484	6.039	95.284
NPRL1415	Good	good	1.375	2.206	2.776	38.675
NPRL1416	Very poor	Extremely low	0.863	2.711	8.867	17.860
NPRL1417	Moderate	very low, but possible	-3.680	4.693	5.688	69.613
NPRL1419	Good	good	-4.005	1.807	1.910	35.160
NPRL1420	Good	opti mal	-3.381	-1.101	1.044	47.605
NPRL1421	Good	low	-2.967	5.141	5.185	44.650
NPRL1422	Good	good	-0.147	4.475	3.821	56.535
NPRL1423	Moderate	very low, but possible	-4.002	6.555	5.757	79.252
NPRL1424	Very poor	very low, but possible	-3.244	5.309	8.095	87.473
NPRL1425	Very poor	very low, but possible	-3.757	2.856	8.018	90.428
NPRL1426	Very poor	Extremely low	-4.821	4.768	7.785	78.543
NPRL1427	Very poor	Extremely low	-0.134	4.289	7.144	62.260
NPRL1428	Very poor	Extremely low	0.422	5.459	7.891	35.720
NPRL1429	Moderate	very low, but possible	-0.859	2.399	5.807	64.347
NPRL1430	Very poor	very low, but possible	-2.471	1.757	7.243	87.622
NPRL1431	Poor	good	-2.043	1.419	6.711	141.244
NPRL1432	Very poor	good	-2.178	0.043	6.836	152.505
NPRL1433	Very poor	good	-2.699	1.419	6.188	152.505
NPRL1434	Very poor	low	-2.199	3.144	7.037	106.643
NPRL1435	Moderate	very low, but possible	-1.074	6.067	6.039	44.650
NPRL1436	Moderate	very low, but possible	-5.192	4.175	6.098	69.613
NPRL1437	Good	low	-0.430	5.037	4.822	42.299
NPRL1438	Good	low	-2.073	3.535	2.562	35.160
NPRL1439	Good	low	2.184	2.997	5.553	47.605
NPRL1440	Good	low	0.455	5.601	4.063	35.720
NPRL1443	Very poor	very low, but possible	2.351	5.567	8.124	47.605
NPRL1444	Very poor	Extremely low	1.711	9.279	8.349	35.720
NPRL1445	Good	opti mal	-5.532	-2.391	0.698	64.347
NPRL1446	Good	opti mal	-7.657	-4.704	0.989	76.791
NPRL1447	Good	low	-1.590	1.355	2.564	52.461
NPRL1448	Good	low	2.182	1.997	3.664	8.930
NPRL1449	Good	low	-0.438	2.644	3.020	52.461
NPRL1450	Good	low	-5.463	4.882	3.585	38.785
NPRL1451	Good	good	0.550	2.101	3.438	20.815
NPRL1452	Good	low	-5.281	6.325	4.042	38.785
NPRL1453	Good	good	-2.659	0.776	2.053	43.531
NPRL1454	Good	very low, but possible	-1.980	3.740	4.226	38.785
NPRL1455	Good	low	-1.449	2.188	2.509	43.531
NPRL1456	Very poor	too soluble	-4.096	-9.507	-2.541	61.837
NPRL1457	Very poor	too soluble	-4.131	-8.960	-2.293	61.837
NPRL1458	Good	opti mal	-5.091	-3.429	0.819	26.230
NPRL1460	Good	good	-1.620	0.570	2.740	26.540
NPRL1462	Good	good	-5.759	-3.424	1.300	26.230
NPRL1463	Poor	opti mal	-5.399	-9.439	-0.420	8.930
NPRL1464	Good	opti mal	-4.489	-3.888	1.699	24.167
NPRL1465	Very poor	too soluble	-2.482	-11.416	-2.747	71.295
NPRL1466	Good	low	2.789	5.477	3.728	34.601
NPRL1468	Good	opti mal	-6.868	-2.633	0.767	32.138
NPRL1469	Good	low	-1.363	4.510	2.994	67.971
NPRL1470	Good	good	-0.012	0.082	3.359	50.561
NPRL1471	Good	opti mal	-5.388	-5.492	0.061	46.884
NPRL1472	Good	low	-3.099	-2.606	3.125	53.840
NPRL1473	Good	good	-1.161	-0.118	3.196	41.631
NPRL1474	Good	good	-0.405	2.502	2.934	26.540
NPRL1475	Good	low	0.500	3.875	3.218	49.377
NPRL1476	Good	low	-5.410	1.661	3.381	42.823
NPRL1477	Good	opti mal	-6.073	-2.902	0.464	47.411
NPRL1478	Good	good	-6.015	-2.095	1.067	50.398
NPRL1480	Good	good	-5.653	0.134	2.632	42.823
NPRL1481	Good	low	0.182	3.787	3.973	0.000

NPRL1482	Poor	good	-6.576	-3.205	1.232	144.094
NPRL1483	Good	good	-2.200	-0.420	1.905	52.461
NPRL1484	Very poor	good	-8.253	-21.396	-0.348	231.990
NPRL1485	Good	good	-2.875	-4.893	2.635	92.192
NPRL1486	Good	low	0.764	-2.026	2.666	56.535
NPRL1487	Very poor	low	-8.253	-20.793	-0.348	231.990
NPRL1488	Very poor	low	-11.508	-22.707	0.031	237.405
NPRL1489	Very poor	good	-12.291	-22.951	0.410	242.820
NPRL1490	Moderate	good	-4.424	-13.612	0.736	136.842
NPRL1491	Good	good	-2.602	-1.319	1.663	73.277
NPRL1492	Good	good	-0.015	-0.322	3.179	68.421
NPRL1493	Poor	good	-4.994	-12.367	1.249	148.727
NPRL1494	Moderate	good	-6.426	-14.338	0.678	136.283
NPRL1495	Good	low	-2.307	-0.529	3.298	69.203
NPRL1496	Good	good	-5.303	-3.237	0.872	73.836
NPRL1497	Good	low	-5.615	-4.924	1.259	70.321
NPRL1498	Very poor	opti mal	-12.625	-2.268	1.506	250.853
NPRL1499	Moderate	very low, but possible	-2.832	1.342	5.928	47.046
NPRL1500	Good	low	-1.621	-1.342	3.776	47.046
NPRL1502	Very poor	opti mal	-14.471	-6.823	0.609	263.408
NPRL1503	Moderate	too soluble	-3.834	-5.743	-1.444	93.533
NPRL1505	Good	low	-1.649	-10.037	3.503	109.513
NPRL1506	Good	low	-5.012	-1.413	4.086	75.608
NPRL1507	Good	low	0.924	2.732	4.918	34.601
NPRL1508	Good	good	-4.388	3.130	2.676	73.277
NPRL1509	Good	good	-5.061	3.583	2.552	73.277
NPRL1510	Good	low	-2.904	3.814	3.913	52.461
NPRL1511	Good	opti mal	-6.383	-2.859	0.237	81.648
NPRL1512	Good	good	-5.353	-0.050	0.968	55.417
NPRL1513	Good	very low, but possible	-1.456	-1.126	4.866	62.122
NPRL1514	Good	low	-1.194	-2.513	3.345	62.122
NPRL1515	Good	very low, but possible	-0.557	1.541	5.338	53.192
NPRL1516	Good	low	-0.915	0.367	3.818	53.192
NPRL1517	Very poor	opti mal	-3.669	-5.919	-1.814	164.910
NPRL1518	Very poor	good	-6.594	-5.310	0.213	211.956
NPRL1519	Good	good	-5.399	-1.887	2.190	55.633
NPRL1520	Good	very low, but possible	-4.140	3.909	4.073	69.139
NPRL1521	Good	opti mal	-7.194	2.379	-0.317	95.284
NPRL1522	Good	opti mal	-2.372	-2.500	0.020	34.601
NPRL1523	Good	good	-4.376	2.668	2.105	35.160
NPRL1524	Good	good	-1.206	1.136	2.569	52.461
NPRL1525	Good	opti mal	-3.509	2.562	0.537	52.461
NPRL1526	Good	opti mal	-6.658	-3.493	-0.541	78.133
NPRL1528	Good	low	-0.805	1.233	3.488	26.230
NPRL1530	Good	good	-3.497	-0.854	1.158	60.832
NPRL1531	Good	good	-5.914	-1.262	1.303	43.531
NPRL1532	Good	low	-0.028	4.674	3.556	48.530
NPRL1533	Good	low	-5.300	0.104	2.600	48.530
NPRL1535	Good	low	-4.678	1.428	2.369	65.831
NPRL1536	Good	good	-0.109	-0.455	3.216	47.046
NPRL1537	Good	good	0.024	1.053	2.819	52.461
NPRL1538	Good	good	-2.879	-0.492	1.122	56.341
NPRL1539	Good	low	-3.390	2.223	2.735	48.530
NPRL1540	Good	good	0.147	-0.418	3.479	38.116
NPRL1541	Good	very low, but possible	0.369	7.320	4.057	35.720
NPRL1542	Good	opti mal	-5.398	-2.460	1.182	55.976
NPRL1543	Good	good	-3.670	0.075	3.218	58.931
NPRL1544	Good	good	-7.533	1.407	1.916	35.160
NPRL1545	Moderate	very low, but possible	-2.541	6.324	5.735	17.860
NPRL1546	Good	good	-6.193	2.704	2.419	26.230
NPRL1547	Good	low	-2.659	6.593	4.676	44.091
NPRL1550	Good	good	-0.375	2.067	3.284	0.000
NPRL1551	Good	good	-3.295	4.106	3.092	43.531
NPRL1552	Good	good	0.159	1.324	2.236	17.300
NPRL1553	Good	good	-1.785	1.007	2.018	35.160
NPRL1555	Moderate	opti mal	-2.762	-8.352	-0.244	29.434
NPRL1556	Good	good	-4.848	2.048	1.698	53.021
NPRL1557	Good	good	-5.169	1.969	1.702	43.531
NPRL1558	Moderate	low	-1.935	3.114	5.105	88.182
NPRL1559	Good	good	-2.774	0.788	1.448	52.461
NPRL1560	Moderate	low	-3.360	3.657	5.443	76.232
NPRL1561	Good	opti mal	-3.463	-9.460	1.032	58.931
NPRL1562	Good	good	-3.332	3.287	4.194	76.232
NPRL1563	Good	low	-2.461	1.533	3.825	55.417
NPRL1564	Good	good	-4.971	1.304	2.170	43.531
NPRL1565	Good	good	-5.463	2.280	2.479	26.230

NPRL1566	Good	low	-1.361	5.817	4.926	35.720
NPRL1567	Good	good	-3.487	-0.491	1.582	34.601
NPRL1568	Good	good	-4.923	1.501	2.348	17.860
NPRL1569	Good	good	-0.986	2.932	3.394	17.860
NPRL1570	Good	good	-4.696	-1.683	1.662	34.601
NPRL1571	Poor	very low, but possible	-2.579	2.259	6.546	58.931
NPRL1572	Good	opti mal	-4.372	-3.079	1.182	55.976
NPRL1573	Good	good	-2.930	2.825	3.056	47.971
NPRL1574	Good	good	-4.462	2.956	2.390	43.531
NPRL1577	Good	low	-5.011	3.789	4.493	26.230
NPRL1578	Good	good	-3.256	-2.603	1.871	52.461
NPRL1579	Good	good	-5.987	3.668	3.452	105.483
NPRL1580	Good	low	-0.721	4.766	4.571	17.300
NPRL1582	Good	low	-4.781	6.087	4.773	29.583
NPRL1583	Good	good	-4.433	0.427	3.346	94.652
NPRL1586	Good	good	-3.912	2.128	2.694	38.116
NPRL1589	Good	good	-4.766	0.821	2.028	17.300
NPRL1590	Good	good	-2.714	6.334	3.513	43.531
NPRL1593	Good	opti mal	-5.194	-4.432	0.197	35.160
NPRL1594	Good	good	-6.722	2.280	2.401	26.230
NPRL1595	Good	good	-6.931	0.224	1.933	26.230
NPRL1596	Moderate	low	-3.588	2.909	5.320	97.048
NPRL1597	Good	good	-5.330	-0.888	2.175	38.116
NPRL1598	Good	good	-3.710	-0.161	2.920	38.116
NPRL1599	Good	opti mal	-5.572	-3.818	-0.100	43.531
NPRL1601	Good	opti mal	-4.754	-2.811	-0.038	43.531
NPRL1602	Good	good	-5.177	3.246	2.469	44.091
NPRL1603	Good	low	-5.746	2.213	5.198	73.277
NPRL1604	Very poor	very low, but possible	0.715	0.409	7.124	26.790
NPRL1606	Good	good	-4.817	3.945	2.288	69.762
NPRL1607	Good	good	-6.594	3.000	3.020	55.814
NPRL1609	Good	low	-1.246	2.661	4.452	17.300
NPRL1614	Good	good	-5.736	1.343	1.685	52.461
NPRL1616	Good	good	-3.908	0.982	2.428	122.224
NPRL1618	Good	low	-3.317	5.234	5.063	35.160
NPRL1620	Good	low	-2.460	3.840	4.160	17.300
NPRL1623	Good	low	-3.722	6.135	5.063	35.160
NPRL1625	Good	opti mal	-5.281	-5.965	1.105	58.931
NPRL1627	Moderate	very low, but possible	-3.707	6.448	6.519	26.230
NPRL1628	Good	good	-1.951	2.942	3.753	55.976
NPRL1629	Good	good	-4.981	4.112	2.463	35.160
NPRL1630	Good	good	-5.633	1.455	3.255	87.622
NPRL1633	Good	opti mal	-6.694	-5.244	0.006	75.908
NPRL1636	Good	good	-2.489	2.985	3.918	38.116
NPRL1637	Moderate	too soluble	-2.295	-5.491	-1.287	69.203
NPRL1639	Good	good	0.151	4.976	3.581	47.046
NPRL1640	Good	low	-4.610	7.659	4.773	29.583
NPRL1641	Moderate	low	-3.034	5.615	5.698	17.860
NPRL1642	Good	low	-2.868	5.123	4.800	35.160
NPRL1643	Good	opti mal	-2.252	-2.535	1.661	17.300
NPRL1644	Good	good	-3.497	-0.854	1.158	60.832
NPRL1646	Good	good	-0.892	-0.506	1.542	43.531
NPRL1649	Good	good	-3.361	0.765	2.048	35.160
NPRL1651	Very poor	Extremely low	-1.274	3.099	7.417	42.823
NPRL1653	Moderate	very low, but possible	-3.059	4.876	5.817	53.580
NPRL1657	Good	low	-8.333	3.426	2.943	107.878
NPRL1658	Good	low	-7.412	0.861	3.692	73.277
NPRL1659	Good	good	-8.524	2.795	2.526	107.878
NPRL1660	Good	good	-9.889	2.302	2.064	107.878
NPRL1661	Moderate	very low, but possible	-8.591	1.359	5.270	99.508
NPRL1662	Good	good	-10.572	-0.230	1.436	116.808
NPRL1663	Good	good	-8.433	3.033	2.520	107.878
NPRL1664	Good	good	-8.082	2.527	1.578	104.214
NPRL1665	Good	good	-6.137	3.669	0.937	87.931
NPRL1666	Good	good	-2.839	5.230	1.554	61.391
NPRL1667	Good	good	-3.059	4.546	2.403	70.321
NPRL1668	Good	low	-4.517	6.531	2.493	61.951
NPRL1669	Good	low	-3.272	3.648	2.819	79.471
NPRL1670	Good	very low, but possible	-1.110	-3.164	4.944	67.916
NPRL1671	Good	very low, but possible	-1.499	-3.495	4.882	67.916
NPRL1672	Good	low	-4.232	-1.301	4.020	94.147
NPRL1673	Good	low	-0.485	-2.449	3.434	88.732
NPRL1674	Good	low	-3.457	-3.569	3.121	88.732
NPRL1675	Poor	very low, but possible	-4.749	4.225	6.554	83.287
NPRL1676	Good	low	-1.100	-0.849	2.980	81.337
NPRL1677	Moderate	very low, but possible	-4.091	-1.301	5.751	56.341

NPRL1678	Poor	very low, but possible	-7.298	-0.944	6.418	99.873
NPRL1679	Poor	very low, but possible	-4.834	-1.995	6.245	94.458
NPRL1680	Good	low	-0.009	3.916	3.579	98.638
NPRL1681	Poor	low	-5.462	-8.711	4.391	148.954
NPRL1682	Poor	very low, but possible	-5.105	-0.776	5.321	99.873
NPRL1683	Good	low	-4.449	-0.486	3.242	102.828
NPRL1684	Good	very low, but possible	-4.498	-0.031	4.529	94.458
NPRL1685	Good	low	-4.051	0.489	2.830	102.828
NPRL1686	Good	low	-4.391	-0.207	4.288	84.851
NPRL1687	Moderate	low	-3.273	-0.679	3.745	118.843
NPRL1688	Poor	very low, but possible	-5.757	-2.035	5.805	99.873
NPRL1689	Good	low	-4.929	-1.836	3.726	102.828
NPRL1690	Moderate	very low, but possible	-5.160	-1.290	5.013	94.458
NPRL1691	Good	low	-4.896	-0.534	3.314	102.828
NPRL1692	Very poor	very low, but possible	-7.175	-0.099	7.361	99.873
NPRL1693	Poor	very low, but possible	-4.695	-1.834	5.282	102.828
NPRL1694	Poor	very low, but possible	-4.702	-0.242	6.569	94.458
NPRL1695	Poor	low	-5.642	-11.112	5.153	118.843
NPRL1696	Poor	very low, but possible	-5.364	-1.682	6.648	99.873
NPRL1697	Moderate	very low, but possible	-4.842	-1.664	4.569	102.828
NPRL1698	Poor	very low, but possible	-7.828	-4.596	6.132	99.873
NPRL1699	Good	low	-5.165	-3.844	4.053	102.828
NPRL1700	Very poor	Extremely low	-6.025	-1.598	7.624	114.928
NPRL1701	Poor	very low, but possible	-4.408	-0.418	5.546	117.884
NPRL1702	Poor	low	-2.911	-5.704	4.440	118.843
NPRL1703	Moderate	low	-5.178	-9.124	3.924	118.843
NPRL1704	Poor	very low, but possible	1.803	-5.723	6.359	133.898
NPRL1705	Poor	very low, but possible	-6.968	-3.602	4.917	120.689
NPRL1706	Moderate	low	-5.240	-3.193	2.838	123.644
NPRL1711	Moderate	low	-3.437	-5.012	3.051	118.843
NPRL1712	Moderate	low	-4.297	-5.523	3.535	118.843
NPRL1713	Poor	good	-4.715	-9.159	2.709	139.658
NPRL1716	Poor	low	-3.212	-6.035	4.378	118.843
NPRL1717	Moderate	low	-5.480	-9.114	3.862	118.843
NPRL1718	Poor	low	-5.943	-11.443	5.091	118.843
NPRL1719	Poor	Extremely low	-1.835	3.918	6.647	55.417
NPRL1720	Moderate	very low, but possible	-3.404	2.804	5.860	76.232
NPRL1721	Moderate	very low, but possible	-2.625	1.034	5.579	79.747
NPRL1722	Good	very low, but possible	-1.534	-0.066	5.044	67.916
NPRL1723	Moderate	very low, but possible	-2.854	1.991	5.932	73.642
NPRL1724	Good	very low, but possible	-2.572	2.350	4.233	82.013
NPRL1725	Very poor	Extremely low	-3.932	2.042	7.972	73.642
NPRL1726	Poor	very low, but possible	-3.935	1.621	6.273	82.013
NPRL1727	Very poor	Extremely low	-3.932	2.042	7.972	73.642
NPRL1728	Good	low	-1.983	0.232	3.780	85.217
NPRL1729	Good	low	-2.578	-1.254	4.104	98.027
NPRL1730	Moderate	Extremely low	-3.476	3.535	6.389	60.638
NPRL1731	Very poor	Extremely low	-3.070	0.909	7.259	73.642
NPRL1732	Moderate	very low, but possible	-2.761	0.889	5.560	82.013
NPRL1733	Poor	very low, but possible	-5.020	-7.580	6.144	98.027
NPRL1734	Poor	very low, but possible	-2.525	-1.989	5.431	98.027
NPRL1735	Poor	very low, but possible	-4.250	-1.784	6.744	73.642
NPRL1736	Good	very low, but possible	-4.957	-0.605	5.044	82.013
NPRL1737	Very poor	Extremely low	-4.064	0.289	7.648	73.642
NPRL1738	Moderate	very low, but possible	-4.067	1.022	5.948	82.013
NPRL1739	Moderate	low	-4.163	-5.490	4.915	98.027
NPRL1740	Very poor	Extremely low	-1.495	0.211	8.722	88.697
NPRL1741	Very poor	Extremely low	-0.405	0.943	7.023	97.068
NPRL1742	Poor	very low, but possible	-3.977	-5.306	5.819	98.027
NPRL1743	Poor	very low, but possible	-4.487	-1.625	5.528	94.458
NPRL1744	Good	low	-5.096	-0.379	3.829	102.828
NPRL1748	Moderate	low	-4.036	-6.173	3.700	118.843
NPRL1751	Very poor	Extremely low	-3.779	5.546	7.910	50.926
NPRL1752	Very poor	Extremely low	-2.257	5.060	8.869	49.377
NPRL1753	Poor	Extremely low	-3.681	3.627	6.925	64.347
NPRL1754	Poor	very low, but possible	-5.175	0.940	6.211	82.572
NPRL1755	Good	low	-3.237	0.691	4.132	85.528
NPRL1756	Poor	very low, but possible	-5.837	-0.319	6.695	82.572
NPRL1757	Good	very low, but possible	-3.899	-0.501	4.616	85.528
NPRL1758	Moderate	low	-3.730	-3.680	4.487	101.542
NPRL1759	Good	low	-2.880	-3.011	4.003	101.542
NPRL1760	Very poor	very low, but possible	-6.501	-2.948	7.022	82.572
NPRL1761	Good	very low, but possible	-5.622	-2.210	4.944	85.528
NPRL1762	Very poor	Extremely low	-6.855	-0.870	7.927	82.572
NPRL1763	Poor	very low, but possible	-4.501	-1.092	5.848	85.528
NPRL1764	Moderate	low	-4.465	-7.247	4.815	101.542

NPRL1765	Poor	very low, but possible	-4.447	2.606	6.214	90.578
NPRL1766	Very poor	Extremely low	-7.350	0.883	8.251	82.572
NPRL1767	Poor	very low, but possible	-4.601	-0.038	6.172	85.528
NPRL1768	Good	low	-2.033	-2.323	3.853	92.246
NPRL1769	Poor	low	-5.322	-9.337	6.043	101.542
NPRL1770	Poor	low	-4.422	-7.063	5.719	101.542
NPRL1771	Good	very low, but possible	-3.261	1.549	4.561	78.133
NPRL1772	Very poor	Extremely low	-4.329	3.358	7.640	90.578
NPRL1773	Very poor	very low, but possible	-5.939	3.675	7.640	90.578
NPRL1774	Very poor	Extremely low	-4.411	3.216	7.640	90.578
NPRL1775	Very poor	very low, but possible	-6.056	4.788	7.989	90.578
NPRL1776	Very poor	Extremely low	-4.446	4.126	7.989	90.578
NPRL1777	Very poor	Extremely low	-2.245	3.751	7.862	81.648
NPRL1778	Very poor	Extremely low	-4.606	4.359	9.314	90.578
NPRL1779	Very poor	Extremely low	-2.847	2.217	7.201	90.578
NPRL1780	Very poor	Extremely low	-6.358	4.218	8.396	73.277
NPRL1781	Very poor	Extremely low	-6.750	3.630	8.396	73.277
NPRL1782	Very poor	Extremely low	-7.641	4.268	8.441	73.277
NPRL1783	Very poor	very low, but possible	-3.533	25.026	8.290	35.160
NPRL1784	Very poor	very low, but possible	-5.939	4.021	7.640	90.578
NPRL1785	Very poor	Extremely low	-5.939	3.433	7.640	90.578
NPRL1786	Very poor	Extremely low	-6.830	4.071	7.686	90.578
NPRL1787	Very poor	very low, but possible	-2.480	24.061	7.534	52.461
NPRL1788	Very poor	too soluble	-10.963	5.182	14.702	184.670
NPRL1789	Very poor	too soluble	-10.259	4.757	16.990	150.069
NPRL1790	Very poor	too soluble	-11.150	5.219	17.081	150.069
NPRL1791	Good	good	-2.483	2.781	3.460	35.160
NPRL1792	Good	good	-4.485	3.082	3.460	35.160
NPRL1793	Good	good	-2.565	1.865	3.460	35.160
NPRL1794	Good	low	-1.275	2.908	3.682	26.230
NPRL1795	Good	very low, but possible	-1.461	0.966	4.182	72.717
NPRL1796	Moderate	very low, but possible	-1.026	1.023	5.882	64.347
NPRL1797	Very poor	Extremely low	-0.470	3.859	8.077	52.461
NPRL1798	Moderate	very low, but possible	0.067	4.019	6.377	60.832
NPRL1799	Good	opti mal	-5.359	-1.471	1.769	47.046
NPRL1800	Good	good	-3.794	1.875	2.118	47.046
NPRL1801	Good	good	-4.690	0.587	1.288	34.601
NPRL1802	Good	good	-4.898	3.793	3.443	47.046
NPRL1803	Good	good	-3.464	3.187	3.443	47.046
NPRL1804	Good	good	-3.421	3.451	3.451	35.160
NPRL1805	Good	good	-4.118	2.883	2.481	35.160
NPRL1806	Good	good	-3.535	3.392	2.937	35.160
NPRL1807	Good	low	-3.168	5.290	5.278	35.160
NPRL1808	Very poor	very low, but possible	-3.645	5.788	7.104	35.160
NPRL1809	Good	good	-5.488	1.407	1.995	35.160
NPRL1810	Good	good	-4.216	4.323	2.344	35.160
NPRL1811	Good	good	-4.293	4.315	2.867	35.160
NPRL1812	Good	low	-3.004	4.183	3.701	35.160
NPRL 1814	Very poor	Extremely low	-3.748	0.374	12.122	29.745
NPRL 1816	Very poor	Extremely low	0.514	1.714	13.106	0.000
NPRL 1817	Poor	very low, but possible	-9.866	-2.415	6.193	82.766
NPRL 1818	Moderate	very low, but possible	-2.498	0.466	5.858	73.277
NPRL 1819	Poor	low	-2.808	-0.148	5.092	105.978
NPRL 1820	Poor	very low, but possible	-4.944	1.628	6.675	68.421
NPRL 1821	Good	low	-9.756	-6.019	3.049	94.652
NPRL 1822	Good	low	-3.546	-2.867	2.714	85.162
NPRL 1823	Good	low	-5.473	-1.245	3.531	80.306
NPRL 1825	Good	very low, but possible	-4.488	-0.683	4.018	73.277
NPRL 1826	Good	very low, but possible	-4.489	-1.994	3.939	68.421
NPRL 1827	Very poor	Extremely low	-6.809	1.090	7.054	73.836
NPRL 1828	Good	very low, but possible	-6.960	-4.398	3.389	73.836
NPRL 1829	Good	good	-4.405	-4.022	1.948	117.863
NPRL 1830	Good	very low, but possible	-5.712	-0.029	3.965	68.421
NPRL 1831	Good	low	-7.457	-1.311	3.910	85.722
NPRL 1833	Good	low	-6.565	-1.998	3.910	85.722
NPRL 1834	Very poor	very low, but possible	-6.329	-0.872	7.323	87.622
NPRL 1835	Good	opti mal	-7.860	-1.415	0.195	64.347
NPRL 1836	Good	good	-6.095	-5.062	1.127	85.722
NPRL 1837	Good	low	-5.709	-3.540	3.564	88.677
NPRL 1838	Good	low	-6.154	-1.502	2.631	101.122
NPRL 1839	Poor	very low, but possible	-5.478	-0.924	6.943	82.207
NPRL 1840	Good	low	-6.066	-3.688	3.799	94.092
NPRL 1841	Poor	very low, but possible	-8.500	-0.466	6.287	106.537
NPRL 1842	Poor	very low, but possible	-9.452	-0.464	5.943	82.766
NPRL 1843	Moderate	low	-8.356	-3.308	3.143	118.422
NPRL 1844	Poor	Extremely low	-7.438	-0.666	6.533	61.951

NPRL 1847	Good	low	-2.734	-0.657	4.766	88.677
NPRL 1848	Good	low	-2.764	-1.179	2.964	67.861
NPRL 1849	Good	good	-5.296	-3.817	1.622	100.562
NPRL 1850	Good	very low, but possible	-6.292	-0.430	4.010	82.207
NPRL 1851	Good	very low, but possible	-7.136	-1.473	4.289	91.137
NPRL 1852	Very poor	opti mal	-6.389	-5.324	-0.976	187.626
NPRL 1854	Very poor	Extremely low	-3.058	1.851	7.695	47.605
NPRL 1855	Very poor	opti mal	-5.218	-7.370	-1.201	199.511
NPRL 1857	Good	good	-4.466	1.094	2.375	44.091
NPRL 1859	Good	low	-4.405	1.241	4.032	17.860
NPRL 1860	Good	low	-5.223	1.134	3.274	26.790
NPRL 1862	Poor	very low, but possible	-7.870	-1.109	5.177	122.783
NPRL 1863	Poor	very low, but possible	-7.245	-1.180	5.623	122.783
NPRL 1864	Poor	very low, but possible	-7.074	-1.488	5.623	122.783
NPRL 1866	Poor	very low, but possible	-6.707	-1.169	4.865	131.713
NPRL 1867	Poor	very low, but possible	-7.110	-1.563	5.160	131.713
NPRL 1868	Poor	very low, but possible	-7.824	-2.414	5.160	131.713
NPRL 1869	Poor	very low, but possible	-7.545	1.303	6.655	122.783
NPRL 1870	Poor	very low, but possible	-7.324	-1.938	6.536	122.783
NPRL 1871	Poor	very low, but possible	-6.174	-1.149	6.070	122.783
NPRL 1873	Good	good	-4.494	-2.988	1.366	84.851
NPRL 1874	Good	good	-5.002	-5.349	0.669	84.851
NPRL 1875	Good	good	-4.627	-6.260	1.764	111.231
NPRL 1876	Good	opti mal	-4.995	-1.166	0.098	116.249
NPRL 1878	Good	low	-0.001	5.263	3.996	35.720
NPRL 1880	Moderate	too soluble	-4.645	-2.840	-0.894	51.210
NPRL 1881	Poor	too soluble	-7.524	-7.902	-1.949	62.471
NPRL 1882	Good	good	0.218	-5.531	3.292	83.262
NPRL 1883	Poor	too soluble	-0.634	-4.882	-1.822	51.210
NPRL 1884	Very poor	too soluble	-3.875	-8.052	-2.256	73.732
NPRL 1885	Good	good	-7.759	1.676	2.581	47.971
NPRL 1886	Good	good	-8.051	-0.343	2.058	74.202
NPRL 1887	Good	low	-1.637	5.472	3.672	58.866
NPRL 1888	Good	low	-3.880	-1.739	2.808	26.230
NPRL 1889	Good	good	-0.093	0.978	2.982	20.815
NPRL 1890	Good	low	-3.599	2.673	3.740	38.785
NPRL 1891	Good	good	-1.228	2.740	2.779	35.160
NPRL 1892	Good	good	-3.959	2.073	0.995	55.976
NPRL 1893	Good	low	-4.264	2.015	2.831	38.785
NPRL 1894	Good	good	-3.681	-0.031	1.169	52.461
NPRL 1895	Good	opti mal	-4.178	1.108	0.790	55.976
NPRL 1896	Good	good	-5.999	1.016	2.345	38.785
NPRL 1897	Good	good	-4.942	2.099	1.276	55.976
NPRL 1898	Good	good	-5.397	0.908	0.774	64.906
NPRL 1899	Good	low	-4.733	0.614	2.831	38.785
NPRL 1900	Good	good	-3.069	1.794	0.996	55.976
NPRL 1901	Good	good	-3.937	4.696	1.455	55.976
NPRL 1902	Good	good	-5.322	0.145	2.329	47.715
NPRL 1903	Good	good	-3.443	1.595	2.551	38.785
NPRL 1904	Good	low	-3.282	3.603	3.009	38.785
NPRL 1905	Good	good	-2.572	-1.010	1.375	52.461
NPRL 1906	Good	good	-1.936	2.445	1.834	52.461
NPRL 1907	Good	good	-2.172	1.029	1.702	52.461
NPRL 1908	Good	low	-3.159	4.159	3.037	38.785
NPRL 1909	Good	low	-3.871	5.097	3.496	38.785
NPRL 1910	Good	good	-6.140	0.573	0.774	64.906
NPRL 1911	Good	good	-5.198	2.065	1.276	55.976
NPRL 1912	Good	low	-4.931	1.689	2.831	38.785
NPRL 1913	Good	good	-5.128	0.473	2.329	47.715
NPRL 1914	Good	low	-4.524	2.933	3.317	38.785
NPRL 1915	Good	good	-2.941	-0.577	1.655	52.461
NPRL 1919	Good	low	-4.722	4.008	3.317	38.785
NPRL 1920	Good	low	-4.686	1.197	2.815	47.715
NPRL 1921	Good	low	-4.492	1.526	2.815	47.715
NPRL 1922	Good	good	-4.028	-1.301	1.929	17.300
NPRL 1923	Good	good	-1.601	-3.292	1.879	58.307
NPRL 1924	Good	good	-10.277	1.212	2.184	42.665
NPRL 1925	Good	good	-10.898	-0.149	1.698	42.665
NPRL 1926	Good	good	-4.899	-2.102	1.611	52.770
NPRL 1927	Good	good	-3.726	-0.901	1.567	43.531
NPRL 1929	Good	good	-2.834	-0.936	2.078	52.461
NPRL 1932	Good	low	-3.005	0.886	3.012	59.600
NPRL 1933	Good	low	-3.428	1.422	3.254	38.785
NPRL 1935	Good	very low, but possible	-2.282	5.509	4.682	38.785
NPRL 1936	Good	low	-1.208	4.359	2.641	55.976
NPRL 1937	Good	good	-3.023	-1.007	2.061	61.391

NPRL 1938	Good	low	-3.338	0.927	3.237	47.715
NPRL 1939	Good	good	-2.508	2.910	2.185	55.976
NPRL 1940	Good	low	-3.744	2.286	3.723	47.715
NPRL 1941	Good	very low, but possible	-1.602	3.680	4.404	38.785
NPRL 1942	Good	low	-1.850	2.513	3.676	59.600
NPRL 1943	Good	low	-1.328	3.124	2.363	55.976
NPRL 1944	Good	low	-2.343	2.715	3.918	38.785
NPRL 1946	Good	good	-1.833	-0.383	2.334	17.300
NPRL 1947	Good	good	-5.642	0.573	1.787	34.601
NPRL 1948	Good	opti mal	-5.488	-1.140	1.786	38.116
NPRL 1949	Good	good	-5.029	0.421	1.771	43.531
NPRL 1950	Good	good	-5.813	-1.340	1.883	35.160
NPRL 1951	Good	low	-1.174	2.854	3.739	35.160
NPRL 1952	Good	low	-1.727	-0.464	3.169	52.461
NPRL 1953	Poor	opti mal	-7.756	-18.132	-0.514	148.168
NPRL 1954	Good	low	-1.879	-0.065	3.153	61.391
NPRL 1955	Good	good	-7.041	-3.875	2.203	38.785
NPRL 1956	Good	low	-1.722	-1.172	3.629	38.785
NPRL 1957	Good	good	-5.947	-10.960	2.186	47.715
NPRL 1958	Good	good	-7.468	-10.026	1.719	89.346
NPRL 1959	Good	good	-6.037	-0.686	2.187	47.715
NPRL 1960	Good	low	-5.468	-1.048	2.485	56.645
NPRL 1961	Good	good	-7.091	-2.380	2.127	77.460
NPRL 1962	Good	low	-4.217	-2.270	3.636	56.645
NPRL 1963	Good	low	-6.232	-3.024	2.469	65.575
NPRL 1964	Good	good	-7.952	-4.581	1.702	98.276
NPRL 1965	Good	good	-7.320	-4.915	2.111	86.390
NPRL 1966	Good	good	-6.429	-1.460	2.187	47.715
NPRL 1967	Good	good	-6.131	-2.749	2.187	47.715
NPRL 1968	Good	good	-6.244	-3.006	2.029	55.976
NPRL 1969	Good	low	-2.598	-0.814	4.339	44.091
NPRL 1970	Good	low	-3.670	0.240	3.502	87.622
NPRL 1971	Good	low	-4.362	-0.973	3.085	87.622
NPRL 1972	Good	low	-2.979	2.016	4.631	87.622
NPRL 1973	Very poor	opti mal	-8.482	-16.331	-0.549	157.098
NPRL 1974	Moderate	very low, but possible	-2.525	2.589	5.048	87.622
NPRL 1975	Good	low	-3.433	0.063	4.602	87.622
NPRL 1976	Good	good	-3.002	-2.089	2.272	87.622
NPRL 1977	Very poor	opti mal	-11.327	-13.936	-0.510	232.276
NPRL 1979	Good	opti mal	-7.559	-1.416	0.195	64.347
NPRL 1980	Good	good	-5.810	1.491	2.535	35.160
NPRL 1981	Moderate	opti mal	-8.770	-16.241	-0.512	139.797
NPRL 1982	Very poor	good	-8.622	-18.469	-2.259	220.104
NPRL 1983	Good	good	-2.522	-2.354	3.331	55.976
NPRL 1984	Good	low	-2.753	12.106	3.208	70.881
NPRL 1985	Good	good	-0.224	-8.418	2.021	113.007
NPRL 1986	Good	low	-1.346	3.097	3.038	79.811
NPRL 1987	Very poor	good	-2.861	-19.766	-0.965	228.475
NPRL 1988	Very poor	low	-1.502	-23.491	-1.545	270.106
NPRL 1989	Good	low	-3.785	3.396	5.058	67.861
NPRL 1990	Poor	low	-3.149	6.563	6.659	79.747
NPRL 1991	Very poor	low	-2.035	-0.277	2.399	166.810
NPRL 1992	Very poor	very low, but possible	1.238	-1.582	6.022	163.678
NPRL 1993	Very poor	very low, but possible	1.017	-3.243	5.055	184.493
NPRL 1994	Poor	very low, but possible	3.208	-4.285	5.252	148.168
NPRL 1995	Very poor	very low, but possible	2.591	-6.871	4.285	168.984
NPRL 1996	Poor	good	-8.188	-17.504	0.268	145.213
NPRL 1997	Very poor	good	-6.558	-17.738	0.436	168.984
NPRL 1998	Good	good	-5.188	1.599	1.404	69.203
NPRL 2001	Very poor	opti mal	-2.218	-8.325	-1.463	164.910
NPRL 2002	Very poor	good	-8.720	-14.231	1.144	151.683
NPRL 2003	Poor	very low, but possible	-2.579	2.259	6.546	58.931
NPRL 2004	Good	low	-4.422	-8.790	3.652	47.715
NPRL 2005	Good	low	-5.378	-9.245	2.485	56.645
NPRL 2006	Good	low	-4.513	-0.592	3.652	47.715
NPRL 2007	Good	good	-7.559	-1.829	1.719	89.346
NPRL 2008	Moderate	very low, but possible	-2.433	1.733	5.048	87.622
NPRL 2009	Good	low	-3.117	1.069	3.123	82.207
NPRL 2010	Good	low	-6.075	-0.206	3.331	87.622
NPRL 2011	Good	good	-5.278	-3.993	2.168	82.207
NPRL 2014	Moderate	good	-6.426	-14.338	0.678	136.283
NPRL 2015	Very poor	good	-6.288	-16.473	0.436	157.098
NPRL 2016	Very poor	low	-6.291	-13.617	2.688	181.428
NPRL 2017	Poor	very low, but possible	-6.039	3.051	5.789	102.463
NPRL 2018	Poor	very low, but possible	-4.417	4.089	5.410	97.048
NPRL 2019	Poor	very low, but possible	-6.001	3.555	5.040	137.065

NPRL 2020	Good	very low, but possible	-3.209	2.128	4.660	47.155
NPRL 2021	Very poor	low	-4.759	-9.399	1.770	276.303
NPRL 2023	Good	good	-1.098	-1.798	2.486	58.931
NPRL 2025	Good	good	-5.516	-1.393	2.042	76.791
NPRL 2026	Good	low	-1.507	7.007	3.653	26.230
NPRL 2027	Good	low	-3.433	2.990	2.682	26.230
NPRL 2028	Good	low	-3.292	3.796	2.682	26.230
NPRL 2029	Good	good	-4.963	0.726	1.193	67.861
NPRL 2032	Good	opti mal	-6.080	-2.477	0.442	43.531
NPRL 2033	Good	opti mal	-5.339	-4.205	1.146	47.046
NPRL 2034	Good	low	-1.784	3.530	3.146	35.160
NPRL 2037	Good	low	-1.040	3.172	3.146	35.160
NPRL 2038	Good	good	-3.813	-0.624	2.175	35.160
NPRL 2039	Good	good	-7.333	3.729	2.434	76.791
NPRL 2042	Good	low	-3.461	-0.525	2.454	73.277
NPRL 2043	Good	good	-3.578	0.046	2.164	76.791
NPRL 2049	Good	opti mal	-2.049	-1.166	1.905	55.417
NPRL 2050	Good	good	-6.318	-2.717	1.480	43.531
NPRL 2051	Good	good	-1.680	0.833	2.687	43.531
NPRL 2052	Good	good	-2.810	-3.629	1.936	43.531
NPRL 2054	Good	good	-6.252	-1.092	1.412	43.531
NPRL 2055	Good	low	-3.939	1.631	3.212	35.160
NPRL 2056	Good	good	-5.917	-1.420	1.723	76.791
NPRL 2057	Good	low	-2.628	0.673	3.669	35.160
NPRL 2058	Good	low	-1.846	4.653	4.639	35.160
NPRL 2059	Good	good	-3.751	-1.651	2.179	76.791
NPRL 2061	Good	low	-3.085	-1.727	2.914	53.021
NPRL 2062	Good	low	-5.908	-0.942	2.458	53.021
NPRL 2065	Good	low	-2.767	-0.023	3.133	35.160
NPRL 2066	Good	low	-3.800	0.498	3.145	65.016
NPRL 2067	Good	good	-6.967	-1.107	1.097	73.386
NPRL 2068	Good	low	-2.407	2.161	3.522	35.160
NPRL 2069	Good	good	-4.885	0.555	2.551	35.160
NPRL 2070	Good	good	-1.333	-1.798	2.392	44.091
NPRL 2071	Good	good	-1.369	-6.145	1.941	67.861
NPRL 2072	Good	good	-2.514	-3.046	2.193	52.461
NPRL 2073	Good	good	-3.379	-3.029	1.463	52.461
NPRL 2074	Good	low	-0.275	-1.739	4.831	61.391
NPRL 2076	Good	good	-5.857	-1.050	0.889	43.531
NPRL 2077	Good	low	-1.560	5.337	3.592	35.160
NPRL 2078	Good	low	-4.038	1.818	2.621	35.160
NPRL 2079	Good	good	-7.094	-1.204	1.422	73.277
NPRL 2080	Good	good	-6.806	-0.633	1.132	76.791
NPRL 2083	Good	opti mal	-5.248	-1.035	0.868	67.861
NPRL 2085	Good	low	-2.757	10.062	4.894	35.160
NPRL 2086	Good	low	-3.987	5.741	3.923	35.160
NPRL 2088	Good	low	-4.433	2.239	2.843	26.230
NPRL 2089	Good	good	-2.538	-2.254	2.115	52.461
NPRL 2090	Good	good	-6.094	-1.383	1.402	43.531
NPRL 2091	Good	low	-1.692	5.572	4.106	35.160
NPRL 2092	Good	low	-3.716	2.005	3.135	35.160
NPRL 2093	Good	good	-5.693	-0.719	1.646	76.791
NPRL 2096	Good	opti mal	-4.236	-3.179	1.105	69.762
NPRL 2097	Good	good	-3.452	-4.268	1.669	52.461
NPRL 2098	Good	good	-2.049	-0.255	1.921	43.531
NPRL 2099	Good	low	0.312	6.668	4.625	35.160
NPRL 2100	Good	low	-1.590	3.362	3.654	35.160
NPRL 2101	Good	opti mal	-5.278	-1.894	1.162	55.976
NPRL 2102	Good	good	-6.602	-2.863	0.956	43.531
NPRL 2103	Good	low	-2.305	3.164	3.660	35.160
NPRL 2105	Good	good	-7.551	-2.173	1.200	76.791
NPRL 2112	Good	very low, but possible	-1.205	2.343	5.073	51.902
NPRL 2113	Moderate	very low, but possible	-3.725	1.088	5.718	64.347
NPRL 2114	Moderate	very low, but possible	-3.987	-0.004	5.683	79.747
NPRL 2115	Moderate	very low, but possible	-1.988	2.349	6.212	41.631
NPRL 2116	Poor	Extremely low	-0.738	-0.108	6.672	47.046
NPRL 2117	Moderate	very low, but possible	-3.209	2.814	5.842	81.648
NPRL 2118	Poor	low	-5.855	3.238	5.472	121.665
NPRL 2119	Moderate	Extremely low	-1.595	0.811	5.944	52.461
NPRL 2120	Good	very low, but possible	-1.873	-1.214	5.383	50.561
NPRL 2121	Poor	very low, but possible	-4.398	1.780	5.473	95.993
NPRL 2122	Good	low	-4.055	0.855	3.377	97.048
NPRL 2123	Good	low	-1.634	-2.004	4.617	83.262
NPRL 2124	Very poor	opti mal	-6.954	-14.159	-1.349	168.821
NPRL 2125	Very poor	opti mal	-7.559	-12.648	-1.124	156.936
NPRL 2127	Good	good	-7.348	-0.492	1.241	52.461

NPRL 2128	Good	opti mal	-5.853	-4.126	0.722	69.762
NPRL 2129	Good	low	-3.909	1.206	2.468	61.391
NPRL 2130	Good	good	-5.410	-2.334	1.399	88.677
NPRL 2131	Good	good	-4.458	0.379	2.188	52.461
NPRL 2132	Good	good	-5.634	0.668	1.687	52.461
NPRL 2134	Good	good	-2.920	-6.177	1.862	67.861
NPRL 2135	Good	good	-4.149	0.682	1.610	78.692
NPRL 2137	Good	good	-4.652	-0.540	1.755	52.461
NPRL 2139	Good	opti mal	-6.953	-4.668	-0.627	64.906
NPRL 2140	Good	opti mal	-5.041	-1.892	0.198	94.092
NPRL 2144	Good	low	-1.761	1.718	3.443	35.160
NPRL 2145	Good	opti mal	-5.190	-1.951	0.430	94.092
NPRL 2146	Good	good	-3.443	-1.554	2.314	47.046
NPRL 2147	Good	low	-3.161	-0.911	2.701	55.976
NPRL 2148	Good	low	-1.439	5.296	5.202	44.091
NPRL 2149	Good	opti mal	-3.679	-2.424	1.049	55.417
NPRL 2150	Good	good	-4.307	4.337	3.211	61.391
NPRL 2151	Good	opti mal	-3.506	-7.414	1.069	67.861
NPRL 2153	Good	good	-2.430	-4.596	2.138	76.232
NPRL 2154	Good	opti mal	-5.594	-4.126	0.382	55.417
NPRL 2155	Good	opti mal	-4.838	-5.891	0.841	47.355
NPRL 2156	Good	good	-3.652	0.991	2.577	47.046
NPRL 2158	Good	opti mal	-5.292	-4.505	0.498	64.347
NPRL 2170	Very poor	low	-13.811	-6.918	1.521	171.444
NPRL 2171	Good	low	-7.905	-4.569	2.757	111.953
NPRL 2172	Moderate	low	-6.990	-2.541	3.137	117.368
NPRL 2173	Moderate	low	-7.480	-1.966	3.803	117.368
NPRL 2174	Good	low	-7.632	-3.001	2.858	108.438
NPRL 2175	Good	low	-7.221	-3.652	1.999	101.122
NPRL 2176	Very poor	good	-10.117	-6.074	0.763	160.613
NPRL 2177	Very poor	good	-9.068	-2.271	2.216	151.970
NPRL 2178	Very poor	good	-9.635	-3.001	1.964	151.970
NPRL 2179	Very poor	good	-8.876	-1.164	2.843	151.970
NPRL 2180	Very poor	good	-9.068	-2.623	2.422	151.970
NPRL 2181	Very poor	good	-8.322	-1.513	2.963	151.970
NPRL 2182	Very poor	good	-9.459	-2.297	2.387	151.970
NPRL 2183	Good	very low, but possible	-5.371	-0.745	5.468	67.861
NPRL 2184	Good	very low, but possible	-4.491	-1.837	5.525	67.861
NPRL 2185	Very poor	Extremely low	-0.274	0.055	7.553	20.815
NPRL 2186	Very poor	good	-4.658	-10.997	-0.114	186.284
NPRL 2187	Very poor	low	-5.056	-15.378	-1.861	266.591
NPRL 2188	Good	good	-4.029	2.041	1.867	73.410
NPRL 2189	Good	low	-2.543	4.479	3.005	61.866
NPRL 2190	Good	very low, but possible	-0.061	5.852	4.432	44.006
NPRL 2191	Good	good	-5.827	-0.639	1.854	73.127
NPRL 2192	Good	good	-6.399	-1.494	1.854	73.127
NPRL 2193	Good	low	-7.244	2.160	3.709	102.773
NPRL 2194	Good	low	-1.997	4.973	3.021	52.936
NPRL 2195	Good	low	-0.503	2.164	3.288	52.267
NPRL 2196	Good	good	-1.970	1.941	2.343	69.568
NPRL 2197	Good	good	-3.131	-1.098	2.154	54.598
NPRL 2198	Good	good	-9.775	-0.200	1.350	82.903
NPRL 2199	Good	opti mal	-10.222	-0.836	0.036	90.649
NPRL 2200	Good	good	-8.105	-3.394	0.783	73.348
NPRL 2201	Good	low	-4.274	-0.384	2.856	73.348
NPRL 2202	Good	good	-14.602	1.135	1.470	113.840
NPRL 2203	Good	good	-2.923	-0.488	2.479	58.392
NPRL 2204	Good	good	-3.692	2.223	2.212	49.277
NPRL 2205	Good	good	-5.011	3.687	2.195	58.207
NPRL 2206	Moderate	very low, but possible	-3.508	10.566	5.381	78.475
NPRL 2207	Poor	very low, but possible	-0.478	11.569	6.323	78.475
NPRL2208	Good	good	-2.965	-1.872	1.813	117.863
NPRL2209	Moderate	good	-4.177	-2.597	0.723	138.679
NPRL2210	Good	low	-0.114	-2.813	2.599	61.951
NPRL2211	Good	low	-0.114	-2.813	2.599	61.951
NPRL2212	Good	low	-0.547	-3.834	2.895	61.951
NPRL2213	Good	low	-0.281	-3.381	2.911	53.021
NPRL2214	Good	low	-0.735	-2.128	3.067	53.021
NPRL2215	Good	low	-0.296	0.599	3.032	64.906
NPRL2216	Good	low	-0.683	1.404	4.499	47.605
NPRL2217	Good	low	-1.346	-0.851	3.231	64.906
NPRL2218	Good	low	-0.296	0.599	3.032	64.906
NPRL2219	Good	good	-3.581	0.807	2.335	82.766
NPRL2220	Very poor	opti mal	-2.123	-17.061	-1.777	157.098
NPRL2221	Good	very low, but possible	-0.545	3.680	5.594	55.417
NPRL2222	Good	very low, but possible	-1.561	2.260	4.804	72.717

NPRL2223	Good	very low, but possible	-0.554	4.946	5.695	51.902
NPRL2224	Good	very low, but possible	-3.622	0.937	5.167	55.976
NPRL2225	Very poor	Extremely low	-0.690	1.821	7.349	20.815
NPRL2226	Very poor	Extremely low	0.179	1.824	7.403	20.815
NPRL2227	Very poor	Extremely low	-2.060	2.833	7.349	20.815
NPRL2228	Very poor	Extremely low	-2.334	2.969	7.417	20.815
NPRL2229	Very poor	Extremely low	0.765	2.056	7.542	20.815
NPRL2230	Moderate	Extremely low	-3.131	2.901	6.416	38.116
NPRL2231	Moderate	very low, but possible	-1.926	0.991	6.223	38.116
NPRL2232	Very poor	Extremely low	-0.372	3.396	7.212	20.815
NPRL2233	Very poor	Extremely low	-0.428	2.424	7.543	20.815
NPRL2234	Very poor	Extremely low	-0.984	2.997	8.060	20.815
NPRL2235	Moderate	very low, but possible	-1.715	1.074	6.326	41.631
NPRL2236	Very poor	Extremely low	1.806	2.836	8.062	20.815
NPRL2237	Very poor	Extremely low	-0.801	2.641	7.303	20.815
NPRL2238	Very poor	Extremely low	-0.801	2.641	7.303	20.815
NPRL2239	Very poor	Extremely low	-0.596	3.722	7.303	20.815
NPRL2240	Moderate	very low, but possible	-1.753	2.055	6.266	41.631
NPRL2241	Moderate	very low, but possible	-2.852	1.429	6.248	41.631
NPRL2242	Moderate	very low, but possible	-1.331	2.946	5.980	29.745
NPRL2243	Very poor	Extremely low	0.036	3.508	7.360	20.815
NPRL2244	Moderate	Extremely low	-0.164	2.698	6.571	20.815
NPRL2245	Very poor	Extremely low	1.598	3.219	8.019	17.300
NPRL2246	Good	low	-0.230	-0.824	3.803	76.232
NPRL2247	Very poor	Extremely low	-2.435	2.147	7.404	52.461
NPRL2248	Very poor	very low, but possible	0.029	1.731	7.212	41.631
NPRL2249	Very poor	Extremely low	-4.483	1.253	8.419	26.790
NPRL2250	Very poor	Extremely low	-0.130	3.513	7.360	20.815
NPRL2251	Very poor	Extremely low	-0.431	4.206	7.360	20.815
NPRL2252	Very poor	Extremely low	-4.483	-0.123	8.373	26.790
NPRL2253	Very poor	Extremely low	-0.274	1.830	7.599	20.815
NPRL2254	Very poor	Extremely low	0.094	3.206	8.119	20.815
NPRL2255	Very poor	Extremely low	0.094	3.206	8.119	20.815
NPRL2256	Good	opti mal	-1.375	-5.223	1.379	12.810
NPRL2257	Good	good	6.256	-9.819	1.216	53.420
NPRL2258	Very poor	too soluble	-3.038	-10.939	-2.822	92.440
NPRL2259	Moderate	opti mal	-3.073	-7.642	-0.474	44.834
NPRL2260	Good	good	-1.544	-4.012	2.048	50.764
NPRL2261	Good	low	2.039	3.850	3.495	94.855
NPRL2262	Good	low	-1.672	1.490	4.054	98.370
NPRL2263	Very poor	opti mal	-3.274	-8.533	-2.414	80.555
NPRL2266	Good	good	4.276	-6.184	1.262	65.305
NPRL2267	Good	low	-0.410	-5.918	2.835	46.634
NPRL2268	Good	good	7.944	-3.361	2.435	39.223
NPRL2269	Good	low	0.985	-0.975	2.597	61.939
NPRL2270	Good	good	-0.327	-2.558	1.808	36.119
NPRL2271	Good	good	-1.227	-5.767	1.362	23.985
NPRL2272	Good	very low, but possible	-1.149	-0.064	3.979	60.253
NPRL2273	Good	good	-7.743	2.161	1.418	85.219
NPRL2274	Good	low	-2.879	-0.419	2.128	61.939
NPRL2275	Moderate	opti mal	-4.071	-17.073	-0.687	136.027
NPRL2276	Good	good	-5.454	3.261	2.185	52.518
NPRL2277	Good	low	-3.549	-3.572	2.456	35.708
NPRL2278	Good	very low, but possible	-4.992	1.297	3.770	94.363
NPRL2279	Good	good	-2.699	-3.774	1.386	48.004
NPRL2280	Good	good	-4.715	-1.549	1.321	36.119
NPRL2282	Good	good	-4.509	-5.591	0.230	56.934
NPRL2283	Good	low	1.048	-1.744	2.103	68.624
NPRL2284	Good	low	1.048	-1.744	2.103	68.624
NPRL2285	Good	good	1.761	-6.078	-0.399	68.475
NPRL2286	Good	good	1.761	-6.078	-0.399	68.475
NPRL2287	Good	good	1.761	-6.078	-0.399	68.475
NPRL2288	Good	good	1.761	-6.078	-0.399	68.475
NPRL2289	Good	good	1.761	-6.078	-0.399	68.475
NPRL2290	Good	good	1.761	-6.078	-0.399	68.475
NPRL2291	Good	low	-2.563	-7.887	1.890	61.790
NPRL2293	Moderate	very low, but possible	1.998	-8.313	4.607	97.910
NPRL2294	Good	very low, but possible	1.220	-9.874	4.425	97.910
NPRL2295	Good	good	-2.632	-6.355	1.750	35.093
NPRL2296	Good	low	-1.574	-5.648	2.482	53.420
NPRL2297	Good	very low, but possible	3.662	-7.938	4.655	62.749
NPRL2298	Moderate	very low, but possible	1.998	-8.313	4.607	97.910
NPRL2299	Good	low	-2.563	-7.887	1.890	61.790
NPRL2300	Good	low	-3.459	-5.969	1.862	61.790
NPRL2301	Moderate	low	0.414	-10.339	3.126	118.725
NPRL2302	Good	good	-0.937	-3.741	1.507	57.793

NPRL2303	Good	good	-4.176	-7.267	0.918	55.909
NPRL2304	Very poor	low	-2.293	-1.266	8.126	49.439
NPRL2305	Good	low	1.738	-3.462	2.233	13.261
NPRL2306	Good	good	-5.094	-3.960	2.611	30.111
NPRL2307	Good	good	-5.040	-0.468	0.668	85.925
NPRL2308	Good	very low, but possible	4.187	2.890	5.352	20.403
NPRL2309	Good	good	-2.110	-2.534	1.807	25.620
NPRL2310	Moderate	opti mal	-0.390	-5.369	-1.875	90.267
NPRL2311	Good	good	-3.481	-8.416	1.216	53.913
NPRL2312	Good	low	-5.044	-6.901	2.500	80.509
NPRL2313	Good	low	-1.058	-3.108	3.178	62.466
NPRL2314	Good	low	2.061	0.315	3.182	32.325
NPRL2315	Good	low	1.203	3.182	3.765	43.337
NPRL2316	Good	good	2.231	-4.835	2.613	18.259
NPRL2317	Good	low	-4.443	2.021	3.731	92.160
NPRL2318	Good	low	2.297	0.762	3.543	70.869
NPRL2319	Moderate	opti mal	-9.816	-6.580	-1.027	47.411
NPRL2320	Good	good	-0.155	-0.053	3.123	21.740
NPRL2321	Good	good	-8.153	-2.246	1.697	45.166
NPRL2322	Good	low	1.920	-2.641	1.674	19.515
NPRL2323	Moderate	opti mal	1.467	-6.305	-1.274	49.656
NPRL2324	Good	low	-1.595	0.389	3.259	18.407
NPRL2325	Good	good	-6.388	-7.345	0.268	60.105
NPRL2326	Moderate	good	-6.354	-8.576	-0.670	42.245
NPRL2327	Good	low	0.889	-0.499	2.218	27.520
NPRL2328	Good	good	-3.798	-10.885	0.561	55.973
NPRL2329	Good	good	-3.142	2.044	2.929	67.861
NPRL2330	Very poor	low	-11.422	-20.578	-0.580	313.638
NPRL2331	Good	low	-3.575	0.001	4.194	88.677
NPRL2332	Good	good	2.288	-0.765	2.615	67.861
NPRL2333	Very poor	too soluble	-4.551	-16.283	-5.062	179.255
NPRL2334	Good	low	2.425	0.566	2.361	63.209
NPRL2335	Very poor	good	-3.410	-16.516	-0.808	165.057
NPRL2336	Very poor	good	-7.237	-18.087	-1.061	172.519
NPRL2337	Very poor	opti mal	-0.660	-16.062	-1.895	157.098
NPRL2338	Very poor	opti mal	-3.036	-17.210	0.208	200.630
NPRL2339	Very poor	too soluble	-3.997	-11.083	-3.639	101.370
NPRL2340	Very poor	too soluble	-3.101	-11.420	-3.174	109.741
NPRL2341	Very poor	too soluble	-3.997	-11.083	-3.639	101.370
NPRL2343	Very poor	opti mal	-6.875	-11.088	-2.502	117.617
NPRL2347	Very poor	too soluble	-3.419	-10.005	-3.124	101.370
NPRL2348	Very poor	too soluble	-2.444	-12.161	-3.533	113.256
NPRL2349	Moderate	opti mal	-2.314	-8.765	-1.884	71.625
NPRL2350	Very poor	opti mal	-4.716	-7.856	-2.791	110.301
NPRL2352	Very poor	opti mal	-6.106	-9.406	-2.510	136.531
NPRL2354	Very poor	opti mal	-8.472	-8.147	-2.539	153.832
NPRL2355	Very poor	opti mal	-2.776	-9.435	-2.765	97.856
NPRL2356	Very poor	Extremely low	0.040	-3.686	6.187	215.471
NPRL2357	Poor	very low, but possible	-2.337	1.941	6.308	106.537
NPRL2358	Very poor	Extremely low	-1.533	-0.511	6.187	215.471
NPRL2359	Very poor	low	-14.104	-56.911	-2.548	448.424
NPRL2360	Very poor	low	-14.223	-56.865	-2.091	448.424
NPRL2361	Very poor	low	-11.568	-44.775	-1.451	431.123
NPRL2362	Moderate	low	-2.434	-3.628	4.452	106.537
NPRL2363	Good	good	-2.974	-1.466	3.217	75.000
NPRL2364	Good	good	-4.175	-6.440	1.719	97.048
NPRL2365	Very poor	low	-8.754	-3.160	2.910	209.001
NPRL2366	Very poor	opti mal	-4.659	-19.043	-2.153	187.626
NPRL2367	Very poor	good	-5.694	-19.934	-0.210	177.354
NPRL2368	Good	low	-4.670	4.516	3.224	78.692
NPRL2369	Good	low	-3.263	-1.260	3.589	83.930
NPRL2370	Moderate	very low, but possible	1.036	3.409	6.116	59.491
NPRL2371	Moderate	very low, but possible	-2.143	6.710	5.691	61.951
NPRL2372	Very poor	Extremely low	1.292	2.875	8.606	206.541
NPRL2373	Moderate	low	2.466	-2.492	3.988	109.492
NPRL2374	Very poor	very low, but possible	-3.029	0.577	7.231	189.240
NPRL2375	Very poor	very low, but possible	-2.893	0.581	7.160	197.610
NPRL2376	Good	low	-0.388	-0.500	2.928	97.607
NPRL2377	Poor	low	-0.637	2.486	5.727	109.492
NPRL2378	Good	low	0.239	2.227	4.810	80.306
NPRL2379	Poor	low	-0.543	2.801	5.727	109.492
NPRL2380	Moderate	low	-0.715	5.185	5.169	94.092
NPRL2381	Poor	very low, but possible	-3.169	4.490	5.542	103.022
NPRL2382	Poor	very low, but possible	-0.465	6.208	6.666	80.306
NPRL2383	Good	low	1.249	2.364	3.503	82.207
NPRL2384	Moderate	low	1.168	4.178	5.991	79.747

NPRL2385	Good	good	-2.111	1.035	2.656	82.766
NPRL2386	Good	low	-1.421	5.840	4.611	81.648
NPRL2387	Good	low	0.406	1.600	4.718	85.722
NPRL2388	Good	low	-1.161	-0.535	4.667	67.861
NPRL2389	Poor	very low, but possible	0.452	5.851	6.892	68.421
NPRL2390	Good	low	-0.173	0.943	4.096	97.607
NPRL2391	Moderate	low	-1.911	2.519	4.069	114.908
NPRL2392	Good	low	-1.557	2.397	4.311	94.092
NPRL2393	Poor	low	-3.658	5.873	6.689	79.747
NPRL2395	Good	good	-0.015	-0.399	3.179	68.421
NPRL2397	Moderate	low	-0.850	2.954	5.014	97.607
NPRL2398	Good	low	-0.362	3.614	3.963	52.461
NPRL2399	Good	low	1.581	4.415	4.297	68.421
NPRL2400	Good	very low, but possible	-1.010	3.463	5.690	47.605
NPRL2401	Good	low	-0.155	4.936	4.450	35.720
NPRL2402	Good	low	2.331	-2.522	4.230	88.677
NPRL2403	Good	low	0.070	2.805	5.035	68.421
NPRL2404	Good	low	-3.009	-0.660	3.043	97.607
NPRL2405	Good	good	-3.622	-1.989	2.224	94.092
NPRL2406	Moderate	very low, but possible	-0.732	5.471	5.855	59.491
NPRL2407	Moderate	very low, but possible	2.938	3.076	6.329	67.861
NPRL2408	Moderate	low	0.134	2.214	5.676	88.677
NPRL2409	Moderate	very low, but possible	-0.435	3.775	5.856	59.491
NPRL2410	Moderate	very low, but possible	-2.111	0.546	6.542	55.976
NPRL2411	Good	low	-2.028	0.278	3.999	59.491
NPRL2414	Good	good	0.732	-3.893	2.615	67.861
NPRL2415	Good	low	-4.472	1.743	2.466	73.277
NPRL2416	Good	good	-2.013	-1.151	1.663	73.277
NPRL2417	Good	opti mal	-3.415	-2.814	0.224	123.278
NPRL2418	Good	good	-3.489	-3.966	1.465	73.277
NPRL2419	Good	opti mal	-5.309	-1.794	1.182	55.976
NPRL2420	Good	opti mal	-4.244	-0.272	1.259	53.021
NPRL2421	Good	good	-3.330	1.893	2.800	109.557
NPRL2422	Good	good	-3.494	0.174	1.938	118.487
NPRL2423	Very poor	low	-7.644	-4.207	2.363	170.884
NPRL2424	Very poor	very low, but possible	-5.782	-24.728	-1.693	326.082
NPRL2425	Moderate	good	-5.369	4.716	4.656	109.557
NPRL2426	Moderate	very low, but possible	-2.790	4.206	4.901	89.346
NPRL2427	Poor	low	-1.885	3.282	6.151	100.562
NPRL2428	Very poor	good	-5.251	-6.956	1.984	165.469
NPRL2429	Good	good	-5.135	0.672	1.004	81.648
NPRL2430	Good	good	-1.883	3.656	2.399	100.626
NPRL2431	Poor	low	-2.126	4.460	5.786	100.626
NPRL2432	Good	good	-4.949	2.757	2.965	91.696
NPRL2434	Good	low	0.806	0.966	3.294	85.162
NPRL2435	Poor	low	-1.371	3.304	5.318	109.492
NPRL2436	Good	low	-1.420	3.723	4.420	94.092
NPRL2437	Poor	low	-0.913	3.739	5.318	109.492
NPRL2438	Poor	low	-1.111	1.738	5.318	109.492
NPRL2439	Good	low	0.355	2.897	4.094	88.677
NPRL2440	Good	good	0.574	-5.952	2.373	88.677
NPRL2441	Good	low	-4.350	3.222	3.593	82.207
NPRL2442	Good	good	1.074	0.944	2.604	79.811
NPRL2443	Good	low	4.521	3.766	4.472	67.861
NPRL2444	Good	good	-1.560	3.565	2.564	82.766
NPRL2445	Good	good	-2.150	3.642	2.641	79.811
NPRL2446	Good	good	-2.946	4.129	3.130	73.836
NPRL2447	Good	good	-4.342	0.513	2.476	97.671
NPRL2448	Good	low	-3.204	1.389	2.564	94.092
NPRL2449	Good	low	-1.604	4.200	4.348	88.677
NPRL2450	Poor	very low, but possible	0.224	4.660	5.951	88.677
NPRL2452	Good	good	1.437	-1.056	2.609	103.022
NPRL2453	Good	good	-5.487	2.358	2.800	109.557
NPRL2455	Good	low	-0.056	3.924	4.433	69.762
NPRL2456	Very poor	low	-11.794	-57.304	-2.799	448.424
NPRL2457	Very poor	good	-10.749	-4.787	1.560	356.308
NPRL2458	Very poor	low	-11.794	-57.304	-2.343	448.424
NPRL2460	Very poor	very low, but possible	2.575	-0.889	5.600	210.055
NPRL2461	Very poor	very low, but possible	-0.713	6.982	8.807	138.743
NPRL2462	Poor	low	1.651	1.216	5.845	109.492
NPRL2463	Poor	very low, but possible	1.871	0.756	6.070	97.607
NPRL2464	Good	low	2.132	0.075	4.439	85.722
NPRL2465	Poor	low	0.001	-0.127	4.639	130.308
NPRL2466	Good	very low, but possible	-6.534	-3.803	4.540	70.321
NPRL2467	Very poor	Extremely low	-2.356	2.057	7.080	134.492
NPRL2468	Good	low	-3.795	1.789	3.210	114.908

NPRL2469	Good	low	-0.623	-1.985	3.958	67.861
NPRL2470	Good	opti mal	-4.748	-0.792	-0.237	98.948
NPRL2471	Good	good	-3.975	2.556	1.949	64.347
NPRL2472	Good	good	-3.671	4.540	3.460	38.116
NPRL2473	Good	very low, but possible	-4.689	3.097	4.383	77.460
NPRL2474	Good	good	2.217	1.675	3.026	105.978
NPRL2475	Good	good	0.000	0.033	2.942	58.931
NPRL2476	Good	good	-5.170	-0.625	2.272	76.232
NPRL2477	Good	good	-2.782	-0.697	2.681	104.923
NPRL2478	Good	good	-0.760	0.826	2.942	58.931
NPRL2479	Good	low	-0.829	0.599	3.462	109.492
NPRL2480	Good	good	-1.312	-7.779	1.721	109.492
NPRL2481	Good	good	-4.979	-1.832	2.188	47.715
NPRL2482	Good	good	-3.618	-4.556	2.172	56.645
NPRL2484	Very poor	very low, but possible	-1.675	-3.163	4.708	177.354
NPRL2485	Very poor	very low, but possible	-0.814	-2.903	3.783	177.354
NPRL2486	Very poor	very low, but possible	-1.891	-3.813	3.856	177.354
NPRL2487	Very poor	low	-1.623	-4.984	2.116	208.441
NPRL2488	Very poor	good	-3.597	-5.640	-0.088	222.787
NPRL2489	Moderate	low	-5.633	-9.719	-1.396	124.244
NPRL2490	Very poor	Extremely low	-4.132	-10.017	-0.180	165.875
NPRL2491	Poor	very low, but possible	-2.252	-3.773	2.929	142.402
NPRL2492	Very poor	low	-5.481	-10.350	-2.543	165.875
NPRL2493	Very poor	too soluble	-2.298	-5.734	-3.087	138.406
NPRL2494	Good	good	-5.582	-1.445	1.108	107.878
NPRL2495	Very poor	opti mal	-2.608	-3.748	0.403	155.979
NPRL2496	Good	good	-3.902	0.891	1.708	70.321
NPRL2497	Good	good	0.174	0.995	3.214	69.203
NPRL2498	Good	good	-4.409	-1.344	1.715	72.717
NPRL2499	Good	good	0.668	1.292	4.126	69.203
NPRL2500	Poor	very low, but possible	-1.990	1.694	6.793	73.277
NPRL2501	Good	opti mal	-5.743	-7.222	-0.210	114.348
NPRL2502	Poor	good	-2.154	-0.818	4.733	121.665
NPRL2503	Moderate	opti mal	-7.174	-14.564	-0.656	124.057
NPRL2504	Poor	low	0.245	1.559	6.865	69.203
NPRL2505	Good	low	0.765	-0.631	5.384	43.531
NPRL2506	Poor	low	1.458	1.420	6.736	51.902
NPRL2507	Poor	low	0.067	1.832	6.407	69.203
NPRL2508	Very poor	Extremely low	0.774	5.021	7.239	43.531
NPRL2509	Good	very low, but possible	0.818	5.638	5.540	51.902
NPRL2510	Moderate	very low, but possible	0.525	4.825	5.984	51.902
NPRL2511	Good	low	-4.302	-2.066	3.029	80.672
NPRL2512	Good	good	-7.327	-2.918	2.641	118.788
NPRL2513	Good	very low, but possible	-1.245	-8.843	3.962	105.998
NPRL2514	Moderate	very low, but possible	-1.858	-9.751	3.755	109.513
NPRL2515	Good	low	-6.810	-1.509	3.408	86.087
NPRL2516	Good	low	-5.568	0.068	3.667	97.972
NPRL2517	Good	good	-2.947	-1.589	3.147	92.557
NPRL2518	Good	good	-3.447	-0.861	3.288	92.557
NPRL2519	Poor	very low, but possible	-1.536	2.709	6.850	80.672
NPRL2520	Good	good	-5.783	-3.500	2.003	113.373
NPRL2521	Good	low	-5.352	-0.333	3.526	97.972
NPRL2522	Good	low	-1.221	-2.617	3.038	90.578
NPRL2523	Good	opti mal	-6.600	-1.909	1.533	76.791
NPRL2524	Good	low	-2.608	-2.157	3.459	69.762
NPRL2525	Good	low	0.334	-0.394	3.882	76.232
NPRL2526	Good	very low, but possible	-0.513	1.944	4.973	55.417
NPRL2527	Good	very low, but possible	-4.953	0.654	4.811	67.861
NPRL2528	Good	very low, but possible	-1.430	0.264	5.367	58.931
NPRL2529	Good	low	-0.853	-0.160	5.111	62.446
NPRL2530	Good	low	-0.123	-1.641	4.276	79.747
NPRL2531	Good	very low, but possible	-3.622	0.937	5.167	55.976
NPRL2532	Good	very low, but possible	-3.536	-0.957	4.827	67.861
NPRL2533	Good	low	-4.871	-1.263	3.736	88.677
NPRL2534	Moderate	very low, but possible	-1.014	0.789	6.212	41.631
NPRL2535	Good	low	-5.159	-1.600	2.904	109.492
NPRL2536	Good	low	-1.555	-0.145	2.780	97.048
NPRL2537	Good	low	0.334	-0.394	3.882	76.232
NPRL2538	Good	low	0.334	-0.394	3.882	76.232
NPRL2539	Good	very low, but possible	-3.450	0.879	4.828	67.861
NPRL2540	Good	very low, but possible	-3.536	-0.090	4.838	67.861
NPRL2541	Moderate	very low, but possible	-1.274	1.025	5.888	67.861
NPRL2542	Good	good	-4.013	0.840	1.621	116.808
NPRL2543	Moderate	good	-3.661	-0.344	2.906	128.694
NPRL2544	Very poor	good	-3.069	-0.882	2.637	166.810
NPRL2545	Good	good	-3.597	2.277	2.862	111.393

NPRL2546	Good	low	1.276	3.934	4.518	60.832
NPRL2547	Good	low	0.350	2.794	4.870	60.832
NPRL2548	Moderate	opti mal	-11.510	-14.900	-0.495	130.867
NPRL2549	Good	opti mal	-5.979	-3.407	0.984	64.347
NPRL2550	Good	good	-12.977	3.885	2.050	111.609
NPRL2551	Poor	very low, but possible	-3.633	1.383	6.222	146.554
NPRL2552	Moderate	low	-0.124	2.433	5.740	58.931
NPRL2554	Very poor	low	-4.985	-1.356	2.012	180.037
NPRL2555	Very poor	low	-4.690	-2.225	0.679	178.696
NPRL2556	Good	good	-4.978	-5.912	2.568	97.048
NPRL2557	Very poor	opti mal	-3.471	-4.375	-2.302	180.037
NPRL2558	Very poor	good	-6.495	-6.276	-0.599	225.183
NPRL2559	Very poor	good	-2.797	-8.138	-1.426	228.697
NPRL2561	Good	low	-2.608	-2.157	3.459	69.762
NPRL2562	Good	good	-6.463	-3.448	0.857	111.393
NPRL2564	Good	low	-3.710	-2.251	3.257	94.092
NPRL2565	Good	low	-3.933	-0.604	3.243	94.092
NPRL2566	Good	low	0.438	-2.049	4.020	83.262
NPRL2567	Good	very low, but possible	-1.082	1.272	5.364	67.861
NPRL2569	Good	low	-2.079	0.089	3.036	93.533
NPRL2570	Good	low	-0.853	-1.010	5.111	62.446
NPRL2571	Moderate	very low, but possible	-3.104	-2.837	5.146	99.508
NPRL2573	Very poor	good	-8.573	-20.910	-3.053	369.614
NPRL2574	Very poor	good	-7.585	-13.219	0.899	216.030
NPRL2575	Very poor	low	-11.723	-18.488	0.565	292.822
NPRL2576	Very poor	good	-9.240	-11.579	1.021	209.001
NPRL2577	Very poor	good	-10.500	-23.298	-1.584	327.983
NPRL2578	Very poor	good	-10.601	-14.610	-1.582	333.398
NPRL2579	Very poor	good	-10.881	-21.938	-1.205	333.398
NPRL2580	Very poor	good	-11.273	-18.739	-1.314	333.398
NPRL2581	Very poor	opti mal	-9.839	-13.705	-2.548	341.769
NPRL2582	Good	good	-1.429	-0.996	2.056	88.677
NPRL2583	Good	good	0.424	-0.764	3.023	67.861
NPRL2584	Very poor	good	-5.698	-13.306	0.310	168.984
NPRL2585	Moderate	good	-4.553	-11.385	2.378	127.353
NPRL2586	Good	good	-2.308	-4.073	2.579	67.861
NPRL2587	Good	good	-1.492	-2.351	2.330	76.791
NPRL2588	Good	low	-0.587	1.297	3.351	34.601
NPRL2589	Good	good	-5.790	-2.224	1.890	58.931
NPRL2591	Good	good	-2.129	2.272	1.932	73.277
NPRL2592	Good	good	-2.162	2.019	1.604	91.137
NPRL2593	Good	good	-1.834	3.310	2.390	73.277
NPRL2594	Good	low	-0.832	-2.608	2.629	73.277
NPRL2595	Good	low	2.904	3.733	2.851	53.021
NPRL2596	Good	low	-3.582	1.962	3.485	70.321
NPRL2597	Good	low	-0.525	-0.720	3.648	70.321
NPRL2598	Very poor	good	-12.297	-21.376	-0.058	330.156
NPRL2599	Very poor	good	-11.570	-30.625	1.050	270.665
NPRL2600	Very poor	good	-11.570	-26.681	1.050	270.665
NPRL2603	Good	very low, but possible	-1.062	0.395	4.614	76.232
NPRL2604	Very poor	low	-5.296	-16.613	-0.431	237.405
NPRL2605	Very poor	low	-4.672	-18.418	-0.415	228.475
NPRL2606	Good	low	-10.835	-1.297	1.447	100.177
NPRL2607	Very poor	low	-14.812	-16.998	-0.180	230.485
NPRL2608	Very poor	good	-12.483	-39.970	-0.677	233.344
NPRL2609	Good	low	-1.335	4.473	5.332	38.116
NPRL2610	Good	low	-1.099	3.321	4.907	38.116
NPRL2611	Good	good	-1.277	-0.278	1.747	34.601
NPRL2612	Good	low	-4.203	7.270	3.531	61.951
NPRL2613	Good	low	-0.201	2.658	2.237	53.580
NPRL2614	Good	low	-2.177	6.754	3.571	61.951
NPRL2615	Good	good	-0.429	2.753	2.566	65.466
NPRL2616	Good	low	-3.316	6.409	3.145	79.252
NPRL2617	Good	low	-1.245	0.906	4.050	74.396
NPRL2618	Good	low	-2.345	3.232	3.851	74.396
NPRL2619	Good	low	-2.622	2.124	4.412	79.811
NPRL2620	Good	very low, but possible	-0.117	4.038	4.868	53.580
NPRL2621	Good	very low, but possible	-0.510	4.687	5.067	53.580
NPRL2622	Moderate	low	-3.772	1.518	3.503	121.442
NPRL2623	Good	very low, but possible	-0.195	2.654	5.266	53.580
NPRL2624	Good	good	-2.457	-2.707	2.515	89.236
NPRL2625	Poor	opti mal	-2.547	-12.374	-0.849	148.168
NPRL2626	Very poor	good	-5.534	-15.752	0.750	169.543
NPRL2627	Very poor	good	-7.347	-12.311	0.517	199.289
NPRL2628	Very poor	good	-13.096	-18.127	0.935	225.520
NPRL2629	Very poor	good	-7.043	-13.717	-0.640	175.518

NPRL2630	Very poor	low	-10.838	-21.313	0.484	249.290
NPRL2631	Very poor	good	-9.877	-23.322	-2.170	246.335
NPRL2632	Very poor	very low, but possible	-12.466	-20.566	-0.385	314.197
NPRL2633	Moderate	opti mal	-5.848	-19.636	-0.584	121.937
NPRL2634	Good	good	-6.988	-11.973	1.459	127.353
NPRL2635	Very poor	opti mal	-2.655	-14.381	-1.758	160.613
NPRL2636	Poor	opti mal	-8.206	-18.844	-0.410	142.753
NPRL2637	Very poor	opti mal	-7.840	-17.703	-1.124	156.936
NPRL26A27:I2738	Very poor	good	-5.523	-17.604	-0.637	186.284
NPRL2639	Very poor	low	-4.366	-19.449	-2.384	266.591
NPRL2640	Very poor	good	-5.869	-14.983	-0.428	183.329
NPRL2641	Very poor	good	-4.835	-11.559	-1.664	242.820
NPRL2642	Very poor	low	-4.835	-18.279	-1.472	267.151
NPRL2643	Very poor	opti mal	-2.476	-7.140	-0.685	177.354
NPRL2644	Very poor	low	-3.583	-29.136	-2.384	266.591
NPRL2645	Good	good	-2.328	-1.008	2.205	106.537
NPRL2646	Very poor	very low, but possible	-4.493	-18.724	-2.478	305.267
NPRL2647	Very poor	good	-3.958	-14.612	-1.619	245.776
NPRL2648	Very poor	good	-3.567	-21.181	-0.209	189.799
NPRL2649	Very poor	good	-2.524	-20.006	0.444	168.984
NPRL2650	Very poor	low	-3.888	-31.457	-0.415	228.475
NPRL2651	Very poor	good	-8.092	-11.061	0.778	157.517
NPRL2652	Moderate	too soluble	-2.100	-13.546	-0.947	121.937
NPRL2653	Very poor	too soluble	-3.034	-16.993	-3.594	165.469
NPRL2654	Moderate	opti mal	-8.695	-15.642	-0.357	139.238
NPRL2655	Very poor	opti mal	-4.885	-21.980	-1.278	202.244
NPRL2657	Very poor	good	-14.519	-21.302	-1.808	260.680
NPRL2658	Very poor	good	-14.519	-22.855	-1.808	260.680
NPRL2659	Very poor	good	-14.127	-20.956	-1.808	260.680
NPRL2660	Very poor	good	-14.519	-22.855	-1.808	260.680
NPRL2661	Poor	good	-9.587	-15.336	0.602	148.168
NPRL2662	Very poor	good	-2.314	-9.655	-0.396	201.684
NPRL2663	Moderate	opti mal	-4.171	-7.938	-0.112	29.434
NPRL2664	Good	low	0.702	4.305	4.249	34.601
NPRL2665	Good	good	-2.266	-3.967	2.154	71.376
NPRL2666	Good	opti mal	-4.982	-7.269	0.687	92.192
NPRL2667	Good	good	-1.665	-2.694	2.419	79.747
NPRL2668	Good	low	-0.507	1.801	5.435	20.815
NPRL2669	Very poor	very low, but possible	0.542	-6.241	5.307	180.869
NPRL2670	Good	good	0.383	-1.853	3.382	58.931
NPRL2671	Good	good	-0.761	-3.500	2.291	79.747
NPRL2672	Good	good	-1.491	-2.846	1.811	88.677
NPRL2673	Good	good	-0.301	-0.613	2.981	67.861
NPRL2674	Good	good	-2.063	-1.978	1.822	88.677
NPRL2675	Good	opti mal	-2.642	-3.761	2.355	83.262
NPRL2676	Good	good	-2.154	-1.475	1.936	85.162
NPRL2677	Good	low	-1.599	0.855	3.588	38.116
NPRL2678	Good	low	-1.737	6.632	3.573	34.601
NPRL2679	Good	low	-0.906	3.284	3.402	34.601
NPRL2680	Good	low	-0.906	3.284	3.402	34.601
NPRL2681	Good	low	-1.128	-4.549	3.487	41.631
NPRL2682	Good	low	-1.128	-4.549	3.487	41.631
NPRL2683	Good	low	-1.128	-4.549	3.487	41.631
NPRL2684	Good	very low, but possible	-1.750	10.439	4.575	17.300
NPRL2685	Good	low	-1.599	0.855	3.588	38.116
NPRL2686	Good	low	-1.599	0.855	3.588	38.116
NPRL2687	Good	low	-1.599	0.855	3.588	38.116
NPRL2688	Good	low	-0.604	-1.243	3.301	38.116
NPRL2689	Good	very low, but possible	-0.889	3.784	4.380	17.300
NPRL2690	Good	good	-2.983	-3.270	2.621	58.931
NPRL2691	Good	low	-2.905	-2.025	3.967	43.531
NPRL2692	Good	very low, but possible	-1.241	2.432	4.266	17.860
NPRL2693	Good	good	-2.983	-3.270	2.621	58.931
NPRL2694	Good	low	-4.217	-4.330	3.379	69.762
NPRL2695	Good	low	-4.006	-6.039	3.408	78.692
NPRL2696	Good	low	-1.569	-0.453	4.022	53.021
NPRL2697	Good	good	-8.626	-7.745	1.905	123.838
NPRL2698	Poor	low	-8.251	-7.664	3.331	140.084
NPRL2699	Good	low	-0.531	4.101	4.075	53.021
NPRL2700	Good	good	-7.505	5.594	2.073	123.838
NPRL2701	Poor	low	-7.698	5.107	3.499	140.084
NPRL2702	Very poor	Extremely low	-2.340	1.723	7.728	26.230
NPRL2703	Very poor	Extremely low	-1.576	3.906	7.739	26.230
NPRL2704	Very poor	Extremely low	-1.414	5.295	8.451	0.000
NPRL2705	Very poor	Extremely low	-1.113	5.227	8.451	0.000
NPRL2706	Very poor	Extremely low	-2.369	2.787	7.209	52.461

NPRL2707	Good	very low, but possible	-0.366	-0.923	5.503	67.861
NPRL2708	Very poor	Extremely low	-1.024	4.318	8.505	0.000
NPRL2709	Poor	Extremely low	-1.751	0.836	6.951	26.230
NPRL2710	Moderate	Extremely low	-2.975	-1.316	6.645	47.046
NPRL2711	Very poor	Extremely low	-2.877	1.820	7.728	26.230
NPRL2712	Very poor	Extremely low	-1.986	1.077	7.682	26.230
NPRL2713	Very poor	Extremely low	0.132	2.992	7.859	20.815
NPRL2714	Very poor	Extremely low	-2.086	1.345	7.682	26.230
NPRL2715	Moderate	very low, but possible	-1.905	1.532	6.258	41.631
NPRL2716	Very poor	Extremely low	-2.693	3.271	7.728	26.230
NPRL2717	Very poor	Extremely low	-1.506	3.276	7.026	17.300
NPRL2718	Very poor	Extremely low	-1.078	4.306	7.404	17.300
NPRL2719	Very poor	Extremely low	-1.651	1.185	7.306	8.930
NPRL2720	Moderate	Extremely low	-1.857	2.514	6.656	38.116
NPRL2721	Moderate	very low, but possible	-1.753	2.055	6.266	41.631
NPRL2722	Very poor	Extremely low	-2.049	4.620	7.871	17.300
NPRL2723	Very poor	Extremely low	-2.334	2.969	7.417	20.815
NPRL2724	Very poor	Extremely low	-1.113	5.574	8.451	0.000
NPRL2725	Good	low	-2.483	-1.108	4.550	62.446
NPRL2726	Very poor	Extremely low	-0.787	4.200	8.645	0.000
NPRL2728	Very poor	Extremely low	-0.373	4.167	8.645	0.000
NPRL2729	Very poor	Extremely low	-1.506	3.276	7.026	17.300
NPRL2730	Very poor	Extremely low	-2.086	1.606	7.682	26.230
NPRL2731	Very poor	Extremely low	-1.986	3.341	7.932	26.230
NPRL2732	Good	low	0.872	0.922	3.147	55.417
NPRL2733	Very poor	Extremely low	-0.658	2.057	7.443	17.300
NPRL2734	Very poor	Extremely low	-3.075	2.940	8.694	26.230
NPRL2735	Very poor	Extremely low	-1.477	1.940	9.149	26.230
NPRL2736	Very poor	Extremely low	-3.075	2.472	8.238	26.230
NPRL2737	Very poor	Extremely low	-1.831	0.681	7.932	26.230
NPRL2738	Very poor	Extremely low	-1.749	1.269	7.861	8.930
NPRL2739	Very poor	Extremely low	0.314	3.002	7.132	34.601
NPRL2740	Moderate	very low, but possible	-2.819	2.507	6.205	38.116
NPRL2741	Very poor	Extremely low	-1.705	3.603	7.644	17.300
NPRL2742	Poor	Extremely low	-3.057	2.170	6.795	43.531
NPRL2743	Moderate	very low, but possible	-0.999	0.581	6.519	41.631
NPRL2745	Very poor	Extremely low	-0.947	4.678	8.451	0.000
NPRL2746	Very poor	Extremely low	-0.947	4.678	8.451	0.000
NPRL2747	Very poor	Extremely low	-1.259	3.202	7.352	8.930
NPRL2748	Moderate	very low, but possible	-1.813	1.985	5.924	38.116
NPRL2749	Moderate	very low, but possible	-1.813	1.985	5.924	38.116
NPRL2750	Moderate	Extremely low	-1.379	4.592	6.025	34.601
NPRL2751	Very poor	Extremely low	-0.226	4.375	8.220	17.300
NPRL2752	Very poor	Extremely low	-0.596	5.545	9.471	0.000
NPRL2753	Very poor	Extremely low	-0.667	3.163	7.553	20.815
NPRL2754	Very poor	Extremely low	-2.157	2.755	7.932	26.230
NPRL2755	Moderate	Extremely low	-1.643	4.369	6.652	34.601
NPRL2756	Very poor	Extremely low	-0.787	3.768	8.850	0.000
NPRL2757	Poor	Extremely low	-1.015	2.982	6.847	34.601
NPRL2758	Poor	Extremely low	-2.070	2.431	6.931	43.531
NPRL2759	Moderate	very low, but possible	-1.360	1.827	6.646	41.631
NPRL2760	Poor	Extremely low	-2.679	2.195	6.403	69.762
NPRL2761	Very poor	Extremely low	1.806	2.836	8.062	20.815
NPRL2762	Very poor	Extremely low	1.361	4.212	8.981	0.000
NPRL2763	Very poor	Extremely low	2.374	4.113	8.981	0.000
NPRL2764	Very poor	Extremely low	0.765	2.056	7.542	20.815
NPRL2765	Very poor	Extremely low	-1.790	4.289	7.449	17.300
NPRL2766	Moderate	very low, but possible	-1.893	2.856	6.382	41.631
NPRL2767	Very poor	Extremely low	-1.377	3.889	8.645	0.000
NPRL2768	Very poor	Extremely low	-0.219	2.748	8.655	0.000
NPRL2769	Very poor	Extremely low	-2.636	3.940	8.304	26.230
NPRL2770	Moderate	Extremely low	-4.162	2.958	6.452	26.230
NPRL2771	Very poor	Extremely low	-2.922	1.737	7.922	26.230
NPRL2772	Very poor	Extremely low	-2.832	0.821	7.356	26.230
NPRL2773	Very poor	opti mal	-6.183	-5.046	-1.488	167.370
NPRL2774	Good	good	-6.944	-2.365	1.398	116.808
NPRL2775	Good	low	-4.938	0.042	2.651	54.725
NPRL2776	Good	good	-5.558	-3.574	1.600	82.207
NPRL2777	Good	good	-9.212	-3.180	0.643	103.022
NPRL2778	Very poor	good	-8.917	-20.197	-1.659	273.907
NPRL2779	Good	good	-6.911	-2.889	-0.089	107.878
NPRL2780	Very poor	opti mal	-5.958	-7.725	-2.222	207.387
NPRL2782	Good	low	-1.925	1.242	2.756	55.564
NPRL2783	Good	opti mal	0.661	-5.545	-0.266	49.656
NPRL2784	Good	low	-2.298	-0.675	2.165	55.564
NPRL2785	Good	good	-6.513	0.790	1.039	60.917

NPRL2786	Good	low	-2.349	-1.008	2.456	52.547
NPRL2787	Good	low	-6.134	3.478	2.773	46.634
NPRL2788	Good	good	-4.015	-0.498	1.236	63.935
NPRL2789	Good	low	-4.267	1.311	3.188	61.477
NPRL2790	Good	low	-3.162	-0.324	1.949	72.865
NPRL2791	Good	low	-1.622	2.211	3.136	35.246
NPRL2792	Good	low	-1.833	-1.116	2.462	76.379
NPRL2793	Good	good	-3.146	-0.773	1.533	84.750
NPRL2794	Good	good	-5.371	0.024	1.505	60.917
NPRL2795	Good	good	1.991	-3.931	2.181	54.096
NPRL2796	Good	low	-7.817	2.042	3.952	26.316
NPRL2797	Good	low	-9.178	1.148	3.100	30.110
NPRL2798	Moderate	opti mal	-6.119	-5.264	-1.950	106.537
NPRL2799	Good	good	-6.349	-2.257	1.405	82.207
NPRL2800	Good	low	-4.529	-2.908	1.712	78.692
NPRL2801	Good	good	-6.595	-3.961	1.730	52.232
NPRL2802	Good	low	-7.121	-2.945	2.042	43.302
NPRL2803	Good	Extremely low	-0.188	2.195	5.513	64.579
NPRL2804	Moderate	opti mal	-6.119	-5.264	-1.415	106.537
NPRL2805	Good	opti mal	-8.628	-4.375	-0.672	85.722
NPRL2806	Good	very low, but possible	0.159	2.208	4.826	56.361
NPRL2807	Good	very low, but possible	0.159	2.208	4.826	56.361
NPRL2808	Good	low	-3.358	-0.962	3.018	30.110
NPRL2809	Good	good	-6.628	4.963	2.864	38.513
NPRL2810	Very poor	Extremely low	-2.057	4.504	7.712	8.930
NPRL2811	Very poor	Extremely low	-0.920	5.001	7.757	8.930
NPRL2813	Moderate	very low, but possible	-0.925	2.576	5.847	55.417
NPRL2814	Moderate	low	-1.234	-0.558	5.789	62.446
NPRL2815	Moderate	low	-1.234	-0.558	5.789	62.446
NPRL2816	Very poor	very low, but possible	-1.605	2.851	7.059	38.116
NPRL2817	Poor	very low, but possible	-3.974	-0.469	6.547	73.277
NPRL2818	Very poor	very low, but possible	-1.605	2.851	7.059	38.116
NPRL2819	Moderate	very low, but possible	-1.902	0.294	5.890	58.931
NPRL2820	Poor	very low, but possible	-1.404	1.372	6.959	41.631
NPRL2821	Good	very low, but possible	-1.923	-1.079	5.657	50.561
NPRL2822	Poor	very low, but possible	-1.404	1.372	6.959	41.631
NPRL2823	Good	very low, but possible	-2.093	0.563	5.757	47.046
NPRL2824	Moderate	very low, but possible	-6.577	3.053	5.243	85.162
NPRL2825	Good	low	-3.657	-0.050	2.284	123.278
NPRL2826	Good	good	-2.431	-2.495	2.086	124.892
NPRL2827	Good	low	-1.088	0.701	3.770	83.262
NPRL2828	Good	low	-2.280	-3.109	3.681	67.861
NPRL2829	Good	good	-0.356	-1.322	3.296	62.446
NPRL2830	Good	low	-1.099	3.321	4.907	38.116
NPRL2831	Good	low	-3.041	3.996	4.775	38.116
NPRL2832	Good	low	-3.310	-4.494	3.397	92.192
NPRL2833	Very poor	low	-14.853	-15.112	0.708	305.267
NPRL2834	Very poor	very low, but possible	-9.449	-19.662	-1.274	418.834
NPRL2835	Very poor	good	-9.922	-11.096	0.709	160.054
NPRL2836	Very poor	very low, but possible	-11.351	-18.170	-0.586	398.018
NPRL2837	Very poor	low	-10.376	-22.403	-0.067	291.481
NPRL2838	Very poor	very low, but possible	-8.258	-22.750	-1.158	312.296
NPRL2839	Very poor	low	-10.767	-21.261	1.023	270.665
NPRL2840	Very poor	very low, but possible	-9.199	-19.249	-1.420	457.510
NPRL2841	Very poor	very low, but possible	-9.199	-19.249	-1.420	457.510
NPRL2842	Very poor	Extremely low	-9.199	-19.595	-2.510	478.325
NPRL2843	Very poor	very low, but possible	-9.709	-20.118	-0.110	303.366
NPRL2844	Very poor	very low, but possible	-7.977	-20.822	-1.412	312.296
NPRL2845	Very poor	Extremely low	-9.279	-18.468	-0.791	401.533
NPRL2846	Very poor	low	-9.717	-18.221	-0.553	252.805
NPRL2847	Very poor	good	-8.394	-19.687	1.346	193.983
NPRL2848	Very poor	low	-14.120	-13.389	0.192	281.991
NPRL2849	Very poor	low	-9.997	-24.892	0.713	231.990
NPRL2850	Very poor	too soluble	-20.476	-44.441	2.069	361.867
NPRL2851	Very poor	Extremely low	-13.427	-20.318	-1.855	462.365
NPRL2852	Very poor	Extremely low	-9.656	-20.229	-2.108	418.834
NPRL2853	Very poor	Extremely low	-11.937	-23.127	-3.218	492.111
NPRL2854	Very poor	Extremely low	-11.937	-19.397	-2.708	471.296
NPRL2856	Good	low	-2.080	-2.956	4.488	71.376
NPRL2858	Good	very low, but possible	-1.923	-1.079	5.657	50.561
NPRL2859	Good	very low, but possible	-2.093	0.563	5.757	47.046
NPRL2860	Moderate	opti mal	-12.244	-3.955	-0.641	120.323
NPRL2861	Very poor	good	-8.893	-6.287	-0.854	150.069
NPRL2862	Moderate	very low, but possible	-2.047	0.606	5.996	58.931
NPRL2863	Good	low	-2.903	-0.501	3.093	67.861
NPRL2864	Good	low	-1.099	3.321	4.907	38.116

NPRL2865	Very poor	opti mal	-13.295	-11.029	-1.138	241.988
NPRL2866	Good	low	-0.613	1.835	4.806	26.230
NPRL2867	Good	opti mal	-3.183	-0.880	-0.927	103.804
NPRL2868	Very poor	good	-3.888	-16.296	-3.946	340.650
NPRL2869	Very poor	good	-3.888	-16.296	-3.946	340.650
NPRL2870	Very poor	good	-5.992	-15.413	-0.411	174.399
NPRL2871	Very poor	good	-5.222	-17.946	-0.637	186.284
NPRL2872	Good	good	-5.898	-1.358	1.518	94.092
NPRL2873	Very poor	good	-4.675	-18.097	0.621	189.799
NPRL2875	Very poor	opti mal	-4.321	-15.412	-1.464	182.770
NPRL2877	Very poor	good	-4.810	-20.188	-1.240	251.191
NPRL2878	Very poor	low	-8.933	-24.859	-0.176	252.805
NPRL2879	Very poor	low	-9.717	-17.565	0.335	231.990
NPRL2880	Very poor	low	-9.328	-12.740	0.401	282.551
NPRL2881	Very poor	low	-5.290	-19.921	2.873	163.568
NPRL2882	Very poor	low	-9.134	-13.695	1.887	284.451
NPRL2883	Very poor	low	-10.281	-11.894	1.424	233.331
NPRL2884	Very poor	low	-7.552	-11.322	3.123	224.960
NPRL2885	Very poor	low	-11.442	-12.713	0.187	292.822
NPRL2886	Very poor	low	-10.659	-13.976	0.187	292.822
NPRL2887	Very poor	low	-7.078	-11.361	2.806	202.244
NPRL2888	Very poor	low	-9.363	-17.255	0.506	299.851
NPRL2889	Very poor	Extremely low	-7.162	-18.491	-2.435	443.164
NPRL2890	Very poor	low	-8.229	-16.494	1.637	223.059
NPRL2891	Very poor	low	-8.714	-23.766	0.508	231.990
NPRL2892	Very poor	very low, but possible	-9.989	-25.582	0.267	303.366
NPRL2893	Very poor	low	-6.878	-18.759	2.295	223.059
NPRL2894	Very poor	low	-9.709	-18.363	1.059	282.551
NPRL2895	Very poor	very low, but possible	-12.116	-26.731	0.190	308.782
NPRL2896	Very poor	low	-12.360	-21.718	1.092	237.405
NPRL2897	Very poor	opti mal	-22.995	-29.902	3.950	207.164
NPRL2898	Very poor	low	-8.931	-25.462	1.107	211.174
NPRL2899	Very poor	low	-9.783	-18.758	1.164	223.059
NPRL2900	Very poor	low	-9.623	-22.502	0.451	270.665
NPRL2901	Very poor	low	-8.931	-25.462	1.107	211.174
NPRL2902	Very poor	low	-9.419	-20.581	0.758	282.551
NPRL2904	Good	low	-1.256	-1.376	3.946	29.745
NPRL2905	Very poor	opti mal	-2.996	-15.714	-1.937	153.583
NPRL2906	Very poor	good	-10.855	-13.649	1.201	422.571
NPRL2907	Moderate	very low, but possible	-2.674	0.839	5.970	58.931
NPRL2908	Very poor	good	-10.236	-19.481	1.123	240.360
NPRL2909	Very poor	good	-10.236	-19.481	1.123	240.360
NPRL2910	Very poor	good	-10.438	-19.909	0.592	299.851
NPRL2911	Very poor	good	-10.955	-18.485	0.492	303.366
NPRL2912	Good	good	-5.036	-1.399	0.961	119.764
NPRL2913	Poor	good	-5.580	-1.597	0.531	145.995
NPRL2914	Good	low	-2.753	12.106	3.208	70.881
NPRL2915	Good	very low, but possible	-0.912	1.947	4.106	72.717
NPRL2916	Good	good	-2.431	-2.495	2.086	124.892
NPRL2917	Good	very low, but possible	-3.470	2.190	4.765	55.976
NPRL2918	Moderate	Extremely low	0.085	1.365	6.195	29.745
NPRL2919	Good	low	-1.047	-0.675	5.156	62.446
NPRL2920	Moderate	Extremely low	-1.857	2.514	6.656	38.116
NPRL2921	Good	low	-0.334	-1.696	4.076	83.262
NPRL2922	Very poor	Extremely low	-0.825	2.175	7.859	20.815
NPRL2923	Very poor	Extremely low	-0.243	2.142	7.530	32.138
NPRL2924	Moderate	very low, but possible	-1.135	-0.069	5.705	67.861
NPRL2925	Very poor	Extremely low	-1.940	-0.074	7.865	26.230
NPRL2926	Very poor	Extremely low	-1.940	-0.074	7.865	26.230
NPRL2928	Poor	very low, but possible	-3.029	0.642	6.248	78.692
NPRL2929	Good	good	-2.312	-1.300	3.177	104.077
NPRL2930	Very poor	low	-4.859	-0.316	4.361	157.385
NPRL2933	Moderate	very low, but possible	-3.632	1.709	6.492	58.931
NPRL2937	Very poor	good	-11.527	-41.471	5.548	252.083
NPRL2938	Good	good	-4.555	1.966	2.789	26.230
NPRL2939	Good	very low, but possible	-4.074	0.302	4.531	38.785
NPRL2940	Poor	low	0.076	4.552	4.519	116.808
NPRL2943	Good	low	-1.036	-0.401	4.290	76.791
NPRL2944	Good	opti mal	-2.387	-4.504	-0.319	76.791
NPRL2945	Good	good	-6.561	-2.754	0.954	43.531
NPRL2946	Very poor	opti mal	-4.923	-15.649	-2.403	204.363
NPRL2947	Good	good	-1.635	-2.503	2.871	76.791
NPRL2948	Good	opti mal	-5.441	-2.336	-0.031	43.531
NPRL2949	Very poor	opti mal	-2.682	-9.605	-0.712	156.539
NPRL2950	Moderate	very low, but possible	-0.938	0.709	5.570	67.861
NPRL2951	Good	low	-3.569	-0.672	4.923	58.976

NPRL2952	Very poor	good	-11.392	-10.485	-0.559	162.623
NPRL2953	Good	good	-6.677	1.983	2.136	35.160
NPRL2954	Very poor	good	-3.309	-0.432	1.696	163.296
NPRL2955	Very poor	good	-6.634	-20.967	-1.406	226.861
NPRL2956	Good	good	-1.395	0.347	2.125	69.762
NPRL2957	Moderate	very low, but possible	-1.735	3.177	6.011	58.931
NPRL2958	Moderate	low	-1.067	2.324	5.911	62.446
NPRL2959	Very poor	very low, but possible	-5.983	-19.708	-2.720	326.082
NPRL2961	Good	good	-4.872	0.982	2.891	50.764
NPRL2962	Very poor	opti mal	-4.992	-22.164	-2.363	162.514
NPRL2963	Good	low	-0.815	6.448	3.221	52.954
NPRL2964	Good	good	-2.612	-0.462	2.330	50.605
NPRL2965	Moderate	opti mal	-7.660	-13.471	-0.602	118.422
NPRL2966	Poor	too soluble	-6.871	-14.728	-1.932	144.872
NPRL2968	Good	good	-7.631	-1.232	1.953	71.579
NPRL2969	Very poor	low	-10.136	-16.823	0.665	279.036
NPRL2970	Good	very low, but possible	-3.130	1.006	5.395	58.931
NPRL2971	Poor	too soluble	-4.754	-16.368	-1.663	135.723
NPRL2972	Good	good	-8.966	-20.271	1.818	95.017
NPRL2973	Good	good	-5.495	-2.920	1.896	38.116
NPRL2974	Very poor	Extremely low	-2.416	-5.304	7.218	60.285
NPRL2975	Very poor	too soluble	-1.258	-9.829	-5.975	64.508
NPRL2976	Very poor	too soluble	-1.978	-9.846	-4.410	64.508
NPRL2977	Very poor	too soluble	-2.811	-13.098	-5.092	83.180
NPRL2978	Very poor	too soluble	-1.965	-12.122	-5.334	103.996
NPRL2979	Moderate	too soluble	-6.204	-9.928	-1.273	60.222
NPRL2980	Good	opti mal	-4.820	-6.330	0.971	60.222
NPRL2981	Very poor	too soluble	-3.743	-13.264	-4.522	77.420
NPRL2983	Very poor	Extremely low	-0.274	1.596	7.486	20.815
NPRL2984	Very poor	Extremely low	-0.842	1.759	7.498	20.815
NPRL2985	Moderate	very low, but possible	-1.428	0.495	6.212	41.631
NPRL2988	Good	low	-2.320	1.177	4.905	79.747
NPRL2990	Very poor	good	-3.721	-10.610	-0.492	186.284
NPRL2992	Very poor	opti mal	-3.614	-11.265	-2.105	85.411
NPRL2993	Very poor	opti mal	-4.020	-9.182	-2.311	82.207
NPRL2994	Good	good	-2.895	-14.012	0.250	56.225
NPRL2995	Good	opti mal	-2.965	-2.534	-0.848	62.695
NPRL2996	Good	good	-4.307	0.119	0.838	59.888
NPRL2997	Good	good	-0.052	-6.293	1.752	42.028
NPRL2998	Good	good	-2.876	-0.010	1.813	50.958
NPRL2999	Good	opti mal	-3.615	-4.627	-1.081	71.625
NPRL3000	Good	low	-3.129	-1.439	3.012	74.233
NPRL3001	Good	good	-0.713	-16.064	1.674	59.328
NPRL3002	Good	low	-3.572	-8.560	2.043	55.665
NPRL3003	Good	very low, but possible	-1.735	-5.419	4.389	23.857
NPRL3004	Good	good	-3.505	-18.774	1.793	59.491
NPRL3005	Very poor	Extremely low	-2.416	-5.304	7.218	60.285
NPRL3006	Very poor	Extremely low	-1.545	-3.567	7.218	60.285
NPRL3007	Very poor	very low, but possible	-6.012	-5.624	7.246	72.170
NPRL3009	Good	good	-6.647	4.073	2.933	47.971
NPRL3010	Moderate	low	-6.637	6.130	6.138	47.971
NPRL3011	Good	low	-2.959	3.234	3.309	38.513
NPRL3012	Good	low	0.617	2.318	3.282	26.230
NPRL3013	Good	good	-2.197	1.063	2.276	47.046
NPRL3014	Very poor	good	-10.482	-6.223	7.003	157.098
NPRL3015	Good	opti mal	-7.517	-3.007	0.934	67.861
NPRL3016	Very poor	good	-4.399	-12.082	-1.324	245.776
NPRL3017	Very poor	good	-4.369	-13.323	-0.465	186.284
NPRL3018	Poor	opti mal	-8.201	-21.400	-0.727	144.653
NPRL3019	Good	good	-2.841	-1.401	1.034	64.347
NPRL3020	Good	good	-6.907	-2.138	1.062	67.861
NPRL3021	Poor	opti mal	-7.073	-20.014	-0.813	148.168
NPRL3022	Poor	opti mal	-8.580	-19.504	-0.685	148.168
NPRL3023	Poor	opti mal	-8.580	-19.504	-0.685	148.168
NPRL3024	Good	low	-6.374	4.786	5.226	47.971
NPRL3025	Good	low	-8.212	5.693	4.245	38.513
NPRL3026	Very poor	low	-0.731	1.095	7.607	30.111
NPRL3027	Good	low	-7.001	4.575	4.689	38.513
NPRL3029	Very poor	Extremely low	-10.119	-25.269	-1.263	329.597
NPRL3030	Good	good	-5.664	-1.060	1.451	105.978
NPRL3031	Poor	very low, but possible	-4.241	2.717	5.483	116.808
NPRL3033	Good	low	-3.321	1.878	3.930	61.951
NPRL3034	Good	low	-3.670	0.968	3.502	87.622
NPRL3035	Good	low	-7.185	-2.131	2.335	96.552
NPRL3036	Very poor	good	-8.355	-19.535	-0.549	157.098
NPRL3037	Moderate	good	-9.552	-19.014	0.283	136.283

NPRL3038	Very poor	opti mal	-7.831	-16.753	-0.484	168.984
NPRL3039	Good	good	-5.962	0.092	1.693	85.162
NPRL3040	Good	good	-1.453	0.107	2.330	47.046
NPRL3041	Good	low	-0.444	3.276	4.022	26.230
NPRL3043	Good	good	-1.282	-5.609	2.629	97.607
NPRL3045	Good	low	-0.785	-1.262	4.279	83.262
NPRL3046	Good	low	-0.853	-1.010	5.111	62.446
NPRL3047	Good	low	-2.363	1.111	4.782	67.861
NPRL3048	Good	very low, but possible	-4.883	-0.898	5.161	73.277
NPRL3049	Moderate	low	-4.120	-1.332	3.265	121.378
NPRL3050	Very poor	too soluble	-5.111	-20.721	-2.916	151.683
NPRL3051	Very poor	opti mal	-2.102	-18.419	-2.381	157.098
NPRL3052	Poor	opti mal	-4.681	-19.724	-1.523	136.283
NPRL3053	Very poor	too soluble	-5.010	-20.510	-3.581	160.613
NPRL3054	Good	low	-1.456	0.556	3.719	20.815
NPRL3055	Good	good	-8.447	-1.865	1.388	103.022
NPRL3056	Moderate	Extremely low	-2.477	2.148	6.558	38.116
NPRL3057	Poor	Extremely low	-3.260	1.561	6.937	43.531
NPRL3058	Very poor	low	-9.709	-19.569	1.059	282.551
NPRL3061	Very poor	low	-10.659	-17.891	-0.323	313.638
NPRL3062	Very poor	Extremely low	-7.162	-21.403	-2.435	443.164
NPRL3063	Very poor	low	-8.292	-21.106	2.027	308.782
NPRL3064	Very poor	low	-7.228	-18.675	2.105	389.088
NPRL3065	Very poor	Extremely low	-8.836	-20.652	-2.415	404.488
NPRL3066	Very poor	Extremely low	-7.057	-24.405	-2.945	463.980
NPRL3067	Very poor	low	-7.496	-20.942	-2.483	423.690
NPRL3069	Very poor	low	-8.544	-21.797	1.126	243.875
NPRL3070	Very poor	low	-8.510	-23.821	2.014	223.059
NPRL3071	Very poor	low	-6.878	-20.568	2.295	223.059
NPRL3072	Very poor	good	-5.290	-19.921	2.873	163.568
NPRL3073	Moderate	good	-4.503	-1.914	0.585	131.649
NPRL3074	Very poor	good	-6.058	-7.321	-1.007	218.985
NPRL3075	Very poor	very low, but possible	-5.912	-15.941	-1.221	324.182
NPRL3076	Very poor	good	-9.490	-20.369	-4.235	400.701
NPRL3077	Very poor	very low, but possible	-6.783	-23.692	-2.724	420.175
NPRL3078	Very poor	very low, but possible	-6.783	-23.692	-2.724	420.175
NPRL3079	Very poor	very low, but possible	-6.783	-22.885	-2.980	423.690
NPRL3081	Very poor	good	-9.351	-5.496	0.643	209.001
NPRL3082	Very poor	low	-11.834	-13.445	0.187	292.822
NPRL3083	Very poor	low	-15.103	-16.818	-3.485	548.088
NPRL3084	Very poor	good	-13.640	-17.207	-2.077	488.596
NPRL3085	Very poor	low	-14.316	-17.762	-3.444	574.318
NPRL3086	Very poor	Extremely low	-9.798	-22.300	-5.430	533.742
NPRL3087	Good	low	-5.497	-3.982	3.393	103.022
NPRL3088	Good	low	-1.623	-2.364	2.853	92.192
NPRL3089	Moderate	good	-10.816	0.935	2.340	127.353
NPRL3090	Good	good	-6.715	-6.261	2.085	88.677
NPRL3092	Good	low	0.284	-0.084	5.122	62.446
NPRL3093	Moderate	good	-3.000	-2.139	2.345	124.892
NPRL3094	Good	good	-2.431	-2.495	2.086	124.892
NPRL3095	Good	good	-2.312	-1.300	3.177	104.077
NPRL3096	Good	low	-0.698	-2.975	4.009	83.262
NPRL3098	Very poor	very low, but possible	-11.176	-23.370	-0.814	333.112
NPRL3099	Very poor	low	-9.997	-24.892	0.713	231.990
NPRL3100	Poor	very low, but possible	-0.527	2.371	6.950	41.631
NPRL3101	Very poor	Extremely low	0.094	3.206	8.119	20.815
NPRL3102	Good	very low, but possible	1.252	3.726	5.578	55.417
NPRL3103	Good	too soluble	-5.668	-16.908	-0.906	101.122
NPRL3104	Moderate	opti mal	-5.907	-20.338	-0.443	121.937
NPRL3105	Poor	opti mal	-7.756	-18.132	-0.514	148.168
NPRL3106	Good	good	-4.830	-4.100	2.170	56.645
NPRL3109	Very poor	opti mal	-5.282	-18.065	-1.234	153.583
NPRL3111	Good	good	-6.645	-2.651	0.938	52.461
NPRL3112	Moderate	very low, but possible	-1.671	2.421	5.048	87.622
NPRL3113	Good	low	-1.799	1.739	2.995	38.785
NPRL3114	Very poor	good	-4.169	-17.903	-1.676	237.964
NPRL3115	Good	low	-6.821	0.406	4.756	73.946
NPRL3116	Very poor	low	1.730	-4.149	3.599	177.354
NPRL3117	Very poor	low	6.195	-4.226	3.883	153.024
NPRL3118	Poor	low	-7.603	-9.795	5.225	125.176
NPRL3119	Very poor	good	-8.329	-19.494	-0.620	166.028
NPRL3120	Good	low	-4.424	0.791	3.538	61.391
NPRL3121	Good	low	-0.083	1.916	3.723	44.091
NPRL3122	Good	very low, but possible	-2.681	-2.995	5.101	56.645
NPRL3123	Good	good	-5.155	-2.143	1.575	73.836
NPRL3124	Good	good	-1.837	1.169	2.478	53.580

NPRL3125	Good	low	-5.038	0.699	3.783	61.391
NPRL3126	Good	good	-2.394	1.089	2.765	103.022
NPRL3127	Good	low	-2.469	1.639	3.955	61.391
NPRL3128	Good	good	-7.001	-10.577	2.127	77.460
NPRL3129	Very poor	good	-7.632	-16.907	1.045	171.444
NPRL3130	Very poor	good	-4.179	-18.580	-1.709	255.824
NPRL3131	Moderate	good	-8.514	-15.099	1.160	142.753
NPRL3132	Good	good	-6.308	-3.480	1.961	59.600
NPRL3133	Good	good	-3.243	1.030	2.692	55.976
NPRL3134	Good	low	-1.795	3.581	5.175	35.160
NPRL3135	Good	good	-9.387	-4.664	2.082	103.691
NPRL3136	Good	low	-2.182	-1.009	4.618	61.391
NPRL3137	Poor	very low, but possible	-3.381	0.392	5.392	108.438
NPRL3138	Very poor	opti mal	-11.590	-7.388	1.346	184.175
NPRL3139	Good	good	-5.148	-0.547	1.899	26.230
NPRL3140	Good	low	-3.182	0.277	3.955	61.391
NPRL3141	Good	good	-3.519	-1.032	2.130	52.461
NPRL3142	Good	good	-5.391	-2.196	2.029	55.976
NPRL3143	Good	low	-2.970	-2.581	3.427	59.600
NPRL3144	Good	opti mal	-6.810	-5.812	0.712	64.347
NPRL3145	Good	low	-3.539	0.907	3.500	79.252
NPRL3146	Very poor	opti mal	-10.705	-7.637	2.463	201.476
NPRL3147	Very poor	opti mal	-9.464	-8.656	2.562	193.105
NPRL3148	Poor	opti mal	-5.004	-17.938	-1.218	144.653
NPRL3149	Very poor	opti mal	-9.869	-9.164	1.012	175.245
NPRL3150	Moderate	opti mal	-6.728	-16.375	-0.289	136.283
NPRL3151	Very poor	good	-9.041	-8.822	1.259	150.069
NPRL3152	Good	good	-5.108	-0.289	1.597	87.063
NPRL3153	Poor	opti mal	-7.457	-18.270	-0.305	145.213
NPRL3154	Good	good	-4.323	-1.436	1.955	64.906
NPRL3155	Good	good	-4.323	-1.436	1.955	64.906
NPRL3156	Good	good	-4.323	-1.436	1.955	64.906
NPRL3157	Good	good	-4.323	-1.436	1.955	64.906
NPRL3158	Good	low	-4.527	-1.875	2.456	43.531
NPRL3159	Good	very low, but possible	-2.998	-6.123	5.479	47.715
NPRL3160	Good	good	-7.091	-2.380	2.127	77.460
NPRL3162	Good	good	-1.429	1.199	2.777	52.461
NPRL3163	Good	low	-3.870	-8.457	2.898	65.016
NPRL3164	Good	good	-7.510	-2.117	1.198	76.791
NPRL3165	Good	good	-7.510	-2.117	1.198	76.791
NPRL3166	Good	good	-7.510	-2.117	1.198	76.791
NPRL3169	Good	good	-4.863	-1.254	3.107	76.791
NPRL3170	Good	low	-1.775	1.297	3.455	70.321
NPRL3171	Good	good	-6.494	-1.399	1.671	76.791
NPRL3172	Good	good	-5.345	-0.572	2.386	26.230
NPRL3173	Good	good	-5.858	-2.311	2.723	77.460
NPRL3174	Good	low	-4.745	-0.117	4.377	68.530
NPRL3175	Good	low	-5.564	1.241	3.492	38.785
NPRL3176	Moderate	very low, but possible	-2.525	1.851	5.048	87.622
NPRL3177	Good	opti mal	-6.777	-3.422	0.743	85.162
NPRL3179	Moderate	opti mal	-6.728	-16.284	-0.289	136.283
NPRL3181	Good	good	-6.218	-9.254	1.961	59.600
NPRL3182	Good	opti mal	-5.800	-1.657	0.840	97.607
NPRL3183	Very poor	good	-1.750	-11.587	-1.910	244.657
NPRL3184	Very poor	good	-7.453	-13.232	1.018	183.329
NPRL3185	Very poor	good	-7.453	-13.309	1.474	183.329
NPRL3186	Good	low	-5.402	-1.899	3.502	87.622
NPRL3187	Good	low	-6.101	0.911	3.783	61.391
NPRL3188	Good	good	-2.394	0.509	2.765	103.022
NPRL3189	Very poor	good	-8.329	-19.494	-0.620	166.028
NPRL3190	Good	low	-3.195	2.003	3.955	61.391
NPRL3191	Good	low	-4.562	0.007	3.502	87.622
NPRL3192	Good	low	-4.035	0.097	3.955	61.391
NPRL3193	Good	good	-5.393	0.220	2.739	68.530
NPRL3194	Good	good	-9.383	-2.407	2.098	94.761
NPRL3195	Good	good	0.456	3.189	3.293	58.931
NPRL3196	Poor	low	-4.923	3.014	5.611	100.562
NPRL3197	Good	good	-5.389	1.360	4.010	88.677
NPRL3198	Good	good	-6.655	1.927	4.058	97.607
NPRL3199	Poor	low	-3.238	6.469	6.884	67.861
NPRL3200	Good	low	-6.035	-0.142	3.573	76.791
NPRL3202	Good	low	-10.803	-3.678	1.347	103.691
NPRL3203	Good	very low, but possible	-3.457	-1.029	3.971	82.316
NPRL3204	Moderate	good	-8.388	-2.063	-0.625	117.477
NPRL3205	Moderate	good	-7.235	-0.135	-1.126	122.224
NPRL3206	Good	good	-6.295	-0.919	1.398	104.364

NPRL3207	Moderate	good	-8.506	-6.502	-0.715	115.130
NPRL3208	Good	low	-6.815	-0.297	2.715	91.247
NPRL3209	Good	good	-9.266	-1.555	0.658	120.433
NPRL3210	Good	good	-8.473	0.130	0.142	113.294
NPRL3211	Good	low	-5.812	-0.718	1.842	104.923
NPRL3212	Good	low	-10.202	-1.579	2.151	117.477
NPRL3213	Good	low	-2.060	-0.935	3.400	39.072
NPRL3214	Moderate	very low, but possible	-1.294	2.467	6.451	41.631
NPRL3215	Good	low	-2.059	0.275	3.136	29.745
NPRL3217	Good	good	-1.570	-3.186	2.848	83.262
NPRL3218	Good	good	-5.037	-0.020	0.708	81.648
NPRL3220	Good	good	-1.826	-2.158	2.767	90.578
NPRL3222	Very poor	low	0.192	-15.447	-1.731	252.805
NPRL3223	Very poor	good	-3.928	-15.854	-0.761	186.284
NPRL3224	Very poor	low	-11.421	-21.338	0.190	305.826
NPRL3225	Very poor	Extremely low	0.129	4.246	7.403	20.815
NPRL3226	Good	low	-1.133	1.450	5.361	62.446
NPRL3227	Poor	very low, but possible	0.361	1.284	6.931	41.631
NPRL3228	Moderate	very low, but possible	-1.294	2.467	6.451	41.631
NPRL3229	Very poor	Extremely low	-1.749	2.372	7.507	8.930
NPRL3231	Good	low	0.418	0.660	3.588	67.861
NPRL3232	Very poor	low	-9.692	-20.123	0.250	290.921
NPRL3233	Very poor	good	0.233	-9.404	-0.223	201.684
NPRL3234	Good	good	-6.284	-5.045	2.489	121.378
NPRL3235	Good	good	-7.293	-11.041	1.716	118.422
NPRL3236	Good	good	-1.383	1.582	3.046	38.116
NPRL3237	Good	opti mal	-3.569	-0.063	0.989	79.747
NPRL3238	Moderate	opti mal	-7.474	-13.366	0.332	139.238
NPRL3241	Very poor	Extremely low	-3.479	1.339	8.303	26.790
NPRL3242	Moderate	very low, but possible	-0.460	1.540	6.312	41.631
NPRL3245	Very poor	Extremely low	-1.111	4.379	7.782	26.230
NPRL3246	Very poor	Extremely low	-2.379	5.314	7.594	38.116
NPRL3247	Very poor	Extremely low	-1.079	2.592	7.403	20.815
NPRL3251	Very poor	good	-9.811	-12.281	0.292	160.054
NPRL3252	Good	low	1.040	0.998	4.672	20.815
NPRL3253	Good	good	-4.807	-2.257	1.687	87.622
NPRL3254	Moderate	good	-9.075	-8.992	1.294	130.867
NPRL3256	Very poor	Extremely low	-0.124	3.742	7.287	8.930
NPRL3258	Very poor	Extremely low	-1.490	4.115	7.503	17.300
NPRL3259	Very poor	Extremely low	0.449	1.550	7.404	17.300
NPRL3261	Very poor	Extremely low	-0.929	2.111	7.404	17.300
NPRL3262	Good	low	-4.419	1.813	3.013	114.348
NPRL3263	Good	good	-4.343	3.803	4.043	94.092
NPRL3264	Good	good	-2.628	1.270	4.075	76.232
NPRL3265	Good	good	-3.883	1.165	4.059	85.162
NPRL3266	Good	opti mal	-8.421	-0.216	0.437	43.531
NPRL3267	Good	good	-4.449	2.053	2.485	35.160
NPRL3268	Moderate	good	-4.474	-3.310	1.653	138.679
NPRL3269	Poor	good	-4.908	-6.249	2.312	140.579
NPRL3270	Very poor	good	-10.298	-20.538	0.566	220.886
NPRL3271	Very poor	good	-9.800	-20.110	0.609	220.886
NPRL3272	Very poor	good	-8.087	-18.351	0.230	215.471
NPRL3274	Poor	Extremely low	-1.941	0.774	6.848	43.531
NPRL3275	Good	very low, but possible	-2.057	2.043	5.352	60.832
NPRL3276	Very poor	Extremely low	-0.756	2.917	7.312	34.601
NPRL3279	Very poor	Extremely low	-0.910	1.192	7.303	20.815
NPRL3280	Very poor	Extremely low	-1.310	1.416	7.782	26.230
NPRL3283	Good	low	-0.802	0.808	3.871	76.232
NPRL3284	Good	low	-5.078	-0.848	3.860	88.677
NPRL3285	Good	low	-3.976	-0.164	2.910	82.766
NPRL3286	Good	low	-5.880	-2.110	1.793	79.252
NPRL3288	Very poor	low	-3.988	-13.898	-1.636	254.706
NPRL3291	Good	good	-3.010	0.784	1.888	61.391
NPRL3292	Poor	good	-6.423	-14.117	0.662	145.213
NPRL3295	Good	good	-2.748	0.220	2.601	70.321
NPRL3296	Good	good	-1.102	-4.335	1.437	85.162
NPRL3297	Very poor	good	-7.642	-10.629	1.814	154.143
NPRL3298	Good	low	-3.013	3.348	3.743	73.836
NPRL3299	Good	low	-0.201	2.658	2.237	53.580
NPRL3302	Poor	Extremely low	-2.407	-7.401	6.992	72.170
NPRL3303	Poor	Extremely low	-2.254	-3.387	6.981	51.355
NPRL3305	Very poor	too soluble	-7.699	-21.410	-3.523	177.914
NPRL3306	Moderate	good	-7.950	-6.138	1.137	142.193
NPRL3307	Poor	good	-9.563	-6.889	1.476	147.609
NPRL3308	Very poor	opti mal	-7.278	-7.304	0.278	163.009
NPRL3309	Poor	low	-7.904	-9.883	6.337	101.122

NPRL3310	Good	opti mal	-9.570	-11.364	-0.054	101.122
NPRL3311	Good	opti mal	-11.666	-12.348	-0.070	110.052
NPRL3312	Good	low	-1.434	-0.569	4.446	85.162
NPRL3313	Good	low	-1.434	-0.569	4.446	85.162
NPRL3315	Very poor	opti mal	-1.363	-17.581	-2.066	157.098
NPRL3316	Very poor	opti mal	-3.617	-16.768	-2.975	177.914
NPRL3317	Very poor	opti mal	-6.576	-21.246	-1.779	186.844
NPRL3319	Very poor	opti mal	0.012	-16.829	-2.280	166.028
NPRL3321	Very poor	too soluble	-2.254	-17.999	-3.100	177.914
NPRL3323	Good	good	-8.209	-3.085	1.445	43.531
NPRL3324	Good	opti mal	-7.034	-1.924	0.516	51.902
NPRL3325	Very poor	low	-3.567	-21.885	-1.619	245.776
NPRL3328	Very poor	low	-3.359	-21.636	-1.861	266.591
NPRL3332	Very poor	opti mal	-0.868	-7.031	-1.810	164.910
NPRL3333	Poor	good	-4.164	-17.467	0.396	148.168
NPRL3334	Very poor	low	-3.660	-15.790	-2.706	326.864
NPRL3335	Good	good	-3.891	2.260	1.932	73.277
NPRL3336	Good	good	-5.220	1.715	1.707	85.162
NPRL3337	Good	low	-0.753	0.755	5.348	20.815
NPRL3338	Very poor	opti mal	-3.971	-15.952	-1.679	181.428
NPRL3339	Good	low	-1.332	2.172	2.559	52.461
NPRL3340	Good	low	-32.823	-7.472	3.139	53.021
NPRL3341	Good	good	-1.160	2.255	2.468	34.601
NPRL3342	Good	low	-0.236	2.589	3.761	43.531
NPRL3343	Good	very low, but possible	-2.522	3.565	4.084	47.155
NPRL3344	Good	low	-0.763	5.391	3.184	43.531
NPRL3346	Good	low	-0.688	3.426	3.655	55.976
NPRL3347	Good	good	0.820	-1.303	2.446	67.861
NPRL3348	Good	good	-4.831	-2.037	1.368	88.677
NPRL3349	Good	good	-2.071	-1.544	1.568	67.861
NPRL3350	Good	good	-0.979	3.454	2.861	64.906
NPRL3352	Good	low	-0.515	5.321	3.913	26.230
NPRL3353	Good	low	-0.618	4.927	3.897	35.160
NPRL3354	Good	low	-0.567	2.603	3.881	44.091
NPRL3355	Good	low	0.622	3.164	3.881	44.091
NPRL3356	Good	low	-0.618	0.859	3.671	47.046
NPRL3357	Moderate	low	-5.699	-12.687	2.229	130.673
NPRL3359	Good	low	-5.492	-22.479	2.710	83.132
NPRL3360	Moderate	low	-3.494	-11.868	4.243	103.947
NPRL3361	Very poor	low	-0.244	1.827	7.331	55.417
NPRL3362	Good	good	-9.375	-4.389	0.871	94.092
NPRL3363	Good	good	-5.628	-2.676	1.123	107.878
NPRL3364	Good	opti mal	-5.082	-3.012	0.723	100.562
NPRL3365	Good	good	-3.086	-1.857	1.814	79.747
NPRL3366	Good	good	-7.923	-2.862	0.928	94.092
NPRL3367	Good	opti mal	-6.329	-2.101	0.200	109.492
NPRL3368	Good	good	-8.774	-6.647	0.857	94.092
NPRL3369	Good	good	-5.028	-5.313	1.108	107.878
NPRL3370	Good	good	-5.091	-4.190	1.065	104.364
NPRL3372	Good	low	-4.804	-2.849	1.593	87.063
NPRL3373	Good	low	-3.290	-2.757	1.977	98.948
NPRL3374	Very poor	good	-6.245	-14.312	-0.721	229.816
NPRL3375	Good	good	-1.915	-0.354	1.465	105.978
NPRL3376	Good	good	-2.921	4.239	2.636	76.791
NPRL3377	Very poor	low	-3.920	-14.063	-0.963	250.632
NPRL3378	Good	good	-2.866	1.869	1.448	114.908
NPRL3379	Good	good	-3.684	3.399	2.893	56.901
NPRL3380	Good	good	-7.781	-3.337	0.967	105.978
NPRL3381	Good	low	-4.947	-2.714	1.248	87.063
NPRL3382	Good	good	-7.675	-4.559	1.250	95.993
NPRL3383	Good	low	-4.444	-3.774	1.267	69.762
NPRL3386	Good	low	-2.450	9.834	3.624	38.116
NPRL3390	Moderate	low	-2.413	-1.617	2.931	122.224
NPRL3391	Good	opti mal	-3.964	-4.553	0.702	60.832
NPRL3393	Good	opti mal	-4.791	-3.982	-0.273	64.347
NPRL3394	Good	good	-4.872	-0.885	1.341	44.091
NPRL3395	Very poor	good	-7.251	-9.568	-0.305	204.704
NPRL3396	Good	good	-2.340	0.211	1.690	94.092
NPRL3397	Good	low	-2.537	2.923	3.054	70.881
NPRL3399	Good	good	-2.923	0.910	1.797	97.112
NPRL3400	Very poor	low	-4.512	-29.652	-0.431	237.405
NPRL3401	Good	opti mal	-6.275	-2.247	0.038	34.601
NPRL3402	Very poor	opti mal	-3.390	-17.068	-1.706	203.585
NPRL3403	Very poor	good	-2.986	-16.937	-1.019	216.030
NPRL3404	Very poor	good	-1.727	-19.177	-1.245	227.915
NPRL3405	Moderate	opti mal	-8.770	-16.241	-0.512	139.797

NPRL3406	Very poor	good	-10.847	-13.644	1.179	153.583
NPRL3407	Very poor	good	-8.720	-14.231	1.144	151.683
NPRL3416	Good	good	-7.468	-10.026	1.719	89.346
NPRL3417	Good	low	-5.468	-1.048	2.485	56.645
NPRL3426	Good	low	-3.323	1.460	3.955	61.391
NPRL3429	Very poor	opti mal	-8.482	-16.331	-0.549	157.098
NPRL3430	Moderate	very low, but possible	-2.525	2.589	5.048	87.622
NPRL3431	Moderate	very low, but possible	-2.525	1.851	5.048	87.622
NPRL3434	Very poor	low	-6.927	-10.791	2.593	185.230
NPRL3437	Very poor	good	-8.622	-18.469	-2.259	220.104
NPRL3441	Very poor	good	-5.214	-18.306	-1.632	224.960
NPRL3448	Poor	good	-8.188	-17.504	0.268	145.213
NPRL3449	Very poor	good	-6.558	-17.738	0.436	168.984
NPRL3459	Good	low	-3.117	1.069	3.123	82.207
NPRL3462	Good	opti mal	-6.413	-12.782	0.105	41.631
NPRL3463	Good	good	-4.797	-1.973	1.653	30.670
NPRL3464	Good	opti mal	-4.735	-13.137	0.834	44.834
NPRL3465	Good	good	-0.057	1.212	2.962	17.860
NPRL3466	Good	good	-6.734	0.239	1.912	47.971
NPRL3467	Good	good	-2.112	-7.715	2.943	86.614
NPRL3468	Good	good	-4.261	-14.136	1.628	59.491
NPRL3469	Good	low	-4.742	-3.282	2.985	61.859
NPRL3470	Good	low	-6.344	0.723	1.972	61.859
NPRL3471	Good	good	-7.424	-6.468	1.540	48.530
NPRL3472	Good	low	-2.684	3.457	1.052	53.021
NPRL3473	Good	good	-5.248	-4.218	0.078	73.836
NPRL3474	Good	low	-3.452	-5.957	1.598	47.854
NPRL3475	Good	good	-5.776	-6.541	1.171	59.739
NPRL3476	Good	good	-1.251	-5.667	2.281	24.019
NPRL3478	Good	low	1.159	7.832	3.795	41.068
NPRL3479	Good	low	-2.054	1.224	3.432	61.722
NPRL3480	Good	low	-5.570	7.779	3.021	75.670
NPRL3481	Good	low	-0.819	5.438	2.501	58.369
NPRL3482	Good	low	-0.257	7.158	3.779	49.998
NPRL3483	Good	low	-2.094	5.238	4.206	76.229
NPRL3484	Good	low	-1.613	4.223	2.983	58.369
NPRL3485	Moderate	very low, but possible	1.510	9.584	6.214	41.068
NPRL3486	Good	very low, but possible	-2.121	6.414	4.776	39.072
NPRL3487	Good	low	0.418	5.860	3.247	41.068
NPRL3488	Good	low	0.331	6.648	3.247	41.068
NPRL3489	Good	good	-1.262	-0.921	2.743	88.610
NPRL3491	Good	low	-1.147	-15.445	2.045	35.720
NPRL3492	Good	good	-1.321	-8.088	1.149	53.021
NPRL3494	Good	good	-1.321	-8.088	1.149	53.021
NPRL3495	Good	low	-5.636	-3.299	2.670	47.443
NPRL3496	Good	low	-5.815	-4.475	3.374	50.958
NPRL3498	Good	low	-3.164	-0.035	2.568	59.888
NPRL3499	Good	low	-3.326	-5.271	3.374	50.958
NPRL3500+A79:K79	Good	low	-4.178	-1.281	3.918	39.072
NPRL3501	Good	low	-4.178	-1.281	3.918	39.072
NPRL3502	Good	low	-0.518	-3.369	2.159	38.924
NPRL3503	Good	low	-4.071	0.048	3.174	50.958
NPRL3504	Good	low	-4.010	0.516	2.637	68.818
NPRL3505	Good	low	-0.576	-5.147	3.357	59.888
NPRL3506	Good	low	-2.000	-18.599	2.244	35.720
NPRL3507	Good	low	-2.000	-18.599	2.244	35.720
NPRL3508	Good	low	-0.277	-8.598	2.365	35.720
NPRL3509	Good	good	-2.662	-11.229	1.213	56.535
NPRL3510	Good	good	-2.662	-11.229	1.213	56.535
NPRL3511	Good	low	-0.923	1.758	2.953	56.373
NPRL3512	Good	good	-2.947	-3.247	0.702	70.321
NPRL3513	Good	good	-5.778	-3.945	1.243	73.526
NPRL3514	Good	good	-3.706	-7.654	1.646	56.535
NPRL3515	Good	good	-1.169	-4.427	1.591	56.225
NPRL3516	Good	low	-4.576	-4.741	3.599	39.072
NPRL3517	Good	very low, but possible	0.429	7.166	3.933	41.068
NPRL3518	Good	very low, but possible	1.339	8.183	4.389	41.068
NPRL3519	Good	good	-5.226	-3.247	0.939	59.739
NPRL3520	Good	good	-3.253	-6.855	2.697	86.614
NPRL3521	Good	good	-4.705	-5.500	1.993	83.099
NPRL3522	Good	good	-1.175	-1.362	2.743	88.610
NPRL3523	Good	low	0.514	4.046	3.646	41.068
NPRL3524	Good	very low, but possible	0.301	4.651	3.471	41.068
NPRL3525	Good	low	-6.783	-8.453	1.928	65.155
NPRL3526	Good	low	-5.148	-6.463	1.817	59.739
NPRL3527	Good	low	-1.890	-2.897	3.083	62.843

NPRL3528	Good	low	-1.629	-11.493	2.179	35.720
NPRL3529	Good	low	-2.082	-4.964	1.774	38.924
NPRL3530	Good	good	-1.358	-13.938	1.954	47.605
NPRL3531	Good	good	-2.722	-15.300	1.728	59.491
NPRL3532	Good	low	-0.406	5.807	4.132	41.068
NPRL3533	Good	low	1.942	1.102	4.214	97.607
NPRL3536	Good	low	-1.087	-2.069	3.161	97.607
NPRL3537	Moderate	low	0.066	2.385	5.017	97.607
NPRL3538	Good	low	3.699	3.942	4.348	88.677
NPRL3539	Poor	very low, but possible	2.147	3.375	6.081	109.492
NPRL3540	Moderate	low	0.733	6.309	4.957	88.677
NPRL3541	Poor	low	-0.835	5.595	6.690	109.492
NPRL3542	Good	opti mal	-4.306	-12.205	0.359	44.834
NPRL3543	Good	low	1.009	-4.417	3.417	6.704
NPRL3544	Moderate	low	0.783	2.405	3.138	119.764
NPRL3545	Very poor	low	-2.729	-0.823	4.336	158.440
NPRL3547	Good	opti mal	-5.754	-4.062	-0.716	72.717
NPRL3549	Moderate	very low, but possible	-3.678	0.483	5.525	79.747
NPRL3551	Very poor	too soluble	-5.010	-20.510	-3.581	160.613
NPRL3554	Good	low	-1.973	3.684	4.907	38.116
NPRL3555	Good	good	-10.026	-0.084	0.192	102.880
NPRL3556	Good	low	-9.178	0.802	1.577	91.670
NPRL3557	Good	low	-8.080	-9.697	2.992	65.291
NPRL3558	Good	low	-4.243	-14.802	2.176	77.177
NPRL3559	Good	good	-6.104	-1.018	1.666	55.976
NPRL3561	Good	good	-4.691	1.958	2.118	90.578
NPRL3562	Very poor	low	-5.211	-0.649	2.101	154.925
NPRL3564	Moderate	good	-9.568	-1.205	0.916	129.111
NPRL3565	Moderate	good	-10.136	-1.461	0.605	129.111
NPRL3566	Good	low	-11.348	1.142	1.265	91.670
NPRL3567	Good	good	-11.124	-0.212	-0.119	102.880
NPRL3568	Moderate	very low, but possible	-1.317	1.373	6.726	38.675
NPRL3569	Very poor	too soluble	-4.416	-8.278	-2.628	97.048
NPRL3572	Good	good	-5.170	-7.188	0.384	119.764
NPRL3573	Good	low	-3.180	1.854	3.087	99.508
NPRL3574	Good	low	-2.925	0.112	2.984	50.670
NPRL3575	Good	low	-4.789	2.751	4.409	21.484
NPRL3576	Very poor	low	-10.446	-18.560	0.484	249.290
NPRL3577	Good	opti mal	-4.028	-4.335	-0.122	72.555
NPRL3578	Good	good	-4.008	-5.601	1.374	55.254
NPRL3579	Good	good	0.097	1.441	2.803	46.884
NPRL3580	Good	good	-2.246	-0.202	3.069	89.236
NPRL3581	Good	low	-5.323	-3.373	3.557	59.166
NPRL3582	Good	low	-3.635	1.184	3.325	39.949
NPRL3583	Good	good	-3.011	3.705	2.534	34.601
NPRL3584	Good	good	-3.020	1.209	2.260	34.601
NPRL3585	Good	good	-2.955	1.622	2.429	63.722
NPRL3586	Good	good	-5.479	-6.744	1.641	54.792
NPRL3587	Good	good	-5.182	-5.506	1.919	88.786
NPRL3588	Good	good	-5.719	1.388	2.349	47.155
NPRL3589	Good	good	-4.091	0.946	2.058	99.508
NPRL3590	Good	opti mal	-6.226	-2.790	0.795	33.783
NPRL3591	Good	good	-2.419	-2.839	2.282	46.884
NPRL3592	Good	good	-2.670	0.099	2.798	38.116
NPRL3593	Good	good	-4.271	-2.404	1.717	58.931
NPRL3594	Poor	very low, but possible	-0.870	2.785	5.935	97.607
NPRL3595	Good	low	-2.362	7.232	4.083	69.762
NPRL3597	Very poor	good	-5.195	-16.727	-1.534	345.843
NPRL3599	Very poor	good	-5.811	-17.687	0.408	183.329
NPRL3600	Very poor	good	-5.915	-19.447	0.166	204.145
NPRL3601	Very poor	opti mal	-3.248	-15.689	-1.916	162.514
NPRL3602	Very poor	opti mal	-1.134	-17.093	-1.937	153.583
NPRL3603	Very poor	good	-6.162	-14.628	-0.280	183.329
NPRL3604	Very poor	good	-8.755	-18.884	-0.625	198.567
NPRL3605	Very poor	good	-11.258	-15.261	1.249	228.313
NPRL3606	Very poor	opti mal	-11.660	-16.435	-0.174	174.796
NPRL3607	Very poor	opti mal	-5.385	-12.824	-1.551	170.884
NPRL3608	Very poor	very low, but possible	-5.946	-7.281	7.246	72.170
NPRL3609	Poor	Extremely low	-2.407	-7.401	6.992	72.170
NPRL3610	Good	very low, but possible	-2.503	-12.708	5.458	60.137
NPRL3612	Good	low	0.730	2.099	3.152	56.373
NPRL3613	Good	low	-4.730	-8.873	3.373	50.958
NPRL3614	Good	low	-4.996	-1.061	3.977	39.072
NPRL3616	Good	low	0.428	4.914	3.646	41.068
NPRL3617	Good	low	-4.100	0.339	2.670	44.091
NPRL3618	Good	low	-2.134	-0.620	3.911	55.976

NPRL3619	Good	low	-1.894	0.044	4.136	44.091
NPRL3620	Good	very low, but possible	-0.330	0.538	5.337	55.976
NPRL3622	Good	good	-4.352	-0.796	2.438	44.091
NPRL3623	Good	good	-3.243	1.030	2.692	55.976
NPRL3624	Good	low	-3.674	4.193	2.760	67.699
NPRL3625	Good	good	-2.351	-1.600	2.346	41.675
NPRL3627	Good	low	-3.338	3.649	3.221	54.023
NPRL3628	Good	good	-2.425	2.034	2.643	80.144
NPRL3629	Poor	very low, but possible	-4.851	2.443	5.903	135.958
NPRL3630	Good	good	-1.089	0.048	2.721	50.398
NPRL3631	Good	low	-3.660	1.705	2.711	79.122
NPRL3632	Good	low	1.202	6.470	4.532	62.284
NPRL3633	Poor	very low, but possible	-1.528	-3.471	5.061	137.892
NPRL3634	Good	good	-2.044	1.502	2.819	79.682
NPRL3635	Good	good	-2.028	1.076	2.643	80.144
NPRL3636	Good	low	-0.184	3.564	4.500	80.144
NPRL3637	Good	low	-0.087	4.191	4.516	71.214
NPRL3638	Good	low	-1.528	3.093	2.869	68.259
NPRL3639	Good	low	-4.417	2.882	2.744	76.629
NPRL3641	Good	good	-6.657	-2.284	1.197	76.791
NPRL3642	Good	good	-6.859	-2.118	1.441	76.791
NPRL3644	Good	good	-2.887	-1.329	2.081	52.461
NPRL3645	Good	good	-1.509	-0.265	2.811	43.531
NPRL3646	Good	good	-2.722	-4.414	1.642	52.461
NPRL3647	Good	good	-4.346	-1.803	1.606	64.906
NPRL3648	Good	opti mal	-5.800	-1.657	0.839	97.607
NPRL3649	Good	good	-2.915	-1.582	2.329	55.976
NPRL3650	Good	good	-6.879	-2.284	1.181	85.722
NPRL3652	Good	good	-3.127	2.959	2.643	80.144
NPRL3653	Good	low	-5.515	2.403	2.518	88.515
NPRL3654	Good	good	-2.652	-0.520	2.594	91.567
NPRL3655	Good	low	-3.984	4.285	3.463	71.214
NPRL3656	Poor	very low, but possible	-2.353	1.351	6.014	114.843
NPRL3659	Good	good	-2.536	-3.214	1.867	44.091
NPRL3660	Good	low	-0.435	2.960	3.054	70.881
NPRL3661	Good	low	-3.311	3.806	3.054	70.881
NPRL3665	Good	good	-6.565	-2.579	1.962	55.417
NPRL3667	Good	good	-4.928	-5.749	1.904	106.287
NPRL3669	Good	good	-5.356	2.052	2.652	67.861
NPRL3671	Good	low	-0.483	4.400	3.134	31.976
NPRL3673	Good	good	-1.369	3.944	2.174	31.976
NPRL3674	Moderate	low	-3.331	-7.663	4.145	118.788
NPRL3677	Good	good	-5.767	-7.827	1.714	122.303
NPRL3678	Good	low	-1.515	3.438	2.620	31.976
NPRL3679	Good	low	-1.515	3.349	2.620	31.976
NPRL3680	Good	low	0.007	6.691	4.031	61.701
NPRL3682	Good	low	-3.120	3.960	2.902	95.959
NPRL3684	Good	low	-2.999	4.666	3.424	52.770
NPRL3685	Moderate	low	-2.358	4.946	4.607	97.607
NPRL3686	Good	very low, but possible	-1.024	6.377	5.259	76.791
NPRL3687	Good	low	-1.822	4.113	3.403	76.791
NPRL3688	Good	low	-2.091	2.598	3.159	85.722
NPRL3690	Moderate	low	-2.894	5.175	5.016	85.722
NPRL3691	Very poor	very low, but possible	-3.260	4.335	8.375	113.853
NPRL3692	Poor	low	-2.547	2.976	5.006	113.853
NPRL3693	Moderate	low	-1.804	4.828	4.317	101.122
NPRL3694	Good	low	-4.722	1.696	3.306	106.537
NPRL3695	Moderate	low	-6.256	-0.418	4.560	108.438
NPRL3696	Moderate	low	-5.933	-1.419	4.103	108.438
NPRL3697	Moderate	very low, but possible	-6.675	-0.298	5.205	87.622
NPRL3698	Good	low	-2.920	-3.112	3.710	69.762
NPRL3699	Good	low	-5.880	-2.110	1.793	79.252
NPRL3700	Good	low	-5.342	-1.571	2.239	103.582
NPRL3702	Good	low	-4.527	5.489	4.537	79.252
NPRL3703	Good	opti mal	-7.908	-7.387	-0.615	108.803
NPRL3704	Poor	Extremely low	-2.381	2.401	6.966	52.461
NPRL3705	Good	very low, but possible	-0.930	1.121	4.556	43.531
NPRL3706	Good	good	-2.274	-0.214	1.798	78.692
NPRL3707	Good	low	-3.511	5.275	3.902	96.552
NPRL3708	Good	low	-2.784	7.152	3.951	69.762
NPRL3709	Good	low	-0.906	3.284	3.402	34.601
NPRL3710	Good	opti mal	-9.364	-5.389	0.194	108.803
NPRL3711	Good	low	-0.786	5.341	5.679	52.461
NPRL3712	Good	low	-1.493	2.662	3.113	69.762
NPRL3713	Good	low	-0.456	-0.821	3.370	37.954
NPRL3714	Good	good	-5.737	-0.197	0.543	73.277

NPRL3715	Good	good	-5.756	-2.725	1.319	95.993
NPRL3716	Good	good	-4.692	1.657	0.826	106.343
NPRL3717	Good	opti mal	-1.960	-3.684	0.720	55.254
NPRL3718	Moderate	opti mal	-5.129	-9.527	-1.445	76.481
NPRL3719	Good	good	-3.851	-0.548	1.811	78.692
NPRL3720	Good	opti mal	-5.158	-1.290	1.191	101.627
NPRL3721	Good	low	-3.534	1.023	2.480	82.572
NPRL3722	Good	low	-2.700	7.079	4.419	69.762
NPRL3723	Good	low	-0.604	-1.243	3.301	38.116
NPRL3724	Good	low	-1.197	1.973	2.516	69.762
NPRL3725	Good	good	-3.459	0.176	1.195	52.461
NPRL3726	Moderate	too soluble	-2.355	-10.327	-1.492	60.222
NPRL3727	Good	very low, but possible	-0.930	1.121	4.556	43.531
NPRL3728	Good	good	-2.420	-0.617	2.164	60.832
NPRL3729	Good	good	-4.494	-1.768	1.362	67.861
NPRL3730	Good	opti mal	-5.427	-2.489	0.561	85.162
NPRL3731	Moderate	too soluble	-2.319	-3.450	-1.401	60.832
NPRL3732	Good	good	-3.883	-2.857	1.621	78.692
NPRL3733	Good	good	-5.694	-2.744	0.621	90.578
NPRL3734	Good	low	-1.197	1.973	2.516	69.762
NPRL3735	Good	very low, but possible	-0.501	-0.011	5.314	70.321
NPRL3736	Moderate	too soluble	-2.116	-2.962	-1.157	60.832
NPRL3737	Very poor	good	-5.245	-3.957	-1.072	163.855
NPRL3739	Good	good	-3.417	0.340	1.440	64.347
NPRL3740	Good	good	-3.056	-4.033	2.802	104.923
NPRL3741	Poor	low	-1.172	2.035	5.096	105.978
NPRL3742	Good	good	-3.309	-1.552	1.766	104.923
NPRL3743	Good	good	-2.007	-1.188	3.266	52.461
NPRL3744	Good	good	-2.777	-2.754	1.758	38.675
NPRL3746	Good	opti mal	-5.219	-2.080	0.242	85.162
NPRL3747	Good	good	-2.232	-1.154	2.353	52.461
NPRL3748	Good	good	-1.388	1.204	2.974	47.046
NPRL3749	Good	low	-4.023	-0.131	3.775	90.415
NPRL3750	Good	good	-2.312	-3.854	2.810	52.461
NPRL3751	Good	opti mal	-4.848	-5.772	0.176	108.803
NPRL3752	Very poor	low	-1.282	5.341	7.504	52.461
NPRL3753	Good	good	-3.529	-0.188	1.386	64.347
NPRL3754	Good	opti mal	-4.215	-2.097	-0.325	88.677
NPRL3755	Good	opti mal	-2.770	-1.734	1.773	52.461
NPRL3756	Good	good	-5.296	-1.897	1.291	52.461
NPRL3758	Good	good	-3.066	2.219	2.442	31.976
NPRL3759	Good	good	-3.066	2.130	2.442	31.976
NPRL3760	Good	low	-2.131	2.875	2.956	31.976
NPRL3761	Good	good	-3.488	1.805	1.995	31.976
NPRL3762	Good	good	-1.192	1.294	2.161	31.976
NPRL3763	Good	good	-1.562	1.906	2.228	31.976
NPRL3764	Good	low	-0.894	2.039	2.675	31.976
NPRL3765	Good	low	-0.566	1.741	2.786	31.976
NPRL3766	Good	low	-1.009	2.001	2.864	31.976
NPRL3767	Good	good	-2.928	1.810	2.364	31.976
NPRL3768	Good	low	-2.302	2.891	2.956	31.976
NPRL3769	Good	good	-3.237	2.146	2.442	31.976
NPRL3770	Good	good	-3.659	1.821	1.995	31.976
NPRL3771	Good	good	-3.237	2.235	2.442	31.976
NPRL3772	Good	good	-3.607	2.758	2.509	31.976
NPRL3773	Good	low	-0.668	2.810	3.378	31.976
NPRL3774	Good	good	-2.129	1.084	2.453	40.906
NPRL3775	Good	good	-3.066	0.030	2.007	40.906
NPRL3776	Good	good	-2.290	-0.439	1.862	40.906
NPRL3777	Good	good	-4.963	0.461	1.493	40.906
NPRL3778	Good	good	-2.696	-0.582	1.939	40.906
NPRL3779	Good	good	-2.696	-0.493	1.939	40.906
NPRL3781	Good	low	-2.073	2.482	2.418	31.976
NPRL3782	Good	low	-1.378	2.524	2.931	31.976
NPRL3783	Good	low	-1.009	1.912	2.864	31.976
NPRL3785	Good	low	-0.617	2.003	2.864	31.976
NPRL3786	Very poor	low	-0.730	1.370	3.386	158.440
NPRL3787	Moderate	very low, but possible	-0.919	3.528	4.944	100.626
NPRL3788	Good	very low, but possible	-1.112	3.574	4.842	65.466
NPRL3792	Good	very low, but possible	-0.140	1.671	5.041	65.466
NPRL3793	Very poor	low	-5.044	-0.036	4.093	150.628
NPRL3795	Good	low	-1.213	1.085	4.020	88.182
NPRL3796	Good	low	-0.820	-2.943	3.810	79.252
NPRL3797	Poor	very low, but possible	-1.425	2.104	5.303	106.042
NPRL3798	Good	low	-0.082	3.423	3.765	62.510
NPRL3799	Good	low	0.541	3.395	5.186	47.605

NPRL3800	Good	low	1.499	2.927	3.910	44.650
NPRL3801	Good	low	-0.984	4.523	4.370	69.203
NPRL3802	Good	low	-0.010	4.129	4.565	69.203
NPRL3803	Very poor	Extremely low	0.094	3.206	8.119	20.815
NPRL3804	Good	very low, but possible	0.567	5.622	4.887	60.832
NPRL3805	Moderate	opti mal	-7.356	-9.626	-0.144	23.857
NPRL3806	Very poor	very low, but possible	-6.388	-4.082	8.251	54.310
NPRL3807	Good	good	-5.217	-2.506	1.655	85.722
NPRL3808	Very poor	very low, but possible	1.848	-3.506	4.840	153.583
NPRL3809	Very poor	good	-8.134	-18.524	-1.033	247.676
NPRL3812	Very poor	good	-8.275	-15.128	-1.623	247.676
NPRL3813	Very poor	good	-8.347	-18.789	-1.049	247.676
NPRL3814	Very poor	good	-8.532	0.082	0.177	167.370
NPRL3815	Very poor	good	-7.397	-1.731	0.548	167.370
NPRL3817	Moderate	very low, but possible	-1.026	1.023	5.882	64.347
NPRL3818	Good	low	-1.995	-0.014	3.938	76.232
NPRL3819	Good	low	-2.045	-0.493	5.178	62.446
NPRL3820	Good	very low, but possible	-1.007	2.562	4.283	69.203
NPRL3821	Good	opti mal	-2.879	-6.134	2.273	58.484
NPRL3825	Good	low	-3.952	3.120	3.522	73.277
NPRL3826	Good	good	-6.771	-3.344	1.393	64.347
NPRL3827	Good	good	-5.093	-0.895	2.339	90.578
NPRL3828	Good	good	-3.040	-0.547	1.690	94.092
NPRL3829	Good	good	-5.737	-0.197	0.543	73.277
NPRL3830	Good	opti mal	-5.034	-0.571	-0.384	107.878
NPRL3831	Good	good	-9.771	-4.222	-0.300	82.207
NPRL3832	Good	good	-4.362	-0.345	1.819	69.762
NPRL3833	Good	good	-4.234	0.388	1.440	64.347
NPRL3834	Good	good	-7.447	-1.646	1.425	64.347
NPRL3835	Good	good	-5.581	2.310	0.646	104.364
NPRL3837	Good	good	-5.505	2.500	1.103	104.364
NPRL3839	Good	good	-5.619	-1.998	1.804	69.762
NPRL3840	Good	good	-4.135	0.479	1.777	64.347
NPRL3841	Very poor	very low, but possible	-2.182	3.743	8.416	69.762
NPRL3842	Good	good	-7.162	-1.387	1.448	64.347
NPRL3843	Good	opti mal	-3.882	-2.060	0.901	67.861
NPRL3844	Good	good	-3.897	-6.968	2.154	38.924
NPRL3845	Good	low	-1.156	0.902	2.939	78.692
NPRL3846	Good	low	-2.983	1.133	2.434	52.461
NPRL3847	Very poor	good	-8.739	-4.276	-0.136	165.469
NPRL3848	Good	good	-5.715	-2.293	1.280	73.277
NPRL3849	Very poor	Extremely low	0.794	-5.511	7.553	211.956
NPRL3850	Moderate	low	-0.186	-2.040	3.790	117.863
NPRL3851	Very poor	very low, but possible	1.406	-3.113	5.625	164.910
NPRL3852	Very poor	very low, but possible	-2.242	-1.128	6.056	153.024
NPRL3853	Very poor	opti mal	-3.146	-0.055	8.234	201.476
NPRL3854	Very poor	low	-2.244	-0.100	5.255	157.385
NPRL3855	Poor	low	1.480	-4.189	4.823	109.492
NPRL3856	Very poor	very low, but possible	-0.994	-1.273	7.463	160.054
NPRL3857	Poor	low	-1.629	0.680	4.925	109.492
NPRL3858	Good	good	-6.695	1.417	2.474	90.794
NPRL3863	Good	opti mal	-5.639	-3.091	0.389	76.232
NPRL3864	Good	opti mal	-8.565	-5.984	-0.039	71.997
NPRL3865	Good	opti mal	-6.263	-4.578	-0.397	88.685
NPRL3866	Very poor	too soluble	-3.111	-5.923	-5.793	124.620
NPRL3867	Poor	low	-3.207	2.182	4.144	139.525
NPRL3868	Good	low	-0.981	-2.750	4.466	54.441
NPRL3869	Poor	good	-2.277	-16.286	0.686	148.168
NPRL3870	Very poor	good	-3.704	-9.982	-1.644	216.030
NPRL3871	Very poor	low	-4.379	-12.815	0.347	210.615
NPRL3872	Good	low	-2.490	-8.807	3.278	58.866
NPRL3873	Good	good	-2.416	-0.090	3.074	60.416
NPRL3874	Moderate	very low, but possible	-0.898	-5.318	6.664	46.981
NPRL3875	Good	opti mal	-4.951	-15.120	-0.583	68.065
NPRL3876	Good	opti mal	-3.733	-13.618	-1.030	68.065
NPRL3877	Moderate	low	0.735	16.329	4.104	104.344
NPRL3878	Poor	low	-4.424	12.712	3.793	147.167
NPRL3879	Moderate	very low, but possible	-0.239	14.336	3.136	127.532
NPRL3880	Good	low	-1.706	10.498	2.337	100.992
NPRL3881	Moderate	very low, but possible	-1.733	13.617	2.615	133.246
NPRL3883	Very poor	good	-7.642	-10.379	1.787	195.214
NPRL3885	Moderate	low	-12.962	-5.654	2.152	133.327
NPRL3888	Very poor	very low, but possible	0.580	5.551	7.639	20.815
NPRL3889	Very poor	very low, but possible	-0.414	8.010	7.639	20.815
NPRL3890	Good	good	-0.454	0.290	2.528	35.160
NPRL3891	Very poor	good	-11.997	7.428	6.304	179.710

NPRL3892	Good	low	-5.834	2.881	4.296	79.252
NPRL3893	Good	good	-6.076	3.739	3.321	70.321
NPRL3894	Good	good	-9.392	6.162	3.918	100.067
NPRL3895	Good	good	-5.592	3.306	1.889	52.461
NPRL3896	Good	low	-0.594	5.072	4.669	60.889
NPRL3897	Good	good	-4.406	2.325	2.218	35.160
NPRL3898	Good	good	-3.824	3.500	3.410	64.906
NPRL3899	Good	good	-2.003	4.629	3.427	55.976
NPRL3900	Good	good	-2.684	1.510	2.227	35.160
NPRL3901	Good	good	-7.216	1.927	1.995	53.021
NPRL3902	Good	good	-5.807	0.772	2.211	44.091
NPRL3903	Good	opti mal	-3.541	-1.303	0.852	67.861
NPRL3904	Good	low	-3.028	-0.611	4.221	39.072
NPRL3905	Good	low	-2.380	1.416	2.536	43.531
NPRL3906	Very poor	Extremely low	-2.216	2.718	7.070	52.461
NPRL3908	Very poor	Extremely low	-2.573	3.423	7.151	52.461
NPRL3909	Very poor	low	-7.483	-11.510	4.585	201.295
NPRL3910	Good	low	-2.900	18.163	2.111	91.696
NPRL3911	Good	good	-3.950	0.729	1.463	64.347
NPRL3914	Good	opti mal	-5.338	-8.112	-0.548	67.551
NPRL3915	Good	low	-1.639	0.967	3.182	17.300
NPRL3916	Good	low	-2.402	-0.241	2.677	17.860
NPRL3917	Good	good	-1.213	0.863	2.879	56.061
NPRL3918	Good	low	-0.280	0.753	3.101	47.131
NPRL3919	Good	low	0.089	0.378	3.101	47.131
NPRL3920	Good	very low, but possible	-1.991	8.371	5.465	20.191
NPRL3921	Good	low	4.023	5.016	4.369	41.006
NPRL3922	Good	low	-0.722	3.443	3.688	49.936
NPRL3923	Good	low	-3.254	1.182	3.704	41.006
NPRL3924	Poor	too soluble	-6.042	-6.205	-0.896	148.455
NPRL3925	Very poor	opti mal	-6.903	-6.201	-2.005	260.903
NPRL3926	Very poor	too soluble	-6.270	-17.942	-5.000	195.580
NPRL3927	Very poor	very low, but possible	-2.545	-15.910	-1.158	270.106
NPRL3930	Very poor	Extremely low	-1.337	0.212	13.597	64.347
NPRL3931	Good	very low, but possible	-1.461	0.966	4.182	72.717
NPRL3932	Very poor	opti mal	-3.684	-16.756	-0.493	165.469
NPRL3933	Very poor	low	-6.433	-13.323	1.227	193.983
NPRL3936	Very poor	low	-3.510	-19.493	0.258	202.244
NPRL3937	Very poor	too soluble	-7.383	-14.868	-2.604	162.733
NPRL3939	Moderate	very low, but possible	-3.678	0.385	5.525	79.747
NPRL3940	Very poor	good	-8.963	-7.164	-1.753	170.884
NPRL3941	Very poor	good	-6.433	-15.301	1.243	185.053
NPRL3942	Very poor	good	-6.594	-5.088	0.213	211.956
NPRL3943	Very poor	good	-4.194	-16.396	-1.706	203.585
NPRL3944	Very poor	opti mal	-5.422	-15.384	-0.267	153.583
NPRL3945	Very poor	good	-5.914	-16.863	1.018	196.938
NPRL3946	Moderate	good	-11.115	-9.370	-0.022	129.253
NPRL3947	Very poor	low	-6.295	-17.165	0.776	217.753
NPRL3949	Very poor	good	-1.683	-16.838	-1.003	207.100
NPRL3951	Poor	opti mal	-6.412	-15.238	-0.251	144.653
NPRL3953	Moderate	very low, but possible	-3.632	1.709	6.492	58.931
NPRL3954	Very poor	very low, but possible	11.605	-1.313	5.040	159.494
NPRL3955	Good	good	-5.769	-2.815	1.815	113.802
NPRL3956	Good	low	-2.049	1.522	3.633	51.851
NPRL3957	Good	good	-4.570	0.934	1.588	50.364
NPRL3958	Good	good	-4.931	-2.121	2.213	105.431
NPRL3960	Good	good	-3.167	-2.389	2.246	87.571
NPRL3961	Good	good	-6.613	-1.661	1.195	68.065
NPRL3962	Good	opti mal	-8.049	-9.128	-0.388	82.744
NPRL3963	Good	good	-5.452	-2.136	0.924	50.364
NPRL3964	Good	good	-3.625	-1.702	1.913	60.222
NPRL3965	Good	good	-8.652	-5.903	0.514	76.995
NPRL3966	Good	opti mal	-8.384	-7.034	0.298	94.295
NPRL3967	Good	good	-8.182	-3.517	1.017	68.065
NPRL3968	Good	low	-1.261	2.134	3.242	60.222
NPRL3969	Very poor	opti mal	-4.128	-25.267	-1.636	180.869
NPRL3970	Good	too soluble	-3.303	-7.041	0.110	100.562
NPRL3971	Good	too soluble	-3.096	-6.881	0.110	100.562
NPRL3972	Moderate	too soluble	-1.059	-6.629	-0.856	121.378
NPRL3973	Very poor	too soluble	-2.877	-12.179	-3.098	62.446
NPRL3974	Very poor	too soluble	-4.679	-22.684	-1.257	156.539
NPRL3975	Good	opti mal	-3.763	-3.486	0.490	76.232
NPRL3976	Very poor	too soluble	-1.768	-13.205	-2.440	96.072
NPRL3977	Very poor	good	-4.369	-13.323	-0.465	186.284
NPRL3978	Very poor	low	-2.993	-19.118	-0.004	210.615
NPRL3979	Very poor	low	-3.988	-13.898	-1.636	254.706

NPRL3980	Very poor	good	-4.462	-13.262	-0.239	174.399
NPRL3982	Very poor	good	-8.327	-16.927	0.127	236.845
NPRL3983	Good	low	-1.051	-5.594	3.386	29.745
NPRL3984	Good	low	-2.123	5.621	4.805	50.926
NPRL3985	Very poor	low	-5.969	-11.305	4.725	193.542
NPRL3986	Very poor	low	-7.665	-12.123	4.341	204.809
NPRL3987	Very poor	low	-6.151	-11.837	4.481	197.057
NPRL3988	Very poor	low	-8.275	-12.076	4.116	216.695
NPRL3989	Very poor	low	-6.760	-12.647	4.256	208.942
NPRL3990	Good	very low, but possible	-1.121	-4.159	4.623	8.930
NPRL3992	Very poor	opti mal	-4.433	-20.072	-1.905	164.151
NPRL3994	Good	good	-4.083	4.671	1.660	115.541
NPRL3995	Good	good	-4.892	5.067	1.660	115.541
NPRL3996	Good	good	-4.208	6.380	2.363	119.055
NPRL3997	Good	good	-5.211	-1.620	1.031	115.541
NPRL3998	Good	good	-5.888	3.459	1.487	115.541
NPRL3999	Moderate	opti mal	-8.998	-4.560	-0.328	128.351
NPRL4000	Moderate	opti mal	-8.536	-4.234	0.128	128.351
NPRL4001	Very poor	good	-10.483	10.726	2.220	223.712
NPRL4002	Good	low	-5.473	-1.245	3.531	80.306
NPRL4003	Good	very low, but possible	-1.186	1.278	4.973	55.417
NPRL4004	Good	good	-1.486	-2.506	2.054	97.048
NPRL4006	Very poor	good	-8.198	20.759	2.937	223.712
NPRL4007	Very poor	too soluble	-8.533	33.268	7.124	324.704
NPRL4008	Good	good	-8.983	-1.690	0.700	73.277
NPRL4009	Good	good	-5.619	-1.998	1.804	69.762
NPRL4010	Good	low	-1.829	0.672	3.169	52.461
NPRL4011	Good	good	-2.741	-0.575	1.448	114.908
NPRL4012	Good	good	-8.066	-0.858	1.173	64.347
NPRL4013	Good	good	-8.915	-0.744	1.140	73.277
NPRL4014	Good	good	-5.334	-1.658	1.827	69.762
NPRL4016	Poor	Extremely low	-3.754	2.401	6.871	64.347
NPRL4017	Poor	Extremely low	-1.349	1.691	6.718	47.046
NPRL4018	Very poor	Extremely low	-2.945	2.773	7.097	52.461
NPRL4019	Good	good	-8.019	-2.907	1.446	64.347
NPRL4020	Good	low	-1.710	3.047	2.902	78.692
NPRL4021	Good	low	-1.529	-12.234	3.503	46.981
NPRL4022	Very poor	good	-7.683	-11.894	0.157	150.069
NPRL4023	Good	low	1.856	8.158	4.341	43.531
NPRL4025	Good	low	-1.600	-2.920	2.481	43.531
NPRL4027	Good	good	-5.737	-0.197	0.543	73.277
NPRL4028	Good	good	-8.950	-2.232	0.528	73.277
NPRL4030	Good	good	-10.094	-1.934	0.220	82.207
NPRL4031	Good	opti mal	-5.580	0.801	1.410	64.906
NPRL4032	Very poor	Extremely low	-2.826	8.594	8.084	20.815
NPRL4033	Good	opti mal	-5.056	-11.925	0.119	64.347
NPRL4034	Good	good	-4.362	-0.345	1.819	69.762
NPRL4035	Good	good	-3.033	0.877	1.323	52.461
NPRL4036	Good	good	-1.253	-0.112	1.669	64.347
NPRL4037	Good	good	-5.991	-6.845	0.118	67.551
NPRL4038	Good	opti mal	-2.405	-9.621	-0.942	67.551
NPRL4039	Good	good	-5.074	-7.165	0.369	67.551
NPRL4040	Good	good	-3.179	-0.118	1.023	64.347
NPRL4041	Good	opti mal	-5.236	-6.941	-0.064	67.551
NPRL4042	Good	opti mal	-5.338	-8.112	-0.548	67.551
NPRL4043	Good	opti mal	-5.053	-7.772	-0.525	67.551
NPRL4044	Good	opti mal	-5.520	-7.622	-0.087	67.551
NPRL4046	Good	good	-6.487	-7.338	-0.169	72.966
NPRL4047	Good	opti mal	-5.108	-9.351	-0.319	64.347
NPRL4048	Good	opti mal	-5.210	-9.691	-0.342	64.347
NPRL4049	Good	good	-8.994	-3.399	1.511	88.352
NPRL4050	Good	good	-11.111	-5.345	0.411	88.352
NPRL4051	Good	low	-4.611	1.963	3.960	69.762
NPRL4052	Good	good	-3.892	-3.706	0.944	73.277
NPRL4054	Good	low	-1.135	-0.250	2.758	43.531
NPRL4055	Good	good	-8.376	-3.967	0.492	103.697
NPRL4056	Good	low	-0.661	2.553	3.152	52.461
NPRL4057	Good	low	-1.946	0.238	2.508	43.531
NPRL4058	Good	very low, but possible	-2.203	4.641	4.610	52.461
NPRL4059	Good	good	-5.298	-0.302	2.505	47.046
NPRL4060	Good	good	-1.524	-3.667	1.072	64.347
NPRL4061	Good	good	-7.534	-4.333	0.874	65.636
NPRL4063	Good	good	-9.728	-3.399	1.120	91.867
NPRL4064	Very poor	too soluble	-2.948	-9.824	-2.442	70.755
NPRL4065	Good	good	-7.670	-5.405	1.788	73.277
NPRL4066	Good	good	-6.911	-2.039	1.655	86.993

NPRL4067	Good	good	-5.857	-1.087	1.539	64.347
NPRL4068	Good	good	-7.692	-5.028	2.599	73.277
NPRL4069	Good	good	-9.787	-5.446	0.899	81.648
NPRL4070	Very poor	too soluble	-3.505	-6.934	-2.383	72.555
NPRL4071	Good	opti mal	-5.805	-0.830	-0.122	78.133
NPRL4072	Good	good	-3.704	0.577	2.580	78.692
NPRL4073	Good	low	-7.035	-1.008	2.028	44.091
NPRL4074	Good	opti mal	-4.133	-4.209	0.336	82.937
NPRL4075	Good	very low, but possible	0.553	9.462	4.549	43.531
NPRL4076	Good	low	-0.040	3.823	4.166	0.000
NPRL4077	Good	low	-3.555	2.396	3.165	70.321
NPRL4078	Moderate	low	-1.614	4.942	5.273	82.207
NPRL4079	Good	low	-2.161	0.611	3.081	87.063
NPRL4080	Good	good	-2.843	-5.377	2.058	29.434
NPRL4082	Good	very low, but possible	1.004	5.395	5.762	52.461
NPRL4083	Moderate	too soluble	-4.211	-6.202	-1.301	58.607
NPRL4085	Moderate	too soluble	-3.381	-7.569	-1.391	58.607
NPRL4086	Good	opti mal	-2.135	-8.269	-1.002	75.908
NPRL4087	Good	opti mal	-5.335	-6.711	0.329	72.555
NPRL4088	Good	opti mal	-1.764	-6.725	-0.465	75.908
NPRL4089	Very poor	very low, but possible	-4.960	1.483	7.181	78.692
NPRL4090	Moderate	opti mal	-2.904	-15.306	-1.120	68.065
NPRL4091	Good	low	-2.269	2.961	3.497	43.531
NPRL4092	Good	good	-2.893	-10.388	0.869	35.720
NPRL4093	Good	low	-1.410	2.833	3.236	30.670
NPRL4094	Good	opti mal	-5.560	-13.563	0.365	29.745
NPRL4095	Good	low	0.202	-0.290	3.529	21.212
NPRL4096	Good	good	-2.893	-10.388	0.869	35.720
NPRL4097	Good	opti mal	-5.936	-3.100	1.065	42.555
NPRL4098	Moderate	opti mal	-4.866	-13.367	0.590	17.860
NPRL4099	Good	good	-4.284	0.742	2.163	60.683
NPRL4100	Good	low	-1.704	-0.921	3.412	54.310
NPRL4102	Good	low	0.144	2.667	4.323	17.860
NPRL4103	Moderate	good	-7.693	-3.190	1.497	128.351
NPRL4104	Moderate	good	-7.227	-2.223	0.658	128.351
NPRL4108	Good	opti mal	-5.809	-2.266	0.055	60.832
NPRL4112	Good	low	-2.112	18.321	3.860	34.601
NPRL4113	Good	good	-4.617	2.946	-0.097	86.503
NPRL4114	Good	good	-1.837	-3.200	2.588	62.446
NPRL4115	Good	good	-5.411	-11.281	1.240	93.533
NPRL4117	Good	low	-1.542	6.609	3.938	38.116
NPRL4118	Good	low	-2.035	8.117	3.473	38.116
NPRL4119	Good	low	-2.038	4.622	3.338	38.116
NPRL4120	Good	low	-1.436	5.589	3.838	41.631
NPRL4121	Good	good	-5.087	-34.505	1.217	93.533
NPRL4123	Good	low	-1.071	7.104	3.688	34.601
NPRL4124	Good	low	0.276	1.188	5.137	41.631
NPRL4127	Good	good	-3.255	0.251	1.744	82.207
NPRL4128	Good	good	-2.871	1.482	1.744	82.207
NPRL4129	Moderate	opti mal	-7.788	-8.590	-0.353	130.308
NPRL4130	Good	opti mal	-6.512	-4.966	0.282	104.077
NPRL4131	Good	good	-3.672	-0.005	1.842	69.762
NPRL4132	Good	low	-2.019	4.434	3.156	34.601
NPRL4133	Moderate	good	-8.857	-8.753	0.693	135.723
NPRL4134	Good	low	0.529	0.965	3.238	35.160
NPRL4135	Good	low	0.247	-0.669	3.423	38.675
NPRL4137	Good	very low, but possible	-2.757	-2.365	4.442	44.091
NPRL4138	Good	good	-9.131	-6.418	0.662	109.492
NPRL4139	Good	opti mal	-5.542	-7.224	-0.852	75.908
NPRL4140	Good	low	-0.094	-1.076	2.624	60.832
NPRL4141	Good	low	-1.737	6.632	3.573	34.601
NPRL4142	Poor	good	-8.072	-3.524	1.702	145.868
NPRL4143	Good	good	-3.834	-3.199	1.848	95.942
NPRL4144	Very poor	good	-10.508	-7.445	1.799	157.944
NPRL4145	Moderate	good	-8.826	-15.630	0.283	136.283
NPRL4148	Good	good	-4.669	-0.688	1.316	64.347
NPRL4149	Good	low	-6.139	1.573	2.542	107.878
NPRL4150	Good	very low, but possible	-4.761	2.318	3.849	87.063
NPRL4152	Good	low	-1.840	-1.483	3.169	90.578
NPRL4155	Good	good	-2.320	1.455	2.119	43.531
NPRL4156	Good	good	-4.336	-15.267	1.502	71.376
NPRL4157	Good	low	-4.766	-1.101	2.774	43.531
NPRL4158	Good	low	-3.544	2.492	2.576	52.461
NPRL4159	Good	low	-4.172	-2.265	3.177	62.843
NPRL4160	Good	low	-4.172	-2.265	3.177	62.843
NPRL4161	Poor	too soluble	-4.149	-9.229	-0.671	17.300

NPRL4162	Good	good	-7.915	-3.939	1.449	112.683
NPRL4163	Good	good	-6.370	-6.505	0.481	94.855
NPRL4164	Good	good	-6.763	-10.638	0.704	85.330
NPRL4165	Good	good	-5.123	-2.324	1.365	60.222
NPRL4166	Good	low	-3.037	0.336	2.886	60.222
NPRL4167	Good	good	-5.332	-2.088	1.880	78.082
NPRL4169	Good	low	-0.780	2.114	3.242	60.222
NPRL4170	Good	opti mal	-7.028	-8.361	-0.388	82.744
NPRL4171	Moderate	good	-8.541	-2.599	1.416	130.544
NPRL4172	Moderate	good	-8.541	-2.946	1.416	130.544
NPRL4173	Good	good	-3.909	-3.908	1.815	113.802
NPRL4175	Moderate	low	-4.421	-0.539	5.015	95.942
NPRL4176	Good	low	-2.034	-0.284	5.047	78.082
NPRL4177	Good	opti mal	-9.585	-7.660	0.154	120.444
NPRL4178	Good	low	-0.122	2.384	3.640	51.851
NPRL4180	Good	good	-6.091	-6.341	0.498	85.925
NPRL4182	Good	opti mal	-7.737	-10.927	-0.620	79.326
NPRL4184	Good	good	-8.230	-5.412	0.481	94.855
NPRL4185	Good	good	-10.552	-6.679	0.425	110.888
NPRL4186	Good	opti mal	-7.722	-5.102	0.531	68.065
NPRL4187	Good	opti mal	-9.179	-10.655	-0.084	83.120
NPRL4188	Good	low	-2.988	-3.394	3.036	64.347
NPRL4189	Good	low	-2.830	2.205	3.278	43.531
NPRL4190	Good	low	-2.703	0.638	3.052	55.417
NPRL4191	Good	good	-3.325	1.192	1.863	51.902
NPRL4192	Good	low	-3.234	2.639	3.296	76.232
NPRL4193	Good	low	0.299	1.302	3.536	43.531
NPRL4194	Good	low	-1.052	1.503	3.311	55.417
NPRL4195	Moderate	opti mal	-5.046	-3.165	-0.178	140.761
NPRL4197	Good	low	-0.098	1.661	3.632	55.417
NPRL4198	Good	very low, but possible	0.708	3.881	5.446	43.531
NPRL4199	Good	low	-1.427	3.454	3.748	52.461
NPRL4200	Good	good	-2.920	-0.796	1.961	76.232
NPRL4201	Good	low	-4.049	1.713	2.444	94.823
NPRL4202	Good	good	-5.247	-0.444	1.879	91.252
NPRL4203	Moderate	good	-7.316	0.856	1.589	136.454
NPRL4204	Good	good	-8.288	-0.167	1.178	104.062
NPRL4206	Good	low	-3.441	2.280	2.343	60.222
NPRL4207	Good	low	-3.355	0.425	2.594	52.461
NPRL4208	Good	low	-1.024	-3.367	2.775	52.461
NPRL4209	Good	good	-3.675	-4.775	1.719	97.048
NPRL4210	Good	low	-0.195	3.255	4.264	52.461
NPRL4211	Moderate	opti mal	-1.964	-6.102	-1.363	107.205
NPRL4212	Good	good	-3.933	0.701	1.630	78.133
NPRL4213	Good	good	-3.073	-1.932	2.324	76.232
NPRL4214	Good	low	-2.927	3.088	4.466	81.648
NPRL4215	Good	good	-2.975	2.385	2.343	87.063
NPRL4216	Good	good	-3.276	0.800	2.343	87.063
NPRL4217	Good	low	-1.753	9.664	3.613	70.881
NPRL4218	Good	low	-4.477	7.340	0.817	103.135
NPRL4219	Good	good	-4.592	12.193	0.591	115.020
NPRL4220	Good	low	-4.505	15.886	2.609	121.807
NPRL4221	Good	low	-1.706	8.666	2.606	70.881
NPRL4222	Good	good	-3.015	23.331	1.886	103.582
NPRL4223	Good	low	-2.278	17.590	2.490	97.112
NPRL4224	Good	low	-2.111	15.136	3.388	82.766
NPRL4225	Good	low	-1.459	16.319	4.361	82.766
NPRL4226	Good	low	-0.980	15.864	3.830	91.696
NPRL4227	Poor	very low, but possible	-0.535	16.471	6.634	91.696
NPRL4228	Good	low	-0.192	17.103	3.045	82.766
NPRL4229	Good	low	-0.094	12.389	3.271	70.881
NPRL4230	Moderate	very low, but possible	-3.100	17.837	4.631	95.577
NPRL4231	Good	very low, but possible	-3.094	12.980	4.856	83.691
NPRL4232	Good	very low, but possible	-1.089	21.749	4.547	95.577
NPRL4233	Good	very low, but possible	-1.083	16.891	4.772	83.691
NPRL4234	Good	low	-0.783	15.890	3.737	86.119
NPRL4235	Good	low	-1.326	10.466	4.055	79.811
NPRL4237	Good	low	-0.751	10.309	3.963	74.233
NPRL4238	Very poor	good	-14.279	-1.298	2.745	157.385
NPRL4239	Good	very low, but possible	-0.112	22.261	4.547	95.577
NPRL4240	Good	very low, but possible	-0.105	17.403	4.772	83.691
NPRL4241	Good	very low, but possible	-0.479	20.604	4.088	95.577
NPRL4242	Good	very low, but possible	-0.560	15.746	4.313	83.691
NPRL4243	Good	very low, but possible	-3.492	17.616	4.461	95.577
NPRL4252	Good	very low, but possible	-5.486	0.020	3.760	82.207
NPRL4255	Good	good	-4.659	-1.964	1.481	38.513

NPRL4256	Good	opti mal	-5.556	-8.486	-0.424	59.180
NPRL4257	Good	good	-11.653	-3.386	1.402	41.468
NPRL4260	Very poor	low	-4.759	-9.399	1.770	276.303
NPRL4261	Good	too soluble	-5.815	-9.111	-0.795	97.048
NPRL4263	Very poor	good	-4.192	-8.260	0.507	208.441
NPRL4264	Poor	good	-6.576	-3.205	1.232	144.094
NPRL4265	Very poor	good	-4.991	-1.693	2.735	185.725
NPRL4266	Good	good	-4.572	-1.781	1.514	87.622
NPRL4267	Moderate	low	-0.414	5.230	4.755	94.092
NPRL4271	Good	good	-2.664	0.434	2.305	53.021
NPRL4274	Good	low	-4.295	6.281	3.258	64.563
NPRL4275	Moderate	good	-2.911	12.136	1.634	127.353
NPRL4277	Good	low	-3.692	4.279	3.437	39.126
NPRL4278	Good	low	-3.371	3.304	3.168	98.456
NPRL4279	Good	low	-0.989	18.840	2.623	117.927
NPRL4280	Good	low	-3.566	6.658	3.744	64.563
NPRL4281	Good	low	-3.348	5.836	3.241	73.493
NPRL4283	Good	good	-4.650	5.090	2.749	110.834
NPRL4284	Good	low	-3.137	4.293	3.359	86.503
NPRL4285	Good	good	-3.858	-8.736	1.549	50.809
NPRL4286	Good	good	-4.626	-7.835	1.050	38.924
NPRL4287	Good	low	-2.118	2.797	3.043	64.282
NPRL4288	Good	low	-2.110	3.562	2.844	64.282
NPRL4289	Good	low	-2.395	6.146	2.876	46.422
NPRL4290	Good	low	-3.026	5.447	2.860	55.352
NPRL4292	Good	good	-2.847	-9.474	0.128	73.277
NPRL4293	Good	good	-4.604	-3.544	0.952	64.906
NPRL4294	Good	good	-4.604	-3.544	0.952	64.906
NPRL4295	Good	good	-3.934	-12.699	1.644	50.561
NPRL4296	Good	good	-2.797	-13.689	1.160	67.861
NPRL4298	Good	good	-2.295	-5.981	1.084	53.021
NPRL4299	Good	good	1.587	-0.535	1.525	53.021
NPRL4300	Good	good	1.006	-1.523	1.724	53.021
NPRL4301	Good	good	-1.874	-6.552	1.283	53.021
NPRL4302	Good	low	0.642	2.113	3.420	52.954
NPRL4303	Good	good	-3.212	-11.109	1.122	64.906
NPRL4304	Good	good	-3.485	-13.085	1.369	64.906
NPRL4305	Good	low	-1.537	-1.902	2.605	47.443
NPRL4306	Good	low	-4.456	-5.886	3.636	42.028
NPRL4307	Good	good	-2.029	-13.794	1.385	55.976
NPRL4309	Good	low	1.970	-1.649	2.448	21.064
NPRL4310	Good	low	-5.663	-5.204	3.620	50.958
NPRL4311	Good	good	-1.321	-8.088	1.149	53.021
NPRL4312	Good	good	-2.246	-11.359	1.348	53.021
NPRL4313	Good	low	-1.019	-4.483	2.659	58.369
NPRL4315	Good	good	-4.143	-12.407	1.143	76.791
NPRL4316	Good	good	-2.553	2.368	0.791	44.091
NPRL4317	Good	good	-2.517	-5.006	1.100	44.091
NPRL4318	Good	good	1.035	1.844	1.542	44.091
NPRL4319	Good	low	-2.755	-5.735	1.774	38.924
NPRL4320	Good	low	-3.409	-4.853	3.148	62.843
NPRL4321	Good	low	1.539	-0.208	2.216	38.924
NPRL4322	Good	very low, but possible	-3.485	12.575	4.686	83.691
NPRL4324	Good	good	-2.317	-7.674	1.057	64.906
NPRL4328	Good	low	-3.983	1.603	2.815	39.600
NPRL4329	Good	good	-3.572	-5.908	1.575	29.745
NPRL4330	Good	good	-5.015	-6.506	0.845	47.295
NPRL4331	Good	good	-2.968	-3.509	1.178	53.021
NPRL4334	Good	low	-1.781	-6.006	1.774	38.924
NPRL4335	Good	low	-6.128	-5.896	2.139	47.443
NPRL4337	Poor	low	-2.227	34.944	3.772	134.520
NPRL4338	Moderate	low	-0.508	28.614	3.797	108.997
NPRL4339	Poor	low	-6.939	15.262	2.535	139.417
NPRL4340	Moderate	low	-5.516	12.374	2.275	125.589
NPRL4341	Good	low	-1.804	13.707	2.381	82.766
NPRL4342	Good	low	-2.683	12.833	1.634	109.306
NPRL4343	Good	opti mal	-6.719	-1.985	0.610	61.701
NPRL4345	Poor	low	-5.320	12.748	3.049	139.108
NPRL4346	Moderate	low	-4.601	16.502	3.022	130.178
NPRL4347	Poor	low	-6.117	16.423	3.161	135.812
NPRL4348	Good	low	-5.363	16.693	3.282	112.877
NPRL4349	Poor	Extremely low	1.832	20.010	6.539	82.766
NPRL4350	Good	low	-0.273	15.196	3.289	82.766
NPRL4351	Good	low	-4.088	10.456	1.822	117.368
NPRL4352	Good	low	-0.251	16.462	2.586	82.766
NPRL4353	Very poor	very low, but possible	0.479	3.438	9.566	26.790

NPRL4354	Moderate	low	-3.019	15.386	3.490	118.512
NPRL4355	Moderate	low	-2.097	15.976	4.026	109.054
NPRL4356	Good	low	-3.048	12.148	1.678	82.766
NPRL4357	Good	low	-6.303	11.411	1.484	95.321
NPRL4358	Poor	low	-6.289	15.325	3.161	135.812
NPRL4359	Moderate	low	-3.239	25.862	3.311	118.236
NPRL4360	Good	low	-3.822	12.546	2.260	105.701
NPRL4361	Good	low	-1.950	12.239	2.702	82.766
NPRL4362	Good	low	-3.304	11.138	2.305	95.577
NPRL4363	Good	good	-3.653	10.646	0.753	82.766
NPRL4364	Good	very low, but possible	1.315	16.129	3.323	82.766
NPRL4365	Good	low	-2.385	16.602	2.982	82.766
NPRL4366	Good	low	-2.044	28.231	4.219	95.049
NPRL4367	Good	good	-8.763	-2.321	1.251	77.984
NPRL4369	Good	low	-1.088	15.209	2.586	82.766
NPRL4370	Moderate	low	-3.890	16.147	2.671	125.688
NPRL4372	Moderate	low	-4.312	15.112	2.275	125.589
NPRL4373	Moderate	low	-3.292	22.467	1.303	135.537
NPRL4374	Moderate	low	-4.850	16.028	2.275	125.589
NPRL4375	Good	opti mal	-4.642	-0.750	2.018	67.861
NPRL4376	Good	good	-4.075	-0.498	2.395	67.861
NPRL4377	Good	good	-1.365	0.157	4.822	67.861
NPRL4378	Good	good	-3.469	0.756	3.203	94.652
NPRL4379	Moderate	good	-3.768	-0.757	3.101	126.793
NPRL4380	Good	opti mal	-3.570	-1.280	1.911	47.046
NPRL4381	Good	good	-3.264	-0.193	2.260	47.046
NPRL4382	Good	good	-2.165	-0.042	2.637	47.046
NPRL4383	Good	good	-0.825	0.657	3.239	47.046
NPRL4384	Good	low	0.581	0.925	5.064	47.046
NPRL4385	Good	good	-1.916	2.032	3.445	73.836
NPRL4386	Very poor	low	-0.667	-21.522	3.065	154.217
NPRL4387	Good	low	-2.180	1.126	3.139	76.232
NPRL4388	Good	very low, but possible	-0.550	0.893	4.059	55.417
NPRL4389	Very poor	very low, but possible	-1.398	-3.214	15.076	62.249
NPRL4391	Good	opti mal	-5.316	-7.475	0.063	52.461
NPRL4392	Very poor	very low, but possible	1.877	2.644	8.476	17.860
NPRL4393	Good	good	-7.634	1.577	1.365	78.543
NPRL4394	Very poor	very low, but possible	1.156	0.249	7.637	34.601
NPRL4395	Very poor	very low, but possible	1.224	1.835	8.433	34.601
NPRL4396	Very poor	very low, but possible	1.465	2.922	8.802	34.601
NPRL4397	Good	good	0.798	1.007	4.466	41.631
NPRL4398	Very poor	low	1.274	2.029	7.659	41.631
NPRL4399	Good	opti mal	-6.433	-6.675	0.375	43.531
NPRL4400	Good	good	0.740	1.313	4.268	0.000
NPRL4401	Very poor	very low, but possible	1.700	2.660	7.349	0.000
NPRL4402	Moderate	good	-4.930	33.686	1.415	133.327
NPRL4403	Good	low	-1.537	30.273	3.104	91.696
NPRL4404	Good	good	-2.846	22.693	1.714	100.067
NPRL4405	Good	good	-14.091	0.495	2.439	100.067
NPRL4406	Good	good	0.160	6.192	2.308	54.792
NPRL4407	Good	good	-2.126	6.378	2.308	54.792
NPRL4408	Good	good	-2.449	7.189	2.308	54.792
NPRL4409	Good	low	-0.935	2.890	3.305	43.337
NPRL4410	Good	low	-0.895	2.435	3.288	52.267
NPRL4411	Good	good	-7.961	3.313	2.776	33.463
NPRL4413	Good	low	-4.725	3.034	3.138	64.550
NPRL4414	Good	low	-1.983	5.756	3.451	55.620
NPRL4415	Good	low	-0.965	8.389	3.912	55.620
NPRL4416	Good	low	-1.125	8.458	4.368	55.620
NPRL4417	Good	low	-1.199	7.234	4.620	55.620
NPRL4418	Good	good	-3.880	2.337	2.432	51.851
NPRL4419	Good	good	-6.010	0.082	2.282	64.133
NPRL4420	Good	low	-3.378	7.119	3.256	70.127
NPRL4421	Good	low	-1.977	5.717	3.272	61.197
NPRL4422	Good	good	-2.251	4.179	2.432	51.851
NPRL4423	Good	good	-2.259	2.442	2.415	60.781
NPRL4424	Good	low	-2.800	5.801	3.096	51.851
NPRL4425	Good	good	-1.875	4.070	2.415	60.781
NPRL4426	Good	good	-2.883	3.900	2.415	60.781
NPRL4427	Good	good	-3.431	3.864	2.415	60.781
NPRL4428	Good	good	-2.690	3.083	2.415	60.781
NPRL4429	Good	low	2.483	7.733	3.953	52.267
NPRL4430	Good	good	-5.278	-3.183	1.929	47.155
NPRL4431	Very poor	good	-6.501	-4.792	1.128	150.069
NPRL4432	Very poor	low	-5.142	-4.099	3.509	155.484
NPRL4433	Good	good	-1.290	1.045	1.877	66.678

NPRL4434	Very poor	low	-6.543	-1.567	3.924	178.419
NPRL4435	Good	low	-0.293	4.332	3.831	32.076
NPRL4436	Very poor	low	-5.301	-4.382	3.053	155.484
NPRL4437	Good	low	0.783	4.192	3.160	21.740
NPRL4438	Good	good	-4.215	2.039	2.185	53.021
NPRL4439	Good	low	1.085	5.185	3.886	21.740
NPRL4440	Good	good	2.356	6.409	2.905	30.670
NPRL4441	Good	good	-6.279	-0.861	1.039	79.561
NPRL4443	Moderate	Extremely low	-1.204	4.042	6.235	34.601
NPRL4444	Good	good	-2.898	4.208	2.086	63.722
NPRL4446	Good	low	-3.342	6.288	3.649	47.046
NPRL4447	Good	low	-1.182	5.483	3.871	38.116
NPRL4448	Good	low	-2.257	5.356	3.649	47.046
NPRL4449	Good	good	-4.068	4.457	3.427	55.976
NPRL4450	Good	good	-1.519	5.761	3.665	38.116
NPRL4451	Good	low	-2.797	6.336	3.342	26.230
NPRL4452	Good	good	-1.312	5.432	3.665	38.116
NPRL4453	Good	good	-4.719	5.957	3.443	47.046
NPRL4454	Good	opti mal	-5.718	-2.386	0.249	91.137
NPRL4455	Good	good	-5.549	-0.122	2.293	58.307
NPRL4456	Good	low	0.233	5.913	4.124	49.936
NPRL4457	Good	opti mal	-3.182	-0.202	-0.212	52.461
NPRL4458	Good	opti mal	-5.466	-1.383	0.322	55.417
NPRL4459	Good	too soluble	-4.952	-6.969	-0.499	76.232
NPRL4460	Good	opti mal	-6.419	0.495	0.453	34.601
NPRL4461	Moderate	opti mal	-3.984	-3.252	-0.111	34.601
NPRL4463	Poor	too soluble	-4.251	-7.573	-1.887	57.123
NPRL4464	Good	low	2.669	5.406	3.205	66.678
NPRL4465	Good	low	0.852	5.997	4.573	67.237
NPRL4466	Good	good	0.591	6.062	2.525	75.608
NPRL4468	Good	low	3.673	7.927	4.795	58.307
NPRL4469	Good	low	4.620	7.722	2.746	66.678
NPRL4471	Good	low	-0.990	5.970	4.114	67.237
NPRL4472	Good	good	-3.104	4.571	1.860	75.608
NPRL4474	Good	low	-2.238	4.735	3.909	67.237
NPRL4475	Good	good	-1.477	6.411	2.525	75.608
NPRL4476	Good	low	-2.764	3.271	3.892	76.167
NPRL4477	Good	good	-3.624	3.815	1.844	84.538
NPRL4480	Good	good	-1.386	5.898	2.066	75.608
NPRL4483	Good	good	0.917	3.854	2.288	66.678
NPRL4484	Good	low	2.455	6.425	2.746	66.678
NPRL4485	Good	opti mal	-8.783	0.999	0.221	52.461
NPRL4486	Good	opti mal	-7.624	-1.069	0.580	47.155
NPRL4487	Good	good	-5.303	-3.237	0.872	73.836
NPRL4488	Good	opti mal	-4.557	-3.788	-0.255	51.902
NPRL4489	Good	opti mal	-5.161	-7.206	-0.569	64.456
NPRL4490	Good	opti mal	-4.993	-4.351	-0.101	51.902
NPRL4491	Very poor	too soluble	-1.861	-4.253	-2.394	69.203
NPRL4492	Very poor	too soluble	-4.627	-8.267	-3.841	117.863
NPRL4493	Good	opti mal	-4.585	-1.556	0.420	52.461
NPRL4494	Good	opti mal	-5.366	-0.460	0.420	52.461
NPRL4495	Good	opti mal	-5.247	-0.282	0.420	52.461
NPRL4496	Good	opti mal	-6.988	1.950	0.178	73.277
NPRL4497	Good	opti mal	-7.249	0.455	0.420	52.461
NPRL4499	Very poor	too soluble	-2.158	-8.619	-2.664	37.805
NPRL4500	Very poor	too soluble	-1.981	-4.974	-2.424	69.203
NPRL4501	Good	opti mal	-6.628	1.570	0.939	34.601
NPRL4502	Good	opti mal	-6.302	-2.615	0.211	55.417
NPRL4503	Good	opti mal	-4.477	0.713	0.244	52.461
NPRL4504	Good	opti mal	-7.350	0.290	0.615	37.954
NPRL4505	Good	opti mal	-6.637	-5.326	-0.031	76.232
NPRL4506	Very poor	opti mal	-3.651	-23.168	-2.421	204.523
NPRL4507	Very poor	too soluble	0.654	-5.567	-3.343	69.203
NPRL4508	Good	opti mal	-6.660	-4.580	0.211	55.417
NPRL4510	Moderate	good	-5.520	-2.063	0.178	128.694
NPRL4513	Good	low	-4.670	6.588	3.622	26.230
NPRL4515	Good	low	-4.222	0.498	3.694	58.931
NPRL4518	Moderate	very low, but possible	-2.398	26.518	6.319	12.282
NPRL4519	Good	low	0.045	-1.024	3.191	85.000
NPRL4520	Poor	low	-8.681	2.050	3.869	142.538
NPRL4521	Very poor	good	-5.148	-51.845	-2.050	205.507
NPRL4522	Poor	good	-3.685	-0.715	4.926	123.431
NPRL4524	Very poor	Extremely low	-8.330	-22.533	-1.198	383.673
NPRL4526	Poor	too soluble	-4.327	-11.361	-2.063	105.978
NPRL4527	Good	good	-5.146	2.267	2.542	34.601
NPRL4528	Good	good	-2.787	1.117	2.448	51.902

NPRL4529	Good	good	-3.606	1.914	1.870	55.417
NPRL4530	Good	low	-5.891	1.719	2.588	120.247
NPRL4531	Good	good	-2.999	2.220	2.249	60.832
NPRL4532	Good	good	-4.651	0.338	0.844	69.203
NPRL4533	Good	good	1.354	3.963	2.768	17.860
NPRL4534	Very poor	very low, but possible	-2.340	1.462	7.476	96.552
NPRL4535	Good	low	0.673	3.951	4.258	52.461
NPRL4536	Good	low	-3.248	2.927	2.855	45.862
NPRL4537	Good	good	-0.587	1.061	2.098	43.531
NPRL4538	Good	good	-1.813	1.274	2.127	76.232
NPRL4539	Good	good	-4.397	2.441	2.336	63.722
NPRL4540	Good	good	-1.736	1.297	2.168	69.203
NPRL4541	Good	good	-3.098	2.168	2.355	55.417
NPRL4542	Good	good	-1.417	1.280	0.990	34.601
NPRL4543	Good	good	-3.020	0.940	2.260	34.601
NPRL4544	Good	good	-5.184	-0.055	2.053	63.722
NPRL4545	Good	low	-4.125	1.146	2.562	73.642
NPRL4546	Good	low	-4.483	1.064	2.444	90.943
NPRL4547	Good	low	-3.506	-1.030	3.042	64.712
NPRL4548	Good	low	-8.032	3.146	3.478	107.535
NPRL4549	Good	low	-2.897	0.499	2.911	73.642
NPRL4550	Good	low	-3.025	2.520	3.570	73.642
NPRL4551	Good	low	-2.971	2.435	3.754	73.642
NPRL4552	Good	low	-3.502	3.019	3.579	64.712
NPRL4553	Good	low	-2.821	1.096	3.499	64.712
NPRL4554	Good	low	-3.295	2.182	3.500	64.712
NPRL4555	Good	low	-3.469	2.471	3.466	64.712
NPRL4556	Good	low	-1.601	0.956	2.918	26.230
NPRL4557	Good	low	-2.022	2.389	2.734	60.832
NPRL4558	Good	good	-2.602	1.927	2.428	51.902
NPRL4559	Good	low	-3.494	2.415	2.886	87.063
NPRL4560	Good	low	-4.100	-1.158	4.078	49.979
NPRL4561	Good	good	-5.875	0.677	2.231	51.210
NPRL4562	Good	good	-8.312	0.897	2.243	89.978
NPRL4564	Good	low	-1.930	3.155	4.361	25.999
NPRL4565	Good	low	-0.876	2.426	4.865	43.462
NPRL4566	Good	low	-0.435	6.206	4.626	3.352
NPRL4567	Good	low	1.304	0.265	3.756	6.556
NPRL4568	Good	opti mal	-4.936	-2.894	0.645	60.832
NPRL4569	Very poor	opti mal	-5.020	-5.792	-2.456	158.440
NPRL4572	Good	low	-3.670	0.240	3.502	87.622
NPRL4573	Moderate	low	-4.882	0.899	3.692	110.834
NPRL4574	Good	low	-4.537	0.655	2.921	113.294
NPRL4575	Good	low	-3.970	0.207	2.903	97.048
NPRL4577	Very poor	low	-5.845	-2.320	0.810	183.889
NPRL4578	Good	good	-2.934	-1.976	1.716	87.622
NPRL4581	Good	low	-3.013	3.348	3.743	73.836
NPRL4583	Very poor	opti mal	-12.022	-10.254	-0.476	271.507
NPRL4584	Good	good	-7.447	-1.646	1.425	64.347
NPRL4589	Very poor	low	-7.907	11.570	4.944	151.918
NPRL4590	Very poor	low	-8.530	5.521	6.087	185.544
NPRL4591	Very poor	low	-5.772	-3.295	2.133	160.233
NPRL4592	Very poor	low	-7.619	9.723	3.378	164.729
NPRL4593	Good	good	-3.336	9.597	0.931	85.970
NPRL4594	Good	good	-3.950	17.144	0.693	117.368
NPRL4595	Poor	low	-2.900	15.920	3.036	139.108
NPRL4596	Very poor	very low, but possible	-2.278	8.291	3.464	154.163
NPRL4597	Poor	low	-5.050	15.483	4.753	146.503
NPRL4598	Very poor	low	-5.528	7.089	4.354	193.549
NPRL4599	Very poor	low	-6.467	12.727	4.838	151.918
NPRL4600	Very poor	low	-4.054	2.451	2.730	178.235
NPRL4601	Very poor	low	-6.146	8.208	3.609	151.918
NPRL4602	Very poor	very low, but possible	-5.705	1.695	5.131	166.973
NPRL4603	Very poor	low	-5.002	12.100	4.374	151.918
NPRL4604	Very poor	low	-5.664	10.536	4.858	151.918
NPRL4605	Very poor	low	-4.583	7.843	3.110	175.344
NPRL4607	Good	good	1.200	7.048	2.117	66.678
NPRL4608	Very poor	good	-10.430	-11.814	-0.313	258.780
NPRL4609	Very poor	good	-9.539	-12.709	-0.358	258.780
NPRL4610	Very poor	good	-10.430	-11.157	0.778	237.964
NPRL4611	Very poor	good	-8.755	-17.266	1.110	237.964
NPRL4612	Very poor	good	-9.647	-16.371	1.156	237.964
NPRL4613	Very poor	good	-8.755	-17.936	0.008	258.780
NPRL4614	Very poor	very low, but possible	0.359	4.674	4.683	183.889
NPRL4615	Very poor	very low, but possible	-0.049	10.743	3.754	192.259
NPRL4616	Very poor	Extremely low	-1.137	11.403	5.072	260.121

NPRL4617	Very poor	Extremely low	-7.429	-8.950	-0.855	443.673
NPRL4618	Very poor	Extremely low	-9.207	-11.973	-0.324	377.425
NPRL4619	Good	low	-1.463	2.325	2.442	69.203
NPRL4620	Good	low	-2.571	3.459	2.702	51.902
NPRL4621	Good	low	-1.721	3.650	2.928	69.203
NPRL4622	Good	low	-1.827	2.241	2.548	51.902
NPRL4623	Very poor	good	-7.561	49.084	0.935	160.118
NPRL4624	Moderate	low	0.265	12.845	3.757	117.368
NPRL4625	Good	low	-5.340	3.107	3.287	82.207
NPRL4626	Good	low	-6.735	1.003	2.671	104.923
NPRL4628	Good	good	-1.472	-18.298	2.358	77.554
NPRL4629	Good	good	-4.123	-13.072	0.641	76.995
NPRL4630	Good	opti mal	-6.430	-13.530	-0.927	86.543
NPRL4631	Good	good	-3.440	-17.178	0.430	91.902
NPRL4632	Good	good	-2.925	-12.107	0.831	89.971
NPRL4633	Good	opti mal	-4.428	-20.336	-0.794	82.972
NPRL4634	Good	opti mal	-4.315	-15.758	-0.117	103.787
NPRL4635	Good	good	-3.751	-10.887	0.235	112.717
NPRL4636	Good	opti mal	-4.378	-12.109	0.108	77.247
NPRL4637	Poor	opti mal	-2.516	-20.930	-1.525	136.738
NPRL4638	Good	opti mal	-6.966	-13.842	-0.648	107.358
NPRL4639	Moderate	opti mal	-4.803	-18.705	-0.911	124.853
NPRL 5781	Poor	low	-10.027	-15.222	4.513	144.314
NPRL 5782	Very poor	good	-13.213	-18.085	3.922	187.137
NPRL 5783	Very poor	good	-11.254	-20.174	2.877	155.575
NPRL 5784	Poor	low	-8.843	-16.592	4.935	144.314
NPRL 5785	Poor	low	-4.158	-17.749	3.021	135.384
NPRL 5786	Poor	low	-7.681	-11.712	4.735	135.384
NPRL 5787	Poor	good	-7.918	-17.005	3.098	146.645
NPRL 5788	Very poor	low	-10.657	-15.188	4.143	178.207
NPRL 5789	Poor	low	-6.736	-14.640	4.455	135.384
NPRL 5790	Poor	low	-6.497	-13.423	5.157	135.384
NPRL 5791	Good	low	-2.489	-19.412	2.573	89.459
NPRL 5792	Good	low	-2.634	-19.259	3.032	89.459
NPRL 5793	Good	low	-5.650	-22.322	3.116	89.459
NPRL 5794	Good	low	-4.240	-22.091	2.368	89.459
NPRL 5795	Good	low	-4.883	-20.436	2.854	89.459
NPRL 5796	Good	low	-4.603	-21.704	2.854	89.459
NPRL 5797	Good	low	-4.833	-22.684	2.352	98.389
NPRL 5798	Good	low	-4.376	-21.868	2.352	98.389
NPRL 5799	Good	low	-3.286	-19.537	3.032	89.459
NPRL 5800	Good	low	-4.259	-20.727	3.928	98.389
NPRL 5801	Good	low	-3.204	-22.245	3.276	89.459
NPRL 5802	Good	low	-2.378	-19.152	3.411	102.269
NPRL 5803	Good	low	-4.758	-21.449	3.686	102.269
NPRL 5804	Good	low	-3.125	-20.112	3.156	102.269
NPRL 5805	Good	low	-3.725	-20.587	2.700	102.269
NPRL 5806	Good	low	-4.027	-20.563	3.019	102.269
NPRL 5807	Very poor	low	-12.283	-4.402	4.117	157.322
NPRL 5808	Very poor	good	-6.542	-12.070	2.966	161.844
NPRL 5809	Very poor	low	-10.819	-10.257	5.060	157.322
NPRL 5810	Very poor	good	-4.060	-17.502	3.909	161.844
NPRL 5811	Very poor	good	-5.780	-24.384	3.461	182.660
NPRL 5814	Good	low	-7.963	2.223	2.964	47.031
NPRL 5815	Good	low	-7.078	-0.127	1.499	67.536
NPRL 5816	Good	low	-3.139	6.944	3.050	110.012
NPRL 5817	Good	low	-4.291	7.990	1.958	110.012
NPRL 5818	Very poor	good	-11.040	-11.853	1.440	160.674
NPRL 5819	Very poor	low	-15.231	-3.460	3.947	157.322
NPRL 5820	Poor	low	-14.327	-5.897	3.843	148.392
NPRL 5821	Very poor	low	-10.961	-9.809	2.723	157.322
NPRL 5822	Very poor	low	-10.305	-12.644	3.178	157.322
NPRL 5823	Very poor	low	-12.829	-9.017	3.409	166.252
NPRL 5824	Very poor	low	-10.947	-12.252	2.198	157.322
NPRL 5825	Very poor	low	-14.669	-6.104	4.160	157.322
NPRL 5826	Very poor	low	-14.367	-5.872	4.481	157.322
NPRL 5827	Very poor	low	-13.640	-5.687	4.938	157.322
NPRL 5828	Very poor	low	-11.662	-16.031	4.612	178.137
NPRL 5829	Very poor	low	-13.040	-8.180	3.670	178.137
NPRL 5830	Very poor	opti mal	-13.881	-36.208	4.291	190.109
NPRL 5831	Very poor	good	-13.261	-16.242	3.880	209.784
NPRL 5832	Very poor	good	-16.771	-8.962	3.039	182.017
NPRL 5833	Very poor	good	-14.499	-8.475	2.083	166.252
NPRL 5834	Very poor	good	-13.915	-9.143	2.567	166.252
NPRL 5835	Very poor	low	-13.680	-8.760	3.410	166.252
NPRL 5836	Very poor	low	-14.158	-8.854	3.799	166.252

NPRL 5837	Very poor	low	-14.144	-9.395	3.866	166.252
NPRL 5838	Very poor	low	-14.307	-8.617	4.123	166.252
NPRL 5839	Very poor	good	-12.449	-15.425	0.020	166.252
NPRL 5840	Very poor	good	-11.310	-16.096	1.349	166.252
NPRL 5841	Very poor	good	-15.108	-11.652	1.580	175.182
NPRL 5842	Very poor	good	-12.105	-8.433	1.802	166.252
NPRL 5843	Very poor	good	-12.250	-7.759	2.261	166.252
NPRL 5844	Very poor	good	-18.053	-12.971	1.491	209.075
NPRL 5845	Very poor	good	-6.776	-14.675	3.570	166.811
NPRL 5846	Very poor	good	-16.556	-14.283	0.446	177.513
NPRL 5847	Very poor	good	-12.347	-13.455	0.894	166.252
NPRL 5848	Very poor	good	-12.536	-9.457	2.008	166.252
NPRL 5864	Very poor	good	-14.092	-8.742	1.033	177.827
NPRL 5865	Very poor	good	-14.039	-9.338	1.517	177.827
NPRL 5866	Very poor	good	-13.538	-9.233	2.360	177.827
NPRL 5867	Very poor	opti mal	-12.871	-15.865	-1.030	177.827
NPRL 5868	Very poor	opti mal	-12.971	-15.772	-0.681	177.827
NPRL 5869	Very poor	opti mal	-11.486	-15.921	0.298	177.827
NPRL 5870	Very poor	opti mal	-14.717	-11.384	0.530	186.757
NPRL 5871	Very poor	good	-11.790	-8.200	1.211	177.827
NPRL 5872	Very poor	good	-12.282	-8.873	0.752	177.827
NPRL 5873	Very poor	opti mal	-17.663	-12.796	0.441	220.650
NPRL 5874	Very poor	good	-13.930	-9.136	2.748	177.827
NPRL 5875	Very poor	good	-13.916	-9.678	2.816	177.827
NPRL 5876	Very poor	good	-14.078	-8.900	3.073	177.827
NPRL 5877	Very poor	good	-6.634	-15.196	2.519	178.386
NPRL 5878	Very poor	good	-12.484	-11.394	1.455	177.827
NPRL 5879	Very poor	good	-12.523	-13.895	-0.156	177.827
NPRL 5880	Very poor	good	-14.465	-9.630	1.611	177.827
NPRL 5881	Very poor	opti mal	-14.529	-16.523	-0.821	177.827
NPRL 5882	Very poor	good	-15.316	-9.760	1.242	177.827
NPRL 5883	Very poor	opti mal	-15.994	-12.402	0.739	186.757
NPRL 5884	Very poor	good	-12.976	-9.718	0.961	177.827
NPRL 5885	Poor	low	-14.225	-5.913	2.934	135.582
NPRL 5886	Poor	low	-8.067	-12.817	3.424	131.091
NPRL 5887	Very poor	low	-14.825	1.348	3.329	152.882
NPRL 5890	Moderate	very low, but possible	-3.181	-9.702	5.301	88.170
NPRL 5891	Moderate	very low, but possible	-4.638	-9.480	5.285	97.100
NPRL 5892	Moderate	very low, but possible	-5.578	-10.573	5.285	97.100
NPRL 5893	Poor	very low, but possible	-2.693	-12.727	5.268	106.030
NPRL 5894	Poor	very low, but possible	-5.356	-10.370	5.268	106.030
NPRL 5895	Poor	very low, but possible	-4.613	-11.838	5.252	114.960
NPRL 5896	Poor	very low, but possible	-3.693	-10.575	6.274	88.170
NPRL 5897	Poor	very low, but possible	-5.820	-8.972	5.069	106.030
NPRL 5898	Poor	very low, but possible	-5.105	-10.629	5.087	106.030
NPRL 5899	Moderate	very low, but possible	-4.932	-8.745	5.359	97.100
NPRL 5900	Poor	very low, but possible	-3.409	-11.387	6.243	88.170
NPRL 5901	Poor	very low, but possible	-1.746	-8.197	5.965	88.170
NPRL 5902	Poor	Extremely low	-0.811	-7.441	6.630	88.170
NPRL 5903	Poor	Extremely low	-1.927	-8.805	6.630	88.170
NPRL 5904	Poor	very low, but possible	-4.575	-11.863	5.180	111.105
NPRL 5905	Moderate	very low, but possible	-4.154	-10.830	5.463	91.522
NPRL 5906	Poor	very low, but possible	0.814	-9.822	6.243	88.170
NPRL 5907	Poor	very low, but possible	0.490	-8.291	6.243	88.170
NPRL 5908	Very poor	Extremely low	-6.684	-8.687	7.421	97.100
NPRL 5909	Moderate	very low, but possible	-3.352	-12.713	5.712	88.170
NPRL 5910	Moderate	very low, but possible	-3.686	-13.244	5.712	88.170
NPRL 5911	Poor	very low, but possible	-3.295	-13.130	5.918	88.170
NPRL 5912	Poor	very low, but possible	-4.656	-11.685	5.490	97.100
NPRL 5913	Good	good	-7.806	-0.436	1.008	70.838
NPRL 5916	Moderate	low	-11.865	-10.919	2.786	124.061
NPRL 5917	Moderate	low	-9.525	-11.204	2.505	124.061
NPRL 5918	Moderate	low	-9.616	-10.377	2.964	124.061
NPRL 5919	Moderate	low	-10.315	-12.174	3.494	124.061
NPRL 5920	Moderate	low	-10.965	-11.634	3.242	124.061
NPRL 5921	Very poor	low	-15.197	-14.521	2.194	166.884
NPRL 5922	Moderate	low	-12.543	-14.152	2.283	132.991
NPRL 5923	Good	low	-12.250	-14.208	2.300	124.061
NPRL 5924	Good	low	-8.168	-18.188	2.136	124.061
NPRL 5925	Moderate	good	-14.559	-18.950	1.149	135.322
NPRL 5926	Moderate	low	-10.577	-14.057	3.208	124.061
NPRL 5927	Moderate	low	-7.766	-19.827	2.307	124.061
NPRL 5928	Good	low	-8.262	-21.013	2.052	124.061
NPRL 5929	Moderate	good	-9.540	-20.389	0.314	127.413
NPRL 5930	Poor	low	-11.504	-15.109	2.267	141.921
NPRL 5931	Moderate	low	-9.902	-13.764	2.711	124.061

NPRL 5932	Very poor	low	-11.340	-12.834	4.854	157.322
NPRL 5933	Good	low	-2.530	-18.821	2.472	106.888
NPRL 5934	Moderate	very low, but possible	-4.197	-16.479	3.967	119.698
NPRL 5935	Moderate	low	-4.885	-20.858	3.584	119.698
NPRL 5936	Poor	low	-5.536	-13.821	3.068	136.999
NPRL 5937	Very poor	low	-13.886	-6.383	3.912	157.322
NPRL 5938	Good	low	-0.273	-14.746	3.240	95.565
NPRL 5939	Good	low	-0.614	-13.710	3.699	95.565
NPRL 5940	Good	low	-2.712	-16.921	3.783	95.565
NPRL 5941	Good	low	-1.279	-13.390	3.699	95.565
NPRL 5942	Good	low	0.192	-13.439	3.699	95.565
NPRL 5943	Good	low	-1.584	-16.388	3.034	95.565
NPRL 5944	Good	low	-3.757	-16.319	3.018	104.495
NPRL 5945	Good	low	-2.485	-15.376	3.520	95.565
NPRL 5946	Good	low	-2.548	-15.264	3.018	104.495
NPRL 5947	Good	very low, but possible	-1.298	-17.077	3.943	95.565
NPRL 5948	Good	good	-2.409	-21.575	1.533	82.754
NPRL 5949	Good	low	-2.900	-19.494	2.423	106.825
NPRL 5950	Good	low	-0.114	-19.880	2.633	86.107
NPRL 5951	Good	good	-4.053	-25.017	0.616	85.958
NPRL 5952	Good	good	-4.189	-26.173	0.112	89.311
NPRL 5953	Good	good	-3.337	-20.299	0.640	116.380
NPRL 5954	Good	good	-3.226	-23.196	1.607	85.958
NPRL 5955	Good	good	-4.415	-23.760	0.822	82.754
NPRL 5956	Good	low	-2.683	-22.644	1.807	82.754
NPRL 5957	Very poor	low	-13.125	-8.974	2.415	153.442
NPRL 5958	Poor	low	-12.797	-8.287	3.972	122.771
NPRL 5959	Moderate	low	-11.832	-6.198	2.654	135.582
NPRL 5960	Poor	low	-11.977	-5.371	3.113	135.582
NPRL 5961	Moderate	good	-12.684	-11.910	0.463	138.934
NPRL 5962	Poor	low	-14.320	-8.929	2.432	144.512
NPRL 5963	Moderate	low	-10.920	-11.507	2.285	135.582
NPRL 5964	Moderate	low	-12.059	-13.385	1.220	135.582
NPRL 5965	Moderate	low	-13.530	-12.649	0.872	135.582
NPRL 5966	Poor	low	-12.263	-7.241	2.859	135.582
NPRL 5967	Moderate	low	-14.028	-8.842	2.448	135.582
NPRL 5968	Poor	low	-14.537	-9.259	2.916	144.512
NPRL 5969	Poor	low	-12.102	-6.529	3.138	135.582
NPRL 5970	Moderate	low	-12.369	-16.577	2.032	135.582
NPRL 5971	Poor	low	-12.526	-8.973	3.465	135.582
NPRL 5972	Very poor	very low, but possible	-13.630	-16.740	4.987	150.637
NPRL 5973	Very poor	very low, but possible	-11.940	-24.131	3.554	150.637
NPRL 5974	Poor	very low, but possible	-14.034	-7.873	4.694	135.582
NPRL 5975	Poor	low	-12.526	-14.783	3.261	135.582
NPRL 5976	Poor	low	-14.519	-9.079	3.759	144.512
NPRL 5977	Poor	very low, but possible	-10.070	-10.619	3.612	135.582
NPRL 5978	Moderate	low	-10.878	-13.196	2.548	135.582
NPRL 5979	Poor	very low, but possible	-11.705	-6.077	3.981	135.582
NPRL 5980	Good	low	-7.793	-11.434	2.169	118.281
NPRL 5981	Moderate	very low, but possible	-7.243	-11.864	3.496	118.281
NPRL 5982	Poor	very low, but possible	-8.250	-21.657	4.503	133.336
NPRL 5983	Very poor	low	-14.131	-12.516	1.267	152.882
NPRL 5984	Very poor	low	-12.084	-12.791	2.595	152.882
NPRL 5985	Very poor	low	-14.628	-3.752	2.843	152.882
NPRL 5986	Very poor	low	-12.485	-0.748	3.049	152.882
NPRL 5988	Very poor	low	-12.577	1.120	3.508	152.882
NPRL 5989	Very poor	low	-11.520	-10.867	2.680	152.882
NPRL 5990	Very poor	low	-13.302	-13.391	1.751	152.882
NPRL 5991	Very poor	low	-12.702	-1.425	3.533	152.882
NPRL 5992	Very poor	low	-15.096	0.345	3.814	152.882
NPRL 5993	Very poor	low	-11.154	-11.696	3.164	152.882
NPRL 5994	Very poor	low	-12.342	-11.959	2.594	152.882
NPRL 5995	Very poor	very low, but possible	-12.932	-0.973	4.376	152.882
NPRL 5996	Very poor	very low, but possible	-10.670	-10.966	4.007	152.882
NPRL 5997	Very poor	low	-11.980	-13.482	3.491	152.882
NPRL 5998	Very poor	low	-13.346	-3.763	3.860	152.882
NPRL 5999	Poor	low	-9.189	-11.464	3.911	131.091
NPRL 6000	Poor	very low, but possible	-6.549	-10.240	4.089	131.091
NPRL 6001	Poor	very low, but possible	-7.200	-10.433	4.089	131.091
NPRL 6002	Poor	low	-5.978	-10.220	3.630	131.091
NPRL 6003	Poor	low	-6.374	-9.494	3.630	131.091
NPRL 6004	Poor	low	-6.724	-12.473	3.630	131.091
NPRL 6005	Poor	very low, but possible	-9.564	-13.315	4.173	131.091
NPRL 6006	Poor	very low, but possible	-9.956	-14.011	4.003	131.091
NPRL 6007	Poor	very low, but possible	-7.915	-13.875	4.333	131.091
NPRL 6008	Poor	low	-9.802	-14.186	3.408	140.021

NPRL 6009	Moderate	low	-8.599	-11.457	3.150	131.091
NPRL 6010	Poor	low	-9.461	-19.724	2.820	143.645
NPRL 6011	Poor	low	-7.063	-15.879	3.459	131.091
NPRL 6012	Poor	low	-9.596	-13.416	3.886	131.091
NPRL 6013	Poor	low	-9.311	-13.416	3.430	131.091
NPRL 6014	Moderate	low	-8.789	-14.243	1.760	131.091
NPRL 6015	Poor	low	-9.337	-15.905	2.274	142.352
NPRL 6016	Poor	very low, but possible	-7.915	-14.717	4.817	131.091
NPRL 6017	Poor	very low, but possible	-9.189	-12.148	4.395	131.091
NPRL 6018	Poor	very low, but possible	-5.978	-10.905	4.114	131.091
NPRL 6019	Poor	very low, but possible	-9.064	-11.590	4.477	131.091
NPRL 6020	Poor	very low, but possible	-6.356	-11.120	4.957	131.091
NPRL 6021	Poor	very low, but possible	-10.898	-14.738	5.124	140.021
NPRL 6022	Poor	very low, but possible	-6.738	-11.181	5.346	131.091
NPRL 6023	Poor	very low, but possible	-7.134	-10.454	5.346	131.091
NPRL 6024	Poor	very low, but possible	-7.484	-13.433	5.346	131.091
NPRL 6025	Poor	low	-9.944	-17.033	4.057	142.352
NPRL 6026	Poor	very low, but possible	-6.724	-11.723	5.413	131.091
NPRL 6027	Poor	very low, but possible	-10.746	-14.373	5.448	140.021
NPRL 6028	Poor	very low, but possible	-7.212	-10.816	5.670	131.091
NPRL 6029	Poor	very low, but possible	-10.564	-14.765	6.043	131.091
NPRL 6030	Poor	very low, but possible	-7.211	-13.212	4.441	131.091
NPRL 6031	Poor	low	-10.719	-16.686	4.220	140.021
NPRL 6032	Poor	very low, but possible	-9.919	-15.081	3.962	131.091
NPRL 6033	Poor	low	-9.484	-18.508	3.085	142.352
NPRL 6034	Very poor	very low, but possible	-10.837	-21.759	5.742	155.076
NPRL 6035	Poor	very low, but possible	-7.303	-18.202	5.963	146.146
NPRL 6036	Poor	very low, but possible	-10.012	-19.530	5.484	146.146
NPRL 6037	Poor	very low, but possible	-7.328	-10.310	4.337	131.091
NPRL 6038	Poor	low	-6.757	-10.290	3.878	131.091
NPRL 6039	Poor	low	-9.465	-11.527	3.398	131.091
NPRL 6040	Poor	low	-7.552	-15.677	3.707	131.091
NPRL 6041	Poor	low	-6.456	-9.901	4.199	131.091
NPRL 6042	Poor	low	-9.020	-13.713	4.902	131.091
NPRL 6043	Poor	low	-5.728	-9.579	4.655	131.091
NPRL 6044	Poor	very low, but possible	-7.568	-9.950	5.114	131.091
NPRL 6045	Poor	low	-6.452	9.326	2.611	140.033
NPRL 6046	Poor	low	-5.393	8.598	3.940	140.033
NPRL 6047	Poor	low	-9.013	12.218	4.188	140.033
NPRL 6048	Poor	low	-9.331	14.969	4.674	140.033
NPRL 6049	Poor	low	-9.173	12.225	4.171	148.963
NPRL 6050	Poor	low	-6.636	14.981	4.393	140.033
NPRL 6051	Poor	very low, but possible	-6.781	15.639	4.852	140.033
NPRL 6052	Very poor	low	-10.003	9.599	3.037	151.294
NPRL 6053	Poor	low	-5.406	10.866	4.024	140.033
NPRL 6054	Poor	low	-6.323	9.956	2.202	143.386
NPRL 6055	Moderate	low	-6.066	9.177	2.628	131.103
NPRL 6056	Poor	low	-8.393	11.537	4.204	131.103
NPRL 6057	Poor	low	-5.021	10.580	4.041	131.103
NPRL 6058	Poor	very low, but possible	-7.734	13.377	5.096	140.033
NPRL 6059	Very poor	low	-12.717	12.086	4.082	182.856
NPRL 6060	Poor	very low, but possible	-6.251	14.695	4.410	131.103
NPRL 6062	Good	opti mal	-3.916	-4.454	0.832	64.152
NPRL 6063	Very poor	opti mal	-7.581	10.059	7.414	210.553
NPRL 6064	Very poor	good	-10.202	-14.548	1.366	161.844
NPRL 6065	Very poor	good	-11.768	-15.076	1.850	161.844
NPRL 6066	Very poor	low	-10.102	-14.276	2.693	161.844
NPRL 6067	Very poor	good	-11.879	-16.848	1.521	161.696
NPRL 6068	Very poor	good	-11.865	-17.390	1.588	161.696
NPRL 6069	Very poor	good	-11.727	-15.819	1.845	161.696
NPRL 6070	Very poor	good	-8.144	-14.051	2.761	161.844
NPRL 6071	Very poor	good	-9.127	-14.406	3.245	161.844
NPRL 6072	Very poor	low	-8.891	-14.103	4.088	161.844
NPRL 6073	Very poor	low	-9.459	-14.116	4.477	161.844
NPRL 6074	Very poor	low	-8.860	-14.658	4.544	161.844
NPRL 6075	Very poor	low	-9.113	-12.639	4.801	161.844
NPRL 6076	Very poor	low	-6.505	-12.747	7.672	161.844
NPRL 6077	Very poor	low	-7.791	-15.043	6.596	161.696
NPRL 6078	Very poor	low	-7.672	-15.248	7.895	161.696
NPRL 6079	Very poor	low	-8.179	-11.833	9.712	161.844
NPRL 6080	Good	low	-9.777	-16.905	1.955	123.331
NPRL 6081	Good	low	-9.601	-16.351	2.343	123.331
NPRL 6082	Moderate	low	-9.588	-16.893	2.411	123.331
NPRL 6083	Poor	low	-9.829	-13.462	3.898	136.141
NPRL 6084	Good	good	-11.548	-18.209	1.197	123.331
NPRL 6085	Good	low	-8.468	-13.288	2.865	118.840

NPRL 6086	Moderate	low	-8.415	-13.803	3.349	118.840
NPRL 6087	Moderate	very low, but possible	-8.319	-13.561	4.192	118.840
NPRL 6088	Poor	very low, but possible	-9.092	-13.464	4.581	118.840
NPRL 6089	Poor	very low, but possible	-9.078	-14.006	4.648	118.840
NPRL 6090	Poor	very low, but possible	-9.566	-12.435	4.905	118.840
NPRL 6091	Very poor	low	-8.170	-15.087	2.759	161.663
NPRL 6092	Moderate	low	-8.816	-12.949	3.113	118.840
NPRL 6093	Moderate	low	-9.197	-12.736	3.434	118.840
NPRL 6094	Good	good	-2.558	-6.123	1.685	43.337
NPRL 6095	Good	good	-5.087	-2.986	1.641	43.337
NPRL 6097	Good	good	-3.768	-1.278	2.700	55.891
NPRL 6098	Good	good	-1.160	0.399	3.031	43.337
NPRL 6099	Good	good	-6.370	-5.142	1.485	66.053
NPRL 6100	Good	good	-3.703	-2.300	2.154	54.598
NPRL 6101	Poor	very low, but possible	-3.923	12.172	5.263	122.822
NPRL 6102	Poor	very low, but possible	-5.228	13.503	5.749	122.822
NPRL 6103	Poor	very low, but possible	-4.925	11.867	5.246	131.753
NPRL 6104	Poor	very low, but possible	-3.056	15.297	5.927	122.822
NPRL 6105	Poor	very low, but possible	-5.057	11.745	6.011	122.822
NPRL 6106	Poor	very low, but possible	-3.721	15.272	5.927	122.822
NPRL 6107	Poor	very low, but possible	-5.035	13.140	6.235	122.822
NPRL 6108	Poor	very low, but possible	-5.802	13.400	6.721	122.822
NPRL 6109	Poor	very low, but possible	-6.208	12.441	6.219	131.753
NPRL 6110	Poor	very low, but possible	-4.575	15.530	6.900	122.822
NPRL 6111	Poor	very low, but possible	-5.069	15.745	6.900	122.822
NPRL 6112	Poor	very low, but possible	-4.137	12.022	5.246	131.753
NPRL 6113	Poor	very low, but possible	-5.613	12.618	5.733	131.753
NPRL 6114	Poor	low	-5.061	11.413	5.230	140.683
NPRL 6115	Poor	very low, but possible	-3.441	14.413	5.911	131.753
NPRL 6116	Poor	very low, but possible	-3.935	14.627	5.911	131.753
NPRL 6117	Poor	low	-4.885	11.863	5.214	149.613
NPRL 6118	Poor	very low, but possible	-6.060	12.587	5.700	149.613
NPRL 6119	Very poor	low	-6.058	11.164	5.197	158.543
NPRL 6120	Poor	very low, but possible	-4.189	14.253	5.878	149.613
NPRL 6121	Good	low	-6.555	4.861	2.927	70.652
NPRL 6122	Good	low	-4.652	5.041	3.613	31.976
NPRL 6123	Good	very low, but possible	-7.155	6.842	5.322	40.906
NPRL 6124	Good	very low, but possible	-2.415	7.516	5.086	31.976
NPRL 6125	Good	low	-2.271	6.753	3.358	58.766
NPRL 6126	Good	low	-5.344	8.140	3.175	49.836
NPRL 6127	Good	low	-2.127	5.439	3.818	31.976
NPRL 6128	Good	very low, but possible	0.919	9.502	5.292	31.976
NPRL 6129	Good	low	-4.625	4.916	3.338	70.652
NPRL 6130	Good	low	-2.841	4.301	4.024	31.976
NPRL 6131	Good	very low, but possible	-5.482	6.400	5.733	40.906
NPRL 6132	Good	very low, but possible	-0.396	6.141	5.497	31.976
NPRL 6133	Good	low	-4.512	6.300	3.132	70.652
NPRL 6134	Good	low	-7.045	7.061	3.175	49.836
NPRL 6135	Good	low	-3.828	5.382	3.818	31.976
NPRL 6136	Good	very low, but possible	-5.523	8.111	5.527	40.906
NPRL 6137	Good	low	-7.720	6.003	2.937	67.696
NPRL 6138	Good	low	-1.206	7.284	3.374	49.836
NPRL 6139	Good	low	-2.274	4.750	3.580	49.836
NPRL 6140	Good	low	-0.356	7.305	4.111	49.836
NPRL 6141	Good	low	-6.698	5.080	3.383	70.652
NPRL 6142	Good	low	-6.101	3.710	3.609	58.766
NPRL 6143	Good	low	-9.174	4.756	3.426	49.836
NPRL 6144	Good	low	-3.598	7.127	3.863	31.976
NPRL 6145	Good	low	-4.945	5.817	3.589	70.652
NPRL 6146	Good	low	-4.371	4.447	3.814	58.766
NPRL 6147	Good	low	-6.906	5.910	3.632	49.836
NPRL 6148	Good	low	-3.097	6.262	4.069	31.976
NPRL 6149	Good	low	-7.292	-0.767	3.252	77.357
NPRL 6150	Good	low	-2.693	0.779	3.732	38.681
NPRL 6151	Good	low	-3.762	-1.048	3.938	38.681
NPRL 6152	Good	very low, but possible	-0.940	0.758	4.469	38.681
NPRL 6153	Good	good	-9.218	-3.090	1.290	31.976
NPRL 6154	Good	good	-6.130	-2.354	1.496	31.976
NPRL 6155	Good	good	-4.486	-0.512	1.496	31.976
NPRL 6156	Good	good	-4.703	-2.708	1.701	31.976
NPRL 6157	Good	good	-5.655	-2.356	1.257	49.836
NPRL 6158	Good	good	-5.015	-6.590	1.615	38.681
NPRL 6159	Good	good	-5.320	-3.823	1.746	31.976
NPRL 6160	Good	good	-1.505	-2.612	2.157	31.976
NPRL 6162	Good	low	-4.389	-10.422	3.589	110.275
NPRL 6163	Good	low	-5.447	-10.687	3.589	110.275

NPRL 6164	Moderate	low	-3.663	-9.509	3.767	110.275
NPRL 6165	Moderate	very low, but possible	-3.845	-9.825	4.432	110.275
NPRL 6166	Good	good	-3.375	-16.601	1.780	110.275
NPRL 6167	Moderate	good	-6.367	-19.086	0.874	131.090
NPRL 6168	Moderate	low	-6.568	-11.856	3.087	119.205
NPRL 6169	Moderate	low	-5.222	-12.929	3.087	119.205
NPRL 6170	Moderate	low	-3.423	-15.824	3.087	119.205
NPRL 6171	Moderate	very low, but possible	-2.994	-11.542	4.432	110.275
NPRL 6172	Good	low	-4.454	-11.350	3.103	110.275
NPRL 6173	Good	good	-4.927	-20.609	1.454	110.126
NPRL 6174	Good	low	-3.540	-15.439	3.309	110.275
NPRL 6175	Good	low	-3.236	-10.545	3.309	110.275
NPRL 6176	Good	low	-4.350	-17.147	3.514	110.275
NPRL 6177	Very poor	low	-8.459	-14.873	2.997	153.098
NPRL 6178	Good	good	-5.873	-16.737	1.849	125.330
NPRL 6179	Good	low	-2.889	-15.036	2.491	121.536
NPRL 6180	Moderate	good	-3.834	-15.338	1.845	132.797
NPRL 6181	Moderate	good	-6.545	-18.264	1.341	132.797
NPRL 6182	Moderate	low	-3.166	-16.470	3.434	121.536
NPRL 6183	Poor	low	-12.361	-11.335	2.058	144.876
NPRL 6184	Poor	low	-12.201	-8.440	2.544	144.876
NPRL 6185	Poor	low	-10.390	-8.398	2.263	144.876
NPRL 6186	Very poor	good	-12.312	-10.950	2.041	153.807
NPRL 6187	Poor	low	-11.653	-8.828	3.000	144.876
NPRL 6188	Very poor	good	-14.434	-15.597	0.907	156.137
NPRL 6189	Poor	good	-7.877	-16.954	2.065	144.876
NPRL 6190	Very poor	low	-14.950	-15.114	1.847	230.523
NPRL 6191	Poor	low	-10.238	-10.957	2.469	144.876
NPRL 6192	Poor	low	-11.575	-11.060	2.966	144.876
NPRL 6193	Poor	low	-11.812	-9.368	3.252	144.876
NPRL 6194	Poor	low	-8.450	-15.055	1.894	144.876
NPRL 6196	Good	good	-11.522	-0.988	0.935	95.801
NPRL 6197	Good	low	-8.505	5.978	3.686	92.597
NPRL 6198	Good	low	-5.040	-1.468	1.452	52.978
NPRL 6199	Good	good	-4.346	0.889	1.246	52.978
NPRL 6200	Good	good	-3.585	1.738	1.246	52.978
NPRL 6201	Good	low	-3.767	-0.531	1.913	52.978
NPRL 6202	Good	low	-3.514	1.826	1.707	52.978
NPRL 6203	Good	low	-2.754	2.675	1.707	52.978
NPRL 6204	Good	very low, but possible	-0.015	6.605	3.930	53.126
NPRL 6205	Good	low	0.238	8.915	3.724	53.126
NPRL 6206	Good	low	0.999	9.763	3.724	53.126
NPRL 6207	Good	low	-4.994	-0.653	1.514	52.978
NPRL 6208	Good	good	-4.741	1.704	1.309	52.978
NPRL 6209	Good	good	-3.980	2.553	1.309	52.978
NPRL 6210	Good	low	-3.898	1.124	2.149	52.978
NPRL 6211	Good	low	-3.645	2.866	1.944	52.978
NPRL 6212	Good	low	-2.884	3.715	1.944	52.978
NPRL 6213	Good	very low, but possible	-1.487	5.493	4.203	49.774
NPRL 6214	Good	low	-1.147	7.850	3.997	49.774
NPRL 6215	Good	low	-0.386	8.699	3.997	49.774
NPRL 6216	Good	low	-2.861	1.925	3.314	52.978
NPRL 6217	Good	low	-2.608	3.956	3.109	52.978
NPRL 6218	Good	low	-1.847	4.805	3.109	52.978
NPRL 6266	Good	low	-1.455	4.711	2.684	73.836
NPRL 6267	Good	low	-0.282	3.991	3.180	43.531
NPRL 6268	Very poor	good	-3.994	-9.124	2.404	172.003
NPRL 6269	Good	good	-4.957	-5.082	1.523	82.019
NPRL 6270	Good	good	-0.389	2.751	2.828	82.766
NPRL 6271	Good	good	-4.627	-2.542	1.610	26.316
NPRL 6272	Moderate	too soluble	-4.594	-10.763	-0.970	50.926
NPRL 6273	Good	opti mal	-4.860	-3.571	1.390	58.931
NPRL 6274	Good	opti mal	-5.477	-5.464	0.856	47.046
NPRL 6276	Good	opti mal	-3.605	-2.725	1.348	55.417
NPRL 6277	Good	opti mal	-6.376	-7.041	-0.119	67.861
NPRL 6278	Good	too soluble	-5.764	-7.066	0.132	83.262
NPRL 6279	Good	opti mal	-6.967	-5.947	0.871	47.046
NPRL 6280	Good	good	-1.939	-3.079	2.328	41.631
NPRL 6281	Good	opti mal	-5.254	-4.597	1.288	50.561
NPRL 6283	Good	good	-2.253	3.229	2.151	95.211
NPRL 6284	Good	good	-1.371	0.323	2.167	86.281
NPRL 6285	Good	good	-2.415	0.477	2.123	97.607
NPRL 6286	Good	good	-2.965	-3.579	1.813	117.863
NPRL 6287	Good	low	-3.970	0.207	2.903	97.048
NPRL 6288	Good	good	-4.036	-2.292	1.812	117.863
NPRL 6289	Good	good	-4.509	-1.652	2.068	114.348

NPRL 6291	Very poor	good	-6.939	-17.803	0.760	177.354
NPRL 6292	Very poor	low	-7.008	-20.944	-0.590	257.661
NPRL 6293	Good	good	-3.505	-0.055	2.221	105.978
NPRL 6294	Moderate	too soluble	-5.520	-13.248	-1.565	116.873
NPRL 6295	Very poor	good	-6.194	-9.447	-2.317	242.261
NPRL 6296	Very poor	opti mal	-2.123	-17.061	-1.777	157.098
NPRL 6297	Very poor	too soluble	-5.510	-20.091	-3.199	168.984
NPRL 6298	Poor	opti mal	-4.971	-18.101	-1.981	148.168
NPRL 6299	Good	low	-0.879	-0.491	4.198	52.461
NPRL 6300	Good	opti mal	-9.043	-4.032	0.518	38.675
NPRL 6302	Good	opti mal	-5.107	-4.543	0.174	55.976
NPRL 6303	Good	opti mal	-5.533	-3.344	0.523	55.976
NPRL 6304	Good	opti mal	-4.016	-4.032	0.497	55.976
NPRL 6305	Good	opti mal	-8.856	-2.666	0.362	47.046
NPRL 6306	Very poor	good	-12.089	-18.312	0.895	216.589
NPRL 6307	Very poor	low	-11.787	-20.694	0.669	228.475
NPRL 6309	Very poor	very low, but possible	-4.537	7.585	7.049	41.631
NPRL 6310	Very poor	Extremely low	-3.143	13.022	8.319	17.300
NPRL 6311	Very poor	very low, but possible	-4.463	7.189	7.117	41.631
NPRL 6312	Very poor	Extremely low	-0.274	0.037	7.166	20.815
NPRL 6314	Good	too soluble	-5.951	-5.095	-0.680	100.562
NPRL 6315	Good	opti mal	-3.636	-2.306	1.251	72.717
NPRL 6316	Good	low	-5.618	-0.018	3.170	82.207
NPRL 6317	Good	good	-4.630	-6.158	1.553	55.417
NPRL 6318	Good	good	-2.132	-6.213	2.655	34.601
NPRL 6319	Poor	low	-5.983	-1.544	5.281	140.084
NPRL 6320	Good	good	-4.426	-0.507	1.948	38.116
NPRL 6321	Good	good	-4.287	-0.870	2.605	38.116
NPRL 6322	Good	opti mal	-5.202	-2.130	1.662	38.116
NPRL 6323	Very poor	very low, but possible	-8.243	-1.089	11.119	144.158
NPRL 6324	Very poor	low	-9.363	0.381	4.467	197.675
NPRL 6325	Very poor	very low, but possible	-8.684	0.646	6.131	197.675
NPRL 6326	Poor	low	-2.925	-4.504	4.975	136.283
NPRL 6327	Very poor	good	-5.846	-5.283	-0.275	189.240
NPRL 6328	Poor	very low, but possible	-7.661	-2.665	3.434	138.743
NPRL 6329	Good	very low, but possible	-7.766	-4.857	3.319	112.512
NPRL 6330	Poor	very low, but possible	-6.789	1.637	5.403	124.397
NPRL 6331	Poor	low	-4.866	-5.377	4.898	141.698
NPRL 6332	Poor	low	-5.492	-5.377	5.354	141.698
NPRL 6333	Poor	low	-6.646	-5.089	6.607	105.978
NPRL 6334	Very poor	low	-6.646	-5.430	7.519	105.978
NPRL 6336	Very poor	low	-6.646	-3.640	7.519	105.978
NPRL 6337	Very poor	good	-1.683	-16.838	-1.003	207.100
NPRL 6338	Very poor	good	-3.958	-16.445	-1.109	224.960
NPRL 6339	Very poor	low	-3.175	-24.637	-1.619	245.776
NPRL 6340	Very poor	low	-3.751	-17.006	-1.351	245.776
NPRL 6341	Very poor	low	-2.968	-24.387	-1.861	266.591
NPRL 6342	Very poor	good	-4.588	-19.385	-1.802	245.776
NPRL 6343	Very poor	good	-11.298	-16.436	0.217	190.358
NPRL 6345	Good	too soluble	-6.562	-4.264	-0.170	62.446
NPRL 6347	Good	opti mal	-4.671	-2.562	0.007	73.277
NPRL 6348	Good	opti mal	-3.771	-3.629	-0.973	73.277
NPRL 6349	Very poor	good	-2.936	-15.120	-0.987	229.034
NPRL 6350	Poor	good	-10.482	-13.588	1.559	148.727
NPRL 6351	Very poor	too soluble	-5.111	-20.721	-2.916	151.683
NPRL 6352	Very poor	too soluble	-3.140	-18.957	-3.766	165.469
NPRL 6353	Very poor	too soluble	-2.206	-16.940	-2.621	144.653
NPRL 6354	Very poor	too soluble	-5.044	-19.283	-2.159	139.797
NPRL 6355	Very poor	opti mal	-6.018	-4.957	-0.115	156.539
NPRL 6356	Good	good	-1.681	-6.130	2.294	109.492
NPRL 6357	Good	good	-5.908	-0.763	1.434	111.393
NPRL 6358	Good	good	-5.397	-0.515	1.670	73.277
NPRL 6359	Good	opti mal	-7.110	-3.616	0.639	88.677
NPRL 6360	Good	good	-4.641	-5.720	1.596	58.931
NPRL 6362	Good	good	-4.082	-1.702	1.747	26.230
NPRL 6363	Good	good	-6.682	-2.630	1.200	76.791
NPRL 6364	Good	good	-4.509	-4.170	1.857	38.785
NPRL 6365	Good	good	-3.427	0.024	1.538	107.878
NPRL 6366	Good	opti mal	-4.197	0.080	0.488	51.902
NPRL 6367	Good	good	-2.759	-2.601	3.043	47.046
NPRL 6368	Good	low	-3.795	-0.209	3.026	68.530
NPRL 6369	Good	good	-3.523	-0.611	3.212	38.116
NPRL 6370	Good	good	-3.523	-0.611	3.212	38.116
NPRL 6371	Good	too soluble	-5.092	-8.847	-0.169	83.262
NPRL 6372	Good	good	-2.787	-4.075	1.706	64.347
NPRL 6373	Good	low	-2.576	-1.021	3.125	82.207

NPRL 6374	Good	opti mal	-5.745	-2.206	1.562	41.631
NPRL 6375	Good	low	-6.566	0.972	2.855	38.785
NPRL 6376	Good	low	-2.283	1.008	4.117	47.715
NPRL 6377	Good	low	-2.346	-1.424	2.723	82.207
NPRL 6378	Good	low	-1.780	-0.608	3.748	52.461
NPRL 6379	Good	low	-3.004	-1.483	4.027	61.391
NPRL 6380	Good	low	-2.971	0.484	3.648	55.976
NPRL 6381	Very poor	good	-11.033	-4.411	1.037	181.715
NPRL 6382	Good	low	-5.195	-5.921	3.633	55.976
NPRL 6383	Very poor	good	-9.179	-3.287	1.869	160.900
NPRL 6384	Moderate	low	-7.953	-1.926	2.297	134.669
NPRL 6385	Good	low	-4.925	-2.475	2.793	70.321
NPRL 6386	Good	good	-5.080	-3.097	1.058	90.578
NPRL 6387	Good	good	-5.317	-2.895	1.437	95.993
NPRL 6388	Very poor	good	-9.452	-2.779	1.125	166.315
NPRL 6389	Good	good	-9.165	-1.953	1.024	122.783
NPRL 6390	Good	low	-1.765	1.228	3.649	67.861
NPRL 6391	Good	low	-0.744	4.301	3.518	60.832
NPRL 6392	Very poor	good	-9.419	-3.891	-0.021	193.600
NPRL 6393	Moderate	low	-2.678	2.471	4.394	104.923
NPRL 6394	Moderate	good	-8.295	-4.328	0.823	143.599
NPRL 6395	Good	low	-2.182	-1.259	3.720	55.976
NPRL 6396	Good	low	-3.565	-0.630	3.769	64.906
NPRL 6397	Good	low	-3.565	-0.630	3.769	64.906
NPRL 6398	Good	low	-3.884	0.900	3.696	55.976
NPRL 6399	Very poor	Extremely low	-2.322	0.388	10.617	53.580
NPRL 6400	Good	low	-2.415	1.242	3.577	20.815
NPRL 6401	Moderate	very low, but possible	-0.257	-0.110	6.069	58.931
NPRL 6402	Good	low	-2.860	-0.650	4.432	83.262
NPRL 6403	Good	too soluble	-5.126	-7.514	0.086	38.116
NPRL 6404	Good	too soluble	-6.721	-7.029	-0.930	72.717
NPRL 6405	Good	too soluble	-9.730	-5.077	-0.456	81.648
NPRL 6406	Good	good	-4.141	-2.479	3.497	100.491
NPRL 6407	Moderate	very low, but possible	0.137	2.845	5.725	60.832
NPRL 6408	Moderate	very low, but possible	-2.609	4.722	6.480	41.631
NPRL 6409	Poor	Extremely low	-1.029	0.864	6.846	38.675
NPRL 6410	Good	low	-0.844	2.512	4.882	47.046
NPRL 6411	Moderate	low	-5.912	-1.035	2.467	125.739
NPRL 6412	Good	very low, but possible	-3.833	1.681	5.190	78.692
NPRL 6413	Good	low	-0.056	0.583	2.924	47.046
NPRL 6414	Good	good	-3.750	-1.667	1.710	47.046
NPRL 6415	Good	good	-5.800	-3.649	0.771	55.976
NPRL 6416	Good	good	-1.626	-1.292	2.540	38.116
NPRL 6417	Good	low	-0.215	-0.197	3.736	20.815
NPRL 6419	Good	low	-0.146	-1.269	4.195	41.631
NPRL 6420	Good	low	-0.815	2.356	4.541	20.815
NPRL 6421	Moderate	good	-7.919	-1.276	1.930	134.669
NPRL 6422	Good	low	-5.150	-2.626	3.274	99.508
NPRL 6423	Poor	low	-5.668	-3.719	4.975	108.438
NPRL 6424	Good	good	-2.846	-2.391	2.165	71.376
NPRL 6425	Good	good	-3.854	-3.419	3.094	97.607
NPRL 6426	Good	low	-3.906	-1.275	3.667	76.791
NPRL 6427	Good	low	-5.124	-3.646	4.046	82.207
NPRL 6428	Good	low	0.160	-3.775	2.435	61.120
NPRL 6429	Good	low	1.996	-3.602	3.004	61.120
NPRL 6430	Good	low	-0.287	-3.327	2.184	23.395
NPRL 6431	Good	very low, but possible	3.017	4.807	4.397	23.543
NPRL 6432	Good	low	0.329	-3.387	2.613	23.395
NPRL 6433	Good	low	2.899	2.762	4.197	34.804
NPRL 6434	Good	low	-1.450	-7.852	2.106	91.454
NPRL 6435	Good	low	-2.583	5.193	4.286	23.543
NPRL 6436	Moderate	Extremely low	6.697	14.315	6.599	23.543
NPRL 6437	Good	low	-2.086	5.758	3.510	92.906
NPRL 6438	Good	low	0.872	6.158	3.826	46.065
NPRL 6439	Good	low	-3.895	2.335	2.741	57.448
NPRL 6440	Good	good	-4.008	-4.173	1.273	47.842
NPRL 6441	Good	low	-3.670	4.173	4.543	89.829
NPRL 6442	Good	low	-5.677	-3.912	3.513	110.644
NPRL 6443	Good	good	-3.150	-1.288	2.465	110.966
NPRL 6444	Moderate	low	-5.077	0.797	3.210	123.455
NPRL 6445	Good	low	-5.607	2.364	3.687	102.639
NPRL 6446	Good	low	-5.055	2.684	4.041	93.181
NPRL 6447	Good	low	-6.587	4.526	3.311	87.085
NPRL 6448	Good	low	-4.252	3.109	2.670	70.802
NPRL 6449	Good	low	-4.442	1.864	2.697	46.127
NPRL 6450	Good	low	-8.016	1.430	2.512	64.915

NPRL 6451	Good	low	-4.438	2.096	2.776	46.127
NPRL 6452	Good	low	-7.441	2.562	2.429	64.915
NPRL 6453	Poor	very low, but possible	-4.627	3.262	5.307	125.574
NPRL 6454	Good	low	-6.687	-0.185	3.739	98.759
NPRL 6455	Good	low	-4.773	1.953	4.036	102.639
NPRL 6456	Poor	very low, but possible	-5.808	2.817	5.214	125.488
NPRL 6457	Poor	very low, but possible	-6.008	3.224	5.422	107.689
NPRL 6458	Good	low	-3.052	3.587	3.328	64.915
NPRL 6459	Good	low	-4.976	3.143	2.326	98.541
NPRL 6460	Good	low	-4.886	2.782	2.803	77.725
NPRL 6461	Good	low	-4.521	3.335	3.292	77.725
NPRL 6463	Poor	very low, but possible	-4.952	4.352	6.756	90.132
NPRL 6464	Good	low	-2.340	5.378	4.131	77.725
NPRL 6465	Good	low	-3.556	3.678	3.151	77.725
NPRL 6466	Good	very low, but possible	-4.353	3.535	4.205	77.725
NPRL 6467	Good	low	-3.957	5.381	3.798	73.845
NPRL 6468	Good	low	-3.913	5.519	3.326	73.845
NPRL 6469	Good	low	-0.048	5.426	2.953	46.065
NPRL 6470	Good	low	0.920	5.216	3.654	49.418
NPRL 6471	Good	low	-0.512	5.215	3.649	58.875
NPRL 6472	Good	low	-0.198	7.011	3.475	54.995
NPRL 6473	Good	very low, but possible	4.059	7.450	4.465	27.337
NPRL 6474	Good	low	-6.242	3.126	2.429	64.915
NPRL 6486	Good	very low, but possible	0.662	11.429	5.020	52.664
NPRL 6487	Good	very low, but possible	2.201	3.089	4.946	34.804
NPRL 6488	Good	low	0.002	-1.112	3.504	46.065
NPRL 6489	Good	good	-8.697	-5.277	1.779	76.176
NPRL 6490	Good	low	-3.371	7.011	3.466	68.268
NPRL 6491	Good	low	-5.068	6.058	2.872	77.198
NPRL 6492	Good	low	-6.100	8.293	3.295	82.775
NPRL 6493	Good	low	-4.648	-1.006	3.221	64.915
NPRL 6494	Good	good	-3.964	0.753	2.368	73.210
NPRL 6495	Moderate	very low, but possible	-7.807	3.283	4.423	100.660
NPRL 6496	Good	very low, but possible	-2.712	7.597	3.722	68.268
NPRL 6497	Good	good	-5.814	-0.218	1.031	109.979
NPRL 6498	Good	low	-7.740	3.052	2.136	91.146
NPRL 6499	Good	low	-4.244	3.711	2.649	64.915
NPRL 6500	Good	low	-4.082	3.388	2.855	55.457
NPRL 6501	Good	low	-4.648	4.742	3.204	55.457
NPRL 6502	Moderate	very low, but possible	-7.415	3.779	4.423	100.660
NPRL 6503	Good	low	-2.335	7.799	3.617	68.268
NPRL 6504	Good	opti mal	-6.357	-4.054	-0.143	67.861
NPRL 6511	Good	good	-6.862	2.346	1.757	85.731
NPRL 6512	Good	low	-6.377	4.491	2.790	100.076
NPRL 6513	Good	good	-7.156	1.074	1.434	124.772
NPRL 6514	Good	low	-4.819	1.842	3.235	88.986
NPRL 6515	Good	low	-1.715	-0.571	3.919	80.132
NPRL 6516	Good	very low, but possible	1.590	1.913	4.878	71.202
NPRL 6517	Good	low	-1.474	-1.411	3.815	83.927
NPRL 6518	Good	very low, but possible	1.567	1.838	4.878	71.202
NPRL 6519	Good	low	0.232	2.373	4.141	71.202
NPRL 6520	Good	low	-1.126	-0.054	3.935	71.202
NPRL 6521	Good	very low, but possible	-0.704	0.145	4.552	71.202
NPRL 6522	Good	low	-0.866	0.326	3.919	80.132
NPRL 6523	Good	low	0.311	2.241	4.141	71.202
NPRL 6524	Moderate	Extremely low	0.525	0.575	5.820	71.202
NPRL 6525	Good	low	-2.547	-1.111	3.814	94.137
NPRL 6526	Good	low	-0.331	0.427	4.346	71.202
NPRL 6527	Good	low	0.859	2.712	4.600	71.202
NPRL 6528	Poor	very low, but possible	-1.800	1.904	6.055	80.132
NPRL 6529	Good	low	-2.067	-1.695	3.307	91.393
NPRL 6530	Good	low	-1.026	-1.773	3.371	82.463
NPRL 6531	Good	low	-9.246	-2.286	3.461	105.804
NPRL 6532	Moderate	low	-5.419	0.422	3.830	114.025
NPRL 6533	Good	low	-0.498	-1.050	3.331	92.018
NPRL 6534	Good	low	-0.670	-1.177	3.331	92.018
NPRL 6535	Good	low	-3.150	-1.959	3.189	97.742
NPRL 6536	Moderate	low	-1.220	4.648	4.709	98.541
NPRL 6537	Moderate	low	-1.220	4.584	5.165	98.541
NPRL 6538	Poor	low	-1.488	4.721	5.621	98.541
NPRL 6539	Good	low	-5.088	1.470	1.962	52.105
NPRL 6540	Good	low	-4.596	2.690	2.418	52.105
NPRL 6541	Good	low	-5.625	2.269	3.234	74.373
NPRL 6542	Good	low	-6.896	8.331	2.945	91.705
NPRL 6543	Good	low	-7.728	7.906	2.770	95.585
NPRL 6544	Good	low	-7.278	7.117	2.293	116.401

NPRL 6545	Good	low	-6.154	2.703	2.677	64.915
NPRL 6546	Good	very low, but possible	1.681	6.617	5.138	38.785
NPRL 6547	Good	very low, but possible	-0.313	6.292	5.059	65.016
NPRL 6548	Good	low	-8.165	1.773	2.606	76.176
NPRL 6549	Good	good	-8.693	1.588	1.228	76.176
NPRL 6553	Very poor	very low, but possible	-4.145	1.613	7.385	112.318
NPRL 6554	Very poor	very low, but possible	-5.115	2.043	7.051	102.860
NPRL 6555	Good	low	1.173	4.617	3.505	55.976
NPRL 6556	Good	very low, but possible	0.186	5.199	4.796	38.785
NPRL 6557	Good	very low, but possible	0.244	3.242	5.047	45.490
NPRL 6558	Good	low	-1.308	4.637	3.970	65.016
NPRL 6559	Good	low	0.355	5.577	4.000	47.715
NPRL 6560	Good	very low, but possible	-0.573	5.166	5.472	38.785
NPRL 6561	Good	low	-0.232	4.250	3.591	59.600
NPRL 6562	Good	very low, but possible	-1.127	2.271	4.369	73.196
NPRL 6563	Good	low	-6.351	-3.586	2.531	50.046
NPRL 6567	Good	good	-6.411	-5.255	0.845	54.792
NPRL 6568	Good	low	-1.367	-0.432	4.250	37.491
NPRL 6569	Good	good	-3.472	-0.738	2.172	43.531
NPRL 6571	Good	low	-1.004	1.165	3.842	38.785
NPRL 6572	Good	low	-3.641	-3.261	3.120	50.046
NPRL 6573	Good	low	-2.398	-2.777	3.494	37.491
NPRL 6574	Good	low	-2.374	-1.910	3.728	37.491
NPRL 6575	Good	low	-0.111	1.756	4.450	26.230
NPRL 6585	Very poor	good	-2.046	-7.427	1.237	151.123
NPRL 6586	Good	low	0.107	4.694	4.637	43.531
NPRL 6587	Very poor	good	-8.752	3.385	4.436	220.197
NPRL 6588	Very poor	good	-7.676	10.204	4.346	229.127
NPRL 6589	Good	low	-2.935	7.538	3.714	70.881
NPRL6590	Very poor	good	-7.200	-15.023	0.177	153.024
NPRL6591	Very poor	good	-5.759	-17.027	-0.065	173.840
NPRL6592	Very poor	good	-6.452	-17.102	-0.065	173.840
NPRL6593	Very poor	good	-7.774	-14.165	0.161	161.954
NPRL6594	Very poor	good	-5.694	-21.186	-0.210	177.354
NPRL6597	Very poor	opti mal	-3.797	-22.085	-1.450	191.140
NPRL6599	Very poor	good	-7.084	-10.665	1.196	186.284
NPRL6600	Very poor	too soluble	-1.627	-8.688	-2.620	66.855
NPRL6601	Very poor	low	-6.758	-9.028	1.811	250.632
NPRL6602	Very poor	good	-14.341	-3.978	1.288	205.486
NPRL6603	Very poor	too soluble	-3.692	-8.779	-2.684	34.601
NPRL6605	Very poor	too soluble	-0.216	-7.639	-2.638	66.855
NPRL6606	Good	opti mal	-4.938	-3.693	-0.778	70.071
NPRL6607	Moderate	opti mal	-5.348	-2.697	0.931	3.352
NPRL6608	Poor	too soluble	-1.746	-6.614	-1.914	66.855
NPRL6609	Poor	too soluble	-3.938	-8.549	-2.095	83.663
NPRL6610	Very poor	too soluble	-2.606	-9.332	-3.883	47.411
NPRL6611	Good	opti mal	-4.887	-2.207	0.866	64.184
NPRL6613	Good	opti mal	-6.551	-3.776	0.085	77.424
NPRL6614	Good	opti mal	-4.887	-2.207	0.866	64.184
NPRL6615	Good	low	-3.902	9.718	3.183	50.764
NPRL6616	Good	low	-4.644	6.625	3.183	50.764
NPRL6617	Good	good	0.716	1.809	1.587	43.531
NPRL6618	Good	opti mal	-4.731	-3.270	-0.778	70.071
NPRL6620	Good	opti mal	-5.873	-2.323	-0.097	61.141
NPRL6622	Good	opti mal	-3.814	-19.009	0.241	65.671
NPRL6623	Moderate	too soluble	-3.819	-16.509	-1.674	88.880
NPRL6624	Good	good	-6.307	-7.217	2.061	103.226
NPRL6625	Very poor	too soluble	-4.718	-11.540	-2.723	83.018
NPRL6626	Good	opti mal	-4.160	-21.511	-0.659	74.601
NPRL6628	Very poor	good	-5.648	-13.157	0.867	165.672
NPRL6629	Good	good	-4.480	-4.389	0.873	73.642
NPRL6630	Good	low	-0.105	2.155	3.485	50.364
NPRL6632	Good	low	-1.860	-0.781	2.821	50.364
NPRL6633	Good	opti mal	-5.247	-5.605	0.219	50.114
NPRL6635	Good	opti mal	-4.029	-1.666	0.977	47.605
NPRL6637	Good	opti mal	-9.304	-1.728	0.570	49.656
NPRL6638	Good	opti mal	-5.092	-1.596	0.318	43.531
NPRL6639	Good	opti mal	-2.249	-2.198	-0.034	34.601
NPRL6640	Good	good	-3.730	0.737	1.466	43.531
NPRL6641	Good	opti mal	-4.397	-4.946	-0.739	73.951
NPRL6644	Good	opti mal	-5.910	-4.785	-0.012	76.232
NPRL6645	Moderate	opti mal	-6.891	-6.543	-0.060	24.167
NPRL6646	Moderate	good	-4.352	24.422	0.636	143.599
NPRL6647	Good	low	-3.470	6.975	2.494	88.182
NPRL6648	Good	good	-1.739	13.496	1.619	107.097
NPRL6649	Good	good	-1.087	29.026	2.163	100.626

NPRL6650	Very poor	very low, but possible	0.145	9.611	7.341	95.211
NPRL6651	Good	low	-2.515	34.872	3.021	100.626
NPRL6652	Good	very low, but possible	-0.224	25.937	5.174	79.811
NPRL6653	Good	low	-1.723	25.191	2.932	108.997
NPRL6654	Good	low	-1.752	14.030	2.966	91.696
NPRL6655	Good	low	-1.793	7.643	2.958	114.413
NPRL6656	Good	low	-2.111	15.136	3.388	82.766
NPRL6657	Poor	very low, but possible	0.112	16.815	4.976	117.927
NPRL6658	Moderate	very low, but possible	-2.659	7.515	5.485	74.396
NPRL6659	Good	good	-1.943	9.248	2.572	104.141
NPRL6660	Good	low	-2.278	17.590	2.490	97.112
NPRL6661	Very poor	low	-5.410	17.889	3.943	182.758
NPRL6662	Good	low	-1.046	18.972	3.613	97.112
NPRL6663	Good	low	-0.323	18.527	3.188	97.112
NPRL6664	Moderate	low	-1.679	18.907	3.990	106.042
NPRL6665	Good	low	-0.484	21.230	4.283	79.811
NPRL6666	Moderate	good	-3.456	23.540	2.684	135.228
NPRL6667	Poor	low	-9.403	13.908	6.664	149.574
NPRL6668	Very poor	opti mal	-3.699	18.078	5.031	194.224
NPRL6669	Good	low	1.111	19.246	4.189	97.112
NPRL6670	Good	good	-5.106	-0.204	1.785	53.021
NPRL6671	Good	good	-4.011	0.340	2.364	53.021
NPRL6672	Good	good	-4.670	1.068	2.364	53.021
NPRL6673	Good	low	-3.323	1.460	3.955	61.391
NPRL6674	Good	good	-3.871	-3.313	1.846	60.832
NPRL6675	Good	very low, but possible	0.056	-0.550	4.801	56.901
NPRL6676	Good	good	-4.715	2.474	1.328	73.277
NPRL6677	Good	very low, but possible	-1.046	7.809	4.859	54.310
NPRL6678	Good	low	2.885	7.035	3.495	60.416
NPRL6679	Good	low	0.695	6.541	3.668	65.831
NPRL6680	Good	low	2.069	8.259	3.871	50.958
NPRL6681	Good	very low, but possible	5.848	4.947	5.462	50.958
NPRL6682	Good	good	-4.432	-2.124	1.499	64.906
NPRL6683	Good	good	-7.122	-3.756	1.190	93.930
NPRL6684	Good	very low, but possible	4.444	7.528	5.535	60.416
NPRL6686	Good	low	0.371	6.873	2.532	64.744
NPRL6688	Good	low	3.919	7.915	3.951	60.416
NPRL6689	Moderate	very low, but possible	2.590	8.164	5.776	60.416
NPRL6692	Good	good	-7.263	2.219	1.777	104.214
NPRL6694	Good	low	1.782	7.919	2.425	74.202
NPRL6699	Good	low	1.620	8.766	4.706	74.202
NPRL6701	Good	very low, but possible	5.012	4.176	5.005	50.958
NPRL6703	Good	good	-6.412	3.858	2.299	107.567
NPRL6704	Moderate	very low, but possible	2.743	5.797	5.708	65.831
NPRL6709	Good	good	-7.322	4.555	1.683	61.391
NPRL6710	Good	low	1.916	6.547	4.125	65.831
NPRL6711	Good	low	-0.054	7.078	2.075	64.744
NPRL6712	Good	good	-2.308	3.770	1.753	61.391
NPRL6713	Moderate	low	-10.497	-3.129	3.339	116.497
NPRL6714	Good	low	3.438	6.864	3.552	74.202
NPRL6715	Good	very low, but possible	1.498	5.776	4.231	56.373
NPRL6719	Good	low	-5.328	5.750	3.161	104.214
NPRL6720	Good	low	2.008	6.034	3.659	64.744
NPRL6724	Good	low	2.780	6.821	3.482	50.958
NPRL6728	Good	very low, but possible	3.347	12.416	5.765	33.495
NPRL6732	Good	good	-8.589	0.563	2.985	111.082
NPRL6733	Good	very low, but possible	3.576	5.877	4.115	64.744
NPRL6734	Good	low	-0.212	8.204	2.100	64.744
NPRL6737	Poor	too soluble	-6.134	-5.166	-1.561	51.210
NPRL6738	Moderate	too soluble	-1.451	-8.294	-1.707	66.855
NPRL6739	Good	good	-8.209	-0.499	1.416	82.045
NPRL6741	Good	low	2.549	6.286	4.905	74.202
NPRL6742	Good	low	1.126	4.159	2.731	64.744
NPRL6743	Good	very low, but possible	3.882	6.280	5.066	50.958
NPRL6744	Good	low	1.945	7.325	3.595	47.443
NPRL6745	Moderate	too soluble	-1.558	-8.288	-1.251	66.855
NPRL6748	Good	very low, but possible	1.387	13.676	5.765	33.495
NPRL6749	Good	very low, but possible	2.771	7.259	5.383	56.373
NPRL6750	Good	very low, but possible	-4.736	-1.670	4.699	73.674
NPRL6751	Good	low	1.547	5.749	2.624	74.202
NPRL6752	Good	low	0.527	5.317	2.299	64.744
NPRL6753	Good	good	-11.965	-1.149	1.033	82.045
NPRL6759	Good	low	-3.972	-0.199	2.999	82.045
NPRL6760	Good	too soluble	-4.947	-8.392	-0.464	58.484
NPRL6761	Poor	too soluble	-2.589	-7.654	-1.119	26.230
NPRL6763	Good	good	-7.204	-4.557	1.479	50.809

NPRL6769	Good	opti mal	-2.829	-6.750	1.817	58.484
NPRL6770	Poor	too soluble	-4.710	-8.851	-0.687	26.230
NPRL6771	Good	good	-11.856	1.334	1.490	82.045
NPRL6774	Good	low	-3.717	-9.332	3.916	88.766
NPRL6775	Good	low	-2.383	-4.562	4.563	74.761
NPRL6776	Good	low	-3.937	-9.316	3.916	88.766
NPRL6777	Moderate	low	-5.198	-0.686	4.490	99.724
NPRL6778	Good	low	-4.863	-21.561	3.132	83.132
NPRL6779	Good	low	-1.103	-5.625	4.546	83.691
NPRL6780	Moderate	very low, but possible	-3.877	-1.576	4.490	99.724
NPRL6782	Moderate	low	-6.358	-7.350	3.573	118.788
