# Supplementary material for: Artificial intelligence-driven prediction and validation of blood–brain barrier permeability and absorption, distribution, metabolism, excretion profiles in natural product research laboratory compounds
Source: Biomedicine (Taipei). 2024 Dec 1;14(4):82–91. doi: 10.37796/2211-8039.1474 (PMC11703399; doi:10.37796/2211-8039.1474)
Supplement: Supplementary file 3 [file bmed-14-04-082-s003.pdf]

ORIGINAL ARTICLE

Artificial intelligence-driven prediction and validation of blood−brain barrier permeability and absorption, distribution, metabolism, excretion profiles in natural product research laboratory compounds

Q6 Jai-Sing Yang [a,1], Eddie TC. Huang [b,1], Ken YK. Liao [b], Da-Tian Bau [a,c,d], Shih-Chang Tsai [e], Chao-Jung Chen [f,g], Kuan-Wen Chen [h], Ting-Yuan Liu [i], Yu-Jen Chiu [j,k], Fuu-Jen Tsai [l,m,n,*]

Genetics Generation Advancement Corporation, Molecular science and digital innovation center, Taipei, Taiwan

[a] Department of Medical Research, China Medical Univ[illegible]
[b] NVIDIA AI Technology Center, NVIDIA Corporation, USA
[c] Graduate Institute of Biomedical Sciences, China Medical University, Taichung, Taiwan
[d] Terry Fox Cancer Research Laboratory, Department of Medical Research, China Medical University Hospital, Taichung, Taiwan
[e] Department of Biological Science and Technology, China Medical University, Taichung, Taiwan
[f] Proteomics Core Laboratory, Department of Medical Research, China Medical University Hospital, Taichung, Taiwan
[g] Graduate Institute of Integrated Medicine, College of Chinese Medicine, Medical University, Taichung, Taiwan
[h] ~~GGA, Corp. Molecular Science and Digital Innovation Center, Taipei, Taiwan~~
[i] Million-Person Precision Medicine Initiative, Department of Medical Research, China Medical University Hospital, Taichung, Taiwan
[j] Division of Plastic and Reconstructive Surgery, Department of Surgery, Taipei Veterans General Hospital, Taipei, Taiwan
[k] Department of Surgery, School of Medicine, National Yang Ming Chiao Tung University, Taipei, Taiwan
[l] School of Chinese Medicine, College of Chinese Medicine, China Medical University, Taichung, Taiwan
[m] China Medical University Children's Hospital, Taichung, Taiwan
[n] Department of Medical Genetics, China Medical University Hospital, Taichung, Taiwan

Abstract

Introduction: **Our previous research demonstrated that a large language model (LLM) based on the transformer architecture, specifically the MegaMolBART encoder with an XGBoost classifier, effectively predicts the blood−brain barrier (BBB) permeability of compounds. However, the permeability coefficients of compounds that can traverse this barrier remain unclear. Additionally, the absorption, distribution, metabolism, and excretion (ADME) characteristics of substances obtained from the Natural Product Research Laboratory (NPRL) at China Medical University Hospital (CMUH) have not yet been determined.**

Objectives: **The study aims to investigate the pharmacokinetic ADME properties and BBB permeability coefficients of NPRL compounds.**

Materials and methods: **A combined model using a transformer-based MegaMolBART encoder and XGBoost classifier was employed to predict BBB permeability. Machine learning (ML) tools from Discovery Studio were used to assess the ADME characteristics of the NPRL compounds. The CCK-8 assay was conducted to evaluate the cytotoxic effects of NPRL compounds on bEnd.3 brain endothelial cells after exposure to 10 μg/mL of the compounds. We assessed the permeability coefficient by subjecting bEnd.3 cell monolayers to the test compounds and measuring the permeability of FITC-dextran.**

Results: **There were 4956 compounds that could cross the blood−brain barrier (BBB+) and 2851 that could not (BBB−) in the B3DB dataset that was utilized for training. A total of 2461 BBB+ and 2184 BBB− compounds were used in the NPRL-CMUH dataset for testing. The permeability coefficient of temozolomide (TMZ) and 21 other BBB + compounds**

Received 14 October 2024; accepted 29 October 2024.
Q1 Available online ■ ■ ■

November 11

* Corresponding author at: School of Chinese Medicine, College of Chinese Medicine, China Medical University, Taichung, Taiwan.
E-mail address: 000704@tool.caaumed.org.tw (F.-J. Tsai).
[1] Contributed equally.

https://doi.org/10.37796/2211-8039.1474
2211-8039/Published by China Medical University 2024. © the Author(s). This is an open access article under the CC BY license (http://creativecommons.org/licenses/by/4.0/).

exceeded 10 × 10^{-7} cm/s. Computational analysis revealed that NPRL compounds exhibited a variety of ADME characteristics.

Conclusion: **Computer-based predictions for the NPRL of CMUH compounds regarding their capacity to traverse the BBB are verified by the findings. Artificial intelligence (AI) prediction models have effectively identified the potential ADME characteristics of various compounds.**

Keywords: Large language model (LLM), Machine learning (ML), Blood–brain barrier (BBB), Absorption, distribution, metabolism, and excretion (ADME), Natural products research laboratories (NPRL)

ORIGINAL ARTICLE

1. Introduction

Human health and well-being face substantial challenges due to neurological conditions, including Alzheimer's disease (AD), Parkinson's disease (PD), stroke (both ischemic and hemorrhagic), epilepsy, headaches, and brain tumors [1,2]. Identifying compounds capable of crossing the blood–brain barrier (BBB) and effectively targeting the central nervous system (CNS) remains a significant challenge in developing treatments for neurological disorders. This highly selective boundary restricts the passage of most substances into the brain [3,4]. To be considered viable therapeutic agent candidates for CNS diseases, compounds must demonstrate therapeutic efficacy and possess the ability to cross the BBB to reach their intended targets [4,5]. Candidates that can cross the BBB usually have certain traits: they are very attracted to fats, have a low molecular mass (usually less than 400–600 Da), do not bind well to plasma proteins very well, and do not break apart easily at physiological pH condition [6,7]. These strict requirements for BBB permeability make it challenging for larger molecules such as peptides, recombinant proteins, monoclonal antibodies, and gene therapies to traverse the barrier independently [8,9]. Furthermore, even when compounds capable of crossing the BBB are identified, their progression is often hindered by their suboptimal pharmacokinetic (PK) properties. These PK properties, which include absorption, distribution, metabolism, excretion, and toxicity (ADMET) profiles, contribute to high failure rates during new drug development [10,11]. In recent years, advancements in computational prediction models and artificial intelligence (AI) have significantly improved the efficiency of identifying compounds with desirable BBB permeability and ADMET characteristics [12–15].

Various AI research groups have developed predictive models for BBB penetration utilizing these tools to expedite CNS drug discovery [1,16–20]. For instance, Zhang *et al.* employed MolconnZ, MOE, and Dragon models to develop a quantitative structure–activity relationship (QSAR) model for BBB permeability, utilizing data from 159 compounds capable of passing through the BBB [21]. Similarly, Wang *et al.* applied six machine learning (ML) algorithms to categorize 2358 compounds using the scikit-learn framework (version 0.19.1) [22]. Miao *et al.* implemented POLY-Support Vector Machine (POLY-SVM), Sigmoid-Support Vector Machine (Sigmoid-SVM), K-Nearest Neighbor (KNN), Radial Basis Function-Support Vector Machine (RBF-SVM), Decision Tree (DT), and other deep learning techniques to predict BBB permeability [23]. Liang *et al.* investigated classification models to elucidate the structural features and transformation rules associated with compounds

List of abbreviations

AI	Artificial intelligence
AD	Alzheimer's disease
ADME	Absorption, distribution, metabolism, excretion
ADMET	Absorption, distribution, metabolism, excretion, and toxicity
BBB	Blood–brain barrier
bEnd.3	Brain endothelial cells bEnd.3
CCK-8	Cell counting kit-8
CMUH	China Medical University Hospital
CNS	Central nervous system
CYP2D6	Cytochrome P450_2D6 enzyme
DMEM	Dulbecco's Modified Eagle medium
DMSO	Dimethyl sulfoxide
FBS	Fetal bovine serum
FITC-dextran	Fluorescein isothiocyanate-dextran
HIA	Human intestinal absorption
LC-MS/MS	liquid chromatography-tandem mass spectrometry
LLM	Large Language Model
ML	Machine learning
MOA	Mechanism of action
NPRL	Natural Product Research Laboratory
~~PD~~	~~Parkinson's disease~~
PD	Pharmacodynamics
PK	Pharmacokinetics
PPB	Plasma protein binding
PSA_2D	2D polar surface area
QSAR	Quantitative structure–activity relationship
SMILES	Simplified molecular input line entry system

(Table 1)

that can traverse the BBB [24]. Kumar *et al.* introduced "DeePred-BBB," a deep learning-based model capable of predicting BBB permeability from simplified molecular input line entry system (SMILES) representations [25].

Our previous research demonstrated the ability of the large language model (LLM) in the transformer-based MegaMolBART encoder with XGBoost classifier to predict BBB permeability across an extensive compound library, although permeability coefficients remain undetermined. Furthermore, the pharmacokinetic ADME characteristics of compounds from the Natural Product Research Laboratory (NPRL) at China Medical University Hospital (CMUH) have not been fully explored. This study's objective was to use the FITC-dextran extravasation model to develop an *in vitro* model to assess BBB permeability and predict the pharmacokinetic (PK) behavior of NPRL-CMUH compounds. Additionally, this study integrates machine learning (ML) techniques (Fig. 1).

2. Methods

2.1. Cell culture

The bEnd.3 cells (immortalized mouse brain endothelial cells) from ATCC (Catalog no. CRL-2299; Manassas, VA, USA) were cultured in Dulbecco's Modified Eagle's Medium (DMEM) (American Type Culture Collection, Manassas, VA, USA) supplemented with 10% fetal bovine serum (R&D Systems, Minneapolis, MN, USA) and 100 μg/mL penicillin/streptomycin (Sigma–Aldrich, St. Louis, MO, USA) at 5% CO_2 and 95% air [26,27].

2.2. In vitro *permeability assay*

The bEnd.3 cells (5.0 × 10^4 cells/cm^2) were cultured onto 24-well transwell inserts (pore size 0.4 μm; Corning Inc., Corning, NY, USA). After 72 h, the cells formed a confluent monolayer and were then treated with NPRL compounds (10 μg/mL) or dimethyl sulfoxide (DMSO) (1 μL) as control for 12 h. Following the treatment, the inserts and chambers were PBS washed, and the medium was replaced with phenol-red free medium. Subsequently, 10 kDa FITC-dextran (10 mg/mL; 10 μL; Sigma–Aldrich) was added to the upper inserts and incubated at 37 °C for 2 h. Fluorescence was measured in the upper and lower chambers using a fluorescence plate reader (SpectraMAX M3; Molecular Devices, Sunnyvale, CA, USA) at an excitation/emission wavelength of 490 nm/520 nm [27]. The permeability coefficient was calculated using the following formula:

Permeability coefficient (cm/s): $P_{dextran} = (RFU_{lower}/RFU_{upper})\ (V)\ (1/t)\ (1/A)$

RFU_{lower}: relative fluorescent units in the lower well.

RFU_{upper}: relative fluorescent units in the upper well.

V: volume of the bottom well.

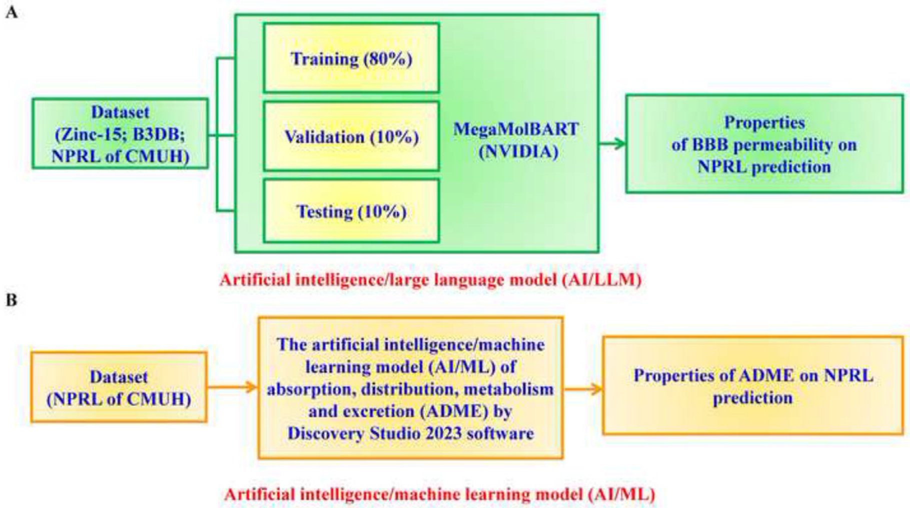

Fig. 1. AI models for predicting BBB permeability and ADME properties. (A) A system that employs AI/LLM to predict how compounds cross the BBB. This system analyzes molecular properties using NVIDIA's MegaMolBART decoder and a SMILES encoder that works with the B3DB, Zinc-15 and NPRL of CMUH datasets. (B) The ML model, implemented using the AI/ML model by Discovery Studio 2023 software, generates predictions of ADME properties.

T: time that the FITC-dextran was allowed to diffuse.

A: total surface area of the monolayer (cm^2).

2.3. *Cytotoxicity assay*

The bEnd 3 cells (5 × 10^3 cells/well) were seeded in 96-well plates, and then incubated for 24 h in a humidified incubator at 37 °C with 5% CO_2. NPRL compounds (10 μg/mL) were then added to the culture media in the plates. The plates were incubated for an additional 20 h. Following this, 10 μL of CCK-8 solution was added to each well. The plates were incubated for 1–4 h, after which the absorbance was measured at 450 nm using an ELISA reader (detection: 450 nm, reference: 650 nm) [28].

2.4. In silico *BBB permeability prediction*

We employed the transformer-based MegaMolBART encoder (NVIDIA, Version 1.0; URL: https://catalog.ngc.nvidia.com/orgs/nvidia/teams/clara/models/megamolbart) with an XGBoost classifier (URL: github.com/dmlc/xgboost) to predict BBB permeability [1]. This model was trained on the B3DB dataset and tested on the NPRL compound library. The MegaMolBART encoder processes the molecular structure information, while the XGBoost classifier uses this encoded information to make BBB permeability predictions. This approach allows efficient and accurate assessment of a molecule's ability to cross the BBB based on its structural features.

2.5. In silico *pharmacokinetic ADME properties prediction*

In silico pharmacokinetic properties prediction studies utilized the ADME algorithm of the Discovery Studio 2023 machine learning model (ML) software (BIOVIA), accessible at (URL: https://www.3ds.com/products/biovia/discovery-studio/qsar-admet-predictive-toxicology) (Fig. 1). This software calculates various ADME properties, including aqueous solubility (predicting the solubility of each compound in water at 25 °C), BBB penetration, CYP2D6 binding (predicting cytochrome P450 2D6 enzyme inhibition), human intestinal absorption (HIA) after oral administration, plasma protein binding (PPB) (predicting the likelihood that a compound will be highly bound to carrier proteins in the blood), as well as AlogP98 and PSA_2D [29,30].

The training set for the aqueous solubility prediction model comprised 775 compounds with molecular weights ranging from 50 to 800. These compounds included alkanes, alkenes, alkynes, halogens, amines, alcohols, nitrogen-containing compounds, ketones, aldehydes, and organic acids. The plot of predicted versus experimental data yielded a linear regression of LogSw (25 °C, pH = 7.0) with R^2 = 0.84 and a standard deviation (SD) of 0.87. The pre-test dataset included 34 compounds, resulting in regression statistics of R^2 = 0.88 and SD = 0.79. Additionally, a validation test dataset of 1615 compounds from the PDR and Comprehensive Medicinal Chemistry database (CMC) produced an overall RMSE (SD) of 1.0 [31].

The BBB penetration model makes educated guesses about how much of a drug will enter the brain after oral administration by using data from over 800 compounds known to penetrate the BBB. This model employs a quantitative linear regression for predicting blood–brain penetration, accompanied by 95% and 99% confidence ellipses in the ADMET_PSA_2D and ADMET_AlogP98 planes [32].

The cytochrome P450_2D6 (CYP2D6) model predicts CYP2D6 enzyme inhibition based on 2D chemical structures. Using modified Bayesian learning [33], training set of 151 structurally different compounds with known CYP2D6 inhibition constants were used to make it. The AI/ML model was created using 182 compounds in the training dataset for human intestinal absorption. This model incorporates AlogP98 and 2D polar surface area (PSA_2D). Compounds with well-documented human intestinal absorption exhibit at least 90% absorption into the human bloodstream and typically fall within the 95% and 99% confidence ellipse regions [34].

The plasma protein binding model predicts whether a compound is likely to be highly bound (≥90% bound) to carrier proteins in the blood. The training dataset comprised 854 compounds divided into 329 binders and 525 non-binders. A modified Bayesian learning approach created a binary classification model [35,36].

2.6. *Statistical analysis*

The results are shown as the mean ± standard deviation (SD). Statistical analysis is performed using a one-way analysis of variance or Tukey's test. Levels of significance are represented as ***$P < 0.001$, indicating statistical discrepancies between the control group and the NPRL-treated groups [37–39].

3. Results

3.1. In silico *BBB permeability prediction*

In our prior research, we utilized a LLM-based on the transformer-based MegaMolBART encoder

with XGBoost classifier architecture to forecast the ~~blood–brain barrier (BBB)~~ BBB permeability of compounds sourced from the NPRL. We validated these predictions by conducting *in vitro* experiments with human BBB spheroid cells and evaluating BBB integrity using liquid chromatography-tandem mass spectrometry (LC-MS/MS). Our findings highlighted the significant potential of this approach for advancing BBB permeability research in new drug discovery. The LLM of the transformer-based MegaMolBART encoder with XGBoost classifier exhibited strong predictive performance, as demonstrated by the composition of the training and testing datasets. The training dataset sourced from the B3DB database, comprised 4956 BBB-permeable (BBB+) and 2851 BBB-impermeable (BBB−) compounds (Fig. 2A). The NPRL testing dataset included 2461 BBB+ and 2184 BBB− compounds (Fig. 2B).

3.2. In vitro *BBB permeability and cytotoxicity analysis*

The effect of NPRL compounds on endothelial cell permeability was evaluated using an *in vitro* blood–brain barrier (BBB) model consisting of confluent bEnd.3 monolayers grown in transwell chambers. As shown in Fig. 3, exposure to 10 μg/mL of various NPRL substances (NPRL309, NPRL358, NPRL588, NPRL818, NPRL833, NPRL835, NPRL836, NPRL842, NPRL1089, NPRL1185, NPRL1188, NPRL1192, NPRL1195, NPRL1241, NPRL1958, NPRL2026, NPRL2029, NPRL2051, NPRL2059, NPRL2148, and NPRL3767) led to a notable increase in monolayer permeability, as evidenced by the greater diffusion of 10 kDa FITC-dextran across the transwell membrane. Conversely, five BBB-impermeable compounds (NPRL2359, NPRL2576, NPRL2646, NPRL3098, and NPRL3183) reduced the monolayer permeability (Fig. 3).

To verify that the enhanced BBB permeability was not caused by the toxic effects on bEnd.3 cells, we assessed cell viability using CCK-8 assays. The results, depicted in Fig. 4, demonstrated that exposing bEnd.3 cells (10 μg/mL) of NPRL compounds had a negligible cytotoxic impact. This indicated that the observed permeability changes were not a result of compound-induced cell damage. These results corroborate our *in silico* predictions of the BBB permeability characteristics of the NPRL compound library.

3.3. In silico *prediction of pharmacokinetic properties (ADME)*

To estimate the drug-like potential of the NPRL compounds, an *in silico* ADME study was conducted using Discovery Studio 2023 software within a ML model. This study predicted various pharmacokinetic properties, including intestinal absorption, compound solubility, metabolism by CYP2D6, plasma protein binding (PPB) capability, and blood–brain barrier (BBB) permeability, alongside the compound's AlogP98a and PSA properties. Computer analysis of the ADME parameters showed that the NPRL compounds had different pharmacokinetic profiles, as shown in Fig. 5 and ~~Table 1~~ Table 2. Additional raw data are presented in Supplementary

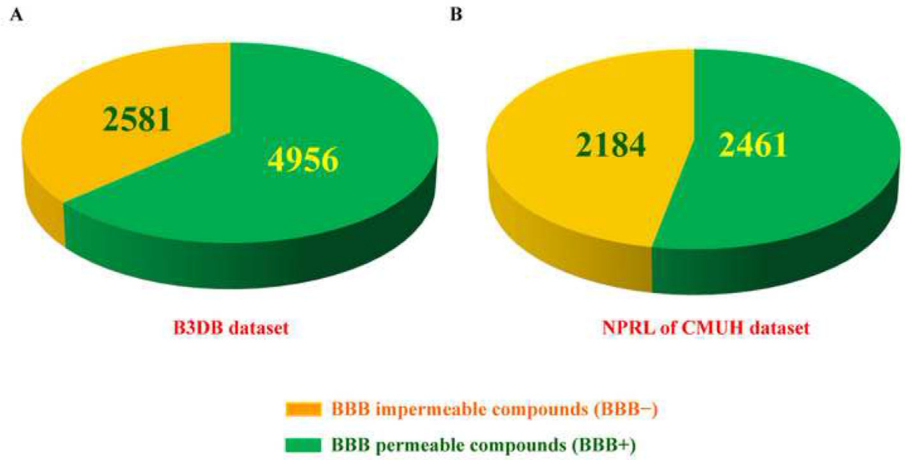

Fig. 2. BBB permeability distribution of compounds in the training and testing datasets. This figure displays compounds that can (BBB+) and cannot (BBB−) penetrate the blood–brain barrier from two databases: the B3DB training dataset (A) and the NPRL-CMUH testing dataset (B). (A) In the B3DB dataset, the total number of BBB-impermeable (BBB−) compounds is 2581, while the number of BBB-permeable (BBB+) compounds is 4956. (B) In the NPRL of CMUH dataset, the total number of BBB-impermeable compounds is 2184, and the number of BBB-permeable compounds is 2461.

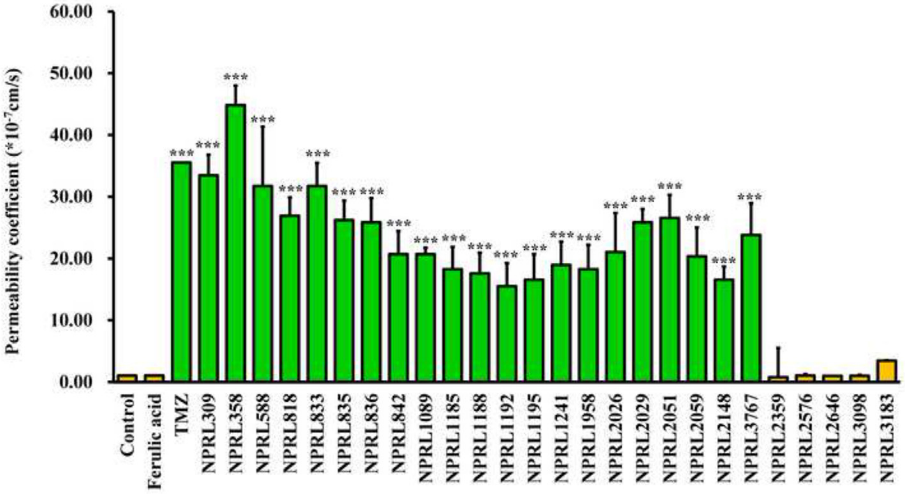

Fig. 3. Permeability coefficients of NPRL compounds on bEnd.3 cells- FITC-dextran extravasation model. The chart illustrates the permeability coefficients (10^{-7} cm/s) for various NPRL compounds, including control substances such as ferulic acid and TMZ. Each column in the graph represents a specific NPRL compound, highlighting notable differences in permeability. Asterisks are used to denote statistical significance, indicating which compounds exhibit significant permeability compared to the control group. NPRL compounds show a range of permeability effects, emphasizing their varying capacities to traverse the BBB. (***$P < 0.001$ relative to the respective vehicle-treated control groups).*

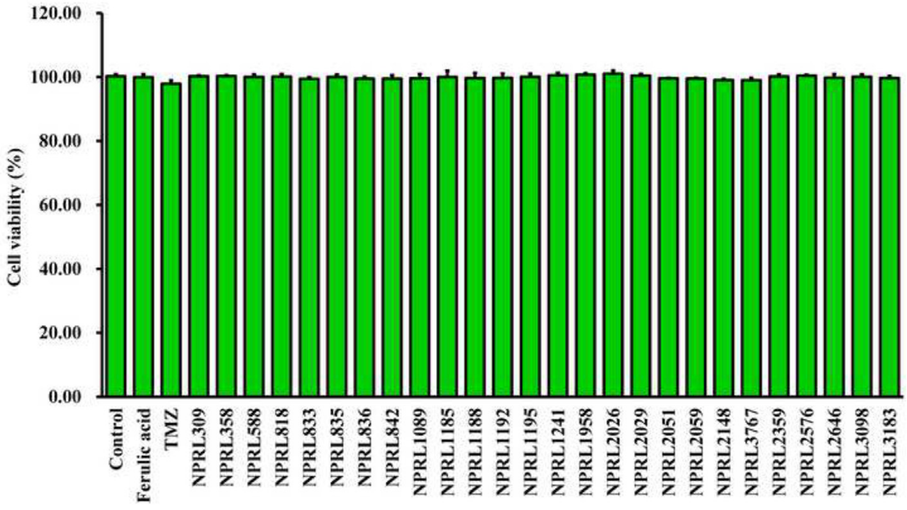

Fig. 4. Cytotoxicity effects of NPRL compounds on bEnd.3 cells. bEnd.3 cells (5×10^3 cells/mL/well) were seeded in 96-well plates and exposed to NPRL compounds (10 μg/mL) for 24 h. Cytotoxicity was assessed using the CCK-8 assay ($n = 3$).

Table 1 (https://www.biomedicinej.com/cgi/editor.cgi?article=1474&window=additional_files&context=biomedicine). These results highlight the differences in ADME properties among the compounds, providing essential information for the future Q3 refinement of new drug development processes (see Table 2).

4. Discussion

In vitro and *in vivo* experiments and clinical trials typically yield the most accurate BBB permeability data. However, obtaining experimental BBB permeability data for numerous compounds, particularly across extensive libraries, is both time consuming and expensive [20]. To address these challenges, AI-based computational methods have been developed to assess BBB permeability, providing a crucial and strategic alternative [1,40]. These AI approaches offer significant benefits, including improved efficiency, reduced costs, and the ability to quickly process large volumes of data [41,42]. In contemporary research, most scientists have utilized the chemical characteristics of substances, such as molecular fingerprints and two-dimensional molecular descriptors, to develop

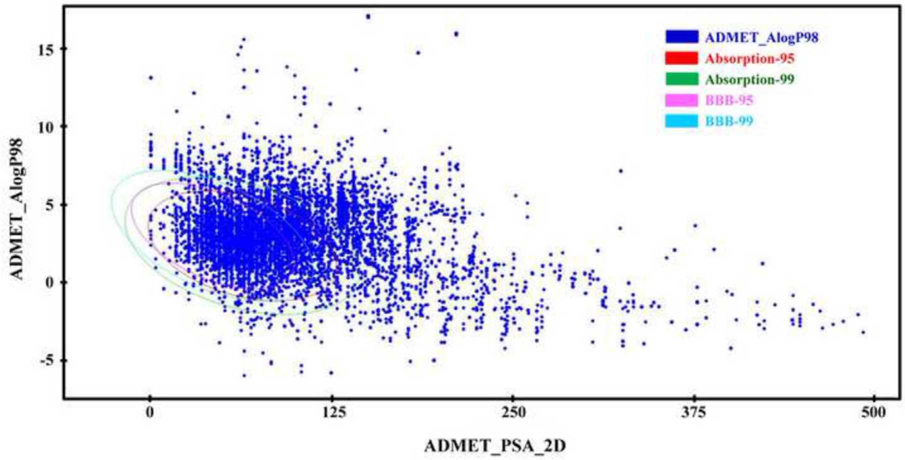

Fig. 5. ADME plot for various NPRL compounds. Each compound's 2D polar surface area (PSA_2D) is plotted against the calculated atom-type partition coefficient (ALogP98). The ellipse's perimeter predicts good absorption without violating ADME properties.

Table 1. The ratio of BBB permeability in different compound libraries.

Dataset	BBB permeable compounds (BBB+)	BBB impermeable compounds (BBB−)	URL	Ref
LightBBB	5453	1709	https://bio.tools/lightbbb	[62]
DeePred-BBB	2607	998	https://github.com/12rajnish/DeePred-BBB	[25]
B3DB	4956	2851	https://github.com/theochem/B3DB	[4]
NPRL of CMUH	2461	2184	Non	[1]

predictive models. Various datasets and algorithms assess the likelihood of compounds penetrating the BBB [43–48]. This method speeds up the process of finding possible drugs for disorders of the CNS by allowing many compounds to be screened simultaneously and reducing the need for extensive experimental testing [43–48].

We used a LLM built on the transformer-based MegaMolBART encoder with XGBoost classifier architecture to try to guess how compounds from the NPRL would pass through the BBB (Fig. 1 up, and Fig. 2). We also performed *in vitro* experiments (Fig. 1 down and Fig. 3) to see how NPRL compounds affected bEnd.3 brain endothelial cells and found BBB permeability coefficients (Fig. 3). The findings revealed that cytotoxicity did not affect the observed BBB permeability of the NPRL compounds, as the bEnd.3 cells exhibited no notable toxic effects at the concentrations tested (Fig. 4). Our research demonstrated the effectiveness of integrating computational predictive models with laboratory-based validation to enhance BBB permeability research in drug development. It is essential to use both these methods to find promising drug candidates for further study, as this study shows that the LLM model can accurately predict BBB permeability without being affected by cytotoxic effects [1].

Researchers continue to face challenges in verifying the mechanisms of action (MOA) for substances that cross the BBB and in validating the accuracy of predictive models without extensive *in vivo* testing [23,49,50]. Several critical factors influence the ability of a compound to penetrate the BBB, including lipophilicity, molecular weight, shape, and charge [51–55]. Additionally, a substance's permeability may be affected by its interactions with various cellular components, such as active or passive membrane transporters, metabolic enzymes, and efflux pumps [52,54,56,57]. Pathological conditions or therapeutic agents can also alter the effectiveness and distribution of compounds by modifying BBB permeability [58,59]. When utilizing *in vitro* models to assess BBB permeability, as in this study, it is essential to consider the physicochemical properties of the compounds, the presence of an unstirred water layer, and the characteristics of the filter [60,61]. These factors can significantly affect the permeability coefficients obtained in cell-based experiments. Table 2

The ADME parameters of NPRL compounds were analyzed to determine their pharmacokinetic characteristics (Fig. 5, ~~Table 1~~ and Supplementary Table 1 (https://www.biomedicinej.com/cgi/editor.cgi?article=1474&window=additional_files&context=biomedicine)). These findings provide valuable insights for the development of pharmaceutical

BioMedicine
2024;xx(xx):1–10

Table 2. ADME analysis of NPRL (NPRL 1–30) using AI/ML module.

Name	Intestinal absorption level	Solubility Level	CYP2D6 score[a]	Plasma Protein Binding (PPB) score[a]	AlogP98[a]	PSA 2D[a]
NPRL 1	Good	Good	−3.777	−0.526	1.847	43.531
NPRL 2	Good	Low	−2.420	1.485	3.133	35.160
NPRL 3	Good	Low	−3.319	1.108	3.133	35.160
NPRL 4	Good	Low	−4.024	0.090	3.173	35.160
NPRL 5	Good	Low	−4.813	0.167	2.670	44.091
NPRL 6	Good	Low	−2.554	5.820	4.165	35.160
NPRL 7	Good	Low	−1.619	1.550	4.046	35.160
NPRL 8	Good	Low	−2.817	2.375	3.841	35.160
NPRL 9	Good	Low	−3.608	−0.330	2.614	35.160
NPRL 10	Good	Low	−2.626	2.861	3.502	35.160
NPRL 11	Good	Good	−4.328	−0.813	2.375	44.091
NPRL 12	Good	Low	−3.920	1.076	3.173	35.160
NPRL 13	Good	Low	−4.282	2.662	3.619	35.160
NPRL 14	Good	Low	−2.819	0.272	3.117	44.091
NPRL 15	Good	Low	−2.196	2.535	4.532	35.160
NPRL 16	Good	Low	−2.497	3.348	3.580	35.160
NPRL 17	Good	Good	−4.125	1.033	2.410	44.091
NPRL 18	Good	Good	−5.625	−1.216	1.644	76.791
NPRL 19	Good	Low	−3.382	3.386	3.619	35.160
NPRL 20	Good	Low	−4.100	−0.093	2.670	44.091
NPRL 21	Good	Very low, but possible	−1.772	5.748	4.875	61.391
NPRL 22	Good	Low	−2.668	0.703	3.609	44.091
NPRL 24	Good	Low	−3.394	3.250	4.327	35.160
NPRL 25	Good	Good	−4.541	2.225	0.660	122.783
NPRL 26	Good	Low	−2.424	3.252	3.481	106.042
NPRL 27	Good	Low	−0.764	4.043	3.502	52.461
NPRL 28	Moderate	Low	−3.281	2.271	4.757	106.042
NPRL 29	Good	Low	−6.166	−3.413	2.137	112.450
NPRL 30	Moderate	Low	−7.498	−5.892	1.272	134.455

[a] ALogP 98 score of <5 and PSA score of <140 indicates good absorption and cell permeability, PPB score of 2.226 or less reflect highly bound (90%) to plasma protein, CYP2D6 score of <0.162 indicate non inhibitor of CYP2D6.

formulations, including those for oral and intravenous delivery, while also advancing the study of drug-metabolomics and pharmacokinetics (PK) and pharmacodynamics (PD). Our *in vitro* experimental results support computer-generated predictions regarding the BBB permeability of NPRL compounds, indicating that computational models can serve as reliable initial tools in new drug discovery. However, further *in vivo* research is needed to validate these predictions fully and to enhance our understanding of the mechanisms of action for potential clinical applications.

5. Conclusion

We conducted research that supports the numerous advantages of employing deep learning techniques to predict the permeability of the BBB. However, it is important to recognize that the current methods are still insufficient to completely elucidate the complex processes involved in drug-transport across the BBB. At present, we are unable to accurately differentiate between the therapeutic effects, adverse effects, and secondary outcomes resulting from a compound's ability to penetrate the BBB, which has significant implications for pharmacological studies. Future research will focus on experimental investigations using *in vivo* animal models and cellular assays to address these knowledge deficits. The objective of these endeavors is to clarify the MOA and explore the potential therapeutic applications of the identified compounds. This type of research is crucial for improving the precision of predictions made by deep learning models and broadening our understanding of pharmacokinetics at the BBB.

Author contributions

JSY, ETCH, KYKL and FJT were responsible for the overall conception and design.

DTB, SCT, CJC and TYL contributed to *in vitro* data analysis and interpretation. JSY, ETCH and KYKL performed the analysis of the *in silico* BBB permeability analysis and interpreted the data. KWC and YJC performed the analysis of the *in silico* ADME analysis by AI/ML module and interpreted

the data. All authors have read and approved the final version of the manuscript.

Data availability

All data generated or analyzed during this study are included in this published article.

Acknowledgement

We sincerely thank the Taiwan Web Service (TWS) to provide AIHPC for Large language model (LLM) training on Taiwania-2 for providing assistance and equipment for the present study. The authors would like to thank the Office of Research and Development, China Medical University (Taichung, Taiwan) for providing Medical Research Core Facilities to perform the experiments and data analysis.

Funding

This work was supported in part of the project Q4 (DMR-113-109) from China Medical University Hospital, Taiwan.

Conflicts of interest

The authors declare that they have no competing interests.

References

[1] Huang ETC, Yang JS, Liao KYK, Tseng WCW, Lee CK, Gill M, et al. Predicting blood-brain barrier permeability of molecules with a large language model and machine learning. Sci Rep 2024;14:15844.
[2] Dehnbostel FO, Dixit VA, Preissner R, Banerjee P. Non-animal models for blood-brain barrier permeability evaluation of drug-like compounds. Sci Rep 2024;14:8908.
[3] Edros R, Feng TW, Dong RH. Utilizing machine learning techniques to predict the blood-brain barrier permeability of compounds detected using LCQTOF-MS in Malaysian Kelulut honey. SAR QSAR Environ Res 2023;34:475–500.
[4] Meng F, Xi Y, Huang J, Ayers PW. A curated diverse molecular database of blood-brain barrier permeability with chemical descriptors. Sci Data 2021;8:289.
[5] Zhang X, Liu T, Fan X, Ai N. In silico modeling on ADME properties of natural products: classification models for blood-brain barrier permeability, its application to traditional Chinese medicine and in vitro experimental validation. J Mol Graph Model 2017;75:347–54.
[6] Kato R, Zeng W, Siramshetty VB, Williams J, Kabir M, Hagen N, et al. Development and validation of PAMPA-BBB QSAR model to predict brain penetration potential of novel drug candidates. Front Pharmacol 2023;14:1291246.
[7] Zhang W, Oh JH, Zhang W, Rathi S, Le J, Talele S, et al. How much is enough? Impact of efflux transporters on drug delivery leading to efficacy in the treatment of brain tumors. Pharm Res (N Y) 2023;40:2731–46.
[8] Patel MM, Goyal BR, Bhadada SV, Bhatt JS, Amin AF. Getting into the brain: approaches to enhance brain drug delivery. CNS Drugs 2009;23:35–58.
[9] Pardridge WM. Brain drug targeting and gene technologies. Jpn J Pharmacol 2001;87:97–103.
[10] Stanimirovic D, Kemmerich K, Haqqani AS, Farrington GK. Engineering and pharmacology of blood-brain barrier-permeable bispecific antibodies. Adv Pharmacol 2014;71:301–35.
[11] Brunden KR, Ballatore C, Lee VM, Smith 3rd AB, Trojanowski JQ. Brain-penetrant microtubule-stabilizing compounds as potential therapeutic agents for tauopathies. Biochem Soc Trans 2012;40:661–6.
[12] Yan L, Guo MS, Zhang Y, Yu L, Wu JM, Tang Y, et al. Dietary plant polyphenols as the potential drugs in neurodegenerative diseases: current evidence, advances, and opportunities. Oxid Med Cell Longev 2022;2022:5288698.
[13] Kemper EM, Boogerd W, Thuis I, Beijnen JH, van Tellingen O. Modulation of the blood-brain barrier in oncology: therapeutic opportunities for the treatment of brain tumours? Cancer Treat Rev 2004;30:415–23.
[14] Siegal T, Zylber-Katz E. Strategies for increasing drug delivery to the brain: focus on brain lymphoma. Clin Pharmacokinet 2002;41:171–86.
[15] Bickel U, Yoshikawa T, Pardridge WM. Delivery of peptides and proteins through the blood-brain barrier. Adv Drug Deliv Rev 2001;46:247–79.
[16] Ding Y, Jiang X, Kim Y. Relational graph convolutional networks for predicting blood-brain barrier penetration of drug molecules. Bioinformatics 2022;38:2826–31.
[17] Vatansever S, Schlessinger A, Wacker D, Kaniskan HU, Jin J, Zhou MM, et al. Artificial intelligence and machine learning-aided drug discovery in central nervous system diseases: state-of-the-arts and future directions. Med Res Rev 2021;41:1427–73.
[18] Guixer B, Arroyo X, Belda I, Sabido E, Teixido M, Giralt E. Chemically synthesized peptide libraries as a new source of BBB shuttles. Use of mass spectrometry for peptide identification. J Pept Sci 2016;22:577–91.
[19] Subramanian K. truPK – human pharmacokinetic models for quantitative ADME prediction. Expet Opin Drug Metabol Toxicol 2005;1:555–64.
[20] Liu X, Tu M, Kelly RS, Chen C, Smith BJ. Development of a computational approach to predict blood-brain barrier permeability. Drug Metab Dispos 2004;32:132–9.
[21] Zhang L, Zhu H, Oprea TI, Golbraikh A, Tropsha A. QSAR modeling of the blood-brain barrier permeability for diverse organic compounds. Pharm Res (N Y) 2008;25:1902–14.
[22] Wang Z, Yang H, Wu Z, Wang T, Li W, Tang Y, et al. In silico prediction of blood-brain barrier permeability of compounds by machine learning and resampling methods. ChemMedChem 2018;13:2189–201.
[23] Miao R, Xia LY, Chen HH, Huang HH, Liang Y. Improved classification of blood-brain-barrier drugs using deep learning. Sci Rep 2019;9:8802.
[24] Liang L, Liu Z, Yang X, Zhang Y, Liu H, Chen Y. Prediction of blood-brain barrier permeability using machine learning approaches based on various molecular representation. Mol Inform 2024:e202300327.
[25] Kumar R, Sharma A, Alexiou A, Bilgrami AL, Kamal MA, Ashraf GM. DeePred-BBB: a blood brain barrier permeability prediction model with improved accuracy. Front Neurosci 2022;16:858126.
[26] Park JS, Choe K, Khan A, Jo MH, Park HY, Kang MH, et al. Establishing Co-culture blood-brain barrier models for different neurodegeneration conditions to understand its effect on BBB integrity. Int J Mol Sci 2023;24.
[27] McMillin MA, Frampton GA, Seiwell AP, Patel NS, Jacobs AN, DeMorrow S. TGFbeta1 exacerbates blood-brain barrier permeability in a mouse model of hepatic encephalopathy via upregulation of MMP9 and downregulation of claudin-5. Lab Invest 2015;95:903–13.
[28] Huang CP, Liu LC, Lu HL, Shyr CR. Effects of hepatocyte growth factor on porcine mammary cell growth and senescence. Biomedicine 2023;13:13–21.
[29] Huang L, Wu X, Fu X, Wang H, Tang B, Xiao Y, et al. Ligand based 3D-QSAR model, pharmacophore, molecular docking

ORIGINAL ARTICLE

and ADME to identify potential fibroblast growth factor receptor 1 inhibitors. J Biomol Struct Dyn 2022;40:7584–97.
[30] Han Y, Zhang J, Hu CQ, Zhang X, Ma B, Zhang P. In silico ADME and toxicity prediction of ceftazidime and its impurities. Front Pharmacol 2019;10:434.
[31] Cheng A, Merz Jr KM. Prediction of aqueous solubility of a diverse set of compounds using quantitative structure-property relationships. J Med Chem 2003;46:3572–80.
[32] Egan WJ, Lauri G. Prediction of intestinal permeability. Adv Drug Deliv Rev 2002;54:273–89.
[33] Jensen BF, Vind C, Padkjaer SB, Brockhoff PB, Refsgaard HH. In silico prediction of cytochrome P450 2D6 and 3A4 inhibition using Gaussian kernel weighted k-nearest neighbor and extended connectivity fingerprints, including structural fragment analysis of inhibitors versus noninhibitors. J Med Chem 2007;50:501–11.
[34] Wessel MD, Jurs PC, Tolan JW, Muskal SM. Prediction of human intestinal absorption of drug compounds from molecular structure. J Chem Inf Comput Sci 1998;38:726–35.
[35] Oie S, Tozer TN. Effect of altered plasma protein binding on apparent volume of distribution. J Pharmaceut Sci 1979;68:1203–5.
[36] Yasuhara M, Fujiwara J, Kitade S, Katayama H, Okumura K, Hori R. Effect of altered plasma protein binding on pharmacokinetics and pharmacodynamics of propranolol in rats after surgery: role of alpha-1-acid glycoprotein. J Pharmacol Exp Therapeut 1985;235:513–20.
[37] Hour MJ, Tsai FJ, Lai IL, Tsao JW, Chiang JH, Chiu YJ, et al. Efficacy of HMJ-38, a new quinazolinone analogue, against the gemcitabine-resistant MIA-PaCa-2 pancreatic cancer cells. Biomedicine 2023;13:20–31.
[38] Tsai SC, Yang JS, Lu CC, Tsai FJ, Chiu YJ, Kuo SC. MTH-3 sensitizes oral cancer cells to cisplatin via regulating TFEB. J Pharm Pharmacol 2022;74:1261–73.
[39] Chiu YJ, Yang JS, Tsai FJ, Chiu HY, Juan YN, Lo YH, et al. Curcumin suppresses cell proliferation and triggers apoptosis in vemurafenib-resistant melanoma cells by downregulating the EGFR signaling pathway. Environ Toxicol 2022;37:868–79.
[40] Kim K, Jang A, Shin H, Ye I, Lee JE, Kim T, et al. Concurrent optimizations of efficacy and blood-brain barrier permeability in new macrocyclic LRRK2 inhibitors for potential Parkinson's disease therapeutics. J Med Chem 2024;67:7647–62.
[41] Hutter MC. In silico prediction of drug properties. Curr Med Chem 2009;16:189–202.
[42] Ecker GF, Noe CR. In silico prediction models for blood-brain barrier permeation. Curr Med Chem 2004;11:1617–28.
[43] Rao M, Nassiri V, Alhambra C, Snoeys J, Van Goethem F, Irrechukwu O, et al. AI/ML models to predict the severity of drug-induced liver injury for small molecules. Chem Res Toxicol 2023;36:1129–39.
[44] Maltarollo VG, Gertrudes JC, Oliveira PR, Honorio KM. Applying machine learning techniques for ADME-Tox prediction: a review. Expet Opin Drug Metabol Toxicol 2015;11:259–71.
[45] Keshavarzi Arshadi A, Salem M, Collins J, Yuan JS, Chakrabarti D. DeepMalaria: artificial intelligence driven discovery of potent antiplasmodials. Front Pharmacol 2019;10:1526.
[46] Demir-Kavuk O, Bentzien J, Muegge I, Knapp EW. DemQSAR: predicting human volume of distribution and clearance of drugs. J Comput Aided Mol Des 2011;25:1121–33.
[47] Jiang J, Ouyang D, Williams 3rd RO. Predicting glass-forming ability of pharmaceutical compounds by using machine learning technologies. AAPS PharmSciTech 2023;24:103.
[48] Fukunishi Y. Structure-based drug screening and ligand-based drug screening with machine learning. Comb Chem High Throughput Screen 2009;12:397–408.
[49] Gao Z, Chen Y, Cai X, Xu R. Predict drug permeability to blood-brain-barrier from clinical phenotypes: drug side effects and drug indications. Bioinformatics 2017;33:901–8.
[50] Boswell CA, Mundo EE, Johnstone B, Ulufatu S, Schweiger MG, Bumbaca D, et al. Vascular physiology and protein disposition in a preclinical model of neurodegeneration. Mol Pharm 2013;10:1514–21.
[51] Segan S, Penjisevic J, Sukalovic V, Andric D, Milojkovic-Opsenica D, Kostic-Rajacic S. Investigation of lipophilicity and pharmacokinetic properties of 2-(methoxy)phenylpiperazine dopamine D2 ligands. J Chromatogr, B: Anal Technol Biomed Life Sci 2019;1124:146–53.
[52] Shimizu F, Nakamori M. Blood-brain barrier disruption in neuroimmunological disease. Int J Mol Sci 2024;25.
[53] Rehman IU, Park JS, Choe K, Park HY, Park TJ, Kim MO. Overview of a novel osmotin abolishes abnormal metabolic-associated adiponectin mechanism in Alzheimer's disease: peripheral and CNS insights. Ageing Res Rev 2024;100:102447.
[54] Zapata-Acevedo JF, Mantilla-Galindo A, Vargas-Sanchez K, Gonzalez-Reyes RE. Blood-brain barrier biomarkers. Adv Clin Chem 2024;121:1–88.
[55] Asimakidou E, Tan JKS, Zeng J, Lo CH. Blood-brain barrier-targeting nanoparticles: [illegible] biomedical applications in [illegible] maceuticals 2024;17.
[56] Virtanen PS, Ortiz KJ, [illegible] Richardson AM. Blood-br[illegible] treatment of primary brain t[illegible] decade. Curr Oncol Rep 2024;26:236–49.
[57] Wakonigg Alonso C, McElhatton F, O'Mahony B, Campbell M, Pollak TA, Stokes PRA. The blood-brain barrier in bipolar disorders: a systematic review. J Affect Disord 2024;361:434–44.
[58] Varghese SM, Patel S, Nandan A, Jose A, Ghosh S, Sah RK, et al. Unraveling the role of the blood-brain barrier in the pathophysiology of depression: recent advances and future perspectives. ~~Mol Neurobiol 2024.~~ Q5
[59] Anwarkhan S, Koilpillai J, Narayanasamy D. Utilizing multifaceted approaches to target drug delivery in the brain: from nanoparticles to biological therapies. Cureus 2024;16:e68419.
[60] Cornelissen FMG, Markert G, Deutsch G, Antonara M, Faaij N, Bartelink I, et al. Explaining blood-brain barrier permeability of small molecules by integrated analysis of different transport mechanisms. J Med Chem 2023;66:7253–67.
[61] Tsinman O, Tsinman K, Sun N, Avdeef A. Physicochemical selectivity of the BBB microenvironment governing passive diffusion–matching with a porcine brain lipid extract artificial membrane permeability model. Pharm Res (N Y) 2011;28:337–63.
[62] Shaker B, Yu MS, Song JS, Ahn S, Ryu JY, Oh KS, et al. LightBBB: computational prediction model of blood-brain-barrier penetration based on LightGBM. Bioinformatics 2021;37:1135–9.

Mol Neurobiol. 2024 May 10. doi: 10.1007/s12035-024-04205-5. Online ahead of print

AUTHOR QUERY FORM

**Journal:** BIOMED<br><br>**Article Number:** 1474	**Please share your corrections or revision and any artwork related corrections to Digital common site.**<br><br>**E-mail: publishing.services@cs.bepress.com**

Dear Author,

Please check your proof carefully and mark all corrections at the appropriate place in the proof (e.g., by using on-screen annotation in the PDF file) or compile them in a separate list. **It is crucial that you NOT make direct edits to the PDF using the editing tools as doing so could lead us to overlook your desired changes.** Note: if you opt to annotate the file with software other than Adobe Reader then please also highlight the appropriate place in the PDF file. To ensure fast publication of your paper please return your corrections within 48 hours.

Any queries or remarks that have arisen during the processing of your manuscript are listed below and highlighted by flags in the proof.

Location in article	Query / Remark: Click on the Q link to find the query's location in text<br>Please insert your reply or correction at the corresponding line in the proof
Q1	Kindly provide revised date for this article.
Q2	Please check the short title that has been created, or suggest an alternative one.
Q3	**Table 2** was not cited in the text. Please check that the citation(s) suggested are in the appropriate place, and correct if necessary.
Q4	Have we correctly interpreted the following funding source(s) and country names you cited in your article: China Medical University Hospital, Taiwan?
Q5	Please add the **volume number and page range** for the bibliography in reference [58].
Q6	Please confirm that given names and surnames have been identified correctly and are presented in the desired order and please carefully verify the spelling of all authors' names.

Thank you for your assistance.
