# Supplementary material for: Artificial intelligence-driven prediction and validation of blood–brain barrier permeability and absorption, distribution, metabolism, excretion profiles in natural product research laboratory compounds
Source: Biomedicine (Taipei). 2024 Dec 1;14(4):82–91. doi: 10.37796/2211-8039.1474 (PMC11703399; doi:10.37796/2211-8039.1474)
Supplement: Supplementary file 2 [file bmed-14-04-082-s002.docx]

Dear Editor,

We and our team members would like to express our sincere gratitude for accepting our paper, "Artificial intelligence-driven prediction and validation of blood-brain barrier permeability and absorption, distribution, metabolism, excretion profiles in Natural Product Research Laboratory compounds" for publication in Biomedicine (BIOMED_1474). In response to the reviewers' feedback, we have carefully implemented all suggested changes and revised the manuscript. After thorough proofreading to ensure accuracy and readability, we are **c**onfident that the revised version is ready for publication. The review process has significantly enhanced the quality of our work, and we greatly appreciate the insight.

We would like to reiterate our thanks for the opportunity to contribute to the field of Biomedicine. We eagerly anticipate the impact of our research when featured in the upcoming issue.

Sincerely,

Dr. Jai-Sing Yang

Department of Medical Research

China Medical University Hospital

China Medical University, Taichung, Taiwan

Email: jaisingyang@gmail.com
